# Multi-Stage buck strategy for power management of triboelectric nanogenerators

**DOI:** 10.1038/s41467-026-78002-0

**Published:** 2026-09-29

**Authors:** Yikui Gao, Lixia He, Xiangyu Hu, Di Liu, Linglin Zhou, Hanlin Sun, Xinyuan Li, Xianggang Dai, Wenyan Qiao, Bingzhe Jin, Zhong Lin Wang, Jie Wang

**Affiliations:** 1 Beijing Key Laboratory of High-Entropy Energy Materials and Devices, Beijing Institute of Nanoenergy and Nanosystems, Chinese Academy of Sciences, Beijing, 101400 P. R. China; 2 School of Nanoscience and Engineering, University of Chinese Academy of Sciences, Beijing, 100049 P. R. China; 3 Department of Mechanical Engineering, The Hong Kong Polytechnic University, Hong Kong, 999077 P. R. China; 4 Department of Mechanical Engineering, Tsinghua University, Beijing, 100084 P. R. China; 5 Guangzhou Institute of Blue Energy, Knowledge City, Huangpu District, Guangzhou, 510555 P. R. China; 6 Yonsei Frontier Lab, Yonsei University, Seoul, 03722 Republic of Korea

**Keywords:** Devices for energy harvesting, Renewable energy

## Abstract

Triboelectric nanogenerators (TENGs) offer transformative potential for self-powered systems, yet their real-world applicability faces a key bottleneck as their performance and efficiency suffer dramatic degradation below 5 V that the operational threshold for most electronics when integrated with conventional power management circuits. Here, we overcome this limitation through the multi-stage buck strategy that synergistically combines dual-stage voltage conversion circuit with TENG’s output characteristic by the *Q-V* curve, enabling precise voltage regulation at critical nodes. The strategy achieves approximately 80% circuit efficiency at the low-voltage range, with 90.9% in the primary circuit and 93.9% in the secondary circuit. Concurrently, the current and power density reach 5.19 A/m^2^ and 9.70 W/m^2^ at 1.87 V, which is nearly an order of magnitude improvement over best prior work. Demonstrated in self-powered hydrogen production system, this approach achieves a record gas yield of 16.5 mL/(min·m^2^), resolving the challenge of low-voltage inefficiency in self-powered systems. Additionally, it is capable of powering a mobile phone. Our work establishes a foundational framework for implementing efficient TENG-powered devices in Internet of Things networks and industrial applications, bridging the gap between energy harvesting technologies and actual energy demands.

## Abstract (web summary)

The work presents a multi-stage step-down power management circuit for triboelectric nanogenerators, regulating the high-voltage pulsed output into usable power, reducing losses during conversion, and improving achievable current delivery for self-powered electronics.

## Introduction

The advent of self-powered systems has revolutionized modern energy systems by addressing the crucial issue of sustainable power supply for widespread sensor networks and electrochemistry systems through advanced energy conversion technologies that harvest ambient mechanical energy, achieving electrically self-sufficient systems and eliminating reliance on conventional batteries and power sources^1–6^. Triboelectric nanogenerators (TENGs), with its advantages of simple fabrication, cost-effectiveness, high energy conversion efficiency, and wide material options, can convert mechanical energy into electrical energy and provide a reliable and sustainable energy solution for distributed electronics^7–9^. However, the inherent output characteristics of TENGs, which generate high voltage but relatively low current, do not align well with the low-voltage power requirements of most electronic devices. Therefore, to fully unlock the potential of TENGs and facilitate the continuous advancement of IoT technologies, effective power management circuits (PMCs) are crucial.

The PMCs for TENGs have primarily utilized classic and efficient circuit architectures, such as inductor-capacitor (LC) circuits^10–16^, transformer-coupled circuits^17,18^, and switched-capacitor convertor (SCC)^19,20^. The optimization strategies for these circuits have consistently adhered to a core principle that achieves comprehensive efficiency improvement by minimizing energy loss within the PMC’s electronic components^12,17,21–25^. For instance, Wang et al. optimized the design of transformers and inductors to provide guidance for LC and inductor-coupled circuits^11,17^; Liu et al. developed a fractal-design SCC to mitigate voltage drop of conventional SCC^19^; Wu et al. reduced charge and voltage losses at the switch while achieving a stable voltage output by commercial chips^25^; Zhang et al. proposed a dual-loop circuit to minimizing rectifier bridge energy loss^23^; and our prior work focused on reducing capacitor leakage current to increase efficiency^13^. Through the optimization of electronic components, reported circuit efficiencies have reached as high as 90%, significantly enhancing the competitiveness of TENG-based systems. However, these efforts remain confined to component-level optimization and have yet to recognize, let alone resolve, two systemic challenges inherent to the TENG-PMC interface (Note S1). First, within the low-voltage range (below 5 V) where most commercial electronics and sensors operate, the actual circuit efficiency is significantly lower than the reported optimal values (Fig. S1). This discrepancy becomes increasingly pronounced as the output voltage decreases. Furthermore, due to insufficient theoretical understanding of the synergy mechanism between TENG output characteristics and the PMCs, a direct application of high-efficiency circuits often leads to a mismatch, causing the actual performance to fall to less than one-tenth of the reported optimal performance in some works (Table S1), severely limiting the real-world applicability of TENG^26–32^. Therefore, accurately understanding these issues and developing a new power management paradigm that is compatible with the TENG output characteristics and maintains high efficiency in the low-voltage range has become an urgent problem to be solved.

Here, we propose a multi-stage buck strategy for power management of TENGs, which aims at regulate the operating voltages at critical nodes within the circuit and thereby address the inherent challenges in conventional PMCs. The multi-stage buck strategy encompasses the design of the primary buck circuit and the secondary buck circuit. By the charge-voltage (*Q-V*) curve of TENGs, we establish the synergy mechanism between the output characteristics of the TENG and the PMCs, and precisely determine the optimal switching operating voltage for the primary buck circuit, thereby maximizing the output energy of TENG. Furthermore, the results of simulation calculations and experiments reveal that increasing the operating voltage of the secondary buck circuit can subsequently reduce the energy wastage in the form of joule heating caused by *V*_diode_ (voltage drop of diode) and *R*_int_ (internal resistance in the circuit), thereby significantly enhancing the efficiency of the circuit and achieving approximately 80% circuit efficiency in the low voltage range. In the application of rotary mode TENG, we have achieved outstanding current density of 5.19 A/m^2^ and power density of 9.70 W/m^2^ at 1.87 V, which represents nearly an order of magnitude improvement compared to the best previously reported value of 0.71 A/m^2^ and 1.41 W/m^2^. Furthermore, the efficiency of both the energy system and the electrochemical system constructed by TENG with this strategy has been significantly improved. In particular, the hydrogen production rate of the self-power hydrogen generation system reaches an impressive 16.5 mL/(min·m^2^), also marking a nearly order of magnitude higher than the prior best of 2.5 mL/(min·m^2^). The multi-stage buck strategy presented here lays the foundation for developing an efficient power management and facilitating the further industrialization of TENGs technology.

## Results

### Multi-stage buck strategy

**Fig. 1:**
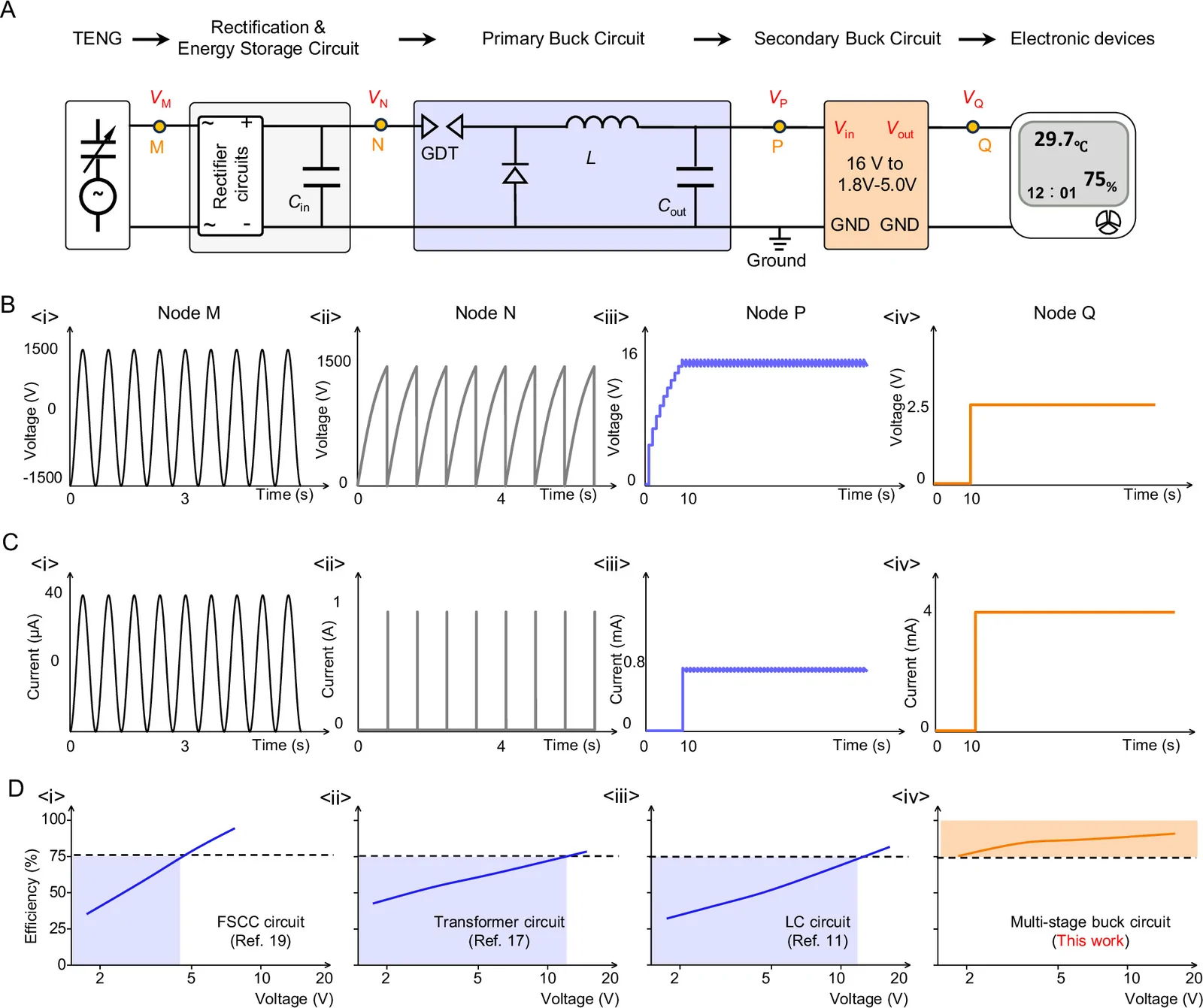
The multi-stage buck strategy. **A** The workflow of the multi-stage buck circuit. The TENG collects mechanical energy from the environment and converts its high-voltage intrinsic output into the output required by the application through a rectification circuit, a primary buck circuit, and a secondary buck circuit. **B** The voltage at <i> node M, <ii> node N, <iii> node P, and <iv> node Q. **C** The current at <i> node M, <ii> node N, <iii> node P, and <iv> node Q. This work achieves high performance and efficiency of the TENG and power management circuit by regulating the operating voltages at nodes N and P. **D** The efficiency of the <i> FSCC circuit, <ii> transformer circuit, <iii> LC circuit and <iv> multi-stage buck circuit at different output voltages. Source data are provided as a Source Data file.

The multi-stage buck circuit for TENGs comprises three functional modules (Fig. 1A). The first one is the rectification and energy storage circuit, rectifying the alternating current (AC) output of TENG (Fig. 1B<i> and Fig. 1C<i > ) into direct current (DC) output, while storing energy in the input capacitor (*C*_in_) to serve as the excitation source for the primary buck circuit. The second module is the primary buck circuit, where the fundamental units include a switch (GDT, gas discharge tube), a freewheeling diode, an inductor, and the output capacitor (*C*_out_). When the nodal voltage *V*_N_ (the input voltage of the primary circuit or the threshold voltage of GDT) reaches the threshold voltage of the GDT, the switch activates, releasing the energy stored in *C*_in_ (Fig. 1B<ii > ) and generating a high pulse current (Fig. 1C<ii > ). The objective of primary buck circuit is to reduce the *V*_N_ to an intermediate voltage *V*_P_ (the output voltage of the primary circuit and the input voltage of the secondary circuit (Fig. 1B<iii > ), while producing a relatively stable high output current (Fig. 1C<iii > ). The third module is the secondary buck circuit, with its core being a commercial integrated circuit chip. Its crucial mission is to further regulates the *V*_P_ to the target output voltage *V*_Q_ (the output voltage of the secondary circuit, Fig. 1B<iv> and Fig. 1C<iv > ). As shown in Fig. 1D, the multi-stage buck circuit designed in this work exhibits an efficiency of over 75% in the low-voltage range, outperforming circuits designed in other works. Although similar circuit architectures have been reported in previous works^25^, the core issues in the TENG field have not been clearly understood and resolved (Note S2–3). This high efficiency of multi-stage buck circuit is largely attributed to the crucial regulation of *V*_N_ and *V*_P_ instead of circuit architecture. Specifically, *V*_N_ plays a vital role in determining the energy extraction efficiency from the TENG to *C*_in_. Meanwhile, *V*_P_ ensures optimal circuit efficiency for the primary circuit. Detailed discussions on these aspects are presented in the following parts.

### The structure and output characteristics of TENG

**Fig. 2:**
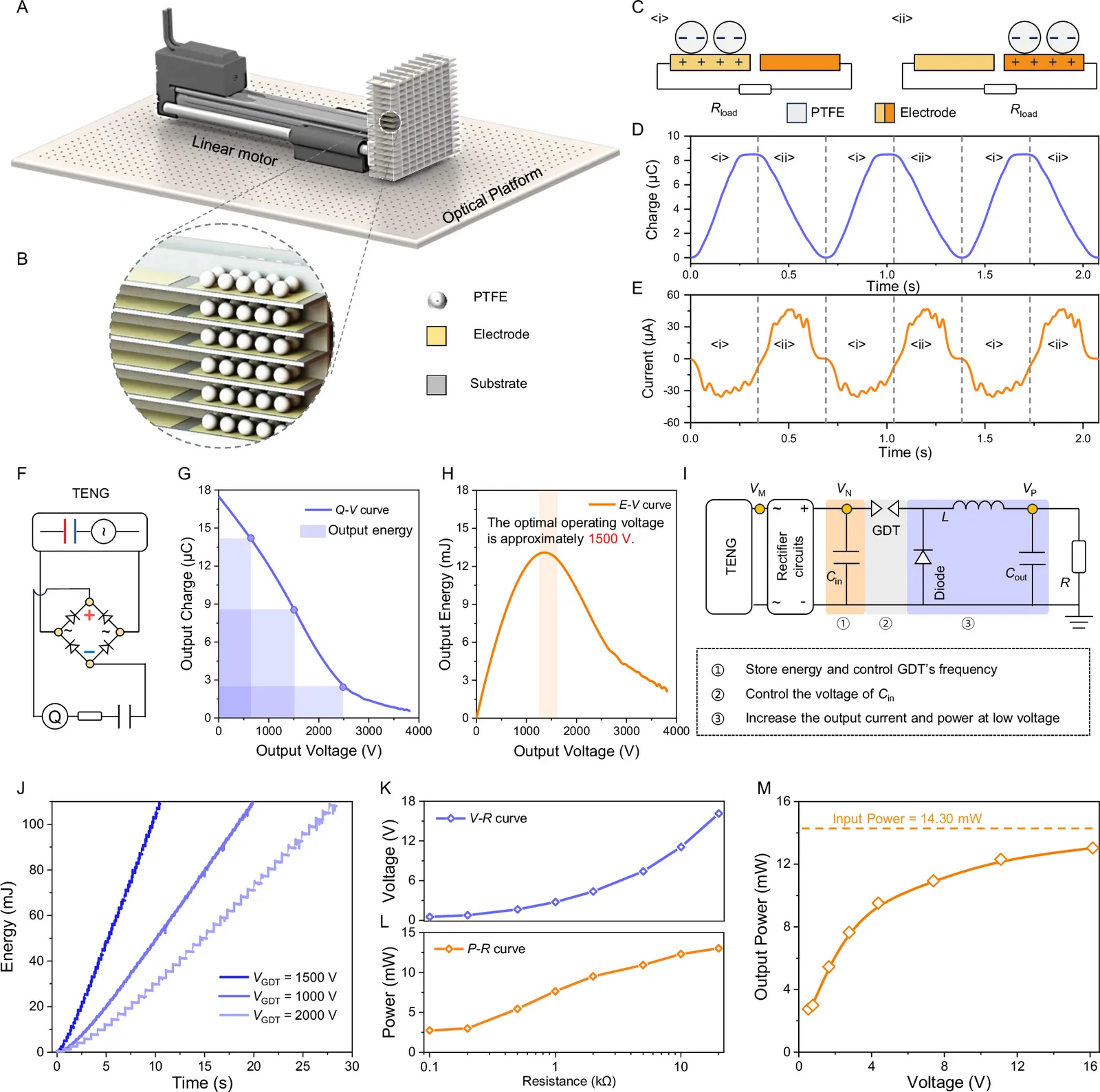
The primary buck circuit. **A**, **B** The structural diagrams of the rolling free-standing mode TENG. **C** The working principle. **D** The output charge. **E** The output current. **F** The schematic diagram of the circuit for testing the *Q-V* curve. The test capacitance is 22 nF. **G** The *Q-V* curve. **H** The *E-V* curve. The approximate optimal operating voltage of the primary circuit is approximately 1500 V. **I** Schematic diagram of the primary circuit. **J** The energy curve of charging the *C*_out_ to 15 V at different operating voltages. **K** The output voltage and **L** output power at different loads. **M** The output power of the primary buck circuit at different output voltages. Source data are provided as a Source Data file.

The basic unit of device employed in this paper is designed with rolling mode free-standing TENG (FS-TENG), which not only demonstrates excellent stability in previous works but also exhibits high efficiency in converting mechanical energy into electrical energy, opening up broad prospects for effective ocean energy harvesting^33^. Here, polytetrafluoroethylene (PTFE) sphere is used as the triboelectric material for this device, due to its excellent triboelectrification performance and stability (Note S4)^34–36^. The detailed structure of TENG is shown in the Fig. 2A, while Fig. 2B presents a magnified view of the device’s local details. In this work, the linear motor is utilized to replace wave-driven forces, driving the device to perform regular mechanical motions (relevant motion parameters are provided in the Fig. S3). It is worth noting that, mechanical inputs (such as, wave energy and wind energy) are always irregular in practical applications. This issue can be addressed either by designing mechanical structures to stabilize the mechanical input, or by implementing circuit design to regulate the output power (Note S5)^25,37–40^. The subsequent experiment results would be demonstrated this conclusion from circuit design. The working principle of this device is illustrated in Fig. 2C: PTFE spheres accumulate surface charges due to triboelectrification effect and subsequently undergo reciprocating motion driven by mechanical force. It causes the two electrodes to induce opposite charges during movement, generating an alternating current signal. The specific output charge and output current are shown in Fig. 2D and Fig. 2E, while maximum open-circuit voltage is presented in the Fig. S4. As shown in Fig. S5, the rolling mode FS-TENG maintains an almost unchanged output charge after 30,000 operational cycles and exhibits excellent stability.

### Regulation of input voltage for primary buck circuit

Herein, we utilize the *Q-V* characteristic curve of the TENG to assist in regulating the nodal voltage *V*_N_, so as to ensure that the primary buck circuit achieves optimal matching with the TENG^41^. The rationale of this method was substantiated by a derivation based on the energy cycle collapse process of the TENG (Fig. S6 and Note S6). Furthermore, the validity of this method has been confirmed by experimental results presented in subsequent experiments. The *Q-V* curve of TENGs reflects the output charge generated within a single motion cycle at different output voltages, which can intuitively determine the performance of TENGs (Note S7 and Fig. S7). The testing circuit and method of the *Q-V* characteristic curve for TENG is shown in Fig. 2F, Fig. S8 and Note S8, and the product of the coordinates of any point on the *Q-V* curve represents the output energy at that voltage (Fig. 2G). Figure 2H indicates that the output energy of TENG reaches the maximum when the *V*_N_ is approximately 1,500 V, thus we set the operating voltage of the switch close to this value when designing the primary buck circuit.

Here, we selected the LC circuit as the core structure for the primary buck circuit, as shown in Fig. 2I. In electric component selection, we strictly adhered to design requirements for voltage, current, and energy of circuit. Firstly, the *C*_in_, GDT, freewheeling diode, and inductor must all withstand at least 1,500 V, and the high-voltage nodes of TENG and PMCs also require proper insulation (Fig. S9 and Note S9). Secondly, the conduction current of the freewheeling diode must be greater than the maximum current (*I*_max_) during the LC circuit’s working process.

1 $${I}_{\max}={V}_{{{\rm{in}}}}\sqrt{\frac{{C}_{{\mbox{out}}}{C}_{{\mbox{in}}}}{{{\mbox{L}}}({{\mbox{C}}}_{{\mbox{in}}}+{{\mbox{C}}}_{{\mbox{out}}})}}$$

2 $${I}_{{{{\rm{diode}}}}}\cap {I}_{{{{\rm{sat}}}}}\ge {I}_{\max }$$

Where *I*_diode_ is the maximum forward current of the diode, *I*_sat_ is the saturation current of the inductor (the detailed analysis is provided in the Note S10 and Figs. S10-11). Thirdly, in order to minimize energy loss in the form of joule heating, it is necessary to reduce internal resistance and capacitor leakage current (the detailed analysis is provided in the Note S11, the detailed parameters of the electric component are shown in the Fig. S12).

By adjusting the threshold voltage of GDT to 1000 V, 1500 V, and 2000 V, we found that the energy storage rate of the capacitor was maximized when the threshold voltage of GDT was set to 1,500 V (Fig. 2J and Fig. S13). The experiment results confirm the effectiveness of the *Q-V* characteristic curve in guiding circuit design. Figure S14 shows that the GDT operates at a frequency (*f*_GDT_) of approximately 28.9 Hz. Based on the frequency, the capacitance of the *C*_in_ and the formula (3), we can calculate the circuit’s input power (*P*_in_) to be 14.3 mW.

3 $${P}_{{{{\rm{in}}}}}={0.5}{*}{C}_{{{{\rm{in}}}}}{*}{V}_{{{{\rm{in}}}}}^2 {\ast }\,{f}_{{{{\rm{GDT}}}}}$$

By adjusting the external load, we obtained the output voltage (Fig. 2K and Fig. S15A), output current (Fig. S15B), and output power (Fig. 2L, Fig. S15C, and Note S12). These results indicate that as the output voltage increases, the output power also increases (Fig. 2M). When the output voltage reaches 16 V, the output power (*P*_out_) attains 13.0 mW, and the efficiency of the primary buck circuit achieves 90.9%.

4 $$\eta (V)={P}_{{{{\rm{out}}}}}(V)/{P}_{{{{\rm{in}}}}}$$

However, we observed that within the voltage range below 5 V (the operating voltages of most electrochemical reactions, sensors, and battery energy storage systems.), the circuit efficiency decreases significantly. This indicates that TENG still faces challenges in achieving efficient energy conversion and utilization in fields such as self-power electrochemistry and self-power energy systems. Consequently, there is an urgent need to explore alternative circuit designs or optimization strategies to enhance the efficiency within this crucial voltage range.

### The reasons for low efficiency in primary circuits

**Fig. 3:**
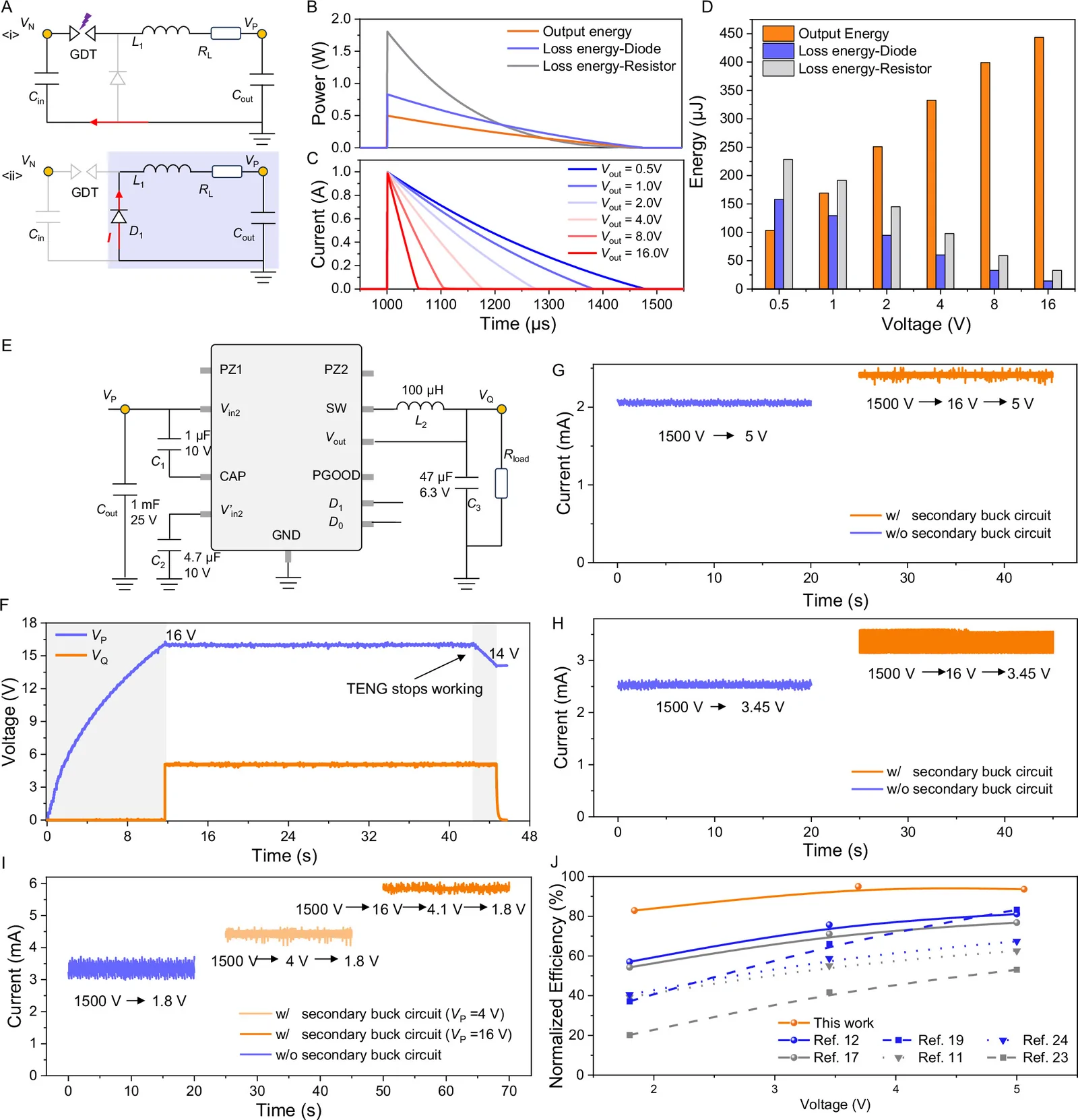
The secondary buck circuit. **A** The schematic diagrams of the primary buck circuit at different stages. Energy loss primarily occurs in the second stage (Fig. 3A<ii >). **B** The output energy and energy consumed by internal impedance when the output voltage is 0.5 V. **C** The current of the primary buck circuit as the output voltage gradually increases from 0.5 V to 16 V. **D** The comparison of output energy and energy consumed by internal impedance as the output voltage gradually increases from 0.5 V to 16 V. **E** The schematic diagram of the secondary buck circuit. **F** The input voltage *V*_P_ and output voltage *V*_Q_ of the secondary buck circuit. **G**–**I** Comparison of output currents when the output voltage *V*_Q_ is set to 5 V, 3.45 *V*, and 1.8 V (without/with the secondary circuit, measured as 5.13/5.06 V, 3.66/3.69 V, and 1.82/1.86/1.84 V, respectively). (J) The normalization efficiency of different works under low-voltage range. Source data are provided as a Source Data file.

In the LC circuit, when the switch is turned off, the current in the inductor does not disappear immediately due to the inductor’s property of maintaining a constant current (Fig. 3A). At this moment, the freewheeling diode conducts to provide a path for the inductor’s current to continue flowing (Fig. 3A <ii > , and the detailed analysis is shown in Note S10.). Our simulation analysis revealed that the primary cause of the LC circuit’s efficiency degradation is the significant increase in energy loss during this process at low voltages (Fig. S16, Fig. S17 and Note S13). When the diode conducts, a voltage drop is generated across its terminals, typically denoted as *V*_diode_. The greater the *V*_diode_, the more energy dissipates (Fig. S16H–J). At the same time, there is inevitably internal resistance (*R*_int_) in the circuit (particularly the internal resistance of inductors), and the energy loss caused by *R*_int_ also cannot be ignored. Consequently, the input energy (*E*_in_) is primarily divided into three components: the energy dissipated in the diode (*E*_diode_), the energy dissipated in the internal resistance (*E*_R_), and the energy used for storage and subsequent utilization (*E*_out_).

5 $${E}_{{{{\rm{in}}}}}={E}_{{{{\rm{diode}}}}}+{E}_{{{{\rm{R}}}}}+{E}_{{{{\rm{out}}}}}$$

6 $${P}_{{{{\rm{in}}}}}={P}_{{{{\rm{diode}}}}}+{P}_{{{{\rm{R}}}}}+{P}_{{{{\rm{out}}}}}=I(t)*({V}_{{{{\rm{diode}}}}}+{V}_{{{{\rm{R}}}}}+{V}_{{{{\rm{out}}}}})$$

Where *P*_in_ and *P*_out_ represent the input and output power of LC circuit, *P*_R_ and *P*_diode_ represent the power loss on internal resistance and diode of LC circuit, *V*_R_ represent the voltage of internal resistance, *I*(*t*) represent the current in the circuit shown in Fig. 3A <ii > .

7 $${I}\left({t}\right)=\left({I}_{{{\mathrm{max}}}}+\frac{{{V}}_{{{\rm{diode}}}}+{{V}}_{{{\rm{out}}}}}{{R}_{{{\rm{int}}}}}\right){{{\rm{e}}}}^{-\frac{{R}_{{{\rm{int}}}}}{{L}}{t}}-\frac{{{V}}_{{{\rm{diode}}}}+{{V}}_{{{\rm{out}}}}}{{R}_{{{\rm{int}}}}}$$

Figure 3B presents the power simulation results for the diode, *R*_int_, and *C*_out_ in the LC circuit when the *V*_out_ is 0.5 V. The corresponding current and voltage are shown in the Fig. S16C, D. Furthermore, according to the formular (8)-(10), the pulse width of the current in the circuit narrows (Fig. 3C) with the increasing *V*_out_, signifying a gradual reduction in the energy loss of the diode and *R*_int_ (Fig. 3D and Note S13), the circuit efficiency of the LC circuit also gradually improves accordingly (Fig. S16K). This trend is consistent with the experimental results shown in Fig. 2M.

8 $${{T}}=-\frac{{L}}{{R}_{{{\mathrm{int}}}}}{{{\mathrm{ln}}}}\left(\frac{{{V}}_{{\mbox{out}}}+{{V}}_{{\mbox{diode}}}}{{{R}}_{{{\mathrm{int}}}}{{I}}_{\max }+{{V}}_{{\mbox{out}}}+{{\mbox{V}}}_{{{\rm{diode}}}}}\right)$$

9 $${{E}}_{{\mbox{diode}}}={\int }_{\!\!\!\!0}^{{T}}{P}_{{{{\rm{diode}}}}}{{{\rm{d}}}}t={{V}}_{{\mbox{diode}}}{\int }_{\!\!\!\!0}^{{T}}{I}\left({t}\right){{{\rm{d}}}}t$$

10 $${{E}}_{{\mbox{R}}}={\int }_{\!\!\!\!0}^{{T}}{{P}}_{{\mbox{R}}}{{{\rm{d}}}}t={{R}}{\int }_{\!\!\!\!0}^{{T}}{I}^2\left({t}\right){{{\rm{d}}}}t$$

In addition, for SCC circuit and transformer circuit, the energy loss caused by the diode and *R*_int_ is also the major reason for their low circuit efficiency at lower output voltages. This has been theoretically demonstrated and experimentally verified in Note S14 and Figure S18-21. Although Schottky diodes with smaller *V*_diode_ can reduce energy loss to a certain extent^23^, this problem remains difficult to solve effectively.

### Regulation of input voltage for secondary buck circuit

Elevating the output voltage of the primary circuit serves as an efficient means to minimize energy losses and increase circuit efficiency. However, this approach may not align with the low-voltage requirements of applications. To guarantee that the output voltage conforms to the application’s demands, a secondary circuit is connected to the output terminals of the primary circuit (Fig. 3E). The primary buck circuit is responsible for reducing the higher input voltage to an intermediate voltage (about 16 V), minimizing energy loss and enhancing its circuit efficiency. The secondary circuit further reduces the intermediate voltage to a target voltage. It should be noted that the intermediate voltage needs to be regulated by the secondary circuit. Taking an input voltage of 1,500 V and an output voltage of 5 V as an example, the traditional step-down path is 1,500 V to 5 V, whereas in this paper, the step-down path is 1,500 V to 16 V then to 5 V.

We select a commercial integrated circuit chip as the core component of the secondary buck circuit (the selection for commercial integrated circuit chips is shown Note S15 and Fig. S22). It efficiently converts a 16 V input voltage into an output voltage ranging from 1.8 V to 5 V. Notably, when the output voltage is 5.0 V or 3.45 V, the LTC3588-2 alone serves as the secondary circuit; when the output voltage is 1.8 V or 2.5 V, the secondary circuit is formed by cascading an LTC3588-1 after an LTC3588-2 to achieve the target voltage (Note S15). Test results for the *V*_P_ and *V*_Q_ during the operation of the secondary circuit are shown in the Fig. 3F, with the *C*_out_ of 1 mF. As the *V*_P_ gradually rises to approximately 16 V, the secondary circuit activates and stably outputs voltage approximately 5 V (the *V*_Q_ can be adjusted as needed, Fig. S23). When the TENG stops working, the *V*_P_ gradually decreases, but its output voltage remains at 5 V until *V*_P_ drops to approximately 14 V. The external load impedance is 2.1 kΩ, and the output power reaches 12.21 mW. According to Fig. 2M, the input power of secondary circuit (which is also the output power of primary circuit at 16 V) is 13.0 mW, so the efficiency of the secondary circuit is as high as 93.9%, and the overall efficiency of circuit (including both the primary and secondary circuits) reaches 85.4%. Compared to the output of the primary circuit under the load voltage of approximately 5 V (measured as 5.13 V and 5.06 V, respectively, Fig. S24), the introduction of the secondary circuit results in an increase in output current and output power to 117.6% (2.05 mA to 2.41 mA) and 116.1% (10.52 mW to 12.21 mW) of their original values, respectively (Fig. 3G). Furthermore, as the *V*_Q_ decreases, there is a noticeable increase in current, power, and overall circuit efficiency. When the *V*_Q_ is adjusted to 3.45 V (measured as 3.66 V and 3.69 V, respectively, Fig. 3H, Figs. S23 and S24), the output current and output power are 132.9% (2.52 mA to 3.35 mA) and 133.9% (9.23 mW to 12.36 mW) of their original values, respectively. When the *V*_Q_ is adjusted to 1.8 V (measured as 1.82 V and 1.84 V, respectively, Fig. 3I, Figs S23 and S24), these figures rise to 175.9% (3.32 mA to 5.84 mA) and 178.2% (6.05 mW to 10.78 mW) of their original values. Additionally, the overall circuit efficiencies improved from 73.6%, 64.5%, and 42.3% to 85.4%, 86.4%, and 75.3%, respectively (detailed data comparison is shown in the Fig. S24). Repeated measurements confirmed that these enhancements were stable (Table S2). Furthermore, the long-term stability and thermal behavior of the circuit are shown in Fig. S25.

Although numerous works have utilized the commercial integrated circuit as the power management module for TENGs, the output of TENGs is severely limited because the optimal load voltage for TENGs is much higher than the operating voltage of these chips^22^. Furthermore, our research has revealed that when the input voltage of the secondary circuit is regulated to 4 V (measured as 3.98 V), the output power and overall efficiency of the circuit are only 8.24 mW and 57.6% (Fig. 3I and Fig. S24). The reason is that the efficiency of the primary buck circuit is below 66.5%, when *V*_P_ is set to 4 V. This result further highlights the critical role of *V*_P_ regulation in the multi-stage buck strategy, demonstrating that precise adjustment of this key node is essential for achieving optimal performance.

Figure 3J and Table S3-4 shows the efficiency of PMCs in the low-voltage range in different works^11,12,17,19,23,24^. Here, we normalized the data against using the best efficiency reported in each cited work (the discussions on different calculation methods are shown in the Note16 and Table S3-4). The core objective of this normalization method is to focus on the one of core issues in circuits of “circuit efficiency drop with decreasing voltage”. This effectively avoids the inherent differences in absolute efficiency values. Comparison results robustly demonstrate that our strategy can maintain high performance across a wide voltage range, especially at low voltages, with a significantly smaller degree of performance decay. More importantly, after replacing the LC circuit with a transformer circuit and applying the multi-stage buck strategy, the output performance also increased significantly (Note 14 and Fig. S21). This further demonstrates the broad applicability of the proposed strategy across different primary circuit topologies.

### The multi-stage buck strategy for rotary mode TENGs

**Fig. 4:**
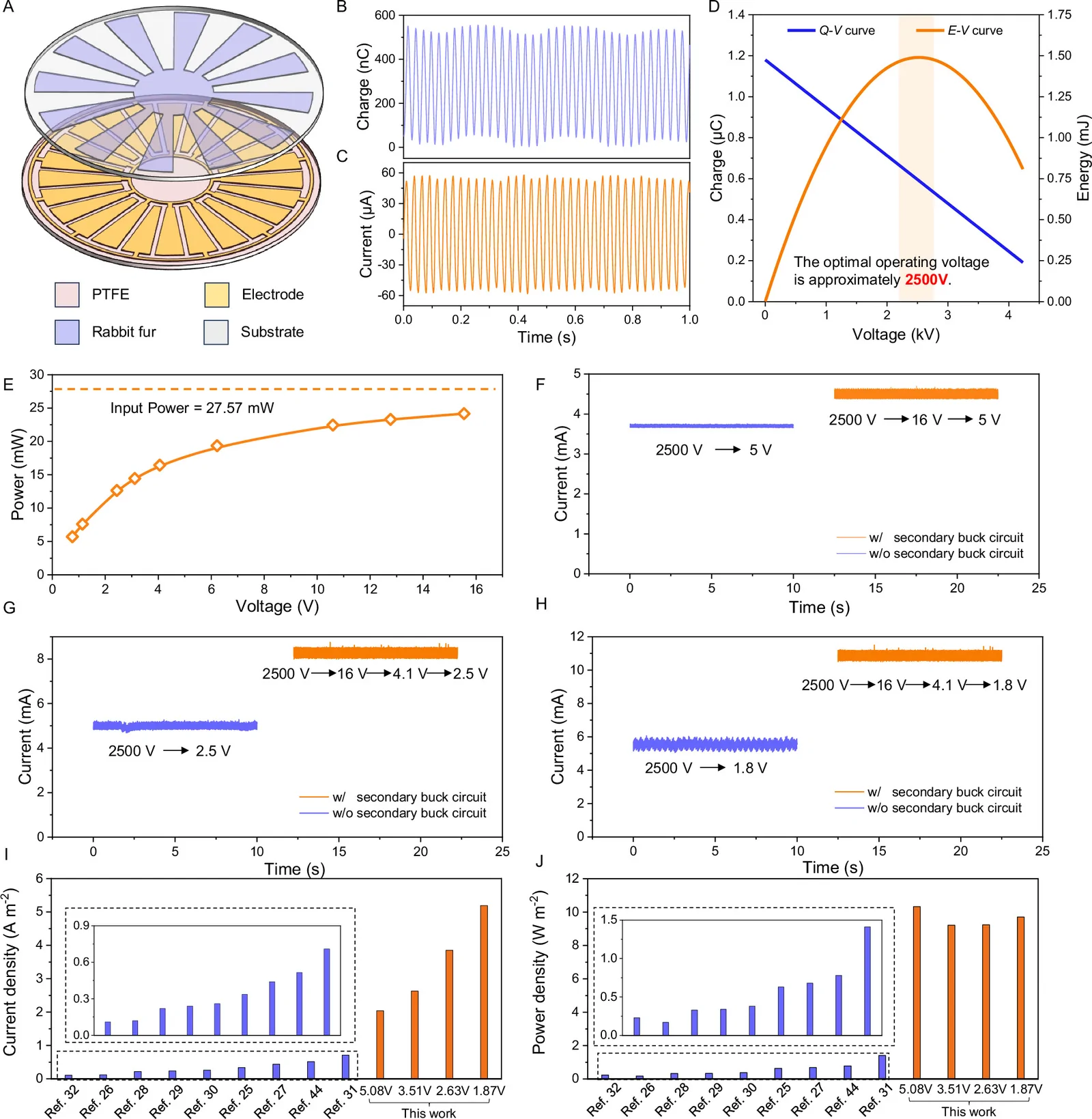
The multi-stage buck strategy for rotary mode TENG. **A** The schematic diagram of the structure for the rotary mode TENG. **B** The output charge and **C** the output current of the rotary mode TENG. The output charge density is about 116 μC m^-2^. **D** The *Q-V* curve and *E-V* curve of the rotary mode TENG. The optimal operating voltage of the primary circuit is approximately 2500 V. **E** The output power of TENG with the primary circuit. **F**–**H** The comparison of output currents when the output voltage *V*_Q_ is set to 5 V, 2.5 V and 1.8 V (without/with the secondary circuit, measured as 5.18/5.20 V, 2.49/2.61 V, and 1.82/1.85 V). **I** The comparison of current density in different works, **J** The comparison of power density in different works. The detail parameters are shown in Table S5. Source data are provided as a Source Data file.

To validate the universality of our strategy, we applied it to the rotary mode TENG, which is commonly employed for harvesting wind energy. Here, PTFE and fur were selected as the triboelectric layers to enhance the stability of the device (Fig. 4A, Fig. S26, and Note S4)^42,43^. At a rotation frequency of 3 Hz, the output charge and current are shown in Figs. 4B and 4C, respectively. As shown in Fig. S27, the rotary mode FS-TENG maintains an almost unchanged output charge after 30,000 operational cycles and exhibits excellent stability. Before designing the primary buck circuit, we used the *Q-V* curve of the device to determine the optimal switching operating voltage range (Fig. S28D). The optimal range was found to be around 2500 V, as shown in Fig. 4D and Fig. S28E. Notably, the optimal voltage results measured at different frequencies are basically consistent, which indicates the important significance for real-world adaptability of this strategy (Fig. S28G–I). Based on the *C*_in_, operating frequency, and operating voltage, we calculated the input power of the primary buck circuit to be 27.57 mW (Fig. S28F). Figure 4E demonstrates that the efficiency of the primary buck circuit remains above 80% at an output voltage of approximately 16 V, but it decreases at voltages below 5 V (the detail data is shown in Figure S28A–C). However, upon introducing the secondary buck circuit, the outperformance has been increased. When the output voltages of the secondary circuit were adjusted to 5 V, 2.5 V, and 1.8 V (without/with the secondary circuit, measured as 5.18/5.20 V, 2.49/2.61 V and 1.82/1.85 V, Fig. S29C), the output current increased from 3.70 mA, 4.99 mA, and 5.52 mA to 4.48 mA, 8.29 mA, and 10.89 mA, respectively (Fig. 4F–H). Similarly, the output powers rose from 19.15 mW, 12.45 mW, and 10.05 mW to 23.28 mW, 21.66 mW, and 20.15 mW, respectively. Consequently, the overall circuit efficiencies improved from 69.5%, 45.1%, and 36.5% to 84.4%, 78.6%, and 73.1%, respectively (detailed data comparison is shown in the Fig. S29). Furthermore, by selecting PTFE and PA as the triboelectric layers due to their high charge transfer efficiency to enhance the device’s performance (Fig. S30-32 and Note S17), we achieved a current density of 5.19 A m⁻² at an output voltage of 1.87 V (Fig. 4I) and a power density of 10.35 W/m^2^ at an output voltage of 5.08 V (Fig. 4J and Table S5). Despite significant performance optimization in previous reported TENG, the current densities and power density remained below 0.71 A/m^2^ and 1.41 W/m^2^(Table S5)^25–32,44^, due to a failure to recognize and address the two PMC issues highlighted in this work.

### The multi-stage buck strategy for self-power systems

**Fig. 5:**
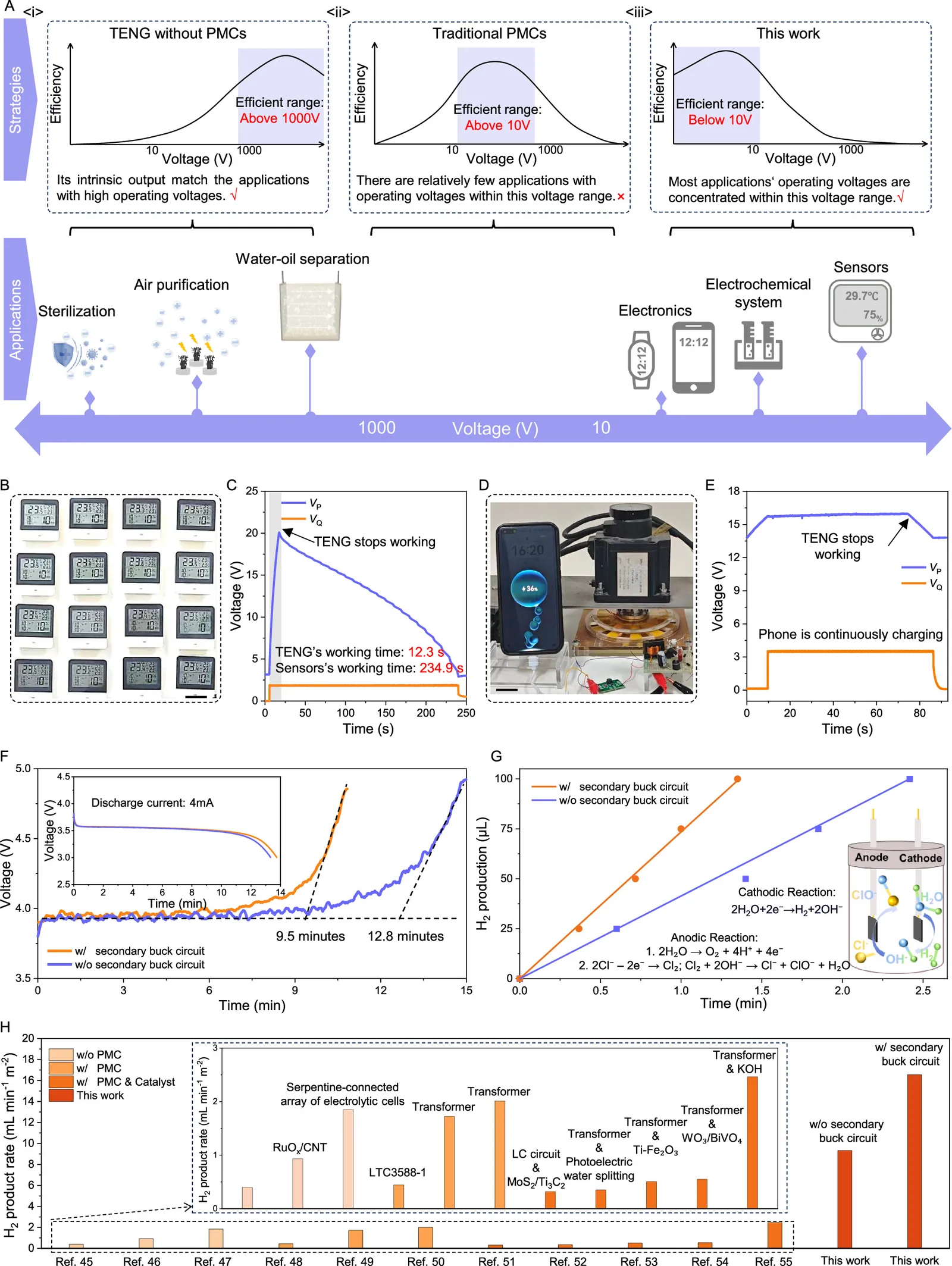
The multi-stage buck strategy for the self-power systems. **A** The relationship between the required voltage of TENG in different applications and the optimal operating voltage of TENG with different power management strategies. **B**, **C** TENG employs a multi-stage buck circuit to drive 16 temperature and humidity sensors. *C*_out_ is 1 mF. The operating frequency is 3 Hz. Scale bar: 5 cm. **D**, **E** TENG charging a mobile phone. Scale bar: 3 cm. **F** The voltage curve of the LIB. The equivalent currents for charging LIB are 5.02 mA and 3.52 mA (Fig. S35), respectively. The inset shows the voltage curve of the LIB when the discharge current is 4 mA. **G** The production rate of the self-powered hydrogen production system. The detailed discussion is shown in Note S20. **H** Comparison of the production rate of the self-powered hydrogen production system with different works. The detail parameters are shown in Table S9. Source data are provided as a Source Data file.

TENGs have inherent output characteristics of high output voltage and low output current, and its output efficiency is usually high when the load voltage reaches above 1,000 V. Therefore, it can be directly used as a high-voltage power source for applications such as sterilization, air purification or water-oil separation without PMCs (Fig. 5A<i > ). Moreover, the operating voltage requirements of most sensors, energy systems, and electrochemical systems are often below 5 V. However, due to the constraints of internal resistance and voltage drop, conventional PMCs can only operate efficiently when the load voltage exceeds 5 V, which obviously mismatches the voltage requirements of most low-voltage applications (Fig. 5A<ii > ). The multi-stage buck strategy proposed in this paper can achieve higher efficiency in the low-voltage range below 5 V. As a power source for these low-voltage applications, it could demonstrate superior performance (Fig. 5A<iii>).

The device shown in Fig. 4A is utilized for application demonstrations. Through the primary circuit, the TENG can charge the *C*_out_ (1 mF) to 20 V in just 12.3 seconds (Fig. 5B, C). Subsequently, with the secondary circuit, even when the TENG stops working, it can drive 16 temperature and humidity sensors (the operating voltage of the temperature and humidity sensor is approximately 1.8 V.) to work continuously and stably for 234.9 seconds. This demonstrates that the secondary circuit provides energy storage and regulation functions, specifically utilizing high-frequency switching to achieve a low voltage ripple (Fig. S33A, B and Note S18), thereby enabling TENGs to deliver a stable and continuous power supply to electronic devices despite fluctuating energy input^25^. In contrast, without the secondary circuit, these sensors can only maintain stable operation for about 55.7 seconds (Fig. S33C, D and Note S19). Furthermore, due to the high efficiency of the multi-stage buck strategy for energy conversion and management, the TENG can also stably powers a pH meter (Fig. S34) and continuously charges a mobile phone (Fig. 5D, E), respectively. Furthermore, it is essential to fabricate efficient energy transfer between TENGs and Lithium-Ion Batteries (LIBs) for promoting the development in combined energy systems. As shown in Fig. 5F, by introducing the secondary buck circuit, the charging time for the LIB is reduced from 12.8 minutes to 9.5 minutes, leading to a 35% improvement in energy storage rate. The corresponding charging current curve is shown in Fig. S35. The internal inset of Fig. 5F shows the voltage curve during constant-current discharge at 4 mA. These results indicate that the multi-stage buck strategy provides a more reliable path for promoting efficient energy transport from the TENG to the LIBs and for constructing an efficient TENG energy system.

More importantly, this strategy proposed in this work introduces a new paradigm for constructing efficient self-powered electrochemical systems driven by TENGs. As shown in Fig. S36, after the steel sheet subjected to cathodic protection was immersed in a 1 mol/L NaCl solution for eight hours, no obvious rust spots were observed on its surface compared to the control group. In addition, hydrogen, as a clean, efficient, and renewable energy carrier, plays a pivotal role in establishing a diversified energy supply system, accelerating the transformation of energy infrastructure and advancing sustainable development. Therefore, a demonstration was conducted using hydrogen production via water electrolysis (Fig. 5G). Specifically, the production rate has increased from 41.38 μL/min to 72.33 μL/min by introducing of the secondary circuit, representing a 75% improvement in overall rate (Table S6). The detailed electrochemical performance, including the specific reaction voltage and current, is comprehensively presented and analyzed in Figs. S37–39, Table S6–8 and Note S20. Notably, neither impedance matching between the hydrogen evolution reaction and TENG via a serpentine-connected array of electrolytic cells, nor simultaneous optimization of the power management circuit and catalyst, has achieved a maximum hydrogen production rate of about 2.5mL/(min·m²) in previous works. Here, the multi-stage buck circuit addresses the issues of reduced circuit efficiency at low voltages and the mismatch between TENGs and PMCs, boosting hydrogen production by nearly an order of magnitude. By applying the *Q-V* curve, we have successfully achieved precise matching between the TENG and PMC, resulting in a production rate of up to 9.3mL/(min·m^2^) even without the secondary buck circuit. Furthermore, the secondary circuit further enhances circuit efficiency, with the production rate reaching 16.5 mL/(min·m^2^) (Fig. 5H and Table S9)^45–55^.

## Discussions

The proposed multi-stage buck strategy establishes a novel power management paradigm for TENGs by accurately regulating the nodal voltage through *Q-V* curves and secondary buck circuit, effectively addressing the critical challenges encountered in energy conversion processes. This strategy reveals the inherent relationship between TENG output characteristics and circuit design parameters through the *Q-V* characteristic curve, guiding circuit optimization to maximize output power. Validation has been achieved in experiments involving rolling-mode, rotary-mode, and sliding-mode TENGs (Fig. S40 and Note S21). Furthermore, simulation results and theoretical analysis indicate that the bottlenecks in conventional circuit strategies stem from internal impedances such as diode voltage drops and internal resistance. By restructuring the single-stage step-down path into a multi-stage path via the secondary buck circuit, efficiency improves significantly, reaching approximately 80% in low-voltage ranges. The strategy demonstrates broad applicability, having been successfully implemented in LC and transformer-coupled circuits (Fig. S21 and Note S14). When applied to the rotary-mode TENG, the system achieves a current density of 5.19 A/m^2^ and power density of 9.70 W/m^2^ at 1.87 V, which represents a nearly order of magnitude improvement over previously reported values of 0.71 A/m^2^and 1.41 W/m^2^. Moreover, both the energy system and the electrochemical system constructed based on multi-stage buck strategy shown substantially enhanced efficiency. Integrated self-powered hydrogen systems exhibit 16.5 mL/(min·m^2^) production rate, also representing a nearly order of magnitude increase compared to prior best of 2.5 mL/(min·m^2^). Comparison presented in Table S10 and Note S22 highlights that the energy cycle collapse theory adopted in this work differs fundamentally from the ideal CMEO theory commonly used in previous works, being particularly suitable for high-performance TENGs where air breakdown is difficult to avoid. The systematic nodal voltage regulation method within the proposed multi-stage buck strategy transcends conventional component-level circuit optimization, establishing a new paradigm for TENG power management design.

Despite the significant progress achieved with the proposed multi-stage buck strategy in this work, TENG power management will likely continue to face several key challenges in future practical applications. First, compatibility of the secondary circuit: the maximum adjustable voltage of the secondary circuit in this work is limited to 16 V. However, in situations where diode voltage drops and internal resistance have a more significant impact, a higher intermediate voltage may be needed to ensure the efficiency of the primary circuit. Second, a three-stage buck circuit: for high-performance TENGs with output voltages reaching tens of thousands of volts, an additional pre-regulation circuit is necessary before the proposed multi-stage buck circuit. This pre-regulation circuit is crucial to adjust the ultra-high voltage to levels that conventional electronic components can handle (Note S23). Third, narrow-hysteresis voltage switches: this switch can extract energy from the TENG in a near‑constant‑voltage manner. When co‑designed with the *Q‑V* curve presented in this work, it can further increase the energy output. Moreover, these switches not only enhance the overall circuit performance but also provide a feasible approach for the miniaturization of high‑performance PMC of TENG (Fig. S41 and Note S24). Finally, challenges for industrial transformation: for successful industrial adoption, the scalability of the circuit fabrication process and the long-term reliability of the integrated system under different environmental conditions are critical challenges that future work must address. In summary, this novel paradigm shift of TENG power management proposed in this work not only overcomes the two fundamental challenges but also enables the practical implementation of high-performance self-charging power systems and self-powered electrochemical systems. This progress accelerates the transition of TENG technology from laboratory prototypes toward industrial-scale applications.

## Methods

### Fabrication of the rolling mode FS-TENG

The device measures 22 cm in height, 20.5 cm in length, and 7 cm in width. It consists of 36 layers, each containing 19 units, and the width of each unit is 1 cm. The air gap between electrodes is set to 5 mm to avoid air breakdown inside the device. The electrodes, which are made of copper, are prepared using printed circuit board (PCB) technology on an epoxy resin substrate that is 1 millimeter thick. For the triboelectric layer, 4-millimeter-diameter PTFE spheres are used, with each unit employing 14 such spheres.

### Fabrication of the rotary mode FS-TENG

Stator: The Cu electrodes were sputtered onto an acrylic substrate with a thickness of 3 mm, resulting in an electrode thickness of approximately 100 nm. The outer diameter of the electrode is 70/130 mm, while the inner diameter is 30/60 mm. The electrodes are evenly divided into 8/12 pairs, with a 1.5/2 mm gap between each electrode. Detailed parameters are shown in Figs. S26 and S30. A 50 µm thick polyimide (PI) layer was then attached to the electrode surface to enhance the device’s insulation performance. Subsequently, a PA/PTFE film was adhered above the electrodes.

Rotor: PTFE/fur was used as the triboelectric layer for the rotor, with dimensions corresponding to the stator electrodes.

### The electric component model numbers

The key components used in the primary circuit include the input capacitor [CBB81-2000V-221J], the GDTs [SXH81-152X], the inductor [SMDRI127-102MT], the freewheeling diode [FR1006GP-TP], and the output capacitor [UHE1E102MHD6TO]. The detailed parameters are shown in Fig. S12. Adjusting threshold voltage and capacitance through series or parallel connection of electronic components.

The main components employed in the secondary circuit are capacitor C1 [GRM319R71A105KA01D], capacitor C2 [GRM31CR71A475KA01K], capacitor C3 [GRM31CC80J476KE18K], inductor L [SLF7045T-101MR50-PF], and the integrated circuit [LTC3588EMSE-2/LTC3588EMSE-1]. The details parameters are shown in Fig. S22.

### Measurement and characterization

The output current and charge of TENG were measured using an electrostatic electrometer (Keithley 6514). The output current and voltage of both the LC circuit and the multi-stage circuit were measured by varying the resistance at the output, and the output power was calculated by current, voltage, and resistance. For the multi-stage circuit tests, the load resistance was carefully adjusted to ensure stable input and output voltages for the secondary circuit (Fig. 3F and Fig. S23), guaranteeing the accuracy of the measurements. The Cu electrode was sputtered on the acrylic substrate using a Denton magnetron sputtering coating instrument (Discovery635). The relative sliding motions of the devices were achieved through a linear motor (TSMV120-1S, LinMot), with detailed sliding motion parameters provided in Fig. S3. Measurement of the *Q-V* curve was conducted using the circuit depicted in Fig. 2F, with a test capacitance of 22nF. The rotary mode FS-TENG was driven by a commercial programmable stepper motor (86BYG250D). Hydrogen gas (10 mL) was collected and analyzed by GC‑TCD (Agilent 8890B) using argon (99.999%) as the carrier gas. All photographs in this work were captured with a Nikon D750 digital camera. Circuit simulations were conducted using LT-SPICE.

## Supplementary information

Supplementary Information

Transparent Peer Review file

## Source data

Source Data

## Data Availability

Source data are provided with this paper. All data generated in this study are provided in the Supplementary Information and Source Data files. Source data are provided with this paper.
