# Supplementary material for: Multi-Stage buck strategy for power management of triboelectric nanogenerators
Source: Nat Commun. 2026 Sep 29;17:11216. doi: 10.1038/s41467-026-78002-0 (PMC13720038; doi:10.1038/s41467-026-78002-0)
Supplement: Supplementary file 1 [file 41467_2026_78002_MOESM1_ESM.pdf]

Supplementary Information

Multi-Stage Buck Strategy for Power Management of Triboelectric Nanogenerators

Yikui Gao[1,2], Lixia He[1,2], Xiangyu Hu[1,2], Di Liu[3], Linglin Zhou[1,2], Hanlin Sun[4], Xinyuan Li[1,2], Xianggang Dai[1,2], Wenyan Qiao[1,2], Bingzhe Jin[1,2], Zhong Lin Wang[1,2,5,6*], Jie Wang[1,2,5*]

[1]Beijing Key Laboratory of High-Entropy Energy Materials and Devices, Beijing Institute of Nanoenergy and Nanosystems, Chinese Academy of Sciences, Beijing 101400, P. R. China.

[2]School of Nanoscience and Engineering, University of Chinese Academy of Sciences, Beijing 100049, P. R. China.

[3]Department of Mechanical Engineering, The Hong Kong Polytechnic University, Hong Kong 999077, P. R. China

[4]Department of Mechanical Engineering, Tsinghua University, Beijing 100084, P. R. China.

[5]Guangzhou Institute of Blue Energy, Knowledge City, Huangpu District, Guangzhou 510555, P. R. China

[6]Yonsei Frontier Lab, Yonsei University, Seoul 03722, Republic of Korea.

*Corresponding Author:

Z. L. Wang: zhong.wang@mse.gatech.edu; J. Wang: wangjie@binn.cas.cn

Keywords: triboelectric nanogenerator, power management, multi-stage buck strategy, self-powered systems, current density

Note S1 Two Core Issues of TENG's PMC

Undoubtedly, power management circuits (PMCs) are indispensable components for the real-world application of TENGs. This necessity arises from the inherent output characteristics of TENGs, which generate high voltage (often exceeding 1 kV) but relatively low current, are fundamentally mismatched with the low-voltage power requirements (typically below 5 V) of most practical applications. Despite nearly a decade of technological advancement, two core issues remain unresolved.

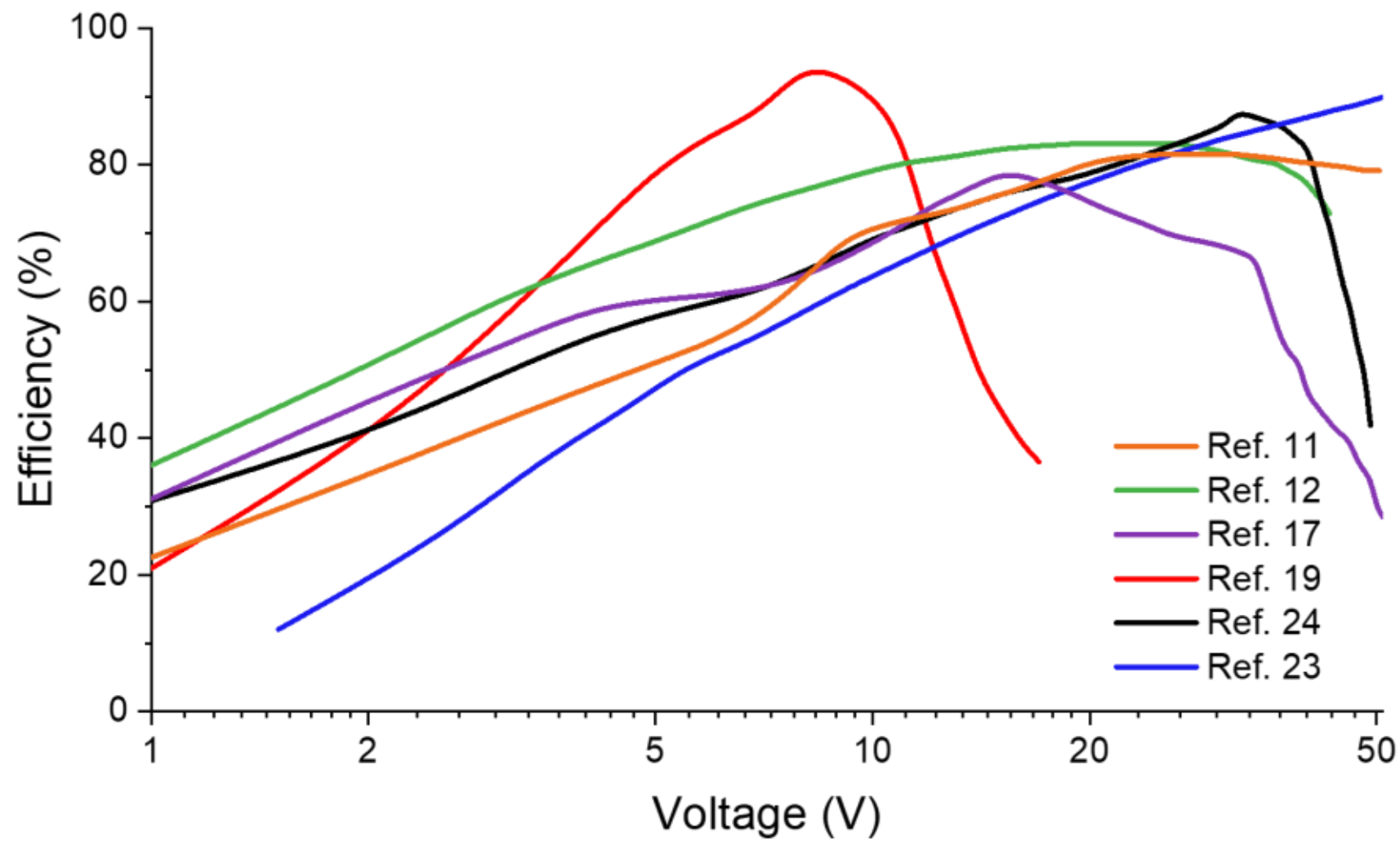

Figure S1 Efficiency of different circuits in different works. Source data are provided as a Source Data file.

First, the issue of low efficiency under low voltage. Although many studies report optimal conversion efficiencies of PMCs as high as over 90%, when actually applied in electronic devices operating below 5V, the practical circuit efficiency is often significantly lower than the reported values as shown in Figure S1. What are the main reasons behind this efficiency gap, and what are the potential approaches to address this issue?

The second issue is the significant drop in output power exhibited by high-performance TENGs when operating under low-voltage after the integration of a PMCs. As shown in Table S1, for the high performance TENGs from recent years (the power density over 10 W/m^2), their actual output power with PMC is often less than one-tenth of the

reported optimal performance. Beyond the reason of reduced circuit efficiency at low voltage, what are the deeper underlying constraints causing this problem?

Table S1 Performance of different high-performance TENGs *.

Ref.	26	27	28	29	30	31	32
Power density without PMCs/Input power density (W/m^2)	7.62	21.6	20.7	12.3	87.26	32.12	23.2
Current density without PMCs (A/m^2)	0.0084	0.0121	0.0067	0.0129	0.032	0.012	0.0061
Power density with PMCs/Output power density (W/m^2)	0.17	0.68	0.33	0.34	0.38	1.41	0.23
Current density with PMCs (A/m^2)	0.12	0.44	0.22	0.24	0.26	0.71	0.11
The ratio of power density with PMCs to power density without PMCs	2.2%	3.1%	1.6%	2.8%	0.4%	4.4%	1.0%
Frequency (Hz)	1	1	1	2	1	2	0.375
The type and operating voltage (kV) of the switch	SCR < 0.8	SCR < 0.8	SCR < 0.8	SCR < 0.8	SCR < 0.8	SCR < 0.8	GDT 0.8
Circuit architectures	LC	LC	LC	LC	LC	LC	LC

*Some works did not report the relevant current density and power density after PMCs; therefore, comparisons were made based on the average charging rate and average power when the capacitor was charged to approximately 3V.

Note S2 The Energy Cycle Collapse and *Q–V* Characteristic Curve

Energy Cycle Collapse: In high-performance TENGs, as the output voltage increases, the strong electric field at the electrode edges can induce air breakdown, leading to decline of the surface charge density (σ) on the triboelectric layer. This phenomenon causes the energy cycle of the TENG to "collapse", preventing it from reaching the ideal curve[1]. This implies that power management strategies based solely on the conventional CMEO (cycle for maximum energy output, which was first proposed by Zi et al) theory and guided only by the *V-Q* curve are not feasible[2]. The commonly observed significant performance degradation of high-performance TENGs when connected to existing PMCs further confirms the theory lack of a suitable design guideline for such devices (Table S1).

On the contrary, if the surface charge density remains stable while the output voltage rises, the energy cycle can achieve the theoretically ideal state. We name this phenomenon is energy cycle stable state. Prior studies have confirmed that the method—replacing the input capacitance (C_{in}) of the PMC with the TENG's inherent capacitance (C_T)—represents the optimal solution[3-5]. Such stable state typically occurs in devices with low surface charge density or where circuit regulation can fully suppress air breakdown at high voltages.

***Q–V* Characteristic Curve and the Optimal Output Energy:** Under ideal conditions (without air breakdown), the TENG output is determined by a fixed charge density σ_0 and the inherent capacitance. However, air breakdown causes σ_0 to decrease dynamically with increasing voltage, producing a series of actual *V–Q* curves that vary with the output voltage (Figure S2). Connecting the actual operating points at different voltages yields the "actual energy-output curve". Swapping the horizontal and vertical coordinates of this curve gives the *Q–V* output characteristic curve, which directly reflects the amount of charge that the TENG can actually output at a given output voltage.

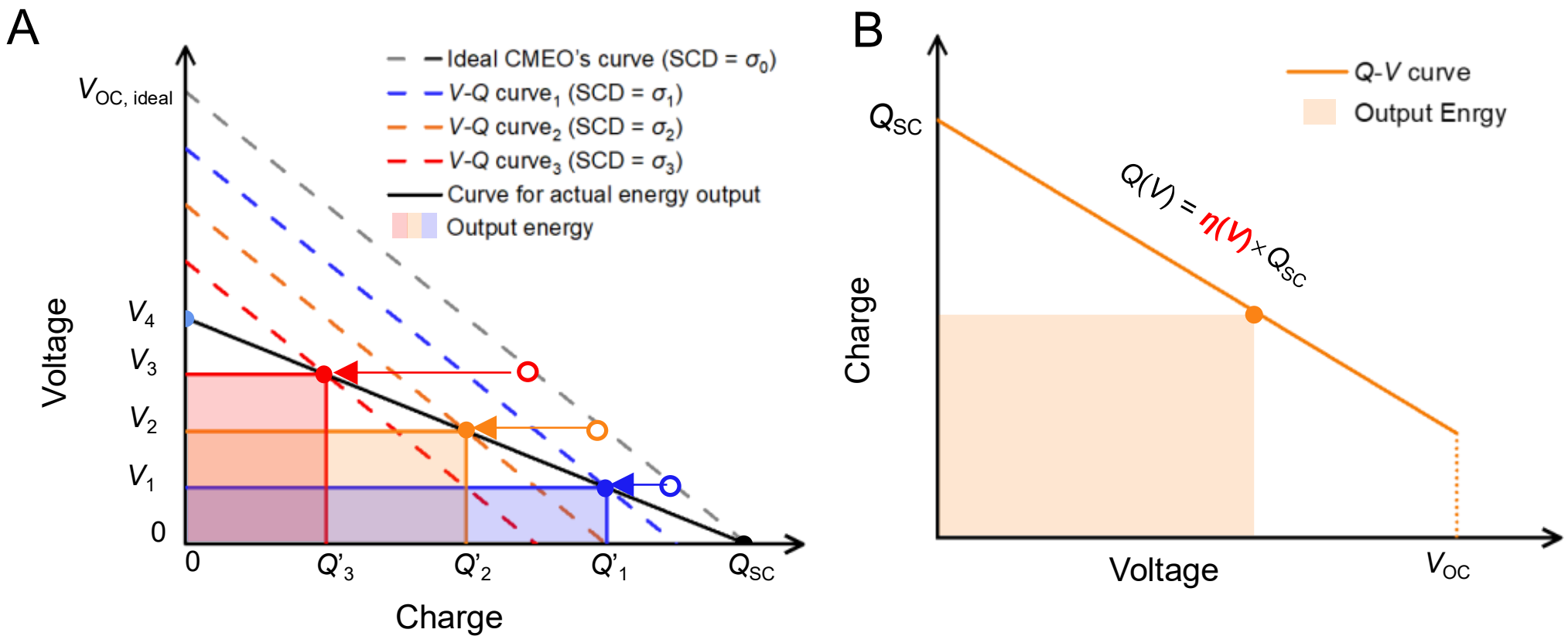

Figure S2. The collapsed state of the energy cycle and the *Q-V* output characteristics curve of TENG. (a) The collapsed state of the energy cycle. The $V_{oc, ideal}$ is the ideal open-circuit voltage. It can be calculated by the short-circuit output charge (Q_{sc}) divided by inherent capacitor of TENG. As the output voltage gradually increases from 0 to V_3, the surface charge density gradually decreases from σ_0 to σ_3. The energy cycle gradually collapses, and the slope of the curve (dashed line) remains unchanged. The corresponding output charge and output energy decrease. (b) The *Q-V* output characteristics curve of TENG. Coulombic efficiency ($\eta(V)$) defined as the ratio of the output charge to the short-circuit charge at a given output voltage. V_{oc} is the actual open-circuit voltage. When the output voltage of the TENG reaches V_{oc}, the voltage stops rising due to breakdown occurs inside the device or the output charge drops to zero. Source data are provided as a Source Data file.

In our previous work, we defined the Coulombic efficiency as the ratio of the actual output charge to the short-circuit charge[1]. Theoretical analysis shows that the energy output of the TENG reaches maximum when the Coulombic efficiency is 50%.

Therefore, in this work we propose that the operating voltage corresponding to this efficiency on the *Q–V* curve represents the theoretical optimal voltage for achieving the best energy match between the TENG and PMC. The key criterion for determining the applicability of the *Q–V* curve is not the motion type of the TENG (e.g., rolling, rotary, contact-separation, or sliding), but whether its energy cycle exhibits collapse characteristics—specifically, whether surface charge density declines as voltage increases.

Note S3 Technical Advance of This Work

Although the circuit architecture of some works is similar to that proposed in this work[6], the power management strategy in this work is fundamentally based on the TENG energy cycle collapse theory. It aims to address the two core issues mentioned above through precise regulation of key voltage nodes, representing a fundamental difference in design logic from these works. This distinction is clearly demonstrated in the following aspects.

One of our technical advances is accurately understanding the root cause of the efficiency drop in conventional circuits under low output voltage and proposing a practical and referable solution. Our analysis reveals that the primary reason for efficiency drop under low output voltage is the energy loss caused by diodes and internal resistance. As shown in Figure 3D, this energy loss increases significantly as the output voltage decreases. To address this, we designed a multi-stage buck circuit with an intermediate voltage of 16 V. This intermediate voltage was chosen based on a clear efficiency advantage: the primary circuit achieves an efficiency of 90.9 % at 16 V, thereby substantially reducing energy loss in primary circuit. More importantly, experimental comparison of two step-down paths (1500 V → 4 V → 1.8 V and 1500 V → 16 V → 4.1V → 1.8 V) shows that when the intermediate voltage is set to 4 V, the overall system efficiency is much lower than with a 16 V intermediate voltage, due to the considerably higher energy loss in the primary circuit (Figure 3I). This clearly confirms the importance of regulating the intermediate voltage node. Through this design, we effectively improve the overall system efficiency from the source. In contrast, other works neither recognized the fundamental issue described above nor provided a targeted solution.

Another key technical advance lies in the application of our proposed *Q-V* output characteristic curve based on Coulombic efficiency, which provides a direct and practical method for the optimal match between TENG and PMCs. This curve visually represents the output performance of a TENG across different output voltages, enabling accurate determination of the circuit's optimal operating voltage to maximize power

delivery. The theoretical analysis on guiding PMC design using the *Q-V* curve demonstrate the feasibility and rationality of this strategy (Note S6). As a result, this work achieves a current density of 5.19 A/m^2 and a power density of 9.70 W/m^2 at 1.87 V, which is markedly higher than that of all previous work. The significant improvement is attributed not only to resolving the low-efficiency issue at low output voltage, but also to synergy mechanism between the output characteristics of TENGs and the PMCs. Furthermore, the enhanced performance is convincingly demonstrated in electrochemical applications, where an impressively high hydrogen production rate of 16.5 mL/(min·m^2) was achieved. This outcome also confirms that the efficiency breakthrough in TENG-powered electrochemical systems not only need application-specific optimizations, but also need to match the TENG's electrical output effectively with the subsequent electrochemical application. In contrast, most works lack such an output-oriented guidance method. The inherent issue of energy cycle collapse in the TENG used by their study has been clarified in our prior work. The phenomenon of energy cycle collapse significantly undermines the validity of their optimization mechanism, which relies solely on the ideal *V-Q* plot.

Thus, the similarity in circuit architecture further underscores the deeper understanding embodied in our work regarding TENG performance enhancement and circuit optimization. The essence of our multi-stage buck design lies not merely in its topological configuration, but in the precise setting and dynamic regulation of the key nodal voltages—which is precisely the key to solving the aforementioned two core issues and represents the critical advancement that other works failed to achieve.

Note S4 The material selection for TENGs

Material selection is a core issue in the fabrication of triboelectric nanogenerators (TENGs). Although the triboelectric series is often used as a reference for materials screening, the triboelectric series reported in different studies often exhibit discrepancies, due to the influence of factors such as device structure and environmental parameters. For instance, in the experiments on triboelectric materials series designed by Liu et al. and Zou et al., the determined results differed significantly due to changes in the environment parameters[7,8]. Therefore, recent works usually need to quantify a triboelectric series applicable to their research by considering the device structure and working environment. In addition, they also comprehensively evaluate parameters such as the material's friction coefficient and stability to select the most suitable materials[9,10]. For the rolling-mode FS-TENG adopted in this manuscript (Figure 2A), existing researches indicate that PTFE spheres, as the triboelectric material for this device, demonstrate the best performance in terms of output performance and stability[11-13]. For the rotary mode FS-TENG, PTFE not only exhibits excellent electrical output performance and stability but also can effectively enhance the mechanical energy conversion efficiency, due to its low friction coefficient. These advantages have been verified through many studies[14, 15].

We consider that a multi-dimensional evaluation system should be established for future material research and development in TENGs: the performance of materials should match TENG structure and mechanism; assess stability under operational environments; balance cost/processability with performance for practical viability. Importantly, the strategies proposed in this work maintain applicability for power management circuit of TENG regardless of materials.

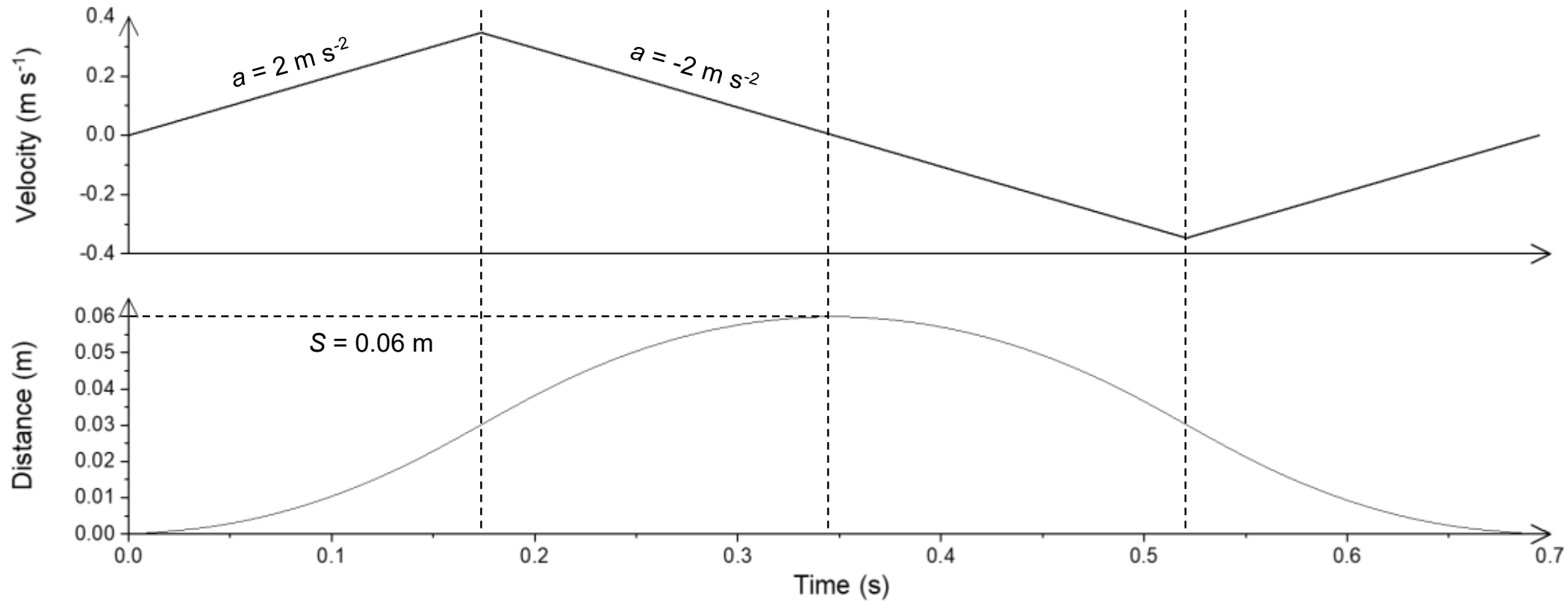

Figure S3. The motion parameters of the rolling mode FS-TENG. Source data are provided as a Source Data file.

Note S5 The Strategy for Addressing Energy Input Fluctuations

All technologies designed for distributed energy supply in the IoT era, aimed at harvesting ambient energy (such as wind, wave, and solar energy), face the challenge of fluctuating or intermittent energy input. Currently, no technology exists that can completely circumvent this fluctuating energy input. For single-source energy harvesting technologies, introducing back-end energy storage systems (such as lithium-ion batteries or supercapacitors) is a common solution to address this challenge. For instance, in solar cell technology applications for IoT, energy storage systems store excess energy during the day or on sunny days and release it at night or on cloudy days, thereby ensuring the stable operation of electronic devices. Additionally, hybrid energy harvesting system strategies are also an effective way to mitigate this shortcoming. As for mechanical energy harvesting technologies (such as piezoelectric generators and triboelectric generators), optimizing structural design to broaden their response frequency and range is another feasible solution[16-18]. A self-learning controller could be considered as a candidate to facilitate optimized performance with rapid tracking of environmental change in the future[19].

The LTC-3588 chip provides energy storage and regulation functions, enabling TENGs to deliver a stable and continuous power supply to electronic devices despite fluctuating energy input[6]. This is also confirmed by our experimental results: in the power supply demonstration experiments for the temperature/humidity sensor and pH meter (Figure 5B, Figure 5C, Figure S34), both devices continued to operate effectively for durations of approximately 235 seconds and 30 seconds, respectively, even after the mechanical input ceased (motor rotation stopped after approximately 12 seconds). More importantly, the *Q-V* curves tested results at different frequencies, indicate the optimal output voltage remained consistent at approximately 2500 V as the increasing frequency (Figure S28D and Figure S28G-I). This indicates that frequency has a negligible effect on the optimal matching voltage when coordinating the TENG's output characteristics with the PMC, which holds important significance for real-world adaptability.

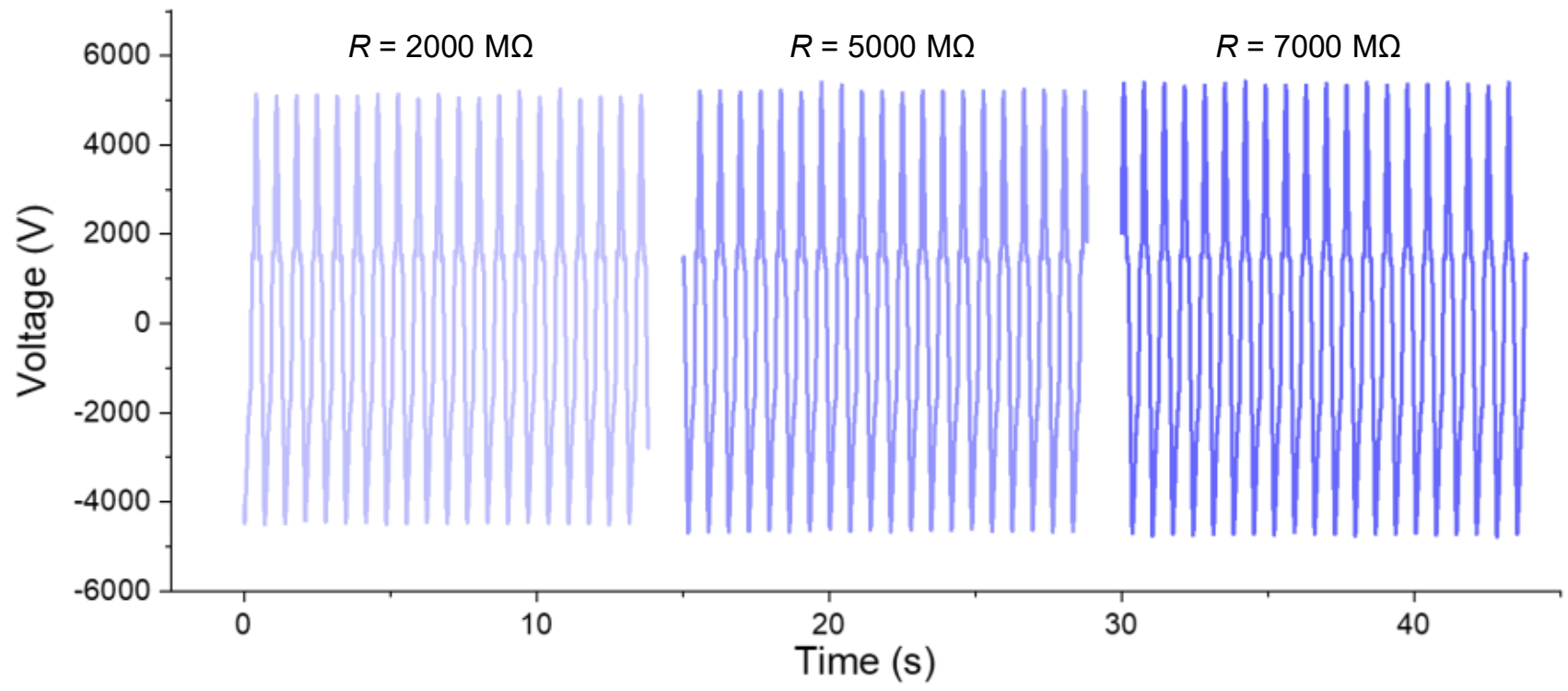

Figure S4. The output voltage of the device rolling mode FS-TENG. Source data are provided as a Source Data file.

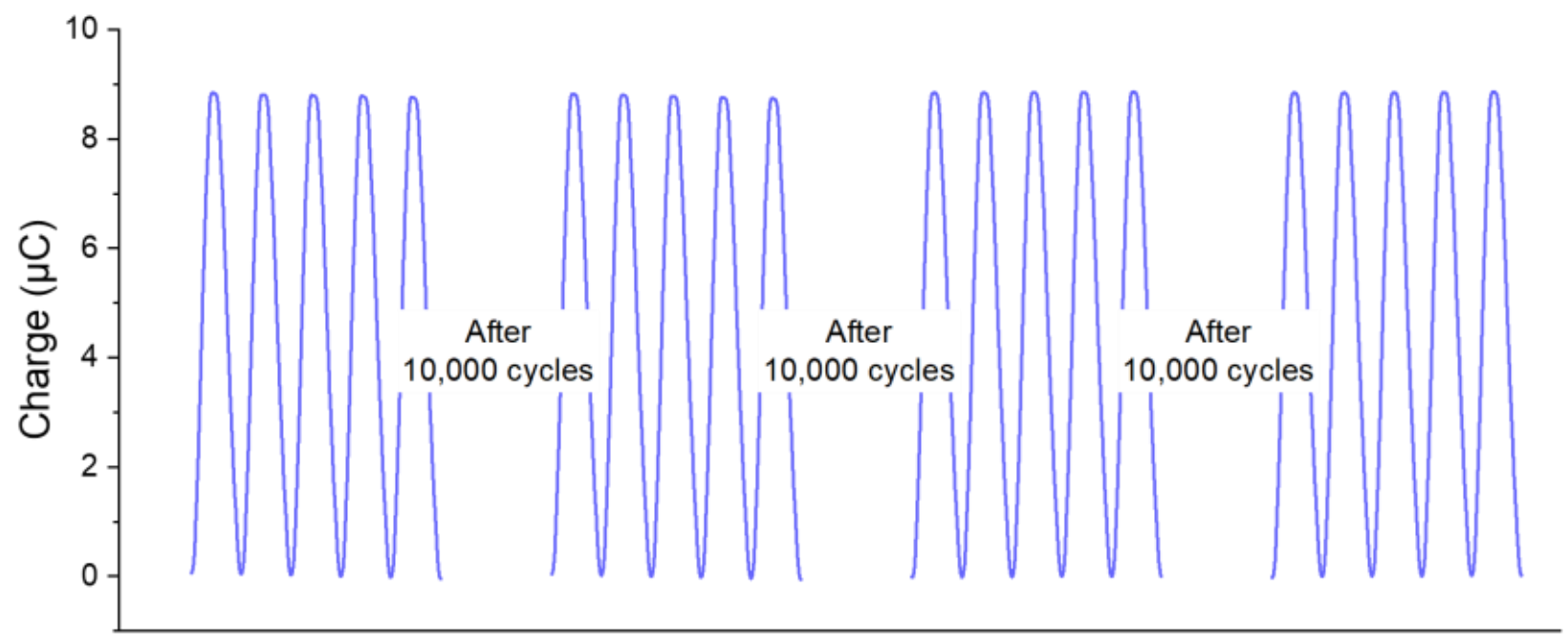

Figure S5. The output charge of rolling mode FS-TENG after 30,000 cycles. Source data are provided as a Source Data file.

Note S6 Theoretical Verification of *Q-V* Curve Guidance for Optimal Operating Voltage of PMC

We start from the energy cycle of the TENG and derive the matching process with a capacitive load (the PMC input capacitance C_{in}).

When the C_{in} is charged to a voltage V_n, the surface charge density of the TENG decays to σ_n. The energy (E) stored in the capacitor is then:

$$E = \frac{1}{2} C_{\text{in}} V_n^2 \tag{1}$$

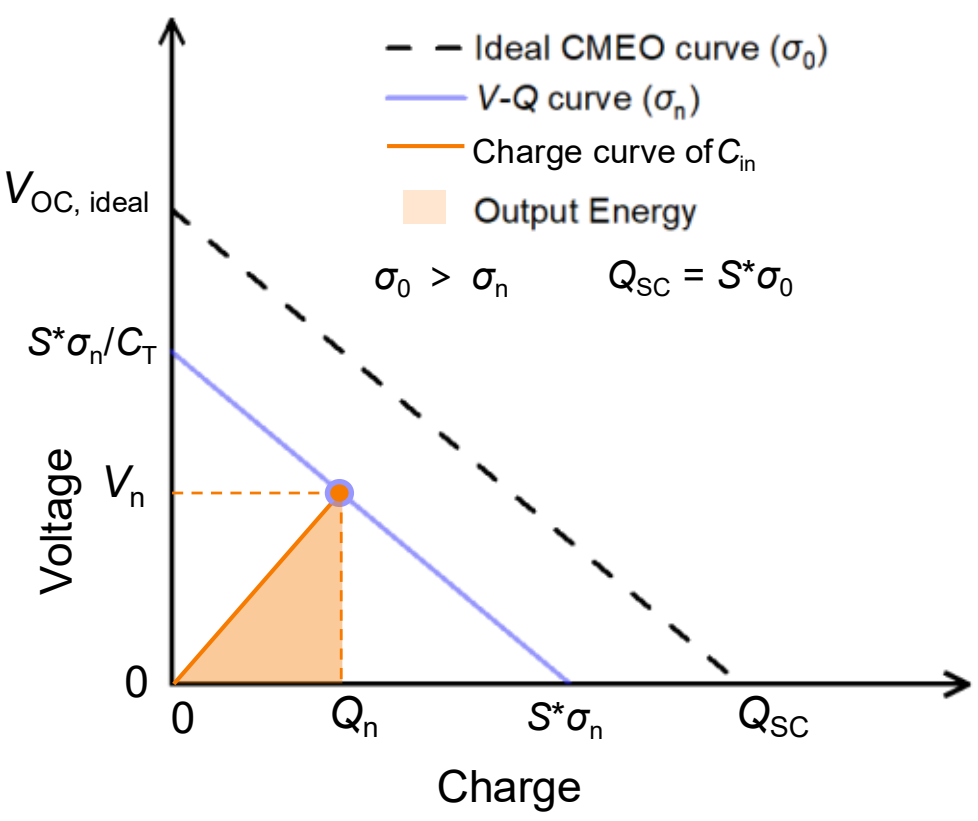

Figure S6. Energy storage process of the capacitor during TENG energy cycle collapse. Assuming the capacitor is ultimately charged to a voltage V_n, its charging curve will intersect the actual energy cycle curve of the TENG corresponding to that voltage V_n. At this point, due to air breakdown, the TENG's surface charge density has decayed to σ_n. The orange shaded area in the figure represents the energy stored in the capacitor. This area is maximized when the TENG's actual output charge Q_n is half of its short-circuit charge Q_{SC}. Source data are provided as a Source Data file.

Intersection point of two curves in Figure S6 simultaneously satisfies the intrinsic *V–Q* equation of the TENG and the capacitor characteristic:

$$V_n = \frac{\sigma_n S}{C_T} - \frac{Q_n}{C_T} \tag{2}$$

$$V_n = \frac{Q_n}{C_{\text{in}}} \tag{3}$$

Where S is the equivalent area of TENG, Q_n is the output charge of TENG.

Combining above three equations yields:

$$E=\frac{1}{2}(\sigma_n S\text{-}C_T V_n)V_n \quad (4)$$

The key point is that the surface charge density σ_n is a function of the voltage V_n. Our earlier work[1] has demonstrated that, within a certain voltage range, the two follow a linear decay relationship:

$$\sigma_n=\sigma_0\text{-k}V_n \;\; \text{(k is a constant)} \quad (5)$$

Substituting this relation into the energy expression and maximizing it, we obtain the optimal operating condition:

$$Q_n=\frac{\sigma_0 S}{2} \quad (6)$$

At this point, the actual output charge Q_n is exactly half of the ideal short-circuit charge Q_{sc}, and the Coulombic efficiency is 50%. This validates the correctness of the conclusions we have drawn above.

It is worth noting that the operating voltage selected in this work corresponds to a Coulombic efficiency on the Q–V curve of approximately 50%, which aligns well with the results discussed in Note S2.

Note S7 The Optimization Direction for Rolling-Mode FS-TENG

Neglecting factors such as electrostatic breakdown and dielectric leakage, its theoretical *Q*-*V* curve is depicted by the red dashed line in the Figure S7B. At a test voltage of 1000 V, the leakage current of the device is approximately 700 nA. Therefore, after accounting for the impact of this leakage current, the actual *Q*-*V* curve of the device is represented by the blue dashed line in the Figure S7B. Furthermore, after completing the *Q*-*V* curve testing, we observed that the short-circuit current output of the device had decreased to 35.4 μA, representing a 26.7% reduction compared to the initial value of 48.3 μA (Figure S7C). This phenomenon indicates that the surface charge density of the device has decreased due to air breakdown. Consequently, after considering the factor of electrostatic breakdown, the actual *Q*-*V* curve of the device is further adjusted to the purple dashed line in the Figure S7B.

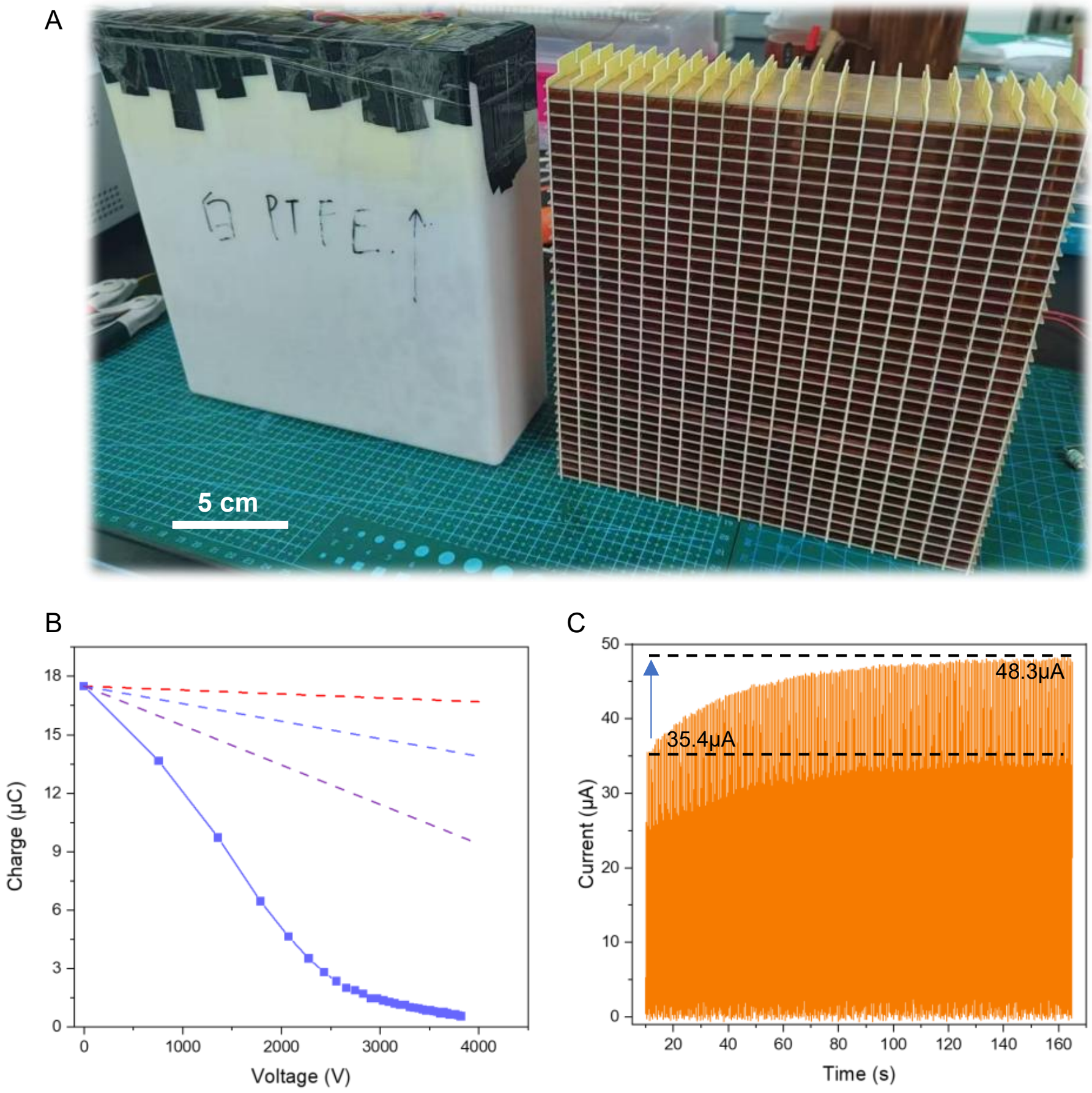

Figure S7. The physical image of the device and its output performance. (A) Physical image of the device (scale bar: 5 cm). (B) *Q*-*V* curve of the device. (C) Output current of the device after open-circuit testing. Source data are provided as a Source Data file.

It is worth noting that as the output voltage changes, the electrostatic force acting on the PTFE sphere will also vary, directly affecting the actual motion state of the sphere. This explains why, even after considering both electrostatic breakdown and material leakage, the actual measured *Q-V* curve (blue solid line) is still lower than the theoretical curve (purple dashed line). Therefore, in future device optimization efforts, addressing the issues of electrostatic breakdown and material leakage will be of utmost importance. Meanwhile, during the performance evaluation of TENGs, the impact of electrostatic forces on the device's operating state should also be more thoroughly considered to ensure the accuracy and comprehensiveness of the evaluation results.

Note S8 The Testing Method Regarding The *Q-V* Characteristics Curve of TENGs.

The testing circuit is shown in Figure S8A, which is composed of a rectifying circuit, a test capacitor (C_{test}), protective resistors (R_p), and an electrostatic electrometer. R_p (1 MΩ) is used to protect the electrometer, which measures the output charge and output current of TENG.

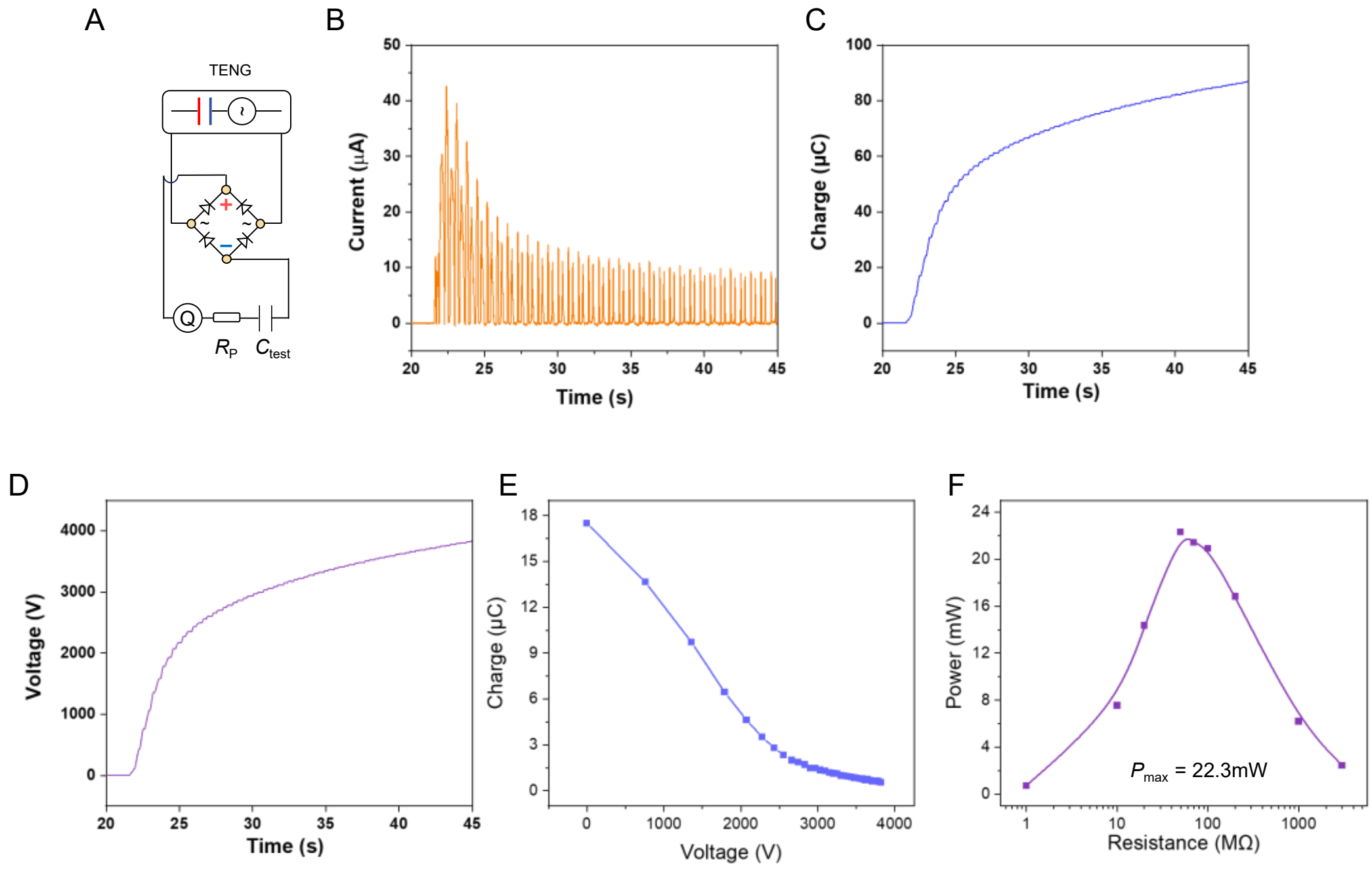

Figure S8. **The testing method regarding the *Q-V* characteristics curve of TENGs.** (A) The schematic diagram of the circuit for testing the *Q-V* curve. (B) The output current. (C) The output charge by integrating the output current. (D) The output voltage. (E) The *Q-V* characteristics curve of TENG. (F) The output power measured by resistance. Source data are provided as a Source Data file.

The output charge can be directly measured or can be determined by integrating the output current (Figure S8B and Figure S8C). The C_{test} is the core component, which is used to store the output charge and increase the output voltage in the next working cycle of TENG. The output voltage of the TENG can be expressed as:

$$V_{\mathrm{TENG}}=V_{\mathrm{C}}+V_{\mathrm{R}} \tag{7}$$

Where V_C is the voltage of capacitor, V_R is the voltage of R_{p1}.

Given that the output current is very small (~10 μA), the V_R is much smaller than V_C. V_R can be neglected, so the following equation can be obtained (Figure S8D).

$$V_{\mathrm{TENG}}\approx V_{\mathrm{C}}=\frac{Q}{C_{\mathrm{test}}} \tag{8}$$

where Q is the charge stored in capacitor. It is noted that if there is leakage current in

the test circuit, the tested charge will be larger than the actual stored charge in the capacitor, and the voltage calculated by equation (8) will be higher than the actual voltage, resulting in inaccurate voltage measurement. Plot the *Q-V* curve (Figure S8E) by the output voltage in the nth cycle as the horizontal axis (x-axis) and the output charge in the nth cycle as the vertical axis (y-axis). The larger the test capacitance, the more data points can be obtained, leading to more accurate experimental results.

Note S9 Insulation Design Considerations for TENGs and PMCs

Air Gap Insulation and Circuit Layout: Air breakdown is a critical consideration in high-voltage circuit design. We characterized the breakdown voltage for various air gaps. Figure S9A show that a 3 mm air gap can withstand up to 4400 V. In the circuit layout, air gap between high-voltage nodes were strictly controlled. As shown in Figure S9B, the high-voltage (red), output (purple), and ground (blue) nodes are clearly identified. Figure S9B shows the minimum distances between these critical nodes. This layout guarantees that internal air breakdown will not occur at the 1.5 kV operating voltage.

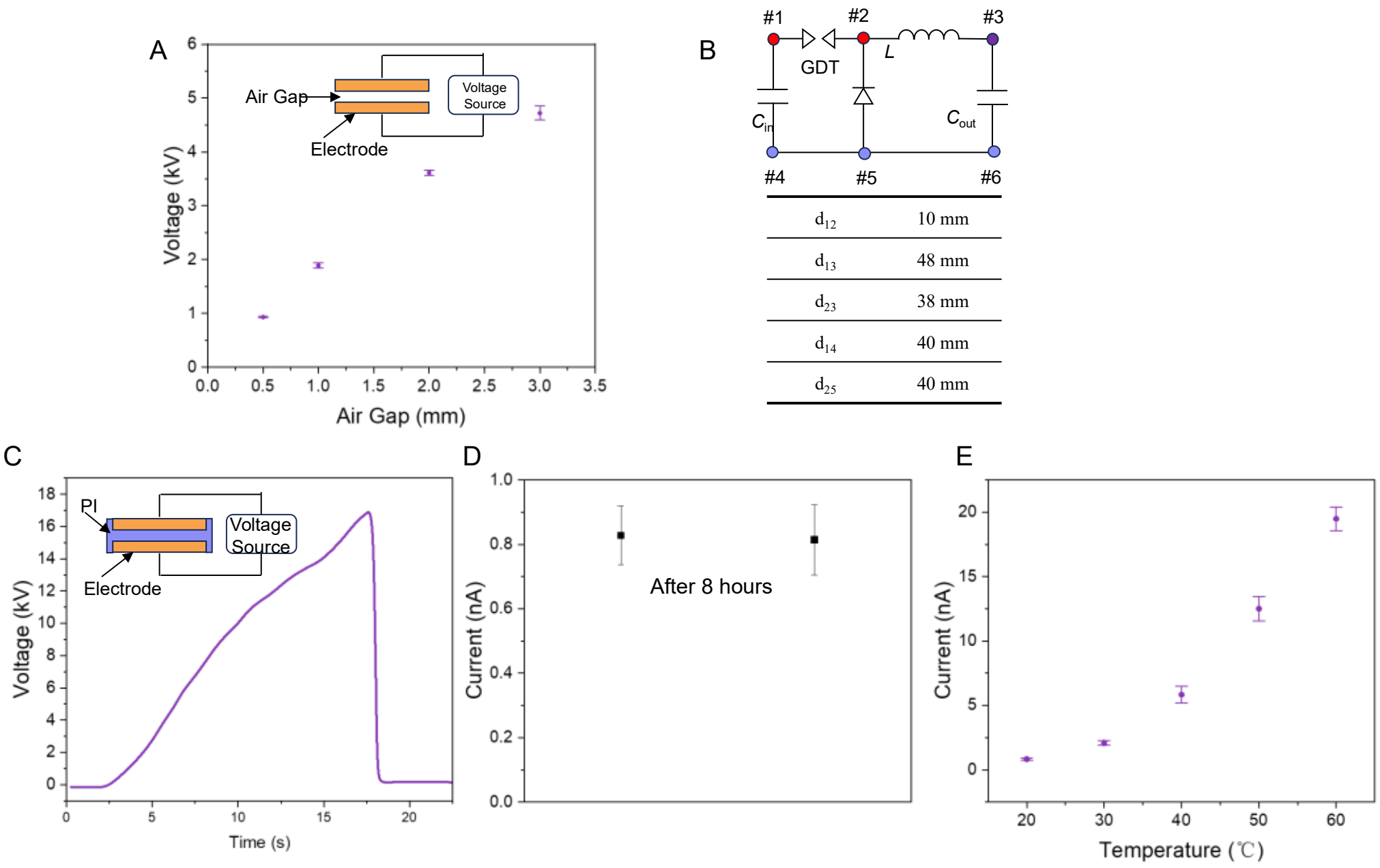

Figure S9. The insulation performance of air and PI. (A) Breakdown voltage under different air gaps (the test circuit is shown in the illustration); (B) Minimum distance between primary circuit pins; (C) Dielectric strength test of PI (the test circuit is shown in the illustration); (D) Leakage current of PI at 1500V (temperature is 20 ℃); (E) Leakage current test of PI under different temperatures (voltage is 1500 V). Error bars represent Standard Deviation (n = 5). Source data are provided as a Source Data file.

Solid Insulation: To eliminate safety hazards and suppress corona discharge at pin tips of electronic components, all high-voltage pins were insulated with polyimide (PI). The employed PI material was characterized: at the 1500 V DC operating voltage, the leakage current is below 1 nA (Figure S9D); the dielectric strength of the 50 µm-thick film is approximately 340 V/µm, corresponding to a breakdown voltage of ~17 kV,

which is an order of magnitude higher than the operating voltage (Figure S9C). Furthermore, an 8-hour continuous withstand voltage test at 1500 V showed stable leakage current, confirming the material's reliability (Figure S9D). When the temperature increased from 20°C to 60°C, the mobility of carriers within the dielectric material rose accordingly. Under a test voltage of 1500V, the leakage current increased to approximately 18nA (Figure S9E), while the insulation properties remained excellent.

TENG Insulation Strategy:

Rolling TENG: The air gap between electrodes is set to 5 mm, which is sufficient to prevent air breakdown at the 1.5 kV operating voltage.

Rotary TENG: For higher integration density, the electrode gaps were reduced to 2 mm and 1.5 mm. Consequently, prior to triboelectric layer deposition, the electrodes were pre-wrapped with PI film. This method effectively suppresses corona discharge and elevates the local breakdown threshold[1], thereby ensuring stable high-voltage output. Its efficacy has been demonstrated in our previous work.

Note S10 The Calculation Process for LC Circuit

Neglecting internal resistance and voltage drop: In the subsequent calculation process, the internal resistance and voltage drop of diodes on the circuit will be temporarily ignored. The specific working process of LC circuit is shown in Figure S10A and S10B, and is divided into two steps. The corresponding current direction and voltage direction of first step are shown in Figure S10A and S10C. The voltage of input capacitor (C_{in}), inductor (L), and output capacitor (C_{out}) are V_{in}, V_L and V_{out}. We assume that when $t = 0$, the initial voltage value of C_{in} is V_{in}, and the initial voltage of C_{out} is 0.

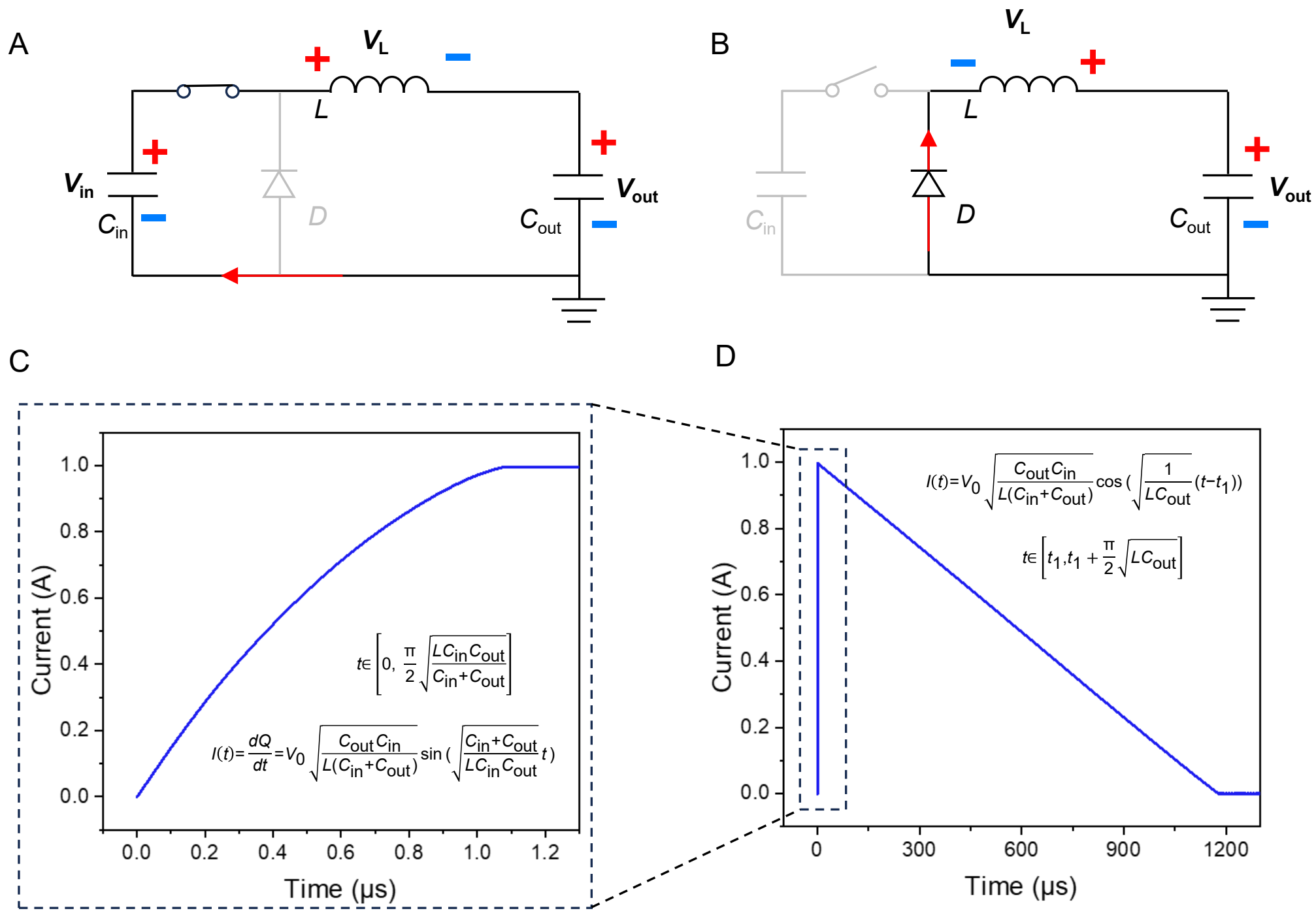

Figure S10. The specific working process of LC circuit. (A) and (B). Schematic diagrams of the circuit at different stages. (C) and (D). Output current at different stages. Source data are provided as a Source Data file.

According to Kirchhoff's voltage law:

$$V_{in}+V_L+V_{out}=0 \tag{9}$$

We assume that transferred charge of C_{in} is Q, so V_T, V_L and V_{out} can be expressed as:

$$V_{in}=\frac{C_{in}V_0\text{-}Q}{C_{in}} \tag{10}$$

$$V_L=\text{-}L\frac{d^2Q}{dt^2} \tag{11}$$

$$V_{out}=\text{-}\frac{Q}{C_{out}} \tag{12}$$

Therefore:

$$V_{\text{in}}=\frac{\mathrm{d}^2Q}{\mathrm{d}t^2}+\frac{Q}{L}\left(\frac{1}{C_{\text{in}}}+\frac{1}{C_{\text{out}}}\right) \tag{13}$$

According to initial condition Q (t = 0) = 0, I (t = 0) = dQ/dt = 0, the solution of differential equation is:

$$Q(\text{t})=-\frac{V_{\text{in}}C_{\text{in}}C_{\text{out}}}{C_{\text{in}}+C_{\text{out}}}\cos\left(\sqrt{\frac{C_{\text{in}}+C_{\text{out}}}{LC_{\text{in}}C_{\text{out}}}}t\right)+\frac{V_{\text{in}}C_{\text{in}}C_{\text{out}}}{C_{\text{in}}+C_{\text{out}}} \tag{14}$$

Therefore, the current of the loop and the voltage of C_{in}, L and C_{out} is:

$$I(t)=\frac{\mathrm{d}Q}{\mathrm{d}t}=V_{\text{in}}\sqrt{\frac{C_{\text{out}}C_{\text{in}}}{L(C_{\text{in}}+C_{\text{out}})}}\sin\left(\sqrt{\frac{C_{\text{in}}+C_{\text{out}}}{LC_{\text{in}}C_{\text{out}}}}t\right) \tag{15}$$

$$V_{\text{in}}(t)=V_{\text{in}}+\frac{V_{\text{in}}C_{\text{out}}}{C_{\text{in}}+C_{\text{out}}}\cos\left(\sqrt{\frac{C_{\text{in}}+C_{\text{out}}}{LC_{\text{in}}C_{\text{out}}}}t\right)-\frac{V_{\text{in}}C_{\text{out}}}{C_{\text{in}}+C_{\text{out}}} \tag{16}$$

$$V_{\text{out}}(t)=-\frac{V_{\text{in}}C_{\text{in}}}{C_{\text{in}}+C_{\text{out}}}\cos\left(\sqrt{\frac{C_{\text{in}}+C_{\text{out}}}{LC_{\text{in}}C_{\text{out}}}}t\right)+\frac{V_{\text{in}}C_{\text{in}}}{C_{\text{in}}+C_{\text{out}}} \tag{17}$$

$$V_{\text{L}}(t)=V_{\text{in}}\cos\left(\sqrt{\frac{C_{\text{in}}+C_{\text{out}}}{LC_{\text{in}}C_{\text{out}}}}t\right) \tag{18}$$

$$t\in\left[0,\ \frac{\pi}{2}\sqrt{\frac{LC_{\text{in}}C_{\text{out}}}{C_{\text{in}}+C_{\text{out}}}}\right] \tag{19}$$

When $t_1=\frac{\pi}{2}\sqrt{\frac{LC_{\text{in}}C_{\text{out}}}{C_{\text{in}}+C_{\text{out}}}}$, the current reach the maximum.

The direction of corresponding current and voltage direction of second step are shown in Figure S10B and S10D. Similarly, according to Kirchhoff's voltage law:

$$V_{\text{L}}+V_{\text{out}}=0 \tag{20}$$

The voltage of L and C_{out} can be expressed as:

$$V_{\text{L}}=-L\frac{\mathrm{d}^2Q}{\mathrm{d}t^2} \tag{21}$$

$$V_{\text{out}}=-\frac{Q}{\text{C}_{\text{out}}} \tag{22}$$

Therefore:

$$\frac{\mathrm{d}^2Q}{\mathrm{d}t^2}+\frac{Q}{LC_{\text{out}}}=0 \tag{23}$$

Considering the initial condition $Q\ (t\text{-}t_1 = 0) = 0$, $I\ (t\text{-}t_1 = 0) = \mathrm{d}Q/\mathrm{d}t = V_{\mathrm{in}}\sqrt{\frac{C_{\mathrm{out}}C_{\mathrm{in}}}{L(C_{\mathrm{in}}+C_{\mathrm{out}})}}$, the solution of differential equation is:

$$Q(t)=V_{\mathrm{in}}C_{\mathrm{out}}\sqrt{\frac{C_{\mathrm{in}}}{C_{\mathrm{in}}+C_{\mathrm{out}}}}\sin\left(\sqrt{\frac{1}{LC_{\mathrm{out}}}}(t-t_1)\right) \tag{24}$$

Therefore:

$$V_{\mathrm{out}}(t)=-V_{\mathrm{in}}\sqrt{\frac{C_{\mathrm{in}}}{C_{\mathrm{in}}+C_{\mathrm{out}}}}\sin\left(\sqrt{\frac{1}{LC_{\mathrm{out}}}}(t-t_1)\right) \tag{25}$$

$$I(t)=V_{\mathrm{in}}\sqrt{\frac{C_{\mathrm{out}}C_{\mathrm{in}}}{L(C_{\mathrm{in}}+C_{\mathrm{out}})}}\cos\left(\sqrt{\frac{1}{LC_{\mathrm{out}}}}(t-t_1)\right) \tag{26}$$

Considering internal resistance and voltage drop: According to the simulation results (Figure S11), when the internal resistance is small (1.82 Ω) and the output voltage is low (5V), the maximum current remains essentially consistent with the case where the internal resistance is 0 Ω and the output voltage is 0 V.

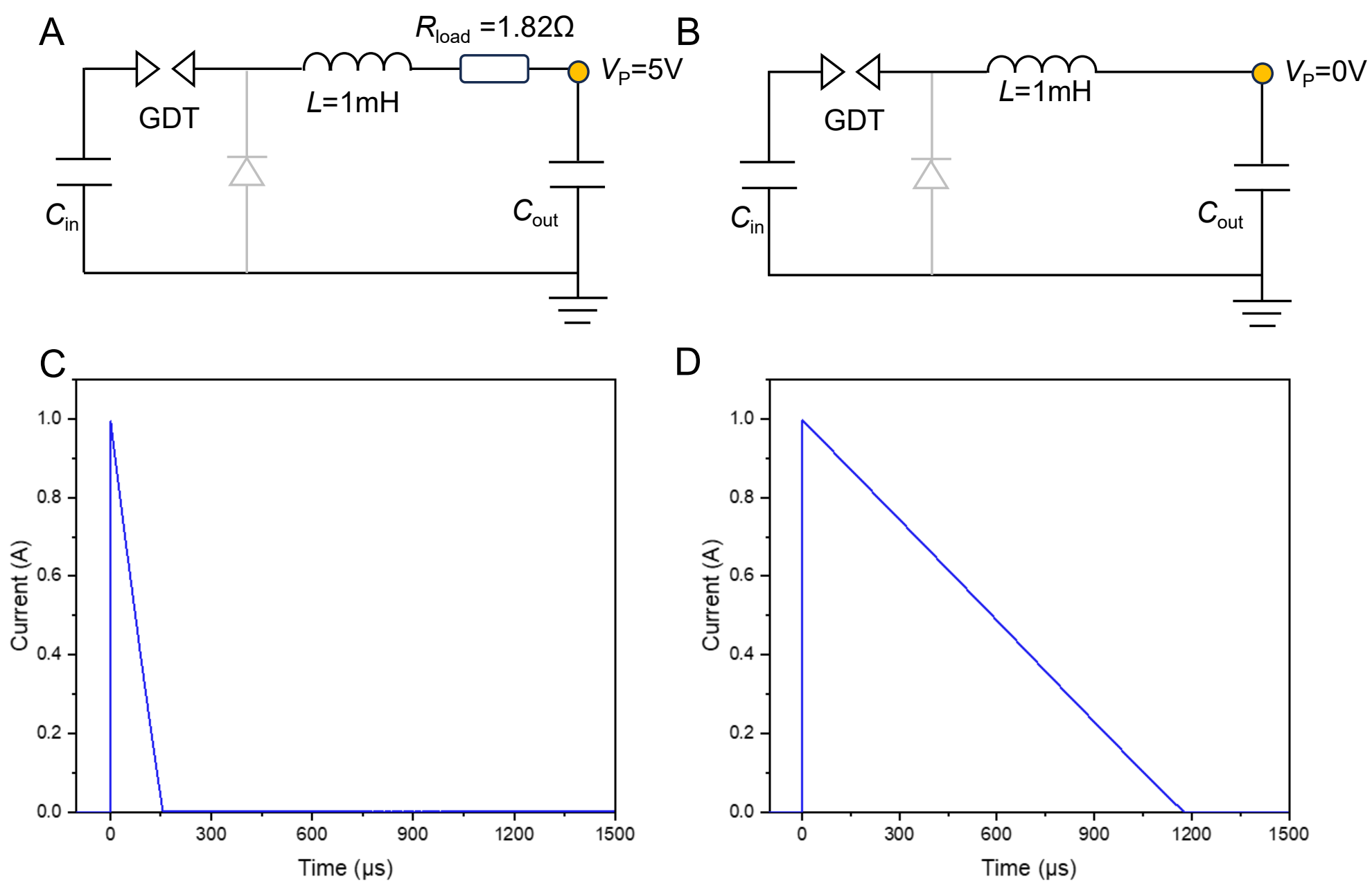

Figure S11. The simulation results of primary buck circuit with or without internal resistance. (A) and (B) Schematic diagram of the circuit with/without internal resistance. (C) and (D) The current of circuit with/without internal resistance. Source data are provided as a Source Data file.

Therefore, according to the formula 15 and 19, the max current of circuit can be calculated as:

$$I_{\max}= V_{\text{in}}\sqrt{\frac{C_{\text{out}}C_{\text{in}}}{L(C_{\text{in}}+C_{\text{out}})}} \tag{27}$$

In the second step of the LC circuit (with the start of the second step as the time zero), the output voltage and the diode's voltage drop remain constant at V_{out} and V_{diode}, respectively.

According to Kirchhoff's voltage law:

$$V_{\text{L}}+V_{\text{out}}+V_{\text{diode}}+V_{\text{R}}=0 \tag{28}$$

$$V_{\text{R}}=IR_{\text{int}} \tag{29}$$

Thus,

$$L\frac{\mathrm{d}I}{\mathrm{d}t}+R_{\text{int}}I+V_{\text{out}}+V_{\text{diode}}=0 \tag{30}$$

When t is 0, $I(t)=I_{\max}$.

Thus,

$$I(t)=\left(I_{\max}+\frac{V_{\text{diode}}+V_{\text{out}}}{R_{\text{int}}}\right)\mathrm{e}^{-\frac{R_{\text{int}}}{L}t}-\frac{V_{\text{diode}}+V_{\text{out}}}{R_{\text{int}}} \tag{31}$$

When $I(T)$ is 0,

$$T=-\frac{L}{R_{\text{int}}}\ln\left(\frac{V_{\text{out}}+V_{\text{diode}}}{R_{\text{int}}I_{\max}+V_{\text{out}}+V_{\text{diode}}}\right) \tag{32}$$

Note S11 Reducing LC Circuit Losses by Lowering Capacitor Leakage and Circuit Internal Resistance

In practical applications, capacitors can be modeled as ideal capacitors in parallel with resistors[3]. Consequently, higher leakage implies lower resistance of capacitor, resulting in greater heat loss. Given the high voltage across the input capacitor, thin-film capacitors are often used as input capacitors to minimize leakage current. Conversely, to reduce output voltage ripple, output capacitors with larger capacitance values are required, leading to the selection of electrolytic capacitors with lower leakage currents as output capacitors.

To lower circuit internal resistance, two main components must be addressed: the resistance of the wires and the resistance of the inductors. Using thicker wires and shortening wire lengths can effectively reduce the circuit's internal resistance. When selecting inductors, priority should be given to meeting voltage, current, and energy requirements, while also choosing inductors with lower internal resistance for the same inductance value.

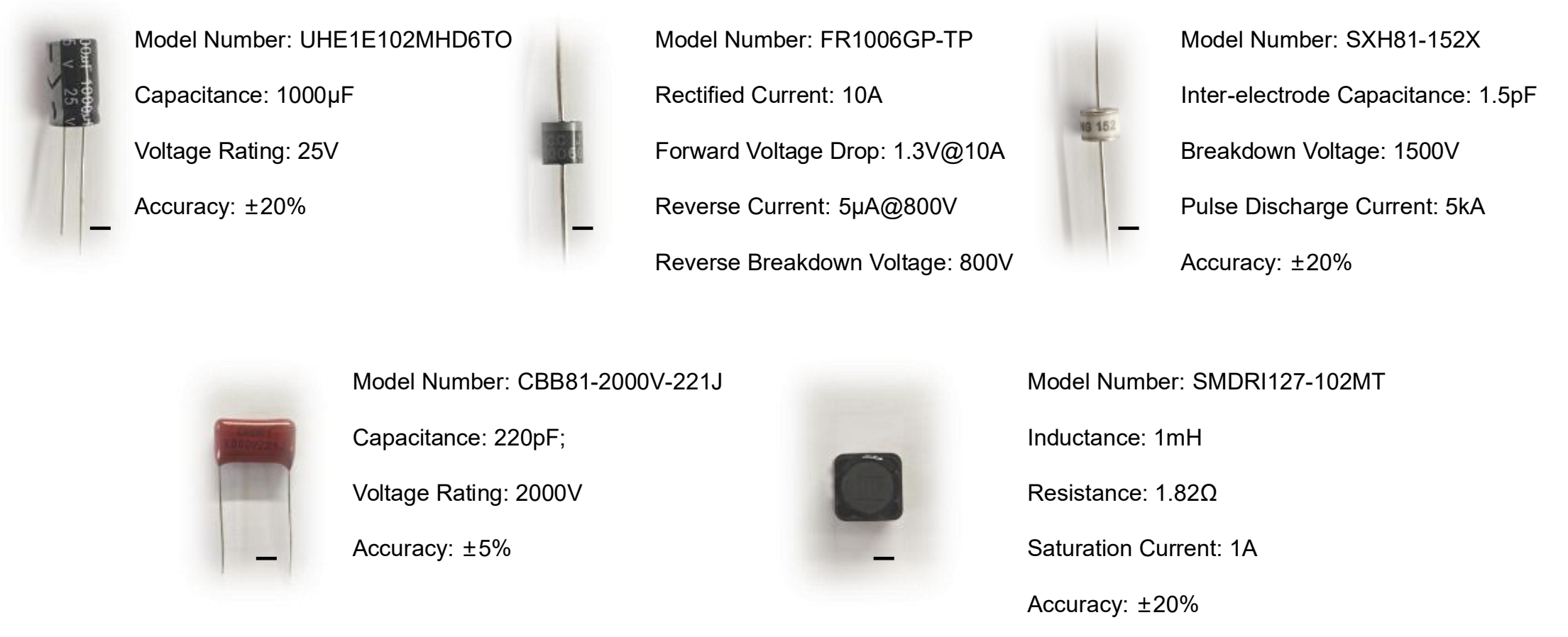

Figure S12. The photos and parameters of electronic components used in the primary circuit. Scale bar: 5 mm

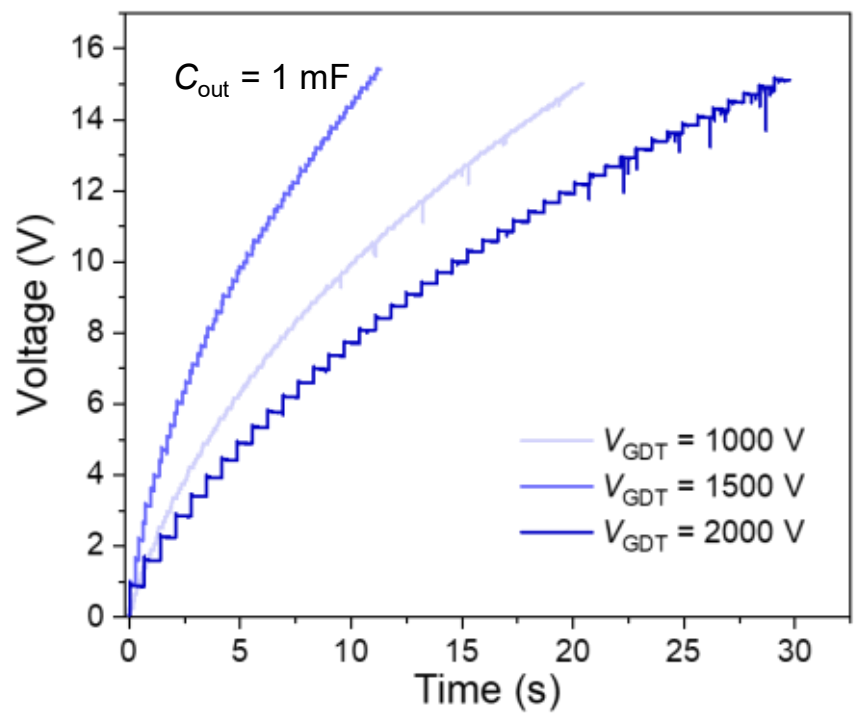

Figure S13. The voltage curve of charging the C_{out} to 15V at different operating voltages for primary buck circuit. Source data are provided as a Source Data file.

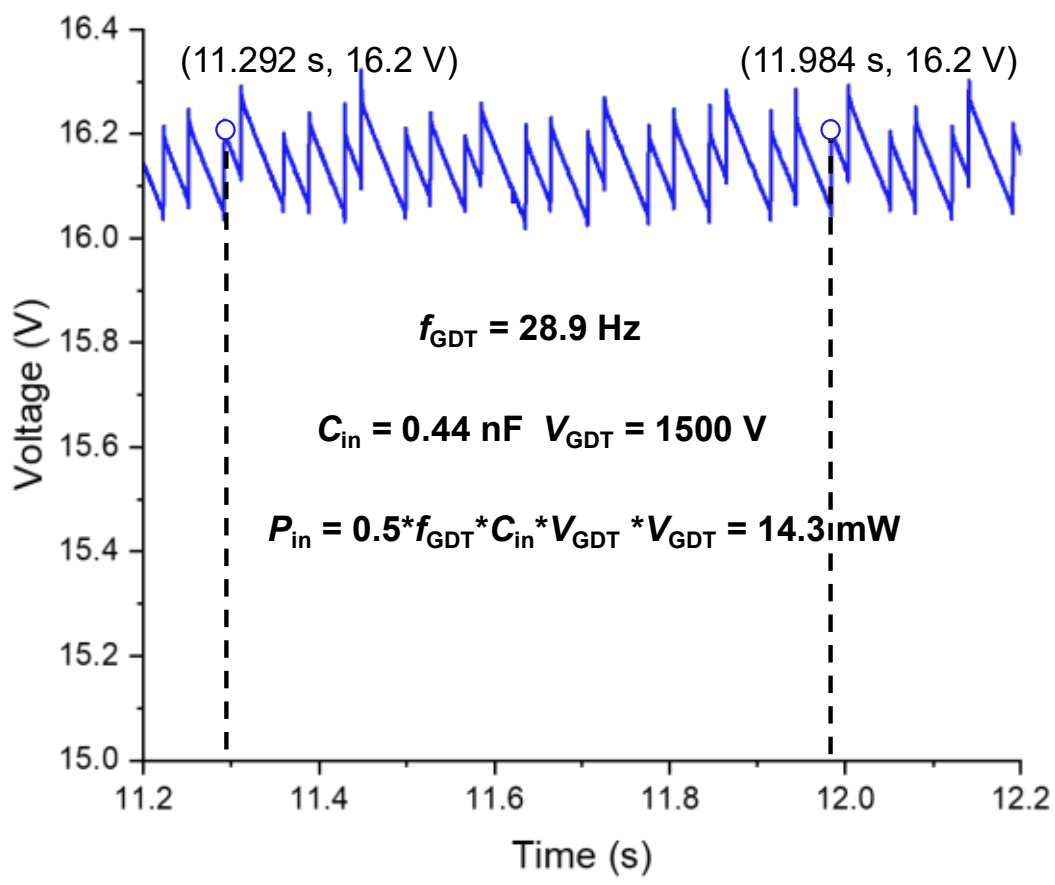

Figure S14. The operation frequency of GDT. Source data are provided as a Source Data file.

Note S12 The Calculation Process for Average Power

The voltage across the resistor was measured by the electrometer (Figure S15A), and the current through the resistor was calculated using formula 33.

$$I=V/R \tag{33}$$

When the resistance was low, the current pulse value was high (Figure S15B). As the resistance gradually increased, the current decreased, and the waveform became more stable. The formula for calculating power is as follows:

$$P=I^2*R \tag{34}$$

Formula 35 is used to calculate the average power (P_{ave}) to avoid the influence of current waveform.

$$P_{ave}=\frac{\int_0^t I^2 dt}{t}R \tag{35}$$

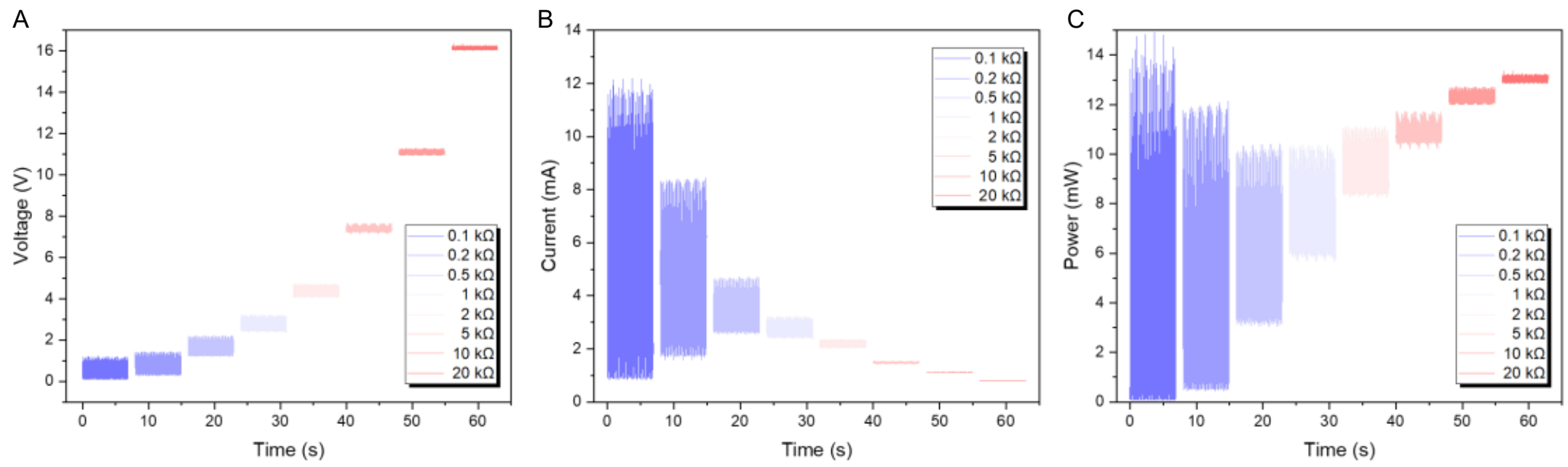

Figure S15. The output performance of TENG with primary buck circuit at different loads. (A) The output voltage. (B) The output current. (C) The output power. Source data are provided as a Source Data file.

Note S13 The Simulation for LC Circuit

The equivalent circuit for the LC circuit is shown in Figure S16A, and the simulation schematic is shown in Figure S16B. In the simulation, a voltage-controlled switch is used to replace the GDT for circuit control. The parameters are as follows: input capacitance (C_{in}) 440 pF, output capacitance (C_{out}) 10 mF, inductance (L) 1 mH, switch threshold voltage 1500 V. The circuit internal resistance (R_{int}, mainly from the inductor) is 1.82 Ω, and the freewheeling diode forward voltage drop (V_{diode}) is 0.83 V (increasing the number of diodes to improve voltage drop). Although a precise GDT switch model was not established, the simulation results of the simplified switch model built based on the parasitic parameters of the GDT indicate that the energy loss caused by the switch is much smaller than that caused by the resistor and the diode (Figure S17). Therefore, it does not affect our analysis, and this model still effectively analyzes the impact of V_{diode} and R_{int} on energy loss.

Figures S16C-G present key waveforms for one operating cycle. Specifically, they show the circuit current, the voltage across the internal resistance, the diode voltage, the input voltage, and the output voltage. Substituting the circuit parameters into Equations (27) and (32) allows calculation of the current duration and the I_{max}. As the voltage increases from 0.5 V to 16 V, the duration decreases sequentially: 473 μs, 379 μs, 272 μs, 175 μs, 103 μs, and 56 μs. Throughout this range, the I_{max} is essentially unchanged at 0.995 A. These calculated values validate the simulation results presented in Figure S16E.

Figures S16H-J show the energy distribution across the diode, internal resistance, and output capacitor. With a total input energy of 495 μJ, most of the energy is distributed among these three components. As the output voltage increases, the energy dissipated by the diode and internal resistance decreases, leading to a higher useful output energy. In addition, increasing the number of diodes raises the diode energy loss at low output voltages, thereby reducing the available output energy. The simulated circuit efficiency in Figure S16K shows a trend consistent with the experimental results (Figure 2M). This trend directly supports our design rationale and the reported high efficiency.

Therefore, in the multi-stage circuit design, an intermediate voltage of 16 V was selected to significantly enhance the efficiency of the primary circuit.

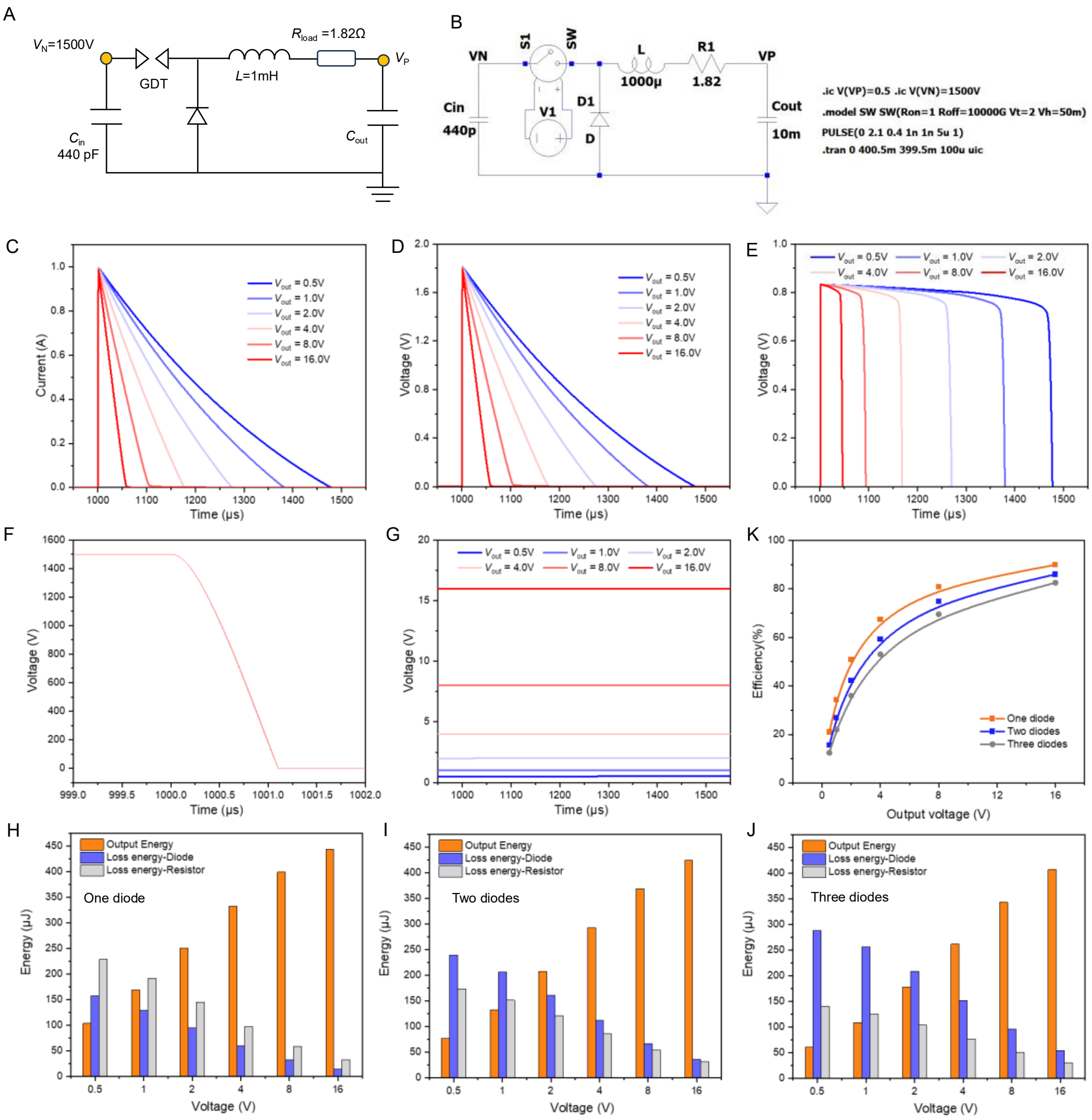

Figure S16. The circuit simulation results. (A) The equivalent circuit of LC circuit; (B) The circuit simulation parameters; (C) The waveform of current; (D) The waveform of voltage across the internal resistance; (E) The waveform of diode voltage; (F) The voltage of input capacitor; (G) The voltage of output capacitor; (H) The loss energy of resistor and one diode and the output energy. V_{diode} is about 0.83 V. (I) The loss energy of resistor and two diodes and the output energy. V_{diode} is about 1.66 V. (J) The loss energy of resistor and three diodes and the output energy. V_{diode} is about 2.49 V. The input energy is 495 μJ. (K) The circuit efficiency. The Source data are provided as a Source Data file.

Regarding the secondary circuit, it is implemented using a commercial integrated circuit (IC). It internally employs synchronous rectification, where integrated

MOSFETs replace Schottky diodes. The on-resistance of these MOSFETs is extremely low (typically tens of milliohms), resulting in minimal voltage drop and lower energy loss during the freewheeling period. Furthermore, the energy losses associated with its internal components are well-characterized in the manufacturer's datasheet. Under the optimized operating conditions achieved by our primary circuit design, the secondary circuit operates with high efficiency. Therefore, additional loss simulations for the secondary circuit were not conducted in here.

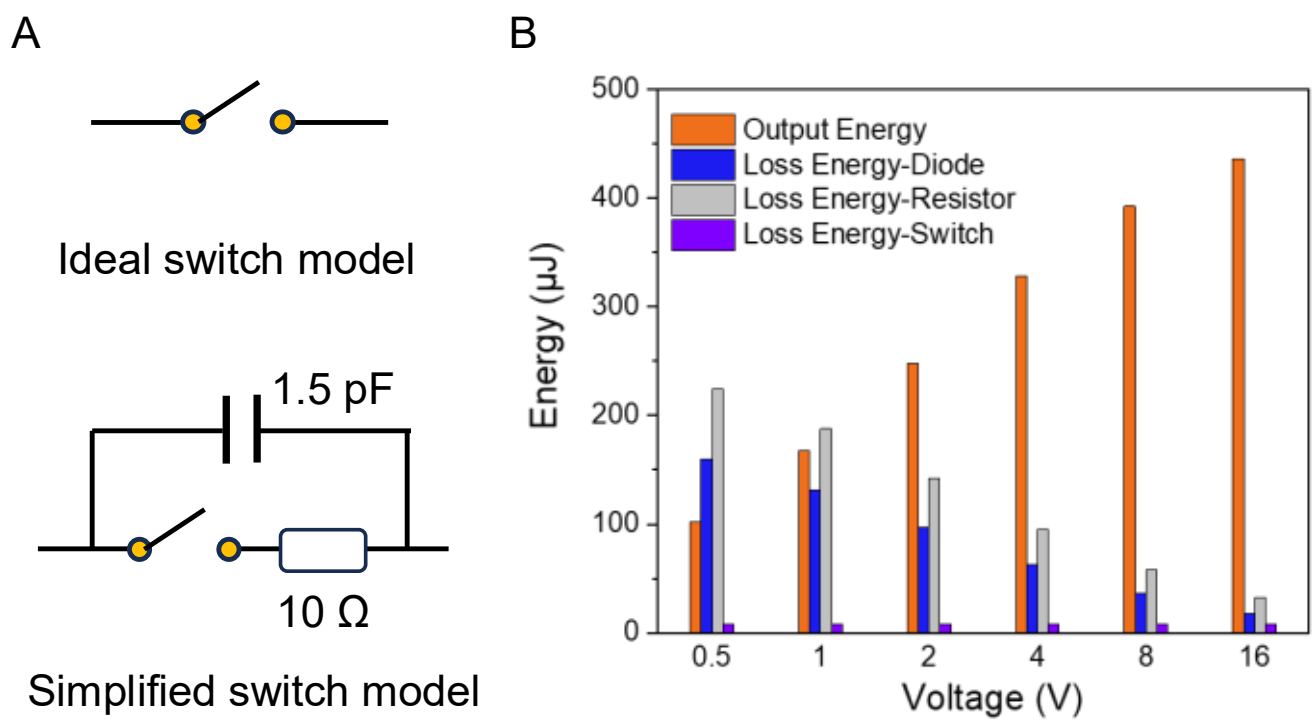

Figure S17. Schematic diagram of a simple circuit model of a gas discharge tube (GDT) switch and its energy loss simulation. (A) Schematic diagram of a simple circuit model of GDT; (B) The results of energy loss simulation. Source data are provided as a Source Data file.

Note S14 Limitations of Existing Power Management Circuits for TENGs

Switched-Capacitor Circuits (SCC): Taking the fractal switched-capacitor converter (FSCC) proposed by Liu et al. as an example[20], its fractal conversion structure effectively mitigates the diode voltage drop (V_{diode}) problem in conventional SCC (Figure S18A), and the total V_{diode} ($V_{diode,\,t}$) is N*V_{diode} (N is the order of FSCC). Ideally (without the V_{diode}), the input energy (the black line in Figure S18B) with an input voltage (V_{in}) and charge (Q_{in}), after FSCC the output voltage reduces to $V_{in}/2^N$ and the charge increases to 2^N*Q_{in} (the orange dashed line and purple dashed line in Figure S18B). However, due to the actual V_{diode}, the practical output performance is illustrated in Figure S18B (the orange line and purple line): the output voltage decreases to $V_{in}/2^N$-N*V_{diode}, and the charge decreases proportionally. This leads to two main issues:

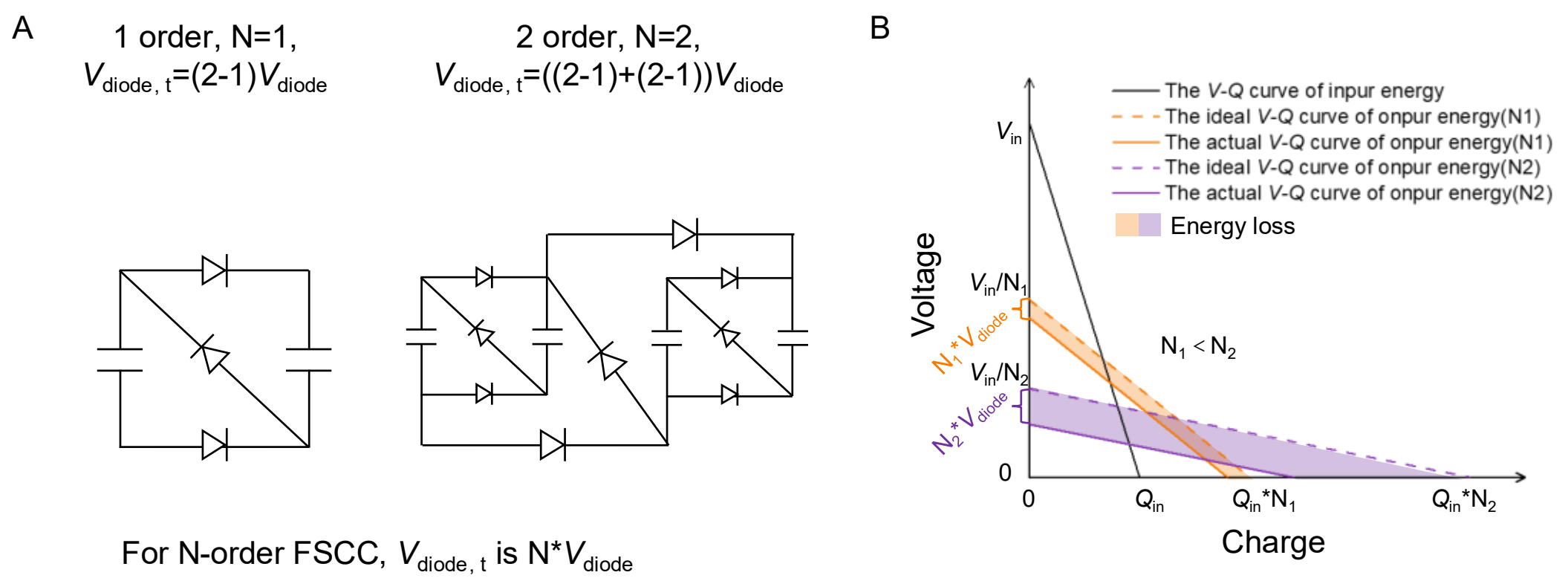

Figure S18. Fractal switched-capacitor converter. (A) Equivalent circuit diagrams (1-order FSCC and 2-order FSCC). (B) *V–Q* plots of input and output energy for the FSCC. The area enclosed by the black curve and the origin represents the input energy. The area enclosed by the orange dashed line and the origin corresponds to the theoretical output energy of the N_1-order FSCC. The area enclosed by the solid orange line and the origin denotes the actual output energy. The orange shaded region indicates the energy loss in practice due to N_1*V_{diode}. Similarly, the area enclosed by the purple dashed line and the origin represents the theoretical output energy of the N_2-order FSCC, while the area enclosed by the solid purple line and the origin shows the actual output energy. The purple shaded area corresponds to the practical energy loss caused by N_2*V_{diode}.

- To match a lower target output voltage, the order of FSCC must be increased. However, this raises the total voltage drop, resulting in lower efficiency. As the output voltage decreases, the proportion of the $V_{diode,\,t}$ increases, leading to greater energy loss (the shadow area in Figure S18B).

- If the order of FSCC is kept constant, although higher circuit efficiency can be maintained, the output may not align well with the target voltage. Consequently, the output energy under low-voltage conditions falls short of the circuit's optimal performance.

Transformer-Coupled Circuits: The efficiency of this circuits is primarily constrained by the following aspects:

- For TENGs that inherently output high-frequency signals, with a fixed transformer turns ratio, variations in output voltage can easily cause the primary coil voltage to deviate from the optimal operating point. This leads to impedance mismatch with TENG, significantly reducing the energy harvested from the device. Even if the output voltage is matched to the target low voltage through turns ratio adjustment, the voltage drop in subsequent rectification stages remains significant, affecting overall efficiency.
- For devices with low-frequency output signals, a spark switch is often used to increase the frequency[21]. Although impedance matching with the TENG output can be achieved via the transformer design, rectification or freewheeling operations are still required after the secondary coil. This process is similar to the secondary stage of LC circuit model and also introduces considerable energy loss under low-voltage conditions (Figure S19).

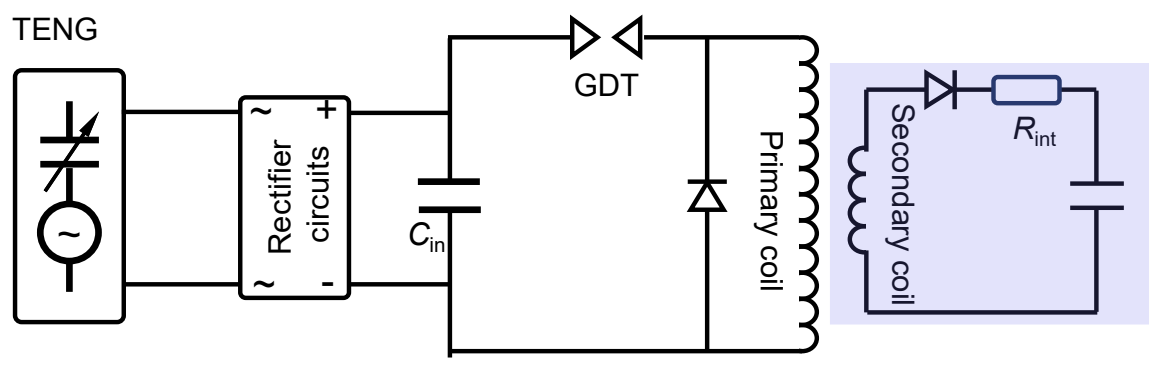

Figure S19. Equivalent circuit diagrams of transformer coupled circuits. The shaded area represents the secondary coil circuit, whose structure is similar to the second stage in the LC circuit (Figure S10B). Consequently, it is also subject to energy loss due to diode voltage drop and circuit internal resistance.

Commercial Energy Harvesting Chips (e.g., LTC3588): While such chips exhibit high internal conversion efficiency (>90%) within their specified input range, their relatively low input voltage requirement is severely mismatched with the high-voltage

(often hundreds of volts) and high-impedance characteristics typical of TENGs. Direct connection results in most of the energy failing to be effectively accepted by the chip input, leading to poor output performance.

Then, we conducted a performance comparison among the LC circuit, transformer-based circuit, commercial chips, and the multi-stage buck circuit proposed in this work (Figure S20A).

Since the theoretical model of FSCC is well-established and sufficiently demonstrates its inherent efficiency degradation under low voltages, while its mechanical switches have limited applicability and are unsuitable for the device presented in this work—coupled with the high complexity (e.g., a 7-order FSCC requires 128 capacitors and 381 diodes)—it was not included in the experimental comparison.

The experimental results are shown in the Figure S20B-H. Under the approximately same output voltage conditions (LTC3588 setpoints: 1.8 V, 3.45 V, and 5.0 V, measured as 1.88/1.88/1.94/1.87V, 3.58/3.58/3.61/3.58V and 5.16/4.96/5.07/5.15V), we compared the circuit performance, and the output current of the multi-stage buck circuit consistently exceeded that of the other three circuits (Figure S20B-D). Both the transformer and LC circuits exhibited a decrease in output power as the output voltage decreased (Figure S20F-G). This further confirms that the performance improvement reported in this work is not due to differences in testing conditions, but addresses the objective issue of low efficiency under low voltage discussed in Note S1.

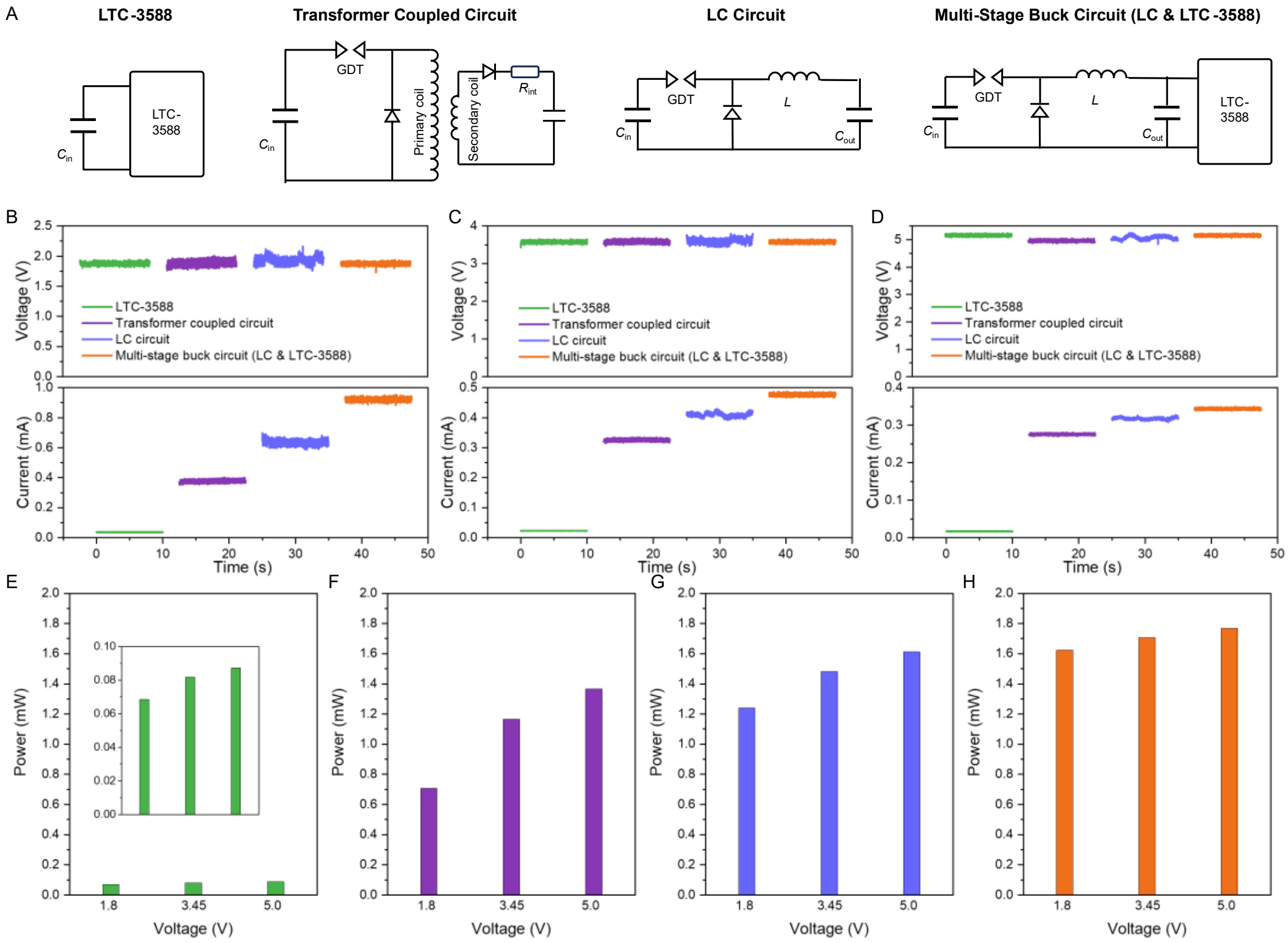

Figure S20. Performance comparison among the LTC3588, transformer coupled circuit, LC circuit, and multi-stage buck circuit. (A) Equivalent circuit diagrams. (B)–(D) Output voltage and current. The output voltage of the multi-stage buck circuit was set to 1.8 V, 3.45 V, and 5.0 V, measured as 1.88/1.88/1.94/1.87V, 3.58/3.58/3.61/3.58V and 5.16/4.96/5.07/5.15V. (E)–(H) Output power. The LTC3588 exhibits lower output power due to its mismatch with the TENG's output characteristics. Moreover, both the LC circuit and the transformer coupled circuit show a decline in output power at lower output voltages. Source data are provided as a Source Data file.

More importantly, after replacing the primary circuit with a transformer coupled circuit and applying the multi-stage buck strategy, the output current also increased significantly (Figure S21). This further demonstrates the broad applicability of the proposed strategy across different primary circuit topologies.

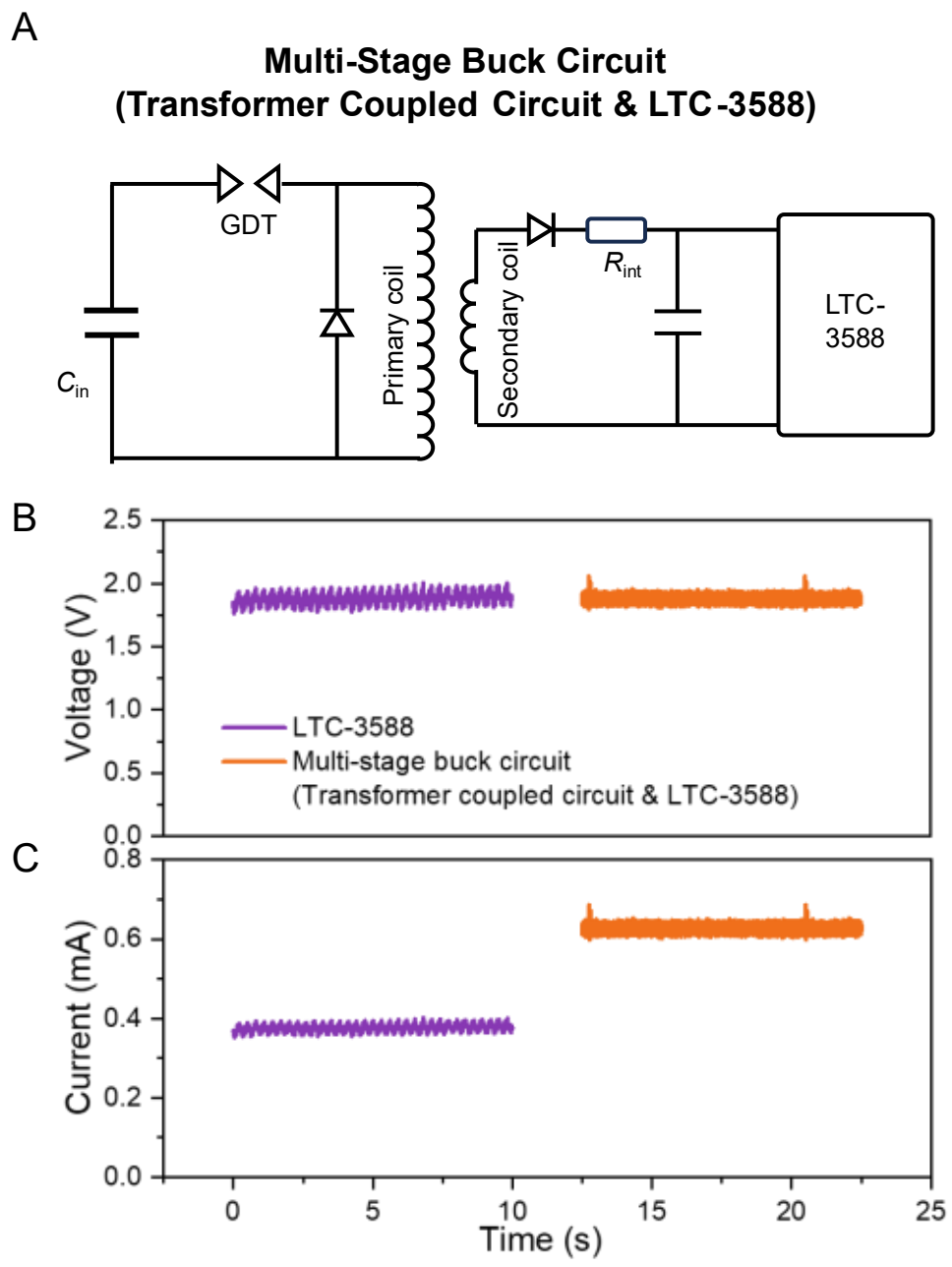

Figure S21. Multi-stage buck circuit based on transformer coupled circuit. (A) Equivalent circuit diagram. (B) Output voltage. (C) Output current. The multi-stage buck circuit achieves an approximately 66.6% increase in output current compared to the transformer coupled circuit. Source data are provided as a Source Data file.

Note S15 The Selection of The Secondary Buck Circuit

When selecting secondary buck circuit, two key considerations should be noted. Firstly, the rated power of the chosen commercial chip should match the output power of the TENG to ensure stable and efficient circuit operation. In this paper, the commercial integrated circuit is LTC-3588, which is suitable for low-power input scenarios (milliwatt-level) and aligns with the output power of the device designed herein. In addition, if high-power applications are targeted, it can be replaced with power chips that offer stronger current-carrying capacity and lower switching losses (such as TPS54331, TPS5430). This modular design endows the circuit architecture with a broad power adaptability.

Secondly, the selection of V_P should guarantee the high efficiency of the primary buck circuit. As illustrated in Figure 3I, setting V_P of 4V can lead to a decrease in the efficiency of the primary circuit, thereby affecting the overall efficiency and output power. Conversely, when V_P is set of 16V, the primary circuit can maintain an efficiency of approximately 90%. Therefore, the setting of V_P should be adjusted based on the actual operating efficiency of the primary buck circuit.

LTC3588-1

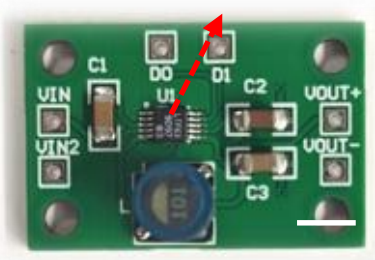

LTC3588-2

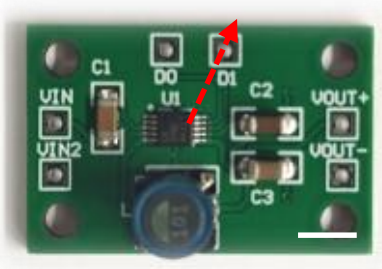

D0	D1	V_{out}(LTC3588-1)	V_{out}(LTC3588-2)
GND	GND	1.8V	3.45V
GND	Vin2	2.5V	4.1V
Vin2	GND	3.3V	4.5V
Vin2	Vin2	3.6V	5.0V

Designator	Description	Model	Parameters
C1	Chip Capacitor	GRM319R71A105KA01D	1uF/10V
C2	Chip Capacitor	GRM31CR71A475KA01K	4.7uF/10V
C3	Chip Capacitor	GRM31CC80J476KE18K	47uF/6.3V
L	Chip Shielded Power Inductor	SLF7045T-101MR50-PF	100uH
U1	Integrated Circuit (IC)	LTC3588EMSE-2/ LTC3588EMSE-1	/

Figure S22. The photos and parameters of electronic components used in the secondary circuit. Scale bar: 5 mm

The commercial integrated circuit chip used in this work is the LTC3588, which comes in two models:

LTC-3588-1, with an input voltage of 4V, allows for adjustable output voltages of 1.8V, 2.5V, 3.3V, and 3.6V via the D0 and D1 pins (Figure S22). LTC-3588-2, designed for higher input voltages, accepts an input of 16V and similarly offers output voltage adjustments of 3.45V, 4.1V, 4.5V, and 5.0V through the D0 and D1 pins (Figure S22). The specific output voltage is determined based on the test results.

In scenarios where the primary circuit operates with a load voltage of 4V, resulting in lower efficiency, we opt to use the LTC3588-2 as the secondary circuit to enhance the overall efficiency of the circuit. When the target output voltage V_Q is 5.0 V or 3.45 V, the LTC3588-2 alone is used as the secondary circuit, corresponding to a step-down path of 1.5/2.5/3.0 kV → 16 V → 5.0/3.45 V. When a lower output voltage (1.8 V or 2.5 V) is required, the LTC3588-1 is cascaded after the LTC3588-2 for further voltage reduction, following a step-down path of 1.5/2.5/3.0 kV → 16 V → 4.1 V → 2.5/1.8 V, while still maintaining a high efficiency. Taking an output voltage of 1.8V as an example, the efficiency of the circuit in this configuration reaches 75.3%, marking a significant improvement over the 57.6% efficiency achieved when using the LTC3588-1 alone (Figure 3I).

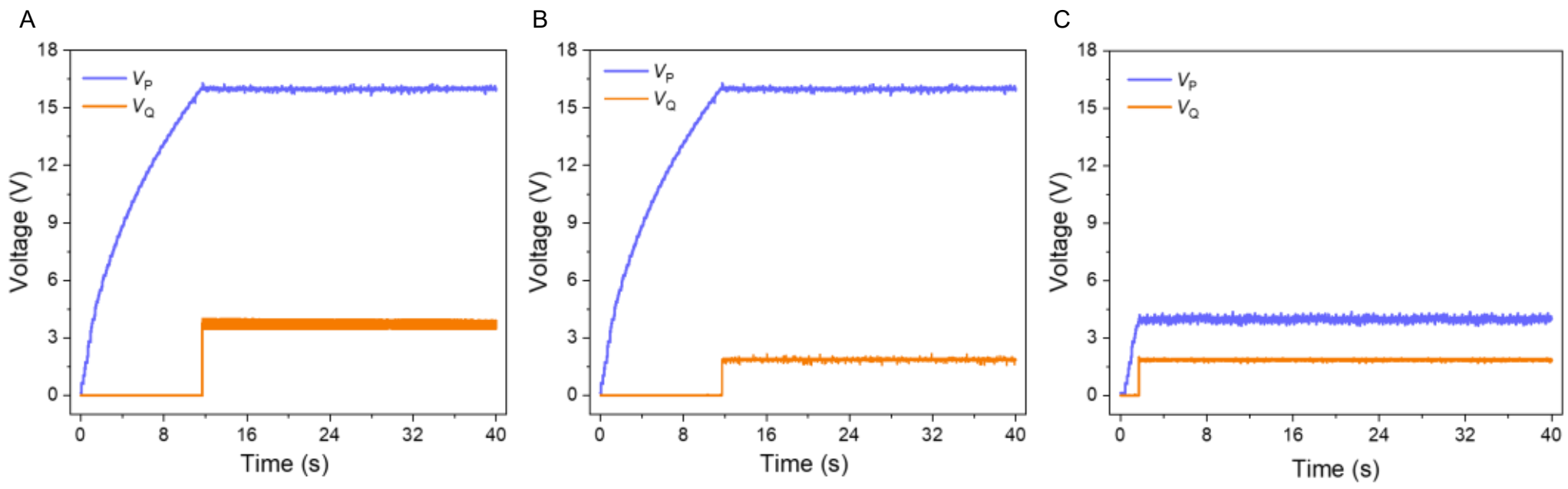

Figure S23. The operation voltages at node P and node Q. (A) The voltage at node Q is set to 3.45V (measured as 3.69 V). (B) The voltage at node Q is set to 1.8V (measured as 1.84 V). (C) The voltage at node Q is set to 1.8V (measured as 1.86 V), while the voltage at node P is set to 4V (measured as 3.98 V). Source data are provided as a Source Data file.

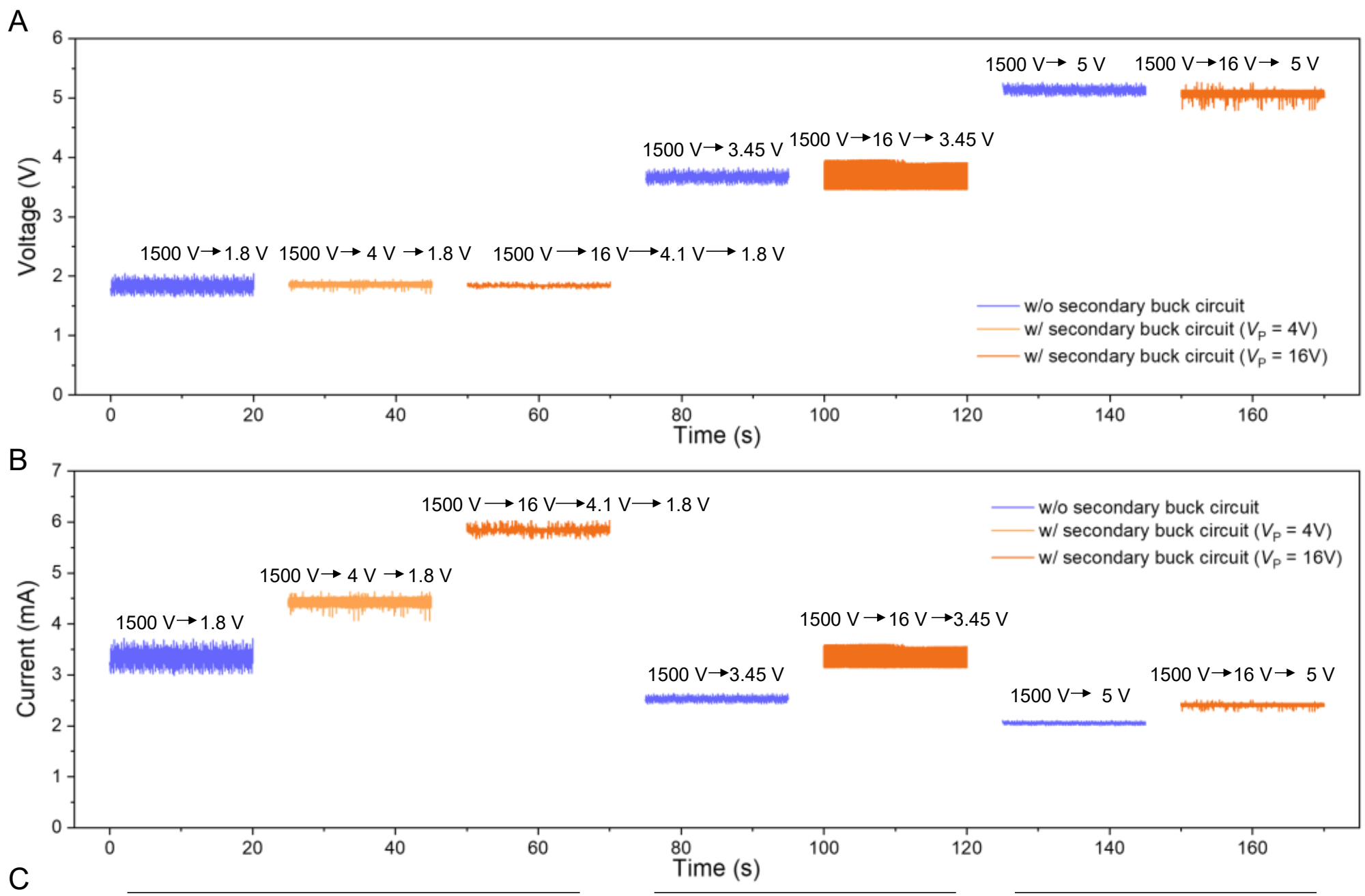

C

	w/o	w/ (V_P=4V)	w/(V_P=16V)
V	1.82 V	1.86 V	1.84 V
I	3.32 mA	4.43 mA	5.84 mA
R_{test}	0.55 kΩ	0.42 kΩ	0.315 kΩ
P	6.05 mW	8.24 mW	10.78 mW
η	42.3%	57.6%	75.3%

	w/o	w/
V	3.66 V	3.69 V
I	2.52 mA	3.35 mA
R_{test}	1.45 kΩ	1.1 kΩ
P	9.23 mW	12.36 mW
η	64.5%	86.4%

	w/o	w/
V	5.13 V	5.06 V
I	2.05 mA	2.41 mA
R_{test}	2.5 kΩ	2.1 kΩ
P	10.52 mW	12.21 mW
η	73.6%	85.4%

Figure S24 Performance comparison of rolling-mode TENG under different step-down paths. (A) The output voltage; (B) The output current; (C) The equivalent voltage (V), equivalent current (I), output power (P), test resistance (R_{test}) and efficiency (η). Source data are provided as a Source Data file.

Table S2 The results of five measurements of circuit efficiency

V_Q	1st	2nd	3rd	4th	5th	Mean	Standard Deviation
1.8V	75.3%	75.4%	75.6%	75.4%	76.3%	75.6%	0.406%
3.45V	86.4%	86%	87.4%	86.8%	85.9%	86.5%	0.616%
5.0V	85.4%	85%	85.3%	85.4%	85.3%	85.28%	0.164%

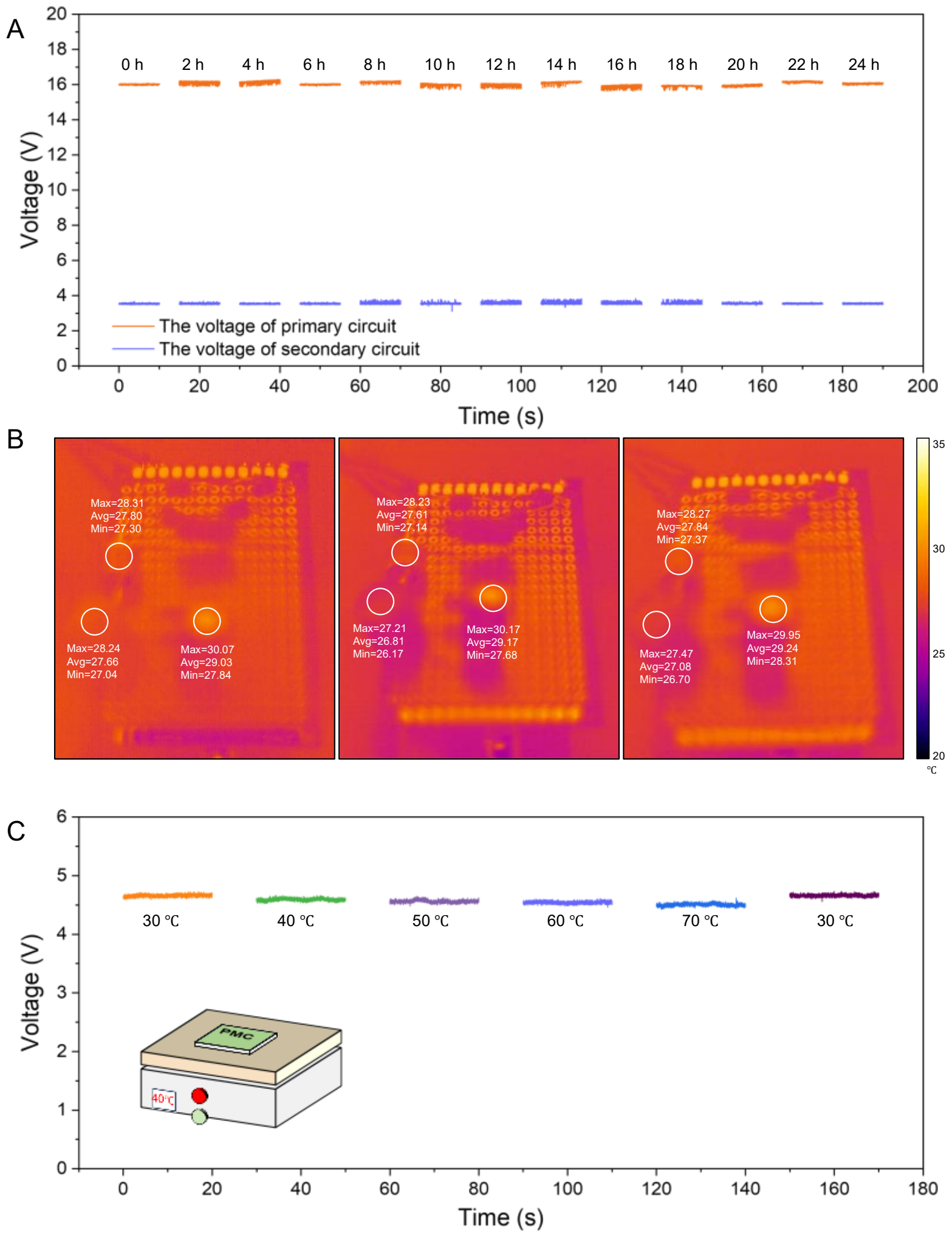

Figure S25. The long-term stability and thermal behavior of the circuit. (A) The output voltage of the circuit after 24 hours of continuous operation. (B) Thermal image of the primary circuit after four hours of continuous operation. The temperature rises on the inductor, capacitor, and GDT were all below 1 ℃, confirming negligible self-heating during this period (30 ℃, 40% RH). (C) Output voltage of primary circuit variation with temperature. The output voltage changed from 4.60 V to 4.51 V (a decrease of ~2.0%) at 70 ℃ and returned to 4.60 V after cooling. Source data are provided as a Source Data file.

Table S3 The reported efficiency in different works*

REF	Circuit	η (~1.8 V)	η (~3.45 V)	η (~5.0 V)	Optimal efficiency
11	LC	32.1%	45.0%	51.1%	81.6%
12	LC	51.3%	68.0%	72.8%	89.8%
17	Transformer	42.6%	55.8%	60.3%	78.5%
19	FSCC	35.2%	62.5%	78.7%	94.5%
23	LC	18.3%	37.8%	48.1%	90.6%
24	LC	35.6%	51.6%	59.1%	87.7%
This Work	Multi-stage buck circuit	75.3% (1.84 V)	86.4% (3.69 V)	85.4% (5.06 V)	90.9%

*The data for these works presented in the table were extracted from the performance curves or values reported in the respective references.

Note S16 Efficiency Comparison of Different Works

The difference between η_{PMC} between η_{total}: The calculation of "efficiency" varies fundamentally in different works[22]. Some works use the maximum output power of the TENG under a resistive load as the denominator, and the "efficiency" calculated seems to reflect the TENG's total energy utilization rate (η_{total}, e.g., refs. 11, 17, 19 in Figure 3J). For instance, in ref. 11, the maximum output power is 1.09 mW at 2 GΩ, and the maximum output with PMC is 0.89 mW, yielding an efficiency of 81.6 %. Other works adopt the actual input energy to the PMC as the denominator, representing the actual conversion efficiency of the circuit (η_{PMC}, e.g., refs. 12, 23, 24 in Figure 3J and this work). For example, in ref. 12, with an input of 280.5μW and an output of 251.9 μW, the efficiency is 89.8%.

The efficiencies reported in this work belong to the actual conversion efficiency of the PMC. To enable a fair and unified comparison across different works, and particularly to highlight the severe efficiency drop of conventional circuits at low voltages, all data were normalized.

Normalization Method: For each referenced work, we divided all efficiency data across various voltages by the highest efficiency value reported in respective work. Thus, the ordinate "Normalized efficiency" represents the efficiency of a circuit at a given voltage as a percentage of its own best performance. This approach effectively eliminates systematic deviations arising from differences in “efficiency” definitions, allowing all data to be compared on a common baseline, which is the degree of performance drop relative to its own optimum.

Taking ref. 19 in Figure 3J as an example, the circuit reported in that work exhibits a peak efficiency of 94.5% at 7.7 V. When the voltage drops to approximately 1.8 V, its efficiency decreases to 35.2%. After normalization: 35.2% / 94.5% ≈ 37.2%. This indicates that at 1.8 V, the performance of this circuit is only 37.2% of its optimal value.

Data Extraction and Comparison: Following the normalization method described above, we extracted the original data points from the relevant figures in all cited

references and calculated the corresponding “normalized efficiency” using the highest efficiency value reported in each work. For a clearer and more direct comparison, the circuit efficiencies at approximately 1.8 V, 3.45 V, and 5.0 V are summarized in the Table S3 and Table S4. With the secondary circuit integrated, the efficiency of our design at 1.84 V remains as high as 82.8% of its optimum value, demonstrating that our strategy can sustain circuit performance much closer to the peak level, especially under low-voltage conditions.

Summary: The core objective of this normalization method is to focus on the performance characteristic of "circuit efficiency drop with decreasing voltage." This effectively avoids the inherent differences in absolute efficiency values. It robustly demonstrates that, compared to conventional approaches, our strategy can maintain high performance more effectively across a wide voltage range, especially at low voltages, with a significantly smaller degree of performance decay.

Table S4 The normalized efficiency in different works*

REF	Circuit	η (~1.8 V)	η (~3.45 V)	η (~5.0 V)	Optimal efficiency
11	LC	39.3%	55.1%	62.6%	100%
12	LC	57.1%	75.7%	81.1%	100%
17	Transformer	54.3%	71.1%	76.8%	100%
19	FSCC	37.2%	66.1%	83.3%	100%
23	LC	20.2%	41.7%	53.1%	100%
24	LC	40.6%	58.7%	67.4%	100%
This Work	Multi-stage buck circuit	82.8% (1.84 V)	95.0% (3.69 V)	93.9% (5.06 V)	100%

*The data for these works presented in the table were extracted from the performance curves or values reported in the respective references.

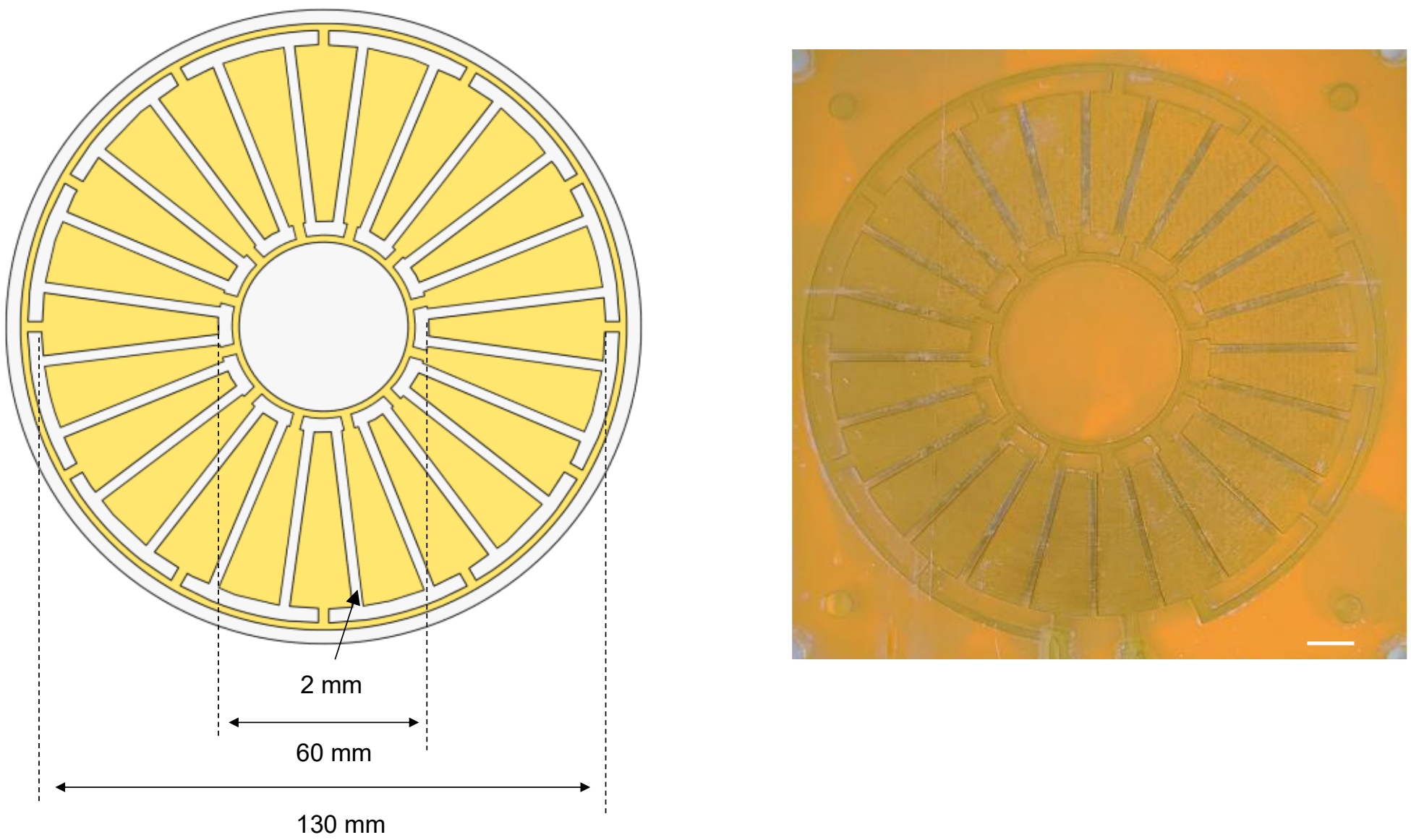

Figure S26. The schematic diagram of the electrode structure of rotary TENG with the triboelectric layers PTFE and fur. Scale bar: 1 cm

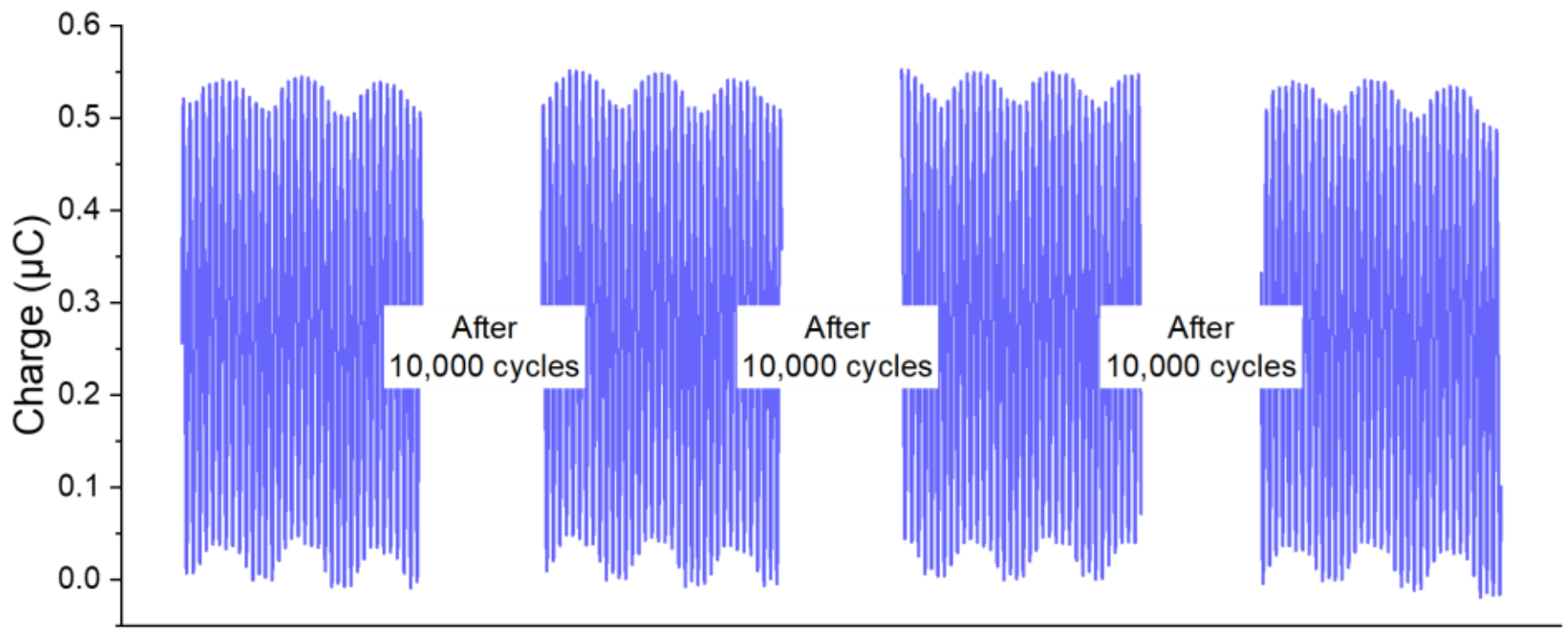

Figure S27. **The output charge of rotary mode FS-TENG after 30,000 cycles.** Source data are provided as a Source Data file.

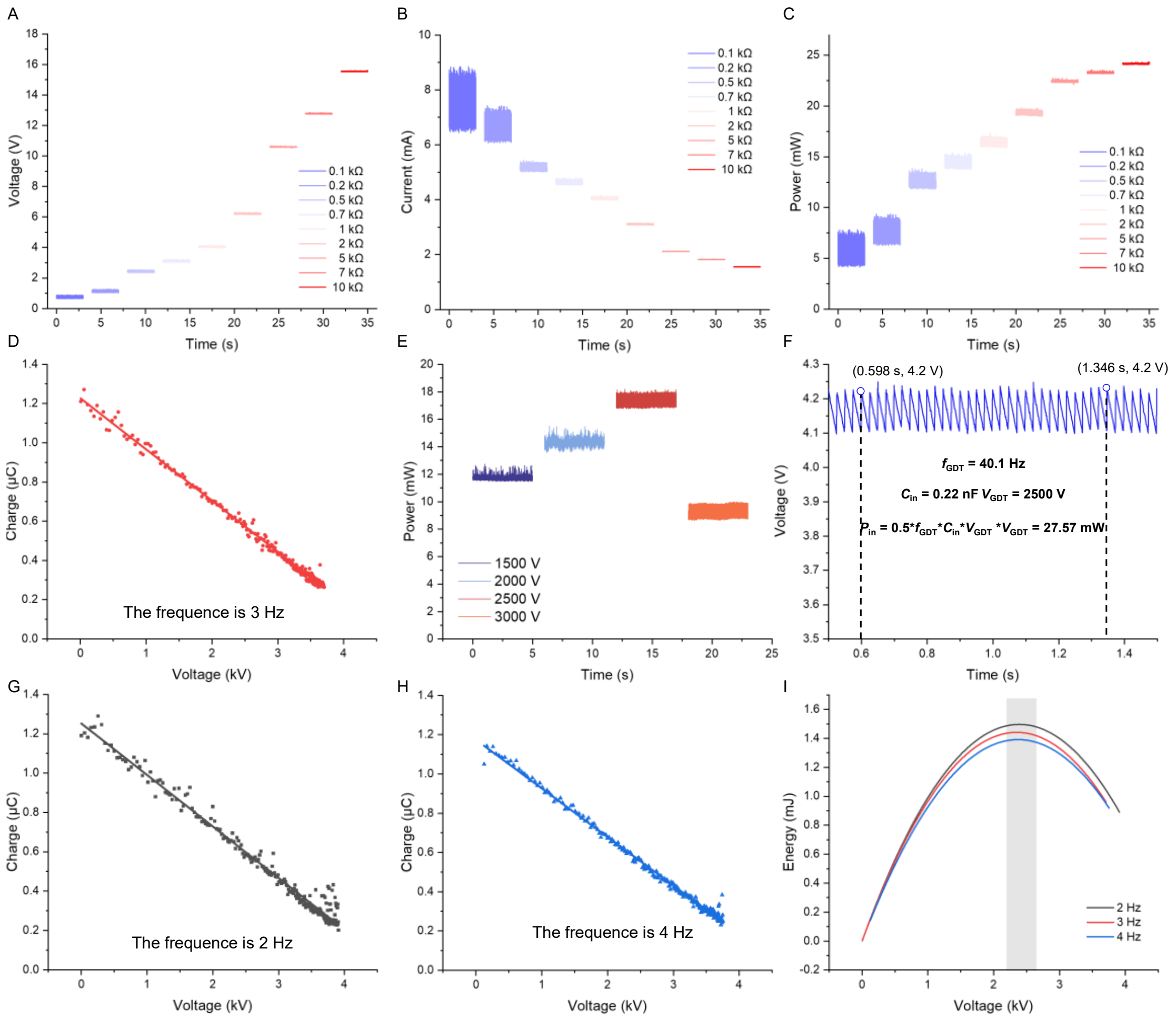

Figure S28. The output performance of rotary TENG with triboelectric layers PTFE and fur. (A) The output voltage of TENG with primary circuit. (B) The output current of TENG with primary circuit. (C) The output power of TENG with primary circuit. (D) The *Q*-*V* curve of TENG at 3 Hz. (E) The output power of primary circuit with different operation voltage. (F) The operation frequency of GDT. (G) The *Q*-*V* curve of TENG at 2 Hz. (H) The *Q*-*V* curve of TENG at 4 Hz. (I) The *E*-*V* curve of TENG. The optimal operating voltage of the primary circuit remains constant when the TENG's operating frequency is 2-4 Hz. Source data are provided as a Source Data file.

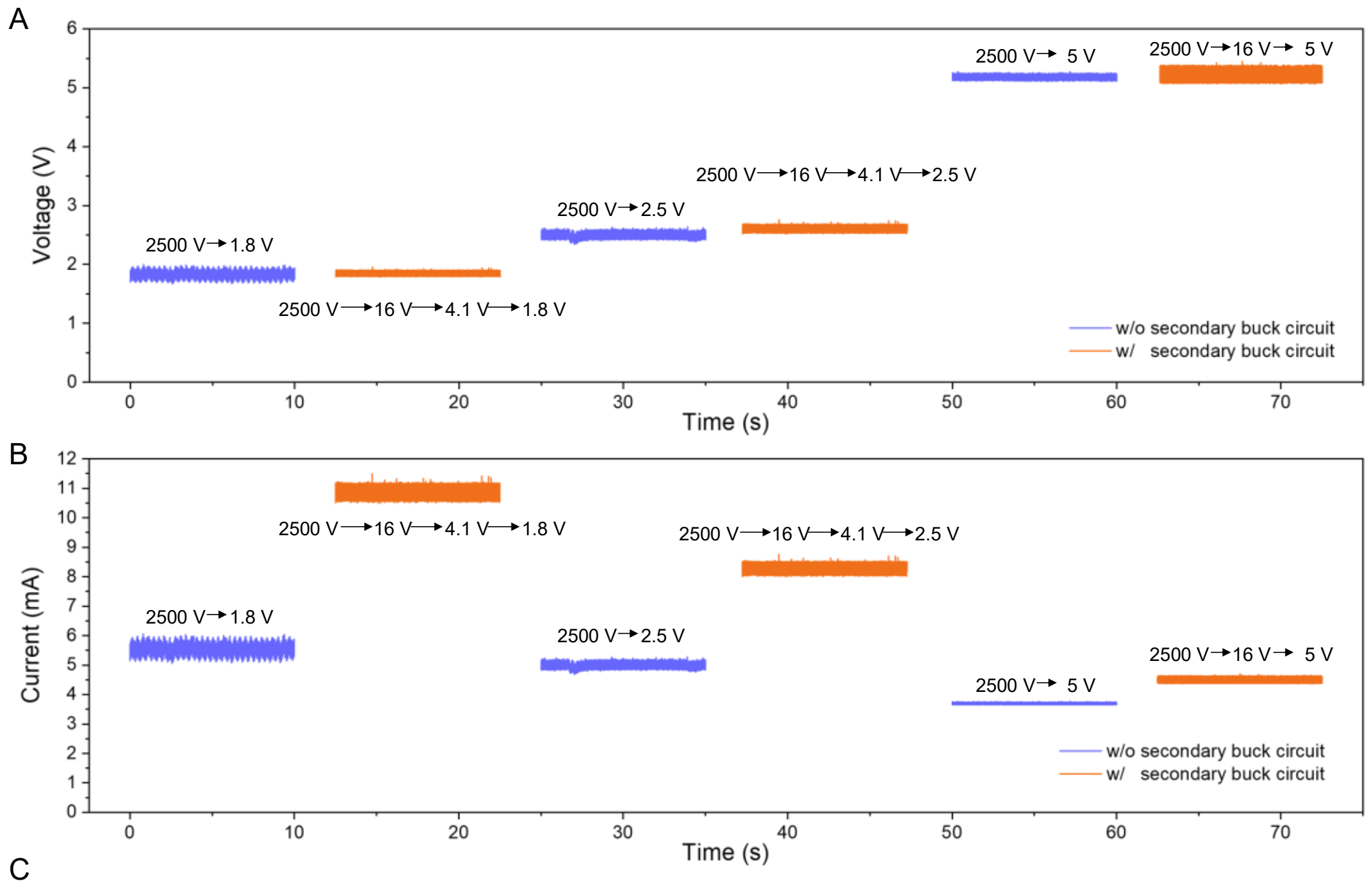

C

	w/o	w/
V	1.82 V	1.85 V
I	5.52 mA	10.89 mA
R_{test}	0.33 kΩ	0.17 kΩ
P	10.05 mW	20.15 mW
η	36.5%	73.1%

	w/o	w/
V	2.49 V	2.61 V
I	4.99 mA	8.29 mA
R_{test}	0.5 kΩ	0.315 kΩ
P	12.45 mW	21.66 mW
η	45.1%	78.6%

	w/o	w/
V	5.18 V	5.20 V
I	3.70 mA	4.48 mA
R_{test}	1.4 kΩ	1.16 kΩ
P	19.15 mW	23.28 mW
η	69.5%	84.4%

Figure S29 Performance comparison of rotary-mode TENG with 12 pairs of electrodes under different step-down paths. (A) The output voltage; (B) The output current; (C) The equivalent voltage (V), equivalent current (I), output power (P), test resistance (R_{test}) and efficiency (η). Source data are provided as a Source Data file.

Note S17 Application of The Multi-Stage Buck Strategy for Rotary TENGs

Here, PTFE and PA are chosen as the triboelectric layers for the rotary TENG due to their high charge transfer efficiency. The detailed structure is shown in Figure S30A-C. At a rotation frequency of 1 Hz, the output charge and current are presented in Figures S30D and Figure S30E, respectively. The frequency of the GDT is 22.1 Hz, C_{in} is 0.22 nF, and the operating voltage of the GDT is 3000 V. Therefore, the input power is 21.88 mW (Figures S30F). Figures S30G and Figure S31 demonstrates that the efficiency of the primary circuit is 77.3% at an output voltage of 18.4V, but it decreases at voltages below 5V. However, upon introducing the secondary circuit and adjusting the output voltages to 5 V, 3.45 V, 2.5 V, and 1.8 V (measured as 4.92/5.08 V, 3.46/3.51 V, 2.54/2.53 V, and 1.81/1.87 V), the current density increased from 1.59A/m^2, 1.76 A/m^2, 2.23 A/m^2 and 2.43 A/m^2 to 2.04 A/m^2, 2.63 A/m^2, 3.65 A/m^2 and 5.19 A/m^2, respectively, and the power density increased from 7.83 W/m^2, 6.11 W/m^2, 5.65 W/m^2 and 4.41 W/m^2 to 10.35 W/m^2, 9.23 W/m^2, 9.25 W/m^2 and 9.70 W/m^2, respectively, (Figure S30H-K, detailed data comparison is shown in the Figure S32).

It is worth noting that since the primary circuit operates at a high voltage of 3000V, four diodes are connected in series to meet the voltage withstand threshold requirement. As a result, the efficiency of the primary circuit is more significantly constrained under low-voltage conditions, making the performance improvement achieved by our strategy more pronounced. At a low voltage of 1.8V (measured as 1.81/1.87 V), the circuit performance is enhanced by 120.1%. In addition, the efficiency of the primary circuit is below 80% at 16 V, which inevitably affects the overall circuit efficiency. If other commercial chips are selected to further increase the input voltage of the secondary circuit, the system performance could be further improved.

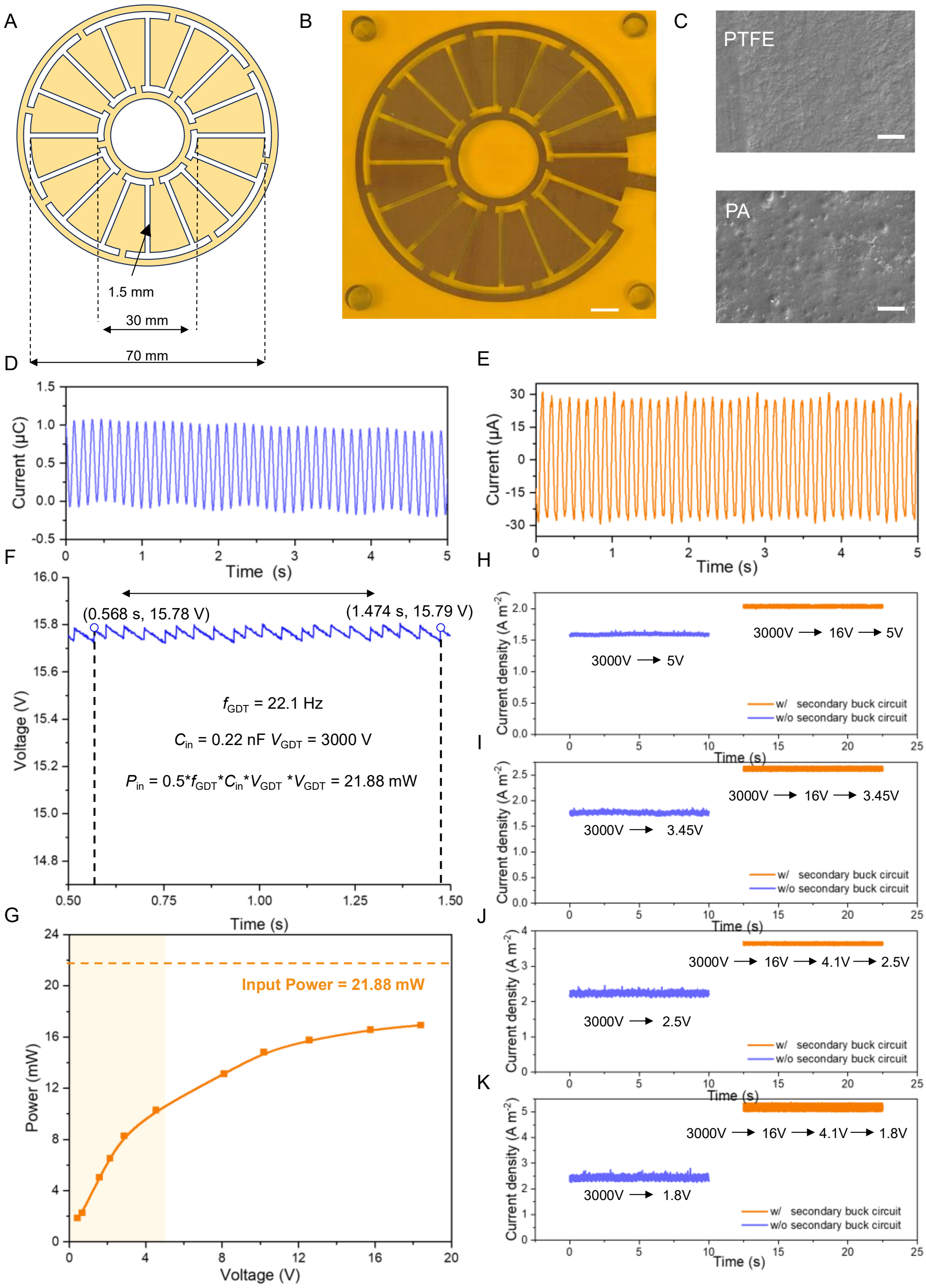

Figure S30. The output performance of rotary TENG with triboelectric layers PTFE and PA. (A) The schematic diagram of the electrode structure. (B) The photo of electrode structure. Scale bar: 1 cm. (C) The SEM images of PTFE and PA. Scale bar: 20 μm. (D) The output charge. the surface charge density is about 800 μC/m^2. (E) The output current. (F) The operation frequency of GDT. (G) The output power of TENG with primary buck circuit. (H)-(K) The output current density. Source data are provided as a Source Data file.

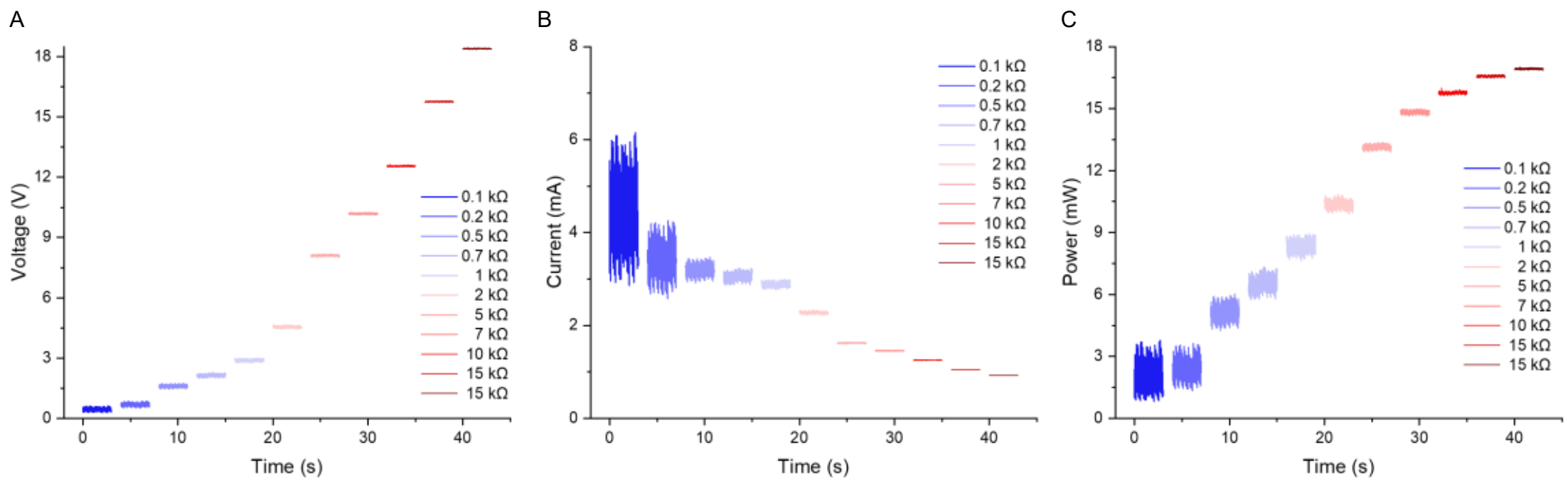

Figure S31 The output performance of 8-pair electrodes TENG with primary buck circuit at different loads. (A) The output voltage; (B) The output current; (C) The output power of TENG with primary circuit. Source data are provided as a Source Data file.

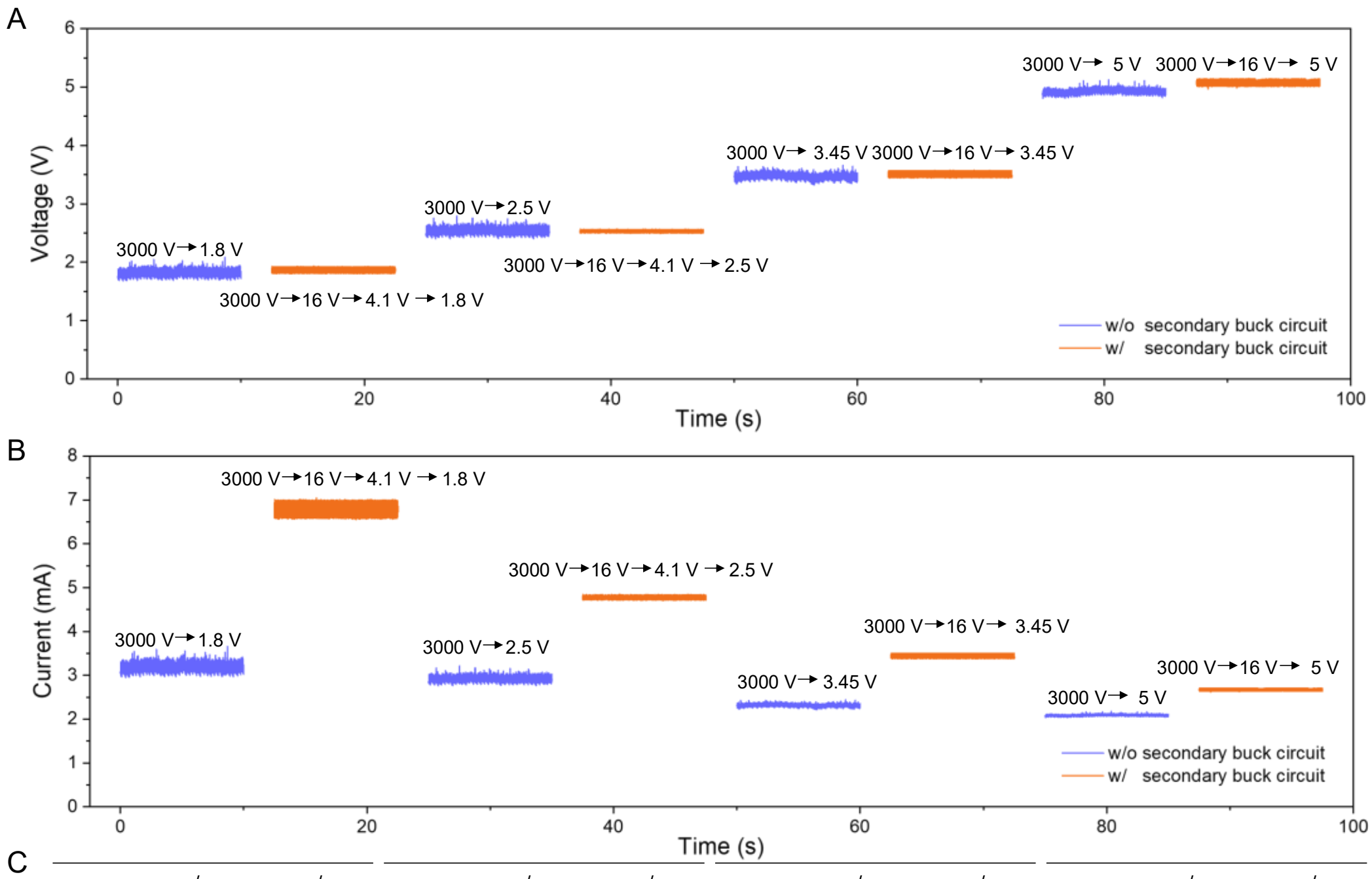

C

	w/o	w/		w/o	w/		w/o	w/		w/o	w/
V	1.81 V	1.87 V	V	2.54 V	2.53 V	V	3.46 V	3.51 V	V	4.92 V	5.08 V
I	3.18 mA (2.43 A m^{-2})	6.8 mA (5.19 A m^{-2})	I	2.92 mA (2.23 A m^{-2})	4.78 mA (3.65 A m^{-2})	I	2.31 mA (1.76 A m^{-2})	3.44 mA (2.63 A m^{-2})	I	2.08 mA (1.59 A m^{-2})	2.67 mA (2.04 A m^{-2})
R_{test}	0.57 kΩ	0.275 kΩ	R_{test}	0.87 kΩ	0.53 kΩ	R_{test}	1.5 kΩ	1.02 kΩ	R_{test}	2.36 kΩ	1.9 kΩ
P	5.78 mW (4.41 W m^{-2})	12.71 mW (9.70 W m^{-2})	P	7.40 mW (5.65 W m^{-2})	12.11 mW (9.25 W m^{-2})	P	8.01 mW (6.11 W m^{-2})	12.09 mW (9.23 W m^{-2})	P	10.26 mW (7.83 W m^{-2})	13.56 mW (10.35 W m^{-2})
η	26.4%	58.1%	η	33.8%	55.4%	η	36.6%	55.3%	η	46.9%	62.0%

Figure S32 Performance comparison of rotary-mode TENG with 8 pairs of electrodes under different step-down paths. (A) The output voltage; (B) The output current; (C) The equivalent voltage (V), equivalent current (I), output power (P), test resistance (R_{test}) and efficiency (η). Source data are provided as a Source Data file.

Table S5 The current density/charging rate and power density for different works*

Ref.	Area (cm^2)	Frequency (Hz)	Current (mA)/ Charging rate ($mC\ s^{-1}$)	Current density (A/m^2)	Power density (W/m^2)
26	95.8	1.5	1.1	0.12	0.17
27	66.1	1	2.95	0.44	0.68
28	100	1	2.2	0.22	0.33
29	26.3	2	0.64	0.24	0.34
30	34.7	2	0.9	0.26	0.38
25	110	1	3.7	0.34	0.63
44	1	2	0.05	0.52	0.78
31	33.2	2	2.35	0.71	1.41
32	62.8	0.375	0.66	0.11	0.23
This work PTFE/fur	43.8	3	10.89 (1.85 V)	2.49 (1.85 V)	4.60 (1.85 V)
This work PTFE/PA	13.1	1	6.80 (1.87 V)<br>4.78 (2.53 V)<br>3.44 (3.51 V)<br>2.67 (5.08 V)	5.19 (1.87 V)<br>3.65 (2.53 V)<br>2.63 (3.51 V)<br>2.04 (5.08V)	9.70 (1.87 V)<br>9.25 (2.53 V)<br>9.23 (3.51 V)<br>10.35 (5.08V)

*Some works did not report the relevant current density data; therefore, comparisons were made based on the average charging rate when the capacitor was charged to approximately 3V.

Note S18 Analysis of output voltage ripple and stability in multi-stage buck circuit

A low output voltage ripple is crucial for the stable operation of electronic devices. This is well achieved in our design. From an energy perspective, when the TENG delivers an average input power P_{in} at a switching frequency f, the energy transferred per cycle (E) that is available at the output satisfies:

$$E = \eta \cdot \frac{P_{in}}{f} = \frac{1}{2} C_{out}(V_{max}^2 - V_{min}^2) \tag{36}$$

Where C_{out} is the output capacitance, and V_{max} and V_{min} are the peak and valley voltages, respectively. This relationship shows that for a constant output power, increasing the operating frequency f is an effective way to reduce the voltage ripple (V_{max} - V_{min}). Here, the secondary circuit (LTC-3588) operates at a high switching frequency, which ensures that the final output voltage ripple is suppressed to merely tens of millivolts (Figure S33A and Figure S33B).

Additionally, in a conventional single-stage circuit, intermittent high-current and high-power events (such as the periodic data acquisition and transmission by 16 hygrothermograph, Figure S33C and Figure S33D) can cause significant voltage fluctuations at the output. In our design, the first circuit (LC circuit) with its large storage capacitor (1 mF) acts as a stable energy buffer, and the secondary circuit (LTC-3588) provides high-bandwidth voltage regulation. These ensuring that the final output voltage remains stable and unaffected by backend power surges.

Furthermore, the long-term stability of the system under continuous operation is shown in Figure S25A, confirming the robustness and endurance of circuit. In summary, the combination of a high-frequency circuit and a multi-stage design with sufficient energy buffering effectively minimizes output voltage ripple and ensures stable power delivery under dynamic loads.

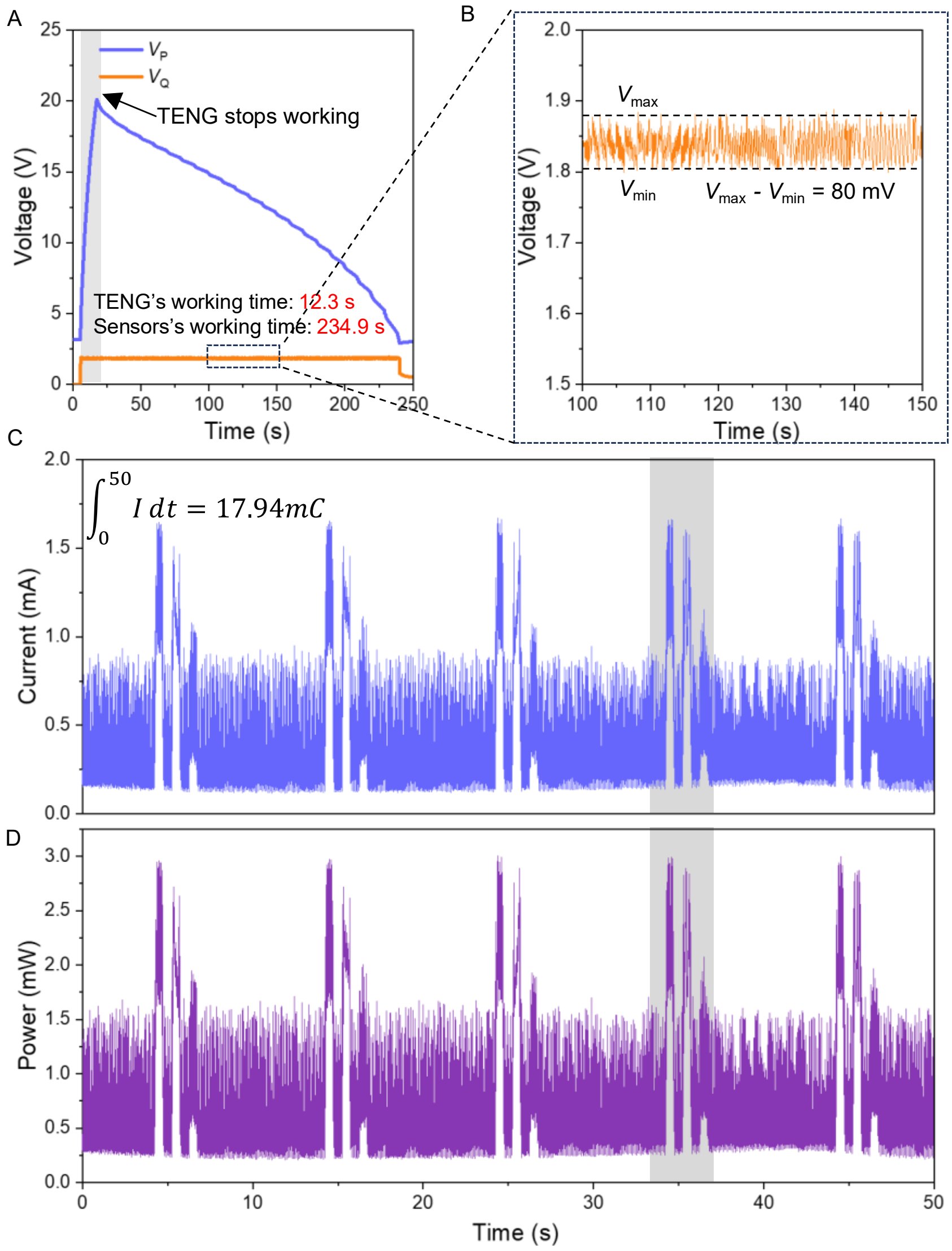

Figure S33. The analysis of voltage ripple for 16 temperature and humidity sensors. (A) Primary and secondary circuit output voltage, (B) secondary circuit output voltage ripple (80 mV), (C) operating current, and (D) operating power of sensors. Source data are provided as a Source Data file.

Note S19 Temperature and Humidity Sensors

The 16 temperature and humidity sensors utilized in this work are connected in parallel, operating at a voltage of approximately 1.8V, with their rated currents illustrated in the Figure S33C. By integrating the current over time (0 s-50 s), we can calculate that these devices consume a total charge of 17.94 mC within 50 seconds. For comparison, a 1 mF capacitor charged to 20V stores 20 mC of charge. Hence, without the secondary buck circuit, these sensors can only operate for roughly 55.7 seconds.

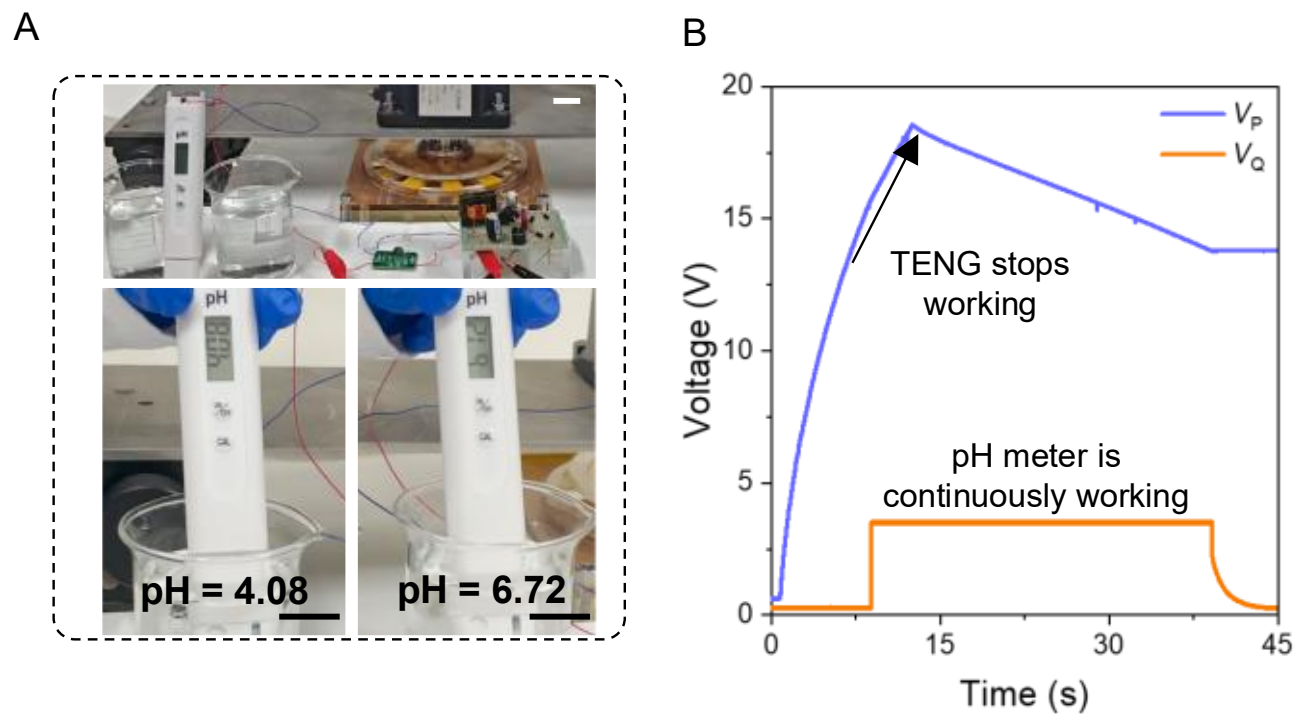

Figure S34. TENG power pH meter. (A) The photos pH meter. The pH values of the solution are 4.08 and 6.72, respectively Scale bar: 3 cm. (B) The output voltage of multi-stage buck circuit. Source data are provided as a Source Data file.

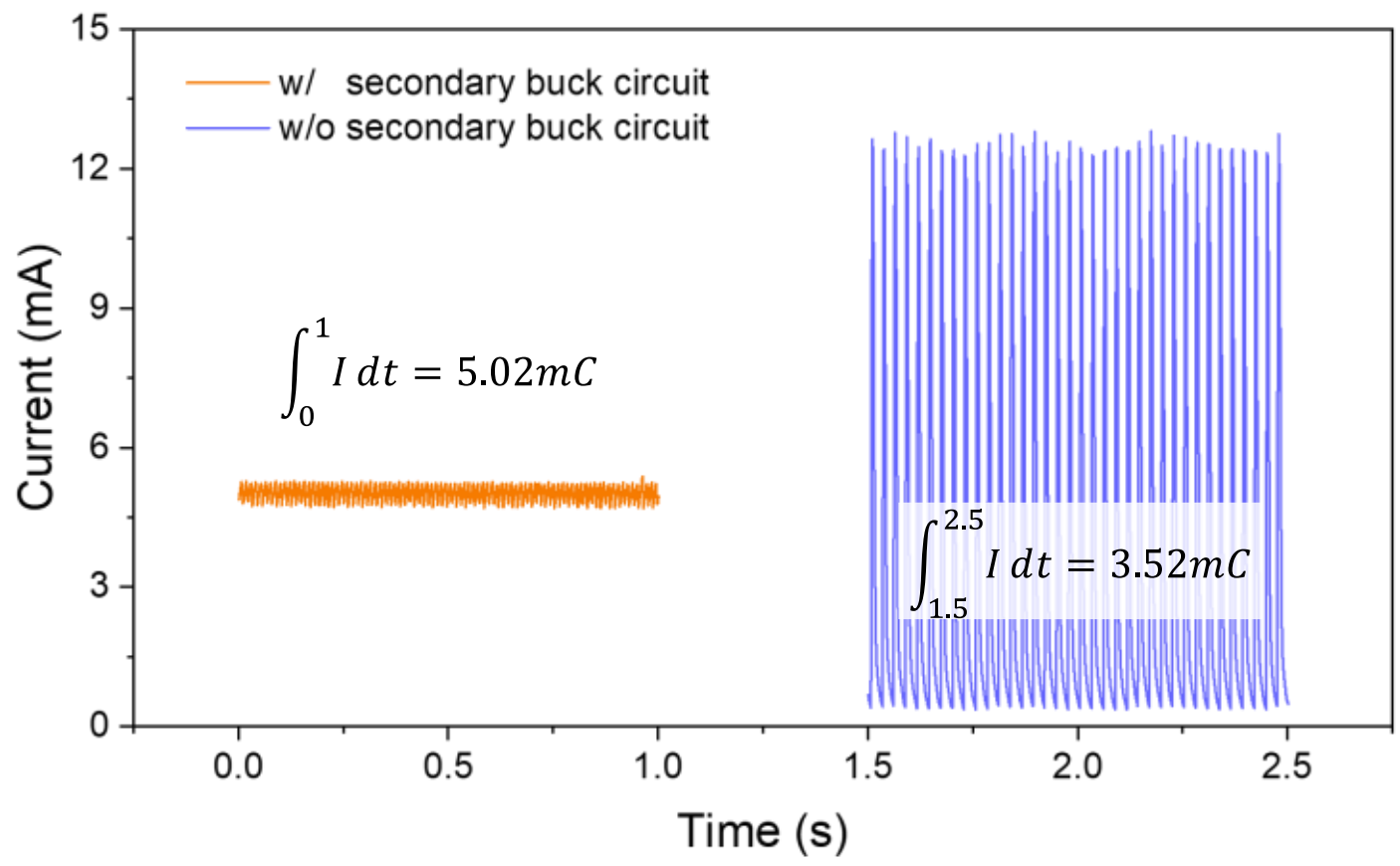

Figure S35. The charging current of lithium-ion battery. Source data are provided as a Source Data file.

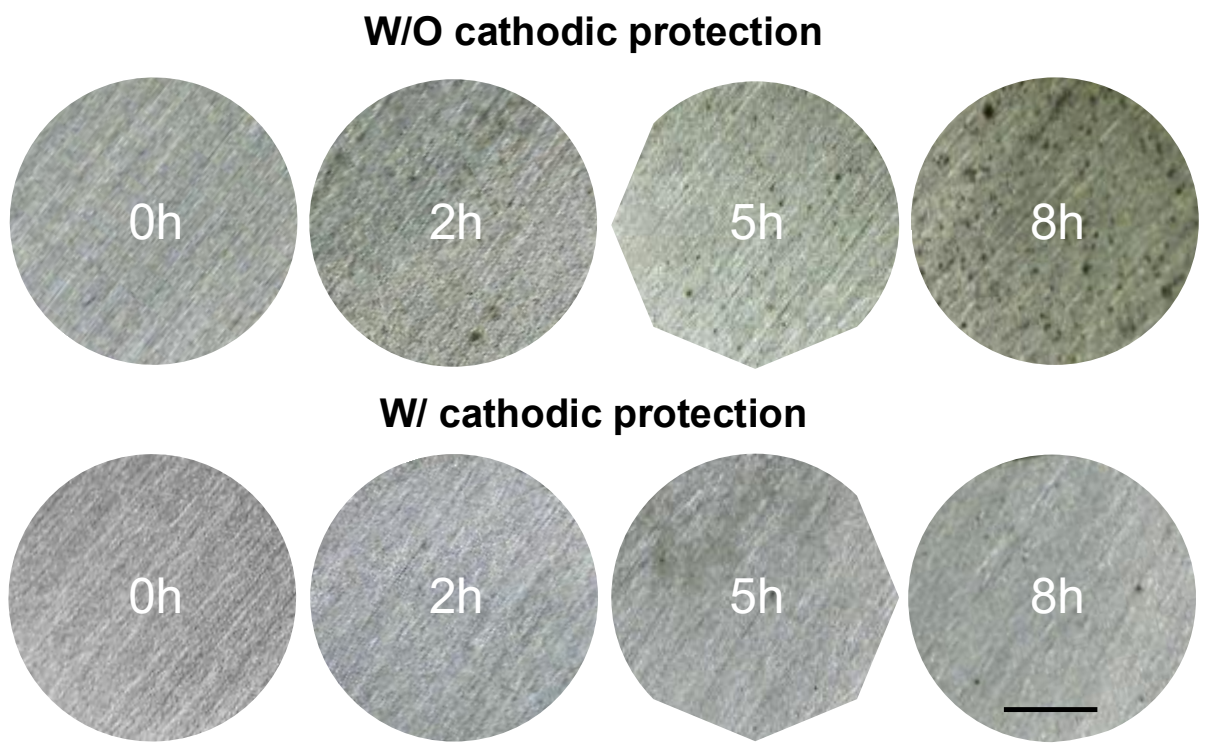

Figure S36. Cathodic protection of metals. Scale bar: 2 mm

Note S20 TENG-Driven Electrochemical Hydrogen Production

In this work, hydrogen production was measured using the volumetric displacement method. The anode is a ruthenium iridium titanium electrode, and the cathode is a graphite electrode. The concentration of the NaCl solution used is 1 mol/L. The time was recorded at every 25 μL increment based on the graduated cylinder scale, with 100 μL constituting one measurement cycle. Five independent tests were performed. The results are summarized in the Table S6 below.

Table S6 The production rate of hydrogen and faradaic efficiency

	The 1st cycle					The 2nd cycle					The 3rd cycle				
Time (s)	0	18	39	64	86	0	25	46	75	84	0	21	42	68	84
Yield (μL)	0	25	50	75	100	0	25	50	75	100	0	25	50	75	100
Production rate (μL min^{-1})	69.77					71.4					71.4				
Faradaic efficiency	98.46%					96.27%					95.29%				
	The 4th cycle					The 5th cycle									
Time (s)	0	17	35	55	80	0	22	43	60	81					
Yield (μL)	0	25	50	75	100	0	25	50	75	100					
Production rate (μL min^{-1})	75.00					74.07									
Faradaic efficiency	97.63%					97.63%									

The average hydrogen production rate was determined to be 72.33 μL min^{-1} (3.23 μmol/min), which corresponds to 16.5 mL/(min·m^2) (calculated by the effective working area of the TENG). The average faraday efficiency measured is 97.06%. The corresponding current data are presented in the Figure S37.

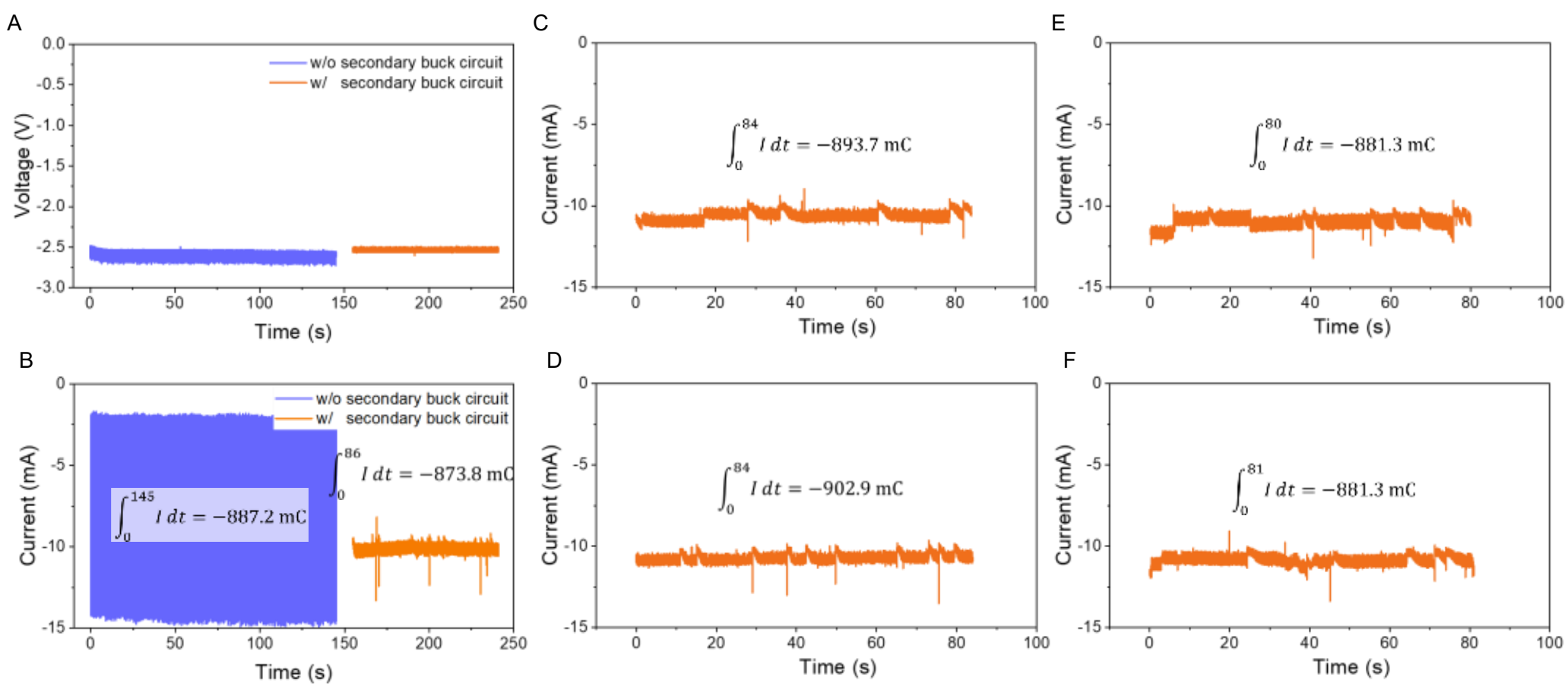

Figure S37. The operating voltage current of the self-power hydrogen production system. (A) Test voltage with/without secondary buck circuit; (B)-(F) Test current. Source Data are provided as a Source Data file.

TENG serves solely as a power source to supply voltage and current for the electrochemical reaction. Therefore, provided that both the TENG and the circuit operate stably, the final hydrogen output maintain stability. The stability of the TENG and the circuit is shown Figure S27 and Figure S25A. Additionally, stability tests were performed by measuring the time required to produce 250 μL of hydrogen, comparing the initial measurement with that taken after one hour (Table S7). The results confirm a stable production rate over time.

Table S7 The stability tests of production rate

	Initial value	After 1 hour
Time (s)	211	205
Yield (μL)	250	250
Production rate (μL min^{-1})	71.43	73.17

In the hydrogen production process studied here, the cathode reaction is:

$2H_2O + 2e^- \rightarrow H_2\uparrow + 2OH^-$

The anode may undergo two reactions:

1. $2Cl^- - 2e^- \rightarrow Cl_2$

   $Cl_2 + 2OH^- \rightarrow Cl^- + ClO^- + H_2O$

2. $2H_2O \rightarrow O_2 + 4H^+ + 4e^-$

To identify the dominant anode pathway, we quantified ClO^- production using ultraviolet-visible (UV-Vis) absorption spectroscopy. The principle is as follows: under acidic conditions, ClO^- oxidizes excess iodide (I^-) to iodine (I_2), which further combines with I^- to form triiodide (I_3^-). I_3^- exhibits a characteristic absorption peak in the UV region (288 nm, shown in Figure S38A). Its absorbance is proportional to the I_3^- concentration, allowing ClO^- concentration to be determined via a calibration curve (Figure S38B).

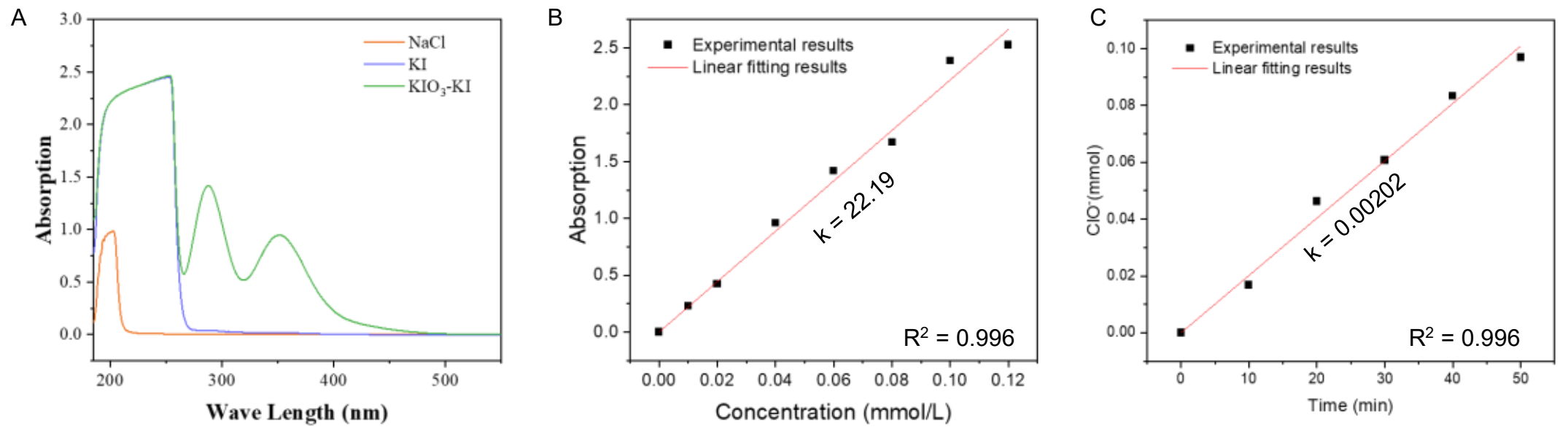

Figure S38 UV-Vis spectra test. (A) UV-Vis spectra of NaCl, KI and KIO_3-KI solution and (B) the standard curve of KI solution for calibrating ClO^- concentration. (C) ClO^- production rate.

During the experiment, a 1 mL sample was taken from solution every 10 min and treated sequentially with:

- 0.1 mol/L KI (providing excess I^-)
- 1 mol/L H_2SO_4 and 0.5 mol/L KOH were used to adjusted PH to about 3.5.
- The mixture was diluted to 25 mL, reacted for 10 min, and then analyzed by UV-Vis.

The measured ClO^- production rate was 2.02 μmol/min (Figure S38C). Based on the stoichiometry of the reaction between anodically generated Cl_2 and OH^- ($Cl_2 + 2OH^- \rightarrow Cl^- + ClO^- + H_2O$), this rate is directly equivalent to the rate of Cl_2 generation that subsequently reacted to form ClO^-. This indicates that at least 62.5% of the anode current was utilized for Cl_2 generation that contributed to the measured ClO^- (H_2 production rate is 3.23 μmol/min).

Gas collected from the anode via water displacement yielded a much lower rate of 7.43 μL/min (0.33 μmol/min) (Table S8). Given the high aqueous solubility of Cl_2, this collected gas is primarily composed of O_2. This gas collection data suggests that approximately 20.4% of the anode current was directed toward the oxygen evolution reaction.

The apparent deficit between the total current and the sum of the currents accountable by ClO^- production (62.5%) and O_2 collection (20.4%) is approximately 17.1%. We attribute this primarily to the loss of Cl_2 via two pathways: (i) the reversibility of the

reaction $Cl_2 + 2OH^- \rightleftharpoons Cl^- + ClO^- + H_2O$, and (ii) direct volatilization of Cl_2 gas into the atmosphere, which was corroborated by a distinct pungent odor detected during experiments. Therefore, the total current fraction for Cl_2 generation (including the portion that later hydrolyzed to ClO^- and the portion that was lost) was approximately 79.6% (62.5% + 17.1%).

Table S8 The production rate of anode gas

	The 1st cycle	The 2nd cycle	The 3rd cycle	The 4th cycle	The 5th cycle
Yield (μL)	125	125	125	125	125
Time (s)	978	1052	947	1043	1035
Production rate (μL/min)	7.67	7.13	7.92	7.19	7.24

Due to experimental limitations, a detailed quantitative analysis of the gas collected from the anode via gas chromatography (GC) was not performed (chlorine is a corrosive gas that can damage the instrument). Based on the above analysis, we assign 79.6% of the current to the Cl_2 generation pathway (with a theoretical thermodynamic voltage of 1.36 V) and the remaining 20.4% to the O_2 generation pathway (theoretical voltage of 1.23 V). With an actual applied cell voltage of 2.53 V and an overall measured Faradaic efficiency of 97.06%, the total energy efficiency of the system is calculated to be 51.2% using the ratio of the weighted average theoretical voltage to the input voltage.

In contrast, we performed gas composition confirmation of the H_2 generated at the cathode with argon as the carrier gas, as shown in Figure S39. The repeated measurements for H_2 collection and the GC results confirm that the performance enhancement is robust and results from the proposed strategy in this work, rather than systematic or measurement errors.

The hydrogen production demonstration in this work primarily aims to validate the high performance of the proposed multi-stage buck strategy, and thus no further optimization of the electrochemical reaction itself was performed. The achieved hydrogen production rate of 16.5 mL/(min·m^2) further underscores the critical importance of efficient circuit management in TENG-based self-powered electrochemical systems.

We believe that future research could combine the circuit strategy proposed here with systematic reaction optimizations to further enhance hydrogen production performance. For instance, developing efficient catalysts and designing structured electrodes could increase active sites and thereby reduce the reaction overpotential. Alternatively, screening suitable electrolyte systems and optimizing their concentration ranges could lower the ohmic loss of the system. These measures are expected to decrease the output voltage and improve the output current of the TENG and the overall energy conversion efficiency of the system while maintaining a high Faradaic efficiency.

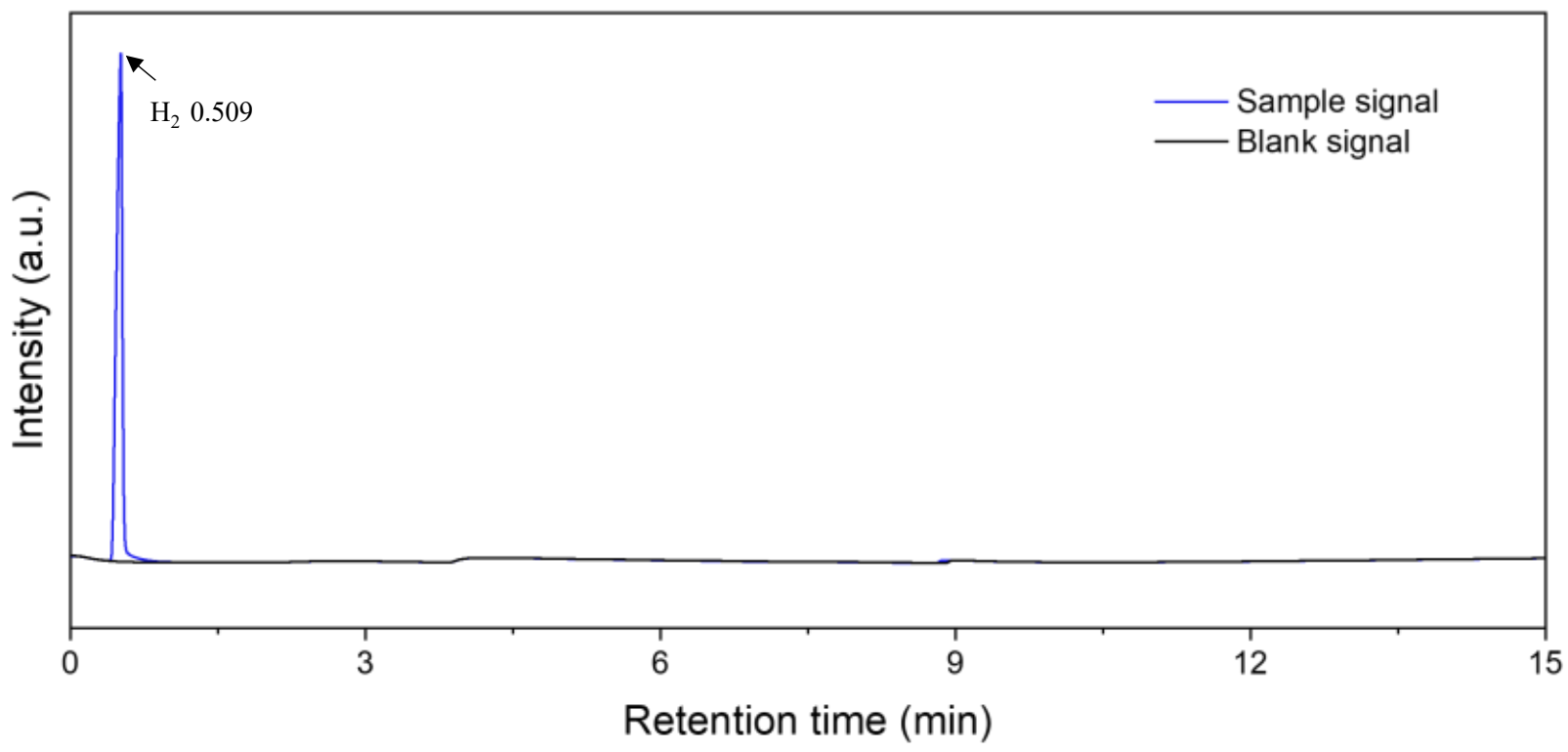

Figure S39. GC spectrum of H_2. Source data are provided as a Source Data file.

Table S9. The production rate of the hydrogen production system powered by TENG of different works.

	REF.	Methods	H_2 product rate (mL/(min·m^2))
Without PMC	45	/	0.4
	46	RuO_x/CNT	0.9
	47	Serpentine-connected array of electrolytic cells	1.8
With PMC	48	LTC3588-1	0.4
	49	Transformer	1.7
	50	Transformer	2.0
With PMC & Catalyst	51	LC circuit & MoS_2/Ti_3C_2	0.3
	52	Transformer & Photoelectric water splitting	0.4
	53	Transformer & Ti-Fe_2O_3	0.5
	54	Transformer & $WO_3/BiVO_4$	0.6
	55	Transformer & KOH	2.5
This work	/	Without secondary buck circuit	9.3
	/	With secondary buck circuit	16.5

Note S21 *Q-V* curve guided voltage selection for PMCs: experimental validation in sliding-mode devices

The key criterion for determining the applicability of the *Q*–*V* method is not the motion type of the TENG (e.g., rolling, rotary, contact-separation, or sliding), but whether its energy cycle exhibits collapse characteristics—specifically, whether surface charge density declines as voltage increases. In our previous work[1], we have demonstrated that sliding-mode DC-TENGs, AC-TENGs, and their structural variants all display behavior consistent with the energy collapse theory, validating that our design principle also applies to these devices. Furthermore, the theory related to selecting the operating voltage of the PMC based on the *Q*–*V* curve has been verified in Note S6.

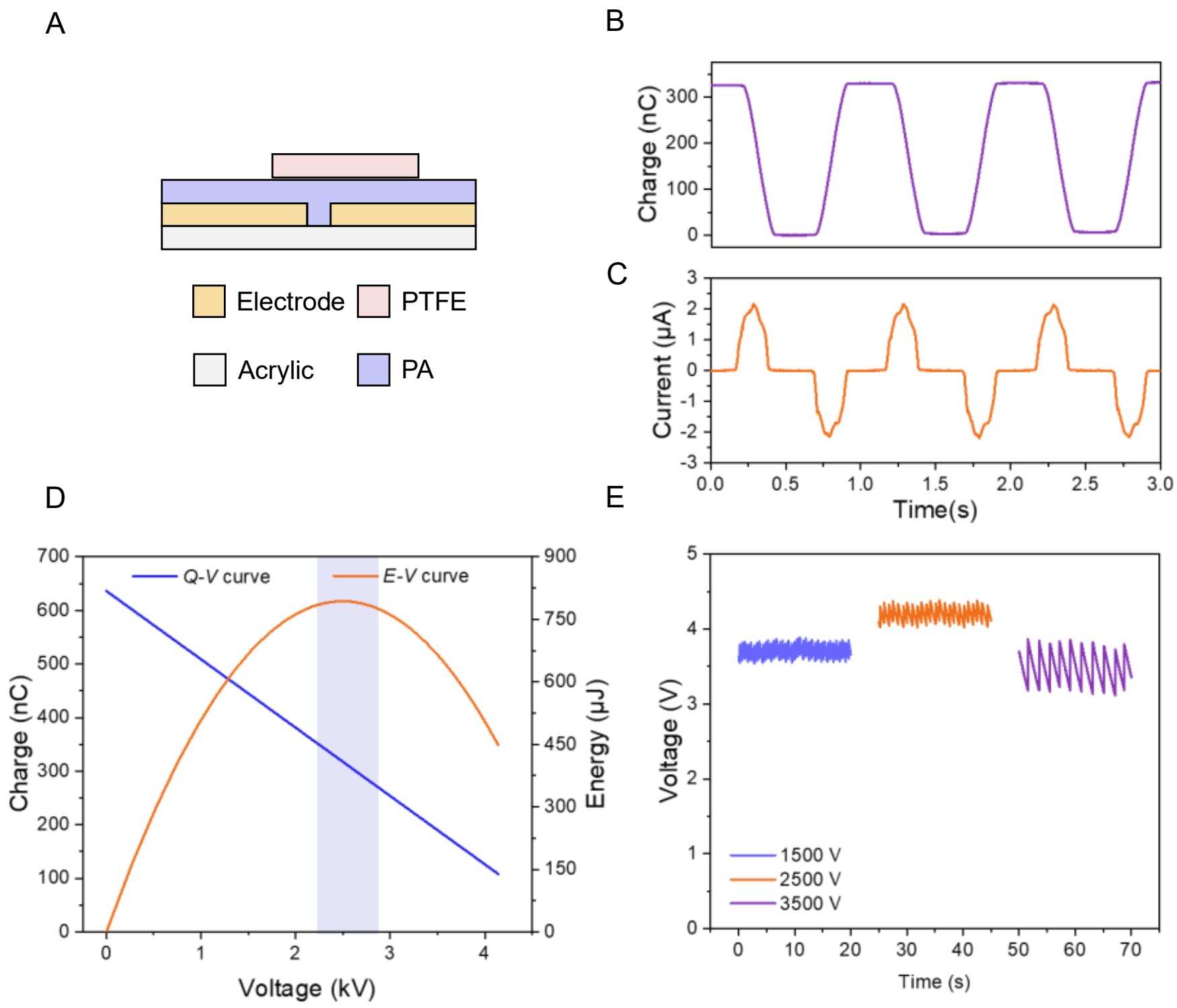

Figure S40 The performance of sliding-mode TENG. (A) The schematic structure of TENG. The effective area of the device is 20 cm^2, and the motion frequency is 1Hz; (B) The output charge; (C) The output current; (D) The Q-V curve; (E) The output voltage of TENG with PMC switching voltages set to 1500 V, 2500 V, and 3500 V. The resisitance is 0.1 MΩ. Source data are provided as a Source Data file.

Here, we also have supplemented the experimental results applying the *Q*–*V* curve to sliding-mode devices, which further support the conclusions above. Figure S40A illustrates the schematic structure of the sliding-mode TENG. Figures S40B and Figures

S40C present its output charge and output current curves, respectively. Figure S40D shows the corresponding *Q*–*V* characteristic curve. The test results indicate that the TENG achieves optimal output performance when the switching voltage of the primary circuit is set to 2500 V. Figure S40E compares the performance under the same load (0.1 MΩ) with switching voltages set to 1500 V, 2500 V, and 3500 V, demonstrating that the performance at 2500 V is the best, consistent with the *Q*–*V* curve results. This further confirms the rationale of using the *Q*–*V* curve to guide the design of the TENG PMC design.

Note S22 Comparison of circuit design and optimization of different works

Here, we conduct a comparison of these works cited in this work from five aspects: the TENG-PMC Matching Mechanism, TENG-PMC Matching Method, Circuit Architectures, Optimization Strategies, as well as performance, as shown in the Table S10. Regarding the TENG-PMC matching mechanism, previous studies have primarily relied on the *V-Q* plot obtained from the CMEO theory, which assumes the surface charge density is stable as the voltage increase. Consequently, such methods are only suitable for devices with lower performance or those in which air breakdown can be completely suppresses by circuit design. Their applicability to high-performance devices is questionable, and employing these existing circuits often leads to significant performance degradation (Table S1). In contrast, our work is based on the energy cycle collapse theory and utilizes the *Q-V* curve to guide the design of the circuit's operating voltage. This approach is broadly applicable to most high-performance devices, thereby achieving substantially higher power density and current density. In terms of circuit optimization, prior works have focused more on component-level improvements while fundamentally neglecting the issue of efficiency degradation under low voltage (capacitor, inductor, transformer, switch, diode etc.). Our study not only identifies and explains this critical problem but also provides a feasible solution by regulating the intermediate voltage of the secondary circuit, ensuring high efficiency even under low-voltage conditions.

Table S10 Comparison of circuit design and optimization of different works

<table>
<tr><td></td><td>Ref.12</td><td>Ref.19</td><td>Ref.24</td><td>Ref.11</td><td>Ref.17</td><td>Ref.23</td><td>Ref.13</td><td>Ref. 25</td><td>Ref. 10</td><td>This work</td></tr>
<tr><td>TENG-PMC Matching Mechanism</td><td colspan="9">The *V*-*Q* plot based on ideal CMEO theory</td><td>The *Q*-*V* curve based on energy cycle collapse theory</td></tr>
<tr><td>TENG-PMC Matching Method</td><td colspan="3">C_{in} extracts and stores energy</td><td colspan="6">TENG's inherent capacitor directly serves as the storage unit.</td><td>Regulation the circuit operating voltage based on *Q*-*V* curve</td></tr>
<tr><td>Circuit Architectures</td><td>LC</td><td>FSCC</td><td>LC</td><td>LC</td><td>Transformer coupled circuit</td><td>LC</td><td>LC</td><td>LC & Commercial chips</td><td>Charge-pump circuit & LC</td><td>Multi-Stage Buck Strategy</td></tr>
<tr><td>Optimization Strategies</td><td>Utilizes SCR to achieve passive switching design</td><td>Fractional circuit to reduce voltage drop of diode</td><td>Optimized GDT</td><td>Optimized inductor</td><td>Optimized transformer</td><td>Double loops design</td><td>Optimized capacitor; Half-wave circuit completely suppresses air breakdown</td><td>Reduced charge and voltage losses at switch; Achieved stable voltage output</td><td>Half-wave circuit completely suppresses air breakdown</td><td>Regulation the intermediate voltage of secondary circuit</td></tr>
<tr><td>Circuit efficiency</td><td>68.0% (~3.45V) 89.8% (Max Eff.)</td><td>62.5% (~3.45V) 94.5% (Max Eff.)</td><td>51.6% (~3.45V) 87.7% (Max Eff.)</td><td>45.0% (~3.45V) 81.6% (Max Eff.)</td><td>55.8% (~3.45V) 78.5% (Max Eff.)</td><td>37.8% (~3.45V) 90.6% (Max Eff.)</td><td>46.5% (~3.6V) 76.9% (Max Eff.)</td><td>N/A</td><td>40.5% (~3.45V) 52.9% (Max Eff.)</td><td>86.4% (3.69 V) 90.9% (Max Eff.)</td></tr>
<tr><td>Power density</td><td>0.10 W/m^2</td><td>0.04 W/m^2</td><td>0.08 W/m^2</td><td>0.09 W/m^2</td><td>0.29 W/m^2</td><td>0.07 W/m^2</td><td>0.28 W/m^2</td><td>0.63 W/m^2</td><td>0.15 W/m^2</td><td>10.35 W/m^2</td></tr>
<tr><td>Performance</td><td colspan="9">1. Most optimal circuit efficiencies exceed 80%; however, efficiency significantly decreases below 5V.
2. The power density of the TENG used does not exceed 1 W/m^2. Whether it is suitable for high-performance TENG (power density exceeding 10W/m^2) is a question.</td><td>1.Efficiency remains around 80% even at low voltage.
2.Power density exceeds 10 W/m^2
3.Current density reaches up to 5.19 A/m^2</td></tr>
</table>

Note S23 The Three-Stage Buck Circuit

The proposed two-stage design can indeed be extended to three or more stages. This becomes particularly necessary for applications requiring an extreme voltage conversion ratio (e.g., from tens of kilovolts down to a few volts)[23,24], because an input voltage at the ten-kilovolt level imposes extremely high rated-voltage requirements on the components in the primary circuit. Taking an LC circuit as an example, even though the voltage rating demand can be reduced by using series-connected freewheeling diodes, the substantially larger step-down ratio and the energy loss due to increased internal resistance would lead to a more pronounced circuit efficiency drop.

Therefore, for such ultra-high input voltages, a practical multi-stage approach could be:

First Stage: The FSCC could efficiently reduce the voltage from the ten-kilovolt level to the kilovolt level. This stage offers two key advantages: it relaxes the voltage rating requirements for subsequent components, and, since its output voltage is far greater than the forward voltage drops of its diodes, it can achieve a conversion efficiency exceeding 99%.

Second Stage: A robust LC resonant or transformer-coupled circuit could then step down the kilovolt-level voltage to an intermediate range (e.g., tens of volts).

Third Stage: Finally, a high-efficiency integrated buck converter (like the LTC3588) would provide precise, stable output at the target low voltage (e.g., 1.8V, 3.45V).

Beyond question, the adding stage is increased system complexity, encompassing control coordination and potential stability considerations. Therefore, while technically feasible, the decision to employ more than two stages should be driven by the specific voltage conversion ratio and power requirements.

Ultimately, our perspective is that optimizing TENG power management extends beyond circuit design alone; it critically depends on matching the output characteristics of the TENG with the input requirements of the PMC. A pursuit of extremely high TENG output voltage can create significant challenges for circuit design, while a pursuit of highly efficient circuit may not be compatible with existing high-performance TENGs.

Note S24 Narrow-Hysteresis Voltage Switches

A narrow-hysteresis characteristic switch stabilizes the circuit's operating voltage within a fixed range by limiting the voltage fluctuation range (Figure S41A). The characteristics of this switch can ensure that the TENG always operates within its optimal voltage range[25]. In contrast, a full-hysteresis characteristic switch exhibits periodic charging and discharging characteristics: the circuit's operating voltage rises from 0V to the rated value, then the switch conducts and resets to 0V (Figure S41B). The full-hysteresis characteristic switch is commonly observed in the spark gap switches, mechanical switches, and most semiconductor switches employed.

Taking the device in this research as an example, if a switch with narrow-hysteresis characteristics can be designed to operate at a threshold of 1500 V or 2500 V and extract energy from the TENG in a near-constant-voltage manner, the final output performance can theoretically be further enhanced. However, it should be noted that the maximum rated voltage of narrow-hysteresis switches reported in current literature only reaches the 500V, making it difficult to directly apply them to this system to overcome the energy bottleneck. Furthermore, this switch will not only enhance the input energy from the TENG to the circuit but also make it possible to increase the switching frequency and then to reduce the volume demand of energy storage components, thus achieving the goal of miniaturization.

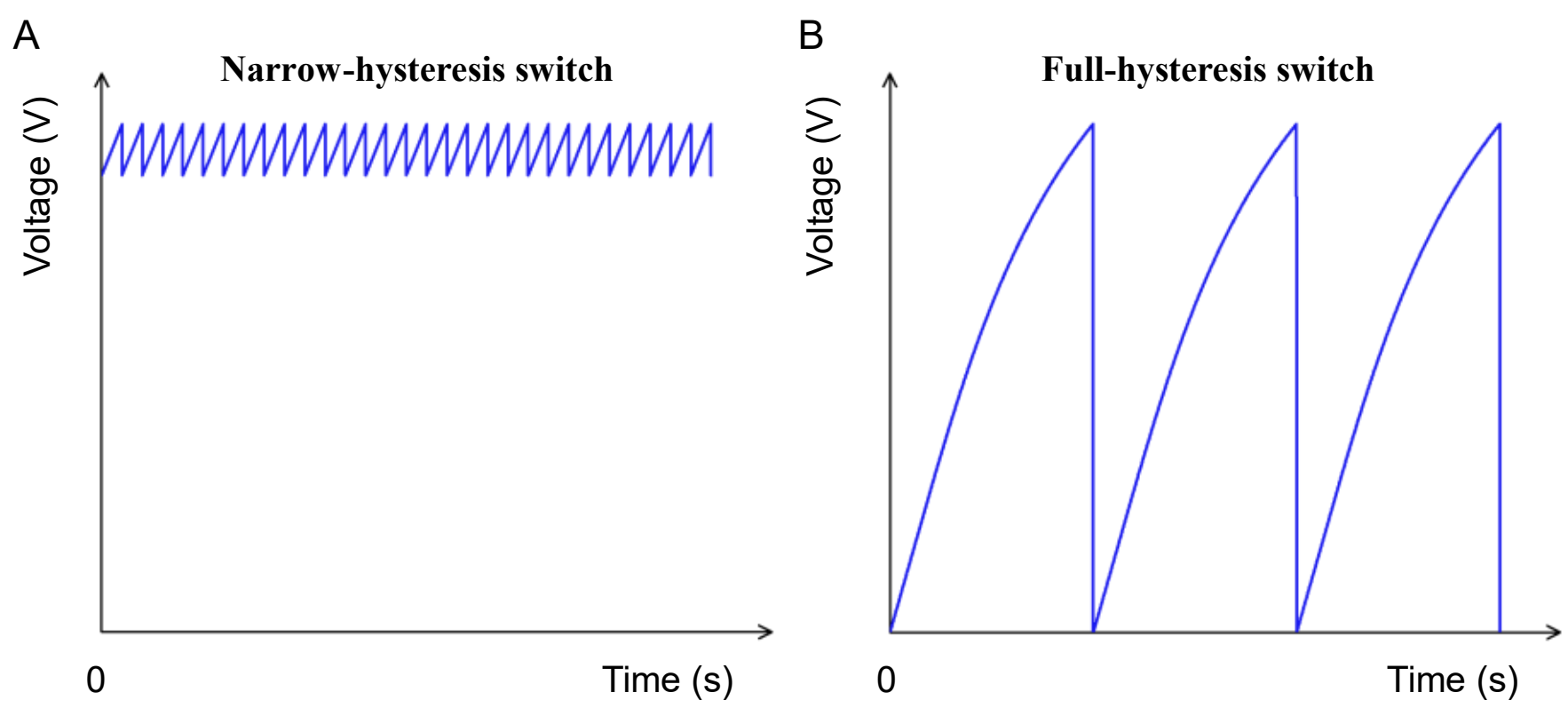

Figure S41. **The narrow-hysteresis switch and the full-hysteresis switch.** (A) The schematic diagram of output voltage of TENG with the narrow-hysteresis switch. (B) The schematic diagram of output voltage of TENG with the full-hysteresis switch.

Supplementary References

1. Gao Y*, et al.* Spontaneously established reverse electric field to enhance the performance of triboelectric nanogenerators via improving Coulombic efficiency. *Nature Communications* **15**, 4167 (2024).
2. Zi Y, Niu S, Wang J, Wen Z, Tang W, Wang ZL. Standards and figure-of-merits for quantifying the performance of triboelectric nanogenerators. *Nature Communications* **6**, 8376 (2015).
3. Gao Y*, et al.* Achieving high-efficiency triboelectric nanogenerators by suppressing the electrostatic breakdown effect. *Energy & Environmental Science* **16**, 2304-2315 (2023).
4. Cao Z*, et al.* Ultrahigh power generation from low-frequency and micro motion by suppressing air breakdown. *Energy & Environmental Science* **17**, 5002-5012 (2024).
5. Gao Y*, et al.* Achieving high performance triboelectric nanogenerators simultaneously with high-voltage and high-charge energy cycle. *Energy & Environmental Science* **17**, 8734-8744 (2024).
6. Wu H*, et al.* Efficient energy conversion mechanism and energy storage strategy for triboelectric nanogenerators. *Nature Communications* **15**, 6558 (2024).
7. Zou H*, et al.* Quantifying the triboelectric series. *Nature Communications* **10**, 1427 (2019).
8. Liu D*, et al.* Standardized measurement of dielectric materials' intrinsic triboelectric charge density through the suppression of air breakdown. *Nature Communications* **13**, 6019 (2022).
9. He L*, et al.* Dynamic interfacial electrostatic energy harvesting via a single wire. *Science Advances* **10**, eado5362 (2024).
10. Zhao Z*, et al.* Selection rules of triboelectric materials for direct-current triboelectric nanogenerator. *Nature Communications* **12**, 4686 (2021).
11. Cao LNY, Su E, Xu Z, Wang ZL. Fully enclosed microbeads structured TENG arrays for omnidirectional wind energy harvesting with a portable galloping oscillator. *Materials Today* **71**, 9-21 (2023).
12. Li X*, et al.* Three-dimensional chiral networks of triboelectric nanogenerators inspired by metamaterial's structure. *Energy & Environmental Science* **16**, 3040-3052 (2023).
13. Duan Y*, et al.* Scalable rolling-structured triboelectric nanogenerator with high power density for water wave energy harvesting toward marine environmental monitoring. *Nano Research* **16**, 11646-11652 (2023).
14. He L*, et al.* A high-output silk-based triboelectric nanogenerator with durability and humidity resistance. *Nano Energy* **108**, 108244 (2023).
15. Chen P*, et al.* Rationally segmented triboelectric nanogenerator with a constant direct-current output and low crest factor. *Energy & Environmental Science* **14**, 4523-4532 (2021).
16. Zhou H, Bhattacharya T, Tran D, Siew TST, Khambadkone AM. Composite Energy Storage System Involving Battery and Ultracapacitor With Dynamic Energy Management in Microgrid Applications. *IEEE Transactions on Power Electronics* **26**, 923-930 (2011).
17. Yang W, Wang Y, Li Y, Wang J, Cheng T, Wang ZL. Integrated flywheel and spiral spring triboelectric nanogenerator for improving energy harvesting of intermittent excitations/triggering. *Nano Energy* **66**, 104104 (2019).
18. Wang Y*, et al.* Gravity triboelectric nanogenerator for the steady harvesting of natural wind energy. *Nano Energy* **82**, 105740 (2021).
19. Xu H*, et al.* An actor–critic algorithm to maximize the power delivered from direct methanol fuel cells. *Nature Energy*, **10**, 951–961 (2025).

20. Liu W*, et al.* Switched-capacitor-convertors based on fractal design for output power management of triboelectric nanogenerator. *Nature Communications* **11**, 1883 (2020).
21. Wang Z*, et al.* Ultrahigh Electricity Generation from Low-Frequency Mechanical Energy by Efficient Energy Management. *Joule* **5**, 441-455 (2021).
22. Niu S, Wang X, Yi F, Zhou YS, Wang ZL. A universal self-charging system driven by random biomechanical energy for sustainable operation of mobile electronics. *Nature Communications* **6**, 8975 (2015).
23. Liu J*, et al.* Achieving Ultra-High Voltage (≈10 kV) Triboelectric Nanogenerators. *Advanced Energy Materials* **13**, 2300410 (2023).
24. Lei R*, et al.* Sustainable high-voltage source based on triboelectric nanogenerator with a charge accumulation strategy. *Energy & Environmental Science* **13**, 2178-2190 (2020).
25. Zhang H*, et al.* Employing a MEMS plasma switch for conditioning high-voltage kinetic energy harvesters. *Nature Communications* **11**, 3221 (2020).
