# Supplementary material for: Multi-Stage buck strategy for power management of triboelectric nanogenerators
Source: Nat Commun. 2026 Sep 29;17:11216. doi: 10.1038/s41467-026-78002-0 (PMC13720038; doi:10.1038/s41467-026-78002-0)
Supplement: Supplementary file 2 [file 41467_2026_78002_MOESM2_ESM.pdf]

nature portfolio

Peer Review File

Multi-Stage Buck Strategy for Power Management of Triboelectric Nanogenerators

Corresponding Author: Professor Jie Wang

This file contains all reviewer reports in order by version, followed by all author rebuttals in order by version.

Version 0:

Reviewer comments:

Reviewer #1

(Remarks to the Author)
The manuscript entitled “Multi-Stage Buck Strategy for Power Management of Triboelectric Nanogenerators” presents a comprehensive and methodically executed study that addresses a long-standing challenge in triboelectric nanogenerator (TENG) research — the substantial drop in power-management circuit efficiency at low voltages (below 10 V). The authors introduce a multi-stage buck configuration that strategically integrates a primary LC buck circuit triggered by a gas discharge tube (GDT) with a secondary commercial buck converter. The innovation lies in using the Q–V characteristic of the TENG to determine the optimal switching voltage, thereby coordinating internal node voltages (VN → VP → VQ) to maximize energy extraction. This approach is experimentally demonstrated to achieve approximately 80–86% efficiency at low output voltages, along with current densities up to 5.19 A m^{-2} and hydrogen-production rates reaching 16.8 mL min^{-1} m^{-2}. The work is well-motivated, scientifically sound in concept, and addresses a widely recognized bottleneck in the field of self-powered systems and triboelectric energy harvesting.

However, several aspects of the work require substantial clarification and additional experimental validation before it can be considered for publication in Nature Communications. First, while the claimed efficiency improvement is impressive, the study lacks a direct comparative benchmark against standard power-management circuits under identical operating conditions. The authors compare their circuit performance to values extracted from literature, but such comparisons are inherently affected by differences in device geometry, load impedance, and mechanical excitation. To substantiate the claimed superiority, the same TENG should be tested under identical mechanical input using three baseline configurations: (i) a transformer-coupled circuit, (ii) a switched-capacitor converter, and (iii) a commercial energy-harvesting IC such as the LTC3588-1. A quantitative comparison of conversion efficiency and output current at 1.8 V, 3.3 V, and 5.0 V would clearly establish the relative advantage of the proposed strategy.
Question: Can the authors perform direct, side-by-side measurements using these reference PMCs under identical excitation to confirm that the reported 80–86% efficiency gain arises from their multi-stage buck approach rather than from differences in test conditions?

Second, the electrolysis experiments demonstrating hydrogen generation are promising but presently lack the quantitative rigor expected in an energy research article. The manuscript reports a hydrogen-production rate of 16.8 mL min^{-1} m^{-2}, which is nearly an order of magnitude higher than previous reports, yet no details are provided on the Faradaic efficiency, gas composition, or stability of the process. Without these data, it is difficult to verify that the increased production rate originates from true electrochemical efficiency rather than differences in system configuration or measurement artifacts. The authors should conduct additional experiments to measure the H_2 : O_2 ratio, quantify Faradaic efficiency over several cycles, and evaluate performance stability over at least one hour of continuous operation.
Question: Could the authors provide Faradaic efficiency measurements, gas composition analysis (e.g., via GC or volumetric displacement), and long-term stability data to confirm that the enhanced hydrogen-production rate reflects genuine electrochemical efficiency?

Third, although the use of Q–V characteristics to guide circuit optimization is a conceptually elegant approach, the manuscript does not sufficiently delineate how this method differs from prior works that have employed Q–V-guided matching or multi-loop PMC strategies. Several studies from 2022–2024 have already explored dual-loop or charge-pump–buck combinations, though typically with efficiencies below 60% at 5 V. To strengthen the novelty claim, the authors should clarify whether their primary contribution lies in the system-level coordination of node voltages or in theoretical derivation of

the switching threshold from Q–V analysis. A figure or table contrasting their architecture with representative prior approaches would be particularly useful.
Question: How does the proposed “multi-stage buck strategy” fundamentally differ from previously reported dual-loop or charge-pump–buck power-management circuits, and can the authors provide a comparative schematic or performance table to clarify this distinction?

Fourth, while the authors demonstrate the applicability of their approach using both rolling-mode and rotary-mode TENGs, further evidence of universality and scalability would strengthen the impact of the work. Since contact-separation and lateral-sliding TENGs dominate many practical implementations, validating the approach on one additional TENG configuration—or at least discussing its theoretical applicability—would support the claim that the method provides a generalized solution for TENG power management.
Question: Could the authors extend their study to another TENG architecture or provide simulations showing that the same Q–V-based optimization principle holds for contact-separation or lateral-sliding modes?

Fifth, given that the primary buck stage operates at approximately 1.5 kV, details regarding insulation, circuit safety, and dielectric breakdown prevention are essential. The supplementary information briefly mentions polyimide insulation, but more quantitative data on leakage current, dielectric strength, and environmental stability (humidity and temperature dependence) should be presented to ensure reproducibility and safety of the high-voltage design.
Question: What specific insulation materials, spacing, and design standards were used to prevent dielectric breakdown in the 1.5 kV primary circuit, and how do these parameters affect long-term reliability?

Finally, the data presentation can be improved for quantitative clarity. Efficiency values should include error bars or statistical variations from repeated measurements, as well as explicit uncertainty sources. The manuscript would also benefit from a concise summary table comparing the proposed strategy with existing PMCs (including LC, transformer, SCC, and commercial ICs) at standard output voltages. This would make the performance comparison transparent and accessible for readers.
Question: Can the authors include statistical analysis of efficiency data and a summary table comparing their method with state-of-the-art PMCs under standard output voltages (1.8 V, 3.3 V, and 5 V)?

In conclusion, the manuscript presents a technically interesting approach that could contribute to the advancement of efficient power management for TENGs. However, the scientific foundation and comparative validation are not yet sufficient to justify publication in Nature Communications. The concept is incremental rather than revolutionary in circuit design, and the practical gains, while notable, need stronger experimental proof and clearer theoretical grounding. If the authors can deliver robust benchmarking, quantitative electrochemical data, and improved mechanistic analysis, the paper could become a valuable contribution to the field. At its current stage, however, it remains primarily an engineering optimization with insufficient depth of scientific validation for this journal.

Reviewer #2

(Remarks to the Author)
The manuscript entailed, "Multi-Stage Buck Strategy for Power Management of Triboelectric Nanogenerators" is presents multistage buck strategy for Triboelectric nanogenerators (TENGs) that synergistically combines dual-stage voltage conversion circuit with TENG’s output characteristic by the Q-V curve, enabling precise voltage regulation at critical nodes for performance improvement. As per the demonstrated results, the proposed strategy achieves approximately 80% circuit efficiency at the low-voltage range while delivering 3.85 A/m2 current density at 2.5 V. The work is worthy to publish in this journal.

Reviewer #3

(Remarks to the Author)
The paper addresses a significant limitation in TENG power management and presents a technically robust solution. However, before publication, the authors should provide:

1. The claimed novelty over existing transformer-based and switched-capacitor TENG PMCs (Refs. 11–19) should be made more explicit. Please elaborate how the “multi-stage buck” differs in operational principle, not only in topology but also in efficiency optimization mechanism.

2. The manuscript frequently cites “one order of magnitude improvement” but lacks a consistent baseline definition. Clarify which benchmark (voltage, power density, or current density) is used for this claim.

3. The use of the Q–V curve to determine optimal GDT threshold voltage (around 1.5 kV) is conceptually strong, but should include mathematical reasoning or modeling showing how energy extraction efficiency varies with switch threshold.

4. Equations (1)–(10) should be supported with circuit simulation validation (LT-SPICE waveforms, efficiency plots). Some terms such as $Imax$, Pin, and $Rint$ are not clearly defined with units and measured values.

5. The reported 80–90% circuit efficiency is remarkably high for TENG-driven systems. Please provide calibration details, measurement uncertainties, and comparison under identical load conditions with other PMCs.

6. The demonstration powering 16 sensors and a mobile phone is compelling, but quantitative stability (voltage ripple, current stability) and endurance under continuous operation should be discussed.

7. The hydrogen production rate of 16.8 mL/(min·m^2) should be compared against theoretical Faradaic limits to assess electrochemical efficiency. Include more context on catalyst material, electrode configuration, and electrolyte concentration. Also, it would strengthen the study to present the energy conversion efficiency of the electrolysis system.

8. Figures 1–5 contain excessive sub-panels and small text, making them difficult to interpret. Simplify or enlarge critical plots, especially efficiency vs. voltage comparisons.

9. Figure 3J ("Normalized efficiency of different works") requires a clearer explanation of the normalization criteria and error margins.

10. Clarify whether "multi-stage buck" can be extended to >2 stages and discuss potential trade-offs (complexity vs. gain).

11. The Methods section should specify model numbers and tolerances of GDTs, diodes, and inductors used.

12. Add a brief limitations statement in the Discussion to indicate scalability and reliability challenges for industrial adaptation.

Reviewer #4

(Remarks to the Author)
In the reviewed manuscript, the authors have introduced an innovative method for power control in Triboelectric Nanogenerators, termed the multi-stage buck strategy. The effectiveness of the proposed method has been successfully tested and validated on various TENGs.

The experiments, the obtained results, and the presentation are highly successful. This work makes a significant and original contribution to the literature.

I congratulate the authors for their valuable work. I recommend that the article be accepted for publication.

Reviewer #5

(Remarks to the Author)
This study proposes a multi-stage buck strategy that synergistically combines a dual-stage voltage conversion circuit with the TENG's output characteristics based on the Q–V curve, enabling precise voltage regulation at critical nodes. I recommend major revision.

1. The authors should provide a more detailed discussion of the state-of-the-art to better support the significance of this work in introduction. To improve the efficiency of PMC for TENGs, what kinds of technologies have been utilized previously? Please include representative studies, their main contributions, and their limitations. Additionally, clarify whether similar work has been reported by other research groups, and clearly state how this work is unique.

2. Please include more detailed information in Table S2 such as input and output power, operating frequency of the TENG, and the circuit topology used.

3. Provide a complete list of major components, including the diodes, inductors (for both the first and second stages of converter), and MOSFETs

4. Include the power losses of major components (diodes, inductors in the first and second stages, and MOSFETs) to support the claimed efficiency.
5. The description of circuit efficiency is unclear in abstract. Please provide more specific details (e.g., the efficiencies of the first and second buck converter stages).

Version 1:

Reviewer comments:

Reviewer #3

(Remarks to the Author)
The authors are required to address the following critical points for further consideration:

The efficiency claims (80–86% at low voltage) require clearer definition of measurement boundaries, calibration method, and uncertainty analysis; without this, the reported improvement cannot be independently validated.

The central theoretical premise—the 50% Coulombic efficiency condition derived from a linear σ–V decay model—needs stronger experimental verification across the full operating range to confirm general validity.

The novelty relative to recent dual-loop and charge-pump–buck PMC strategies is not sufficiently differentiated; a concise comparative schematic and performance table against 2022–2024 literature is necessary.

The hydrogen-production validation lacks independent gas-composition confirmation (e.g., GC analysis), which weakens the strength of the electrochemical performance claims.

Long-term operational stability and scalability toward sustained higher power density operation are insufficiently demonstrated for practical deployment relevance.

The impact of parasitic elements, switching losses, and thermal behavior in the high-voltage primary stage is not quantitatively analyzed, which may affect real-world efficiency.

Reviewer #6

(Remarks to the Author)
The authors have made substantive revision to the manuscript and addressed the major concerns raised by the reviewers. In particular, the authors now include a detailed benchmarking against other power-management configurations, which substantiates their claim that the proposed multi-stage buck strategy offers a low-voltage performance advantage over these other configurations. Further, the inclusion of repeated efficiency measurements with statistical variation, additional validation on a sliding-mode TENG, and expanded analysis of energy extraction efficiency has improved the rigor, scope, and reproducibility of the work. Overall, I consider the present version suitable for publication.

Version 2:

Reviewer comments:

Reviewer #3

(Remarks to the Author)

Open Access This Peer Review File is licensed under a Creative Commons Attribution 4.0 International License, which permits use, sharing, adaptation, distribution and reproduction in any medium or format, as long as you give appropriate credit to the original author(s) and the source, provide a link to the Creative Commons license, and indicate if changes were made.
In cases where reviewers are anonymous, credit should be given to 'Anonymous Referee' and the source.
The images or other third party material in this Peer Review File are included in the article’s Creative Commons license, unless indicated otherwise in a credit line to the material. If material is not included in the article’s Creative Commons license and your intended use is not permitted by statutory regulation or exceeds the permitted use, you will need to obtain

permission directly from the copyright holder.
To view a copy of this license, visit https://creativecommons.org/licenses/by/4.0/

Point-by-point responses to the reviewers' comments

We (the authors) would like to thank the editor and reviewers for dedicating their valuable time and expertise to assess the manuscript. We appreciate the reviewers' thorough and insightful comments, which significantly contributed to enhancing the quality of the present work. In the following we elucidate our response to each remark, along with the corresponding revisions made in the manuscript, in our pursuit of continued consideration for this submission. (**Comments in Black, responses in Blue, revisions in Red**)

REVIEWER COMMENTS

Response to Reviewer #1

The manuscript entitled "Multi-Stage Buck Strategy for Power Management of Triboelectric Nanogenerators" presents a comprehensive and methodically executed study that addresses a long-standing challenge in triboelectric nanogenerator (TENG) research — the substantial drop in power-management circuit efficiency at low voltages (below 10 V). The authors introduce a multi-stage buck configuration that strategically integrates a primary LC buck circuit triggered by a gas discharge tube (GDT) with a secondary commercial buck converter. The innovation lies in using the Q–V characteristic of the TENG to determine the optimal switching voltage, thereby coordinating internal node voltages ($V_N \rightarrow V_P \rightarrow V_Q$) to maximize energy extraction. This approach is experimentally demonstrated to achieve approximately 80–86% efficiency at low output voltages, along with current densities up to 5.19 A m^{-2} and hydrogen-production rates reaching 16.5 mL/(min·m^2). The work is well-motivated, scientifically sound in concept, and addresses a widely recognized bottleneck in the field of self-powered systems and triboelectric energy harvesting.

Response: We highly appreciate the reviewer for carefully reviewing our work, and thank your positive comments on our research work, which has encouraged us greatly.

Comment 1

However, several aspects of the work require substantial clarification and additional experimental validation before it can be considered for publication in ***Nature Communications***. First, while the claimed efficiency improvement is impressive, the study lacks a direct comparative benchmark against standard power-management circuits under identical operating conditions. The authors compare their circuit performance to values extracted from literature, but such comparisons are inherently affected by differences in device geometry, load impedance, and mechanical excitation. To substantiate the claimed superiority, the same TENG should be tested under identical mechanical input using three baseline configurations: (i) a transformer-coupled circuit, (ii) a switched-capacitor converter, and (iii) a commercial energy-harvesting IC such as the LTC3588-1. A quantitative comparison of conversion efficiency and output current at 1.8 V, 3.3 V, and 5.0 V would clearly establish the relative advantage of the proposed strategy.

Question: Can the authors perform direct, side-by-side measurements using these reference PMCs under

identical excitation to confirm that the reported 80–86% efficiency gain arises from their multi-stage buck approach rather than from differences in test conditions?

Response: We sincerely thank the reviewer for the careful evaluation and constructive suggestions.

The circuit efficiency at low voltages reported in this work is achieved by introducing a secondary circuit to regulate the output voltage of the primary circuit. This approach reduces the energy loss caused by the diode forward voltage drop and internal resistance in the primary circuit.

Moreover, when comparing efficiencies across different studies, to avoid misleading comparisons of absolute efficiency values due to variations in testing and calculation methods, we adopted a normalized efficiency: taking the best efficiency reported in each paper as the 100% baseline and examining how efficiency declines as the output voltage decreases (the discussion of normalized efficiency is shown in **Note S16**). This method focuses on revealing the inherent "efficiency reduction at low voltage " issue in various PMC topologies. **It thereby highlights the one of our work's core objectives: to address the common challenge of sharply dropping efficiency in TENG power management under low-voltage applications.**

Here, from a theoretical perspective, we first analyze the fundamental challenges that various PMCs face when powering low-voltage loads.

1. Switched-Capacitor Circuits (SCC)

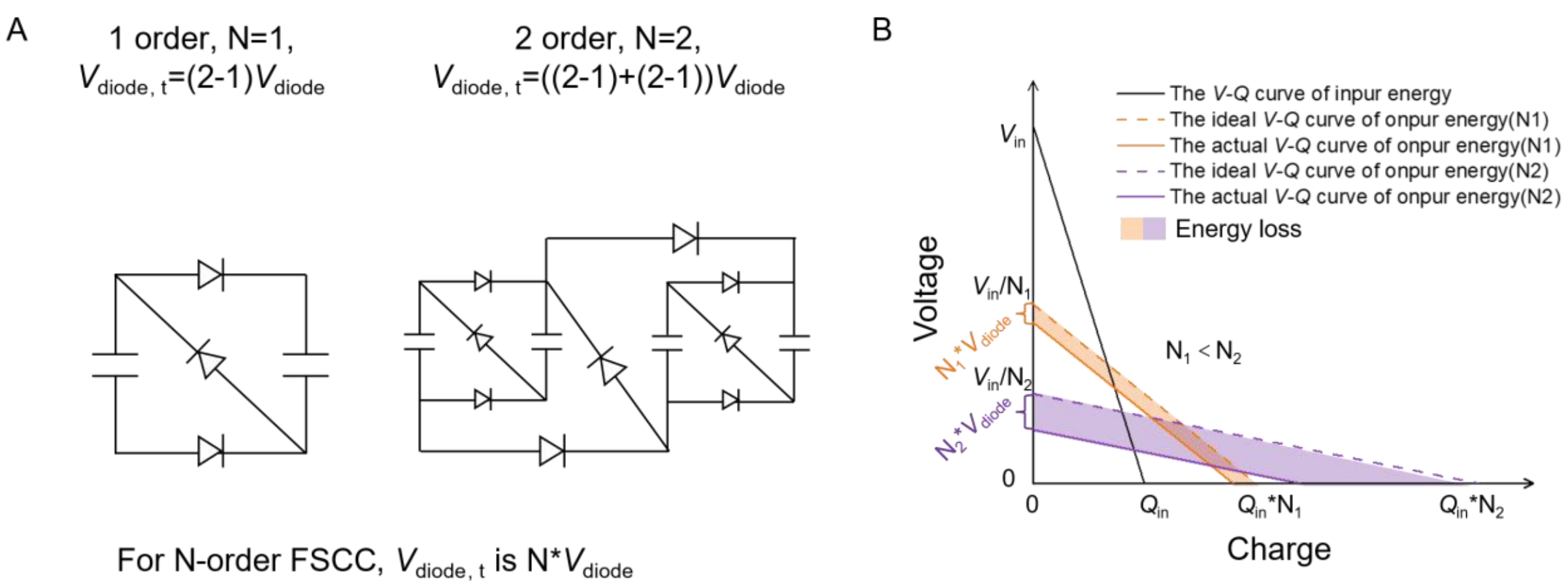

Figure R1 (Revised Figure S17). Fractal switched-capacitor converter. (A) Equivalent circuit diagrams (1-order FSCC and 2-order FSCC). (B) *V–Q* plots of input and output energy for the FSCC. The area enclosed by the black curve and the origin represents the input energy. The area enclosed by the orange dashed line and the origin corresponds to the theoretical output energy of the N_1-order FSCC. The area enclosed by the solid orange line and the origin denotes the actual output energy. The orange shaded region indicates the energy loss in practice due to N_1*V_{diode}. Similarly, the area enclosed by the purple dashed line and the origin represents the theoretical output energy of the N_2-order FSCC, while the area enclosed by the solid purple line and the origin shows the actual output energy. The purple shaded area corresponds to the practical energy loss caused by N_2*V_{diode}.

Taking the fractal switched-capacitor converter (FSCC) proposed by Liu et al[1]. as an example, its fractal conversion structure effectively mitigates the diode voltage drop (V_{diode}) problem in conventional SCC (**Figure R1A**), and the total V_{diode} ($V_{\text{diode, t}}$) is N*V_{diode} (N is the order of FSCC). Ideally (without the V_{diode}), the input energy (the black line in **Figure R1B**) with an input voltage (V_{in}) and charge (Q_{in}), after FSCC the output voltage reduces to $V_{\text{in}}/2^N$ and the charge increases to 2^N*Q_{in} (the orange dashed line and purple dashed line in **Figure R1B**). However, due to the actual V_{diode}, the practical output performance is illustrated in **Figure R1B** (the orange line and purple line): the output voltage decreases to $V_{\text{in}}/2^N$-N*V_{diode}, and the charge decreases proportionally. This leads to two main issues:

- To match a lower target output voltage, the order of FSCC must be increased. However, this raises the total voltage drop, resulting in lower efficiency. As the output voltage decreases, the proportion of the $V_{\text{diode, t}}$ increases, leading to greater energy loss (the shadow area in **Figure R1B**).
- If the order of FSCC is kept constant, although higher circuit efficiency can be maintained, the output may not align well with the target voltage. Consequently, the output energy under low-voltage conditions falls short of the circuit's optimal performance.

2. **Transformer-Coupled Circuits**

The efficiency of this circuits is primarily constrained by the following aspects:

- For TENGs that inherently output high-frequency signals, with a fixed transformer turns ratio, variations in output voltage can easily cause the primary coil voltage to deviate from the optimal operating voltage. This leads to impedance mismatch with TENG, significantly reducing the energy harvested from the device. Even if the output voltage is matched to the target low voltage through turns ratio adjustment, the voltage drop in subsequent rectification stages remains significant, affecting overall efficiency.
- For devices with low-frequency output signals, a spark switch is often used to increase the frequency[2]. Although impedance matching with the TENG output can be achieved via the transformer design, rectification or freewheeling operations are still required after the secondary coil. This process is similar to the secondary stage of LC circuit model and also introduces considerable energy loss under low-voltage conditions (**Figure R2**).

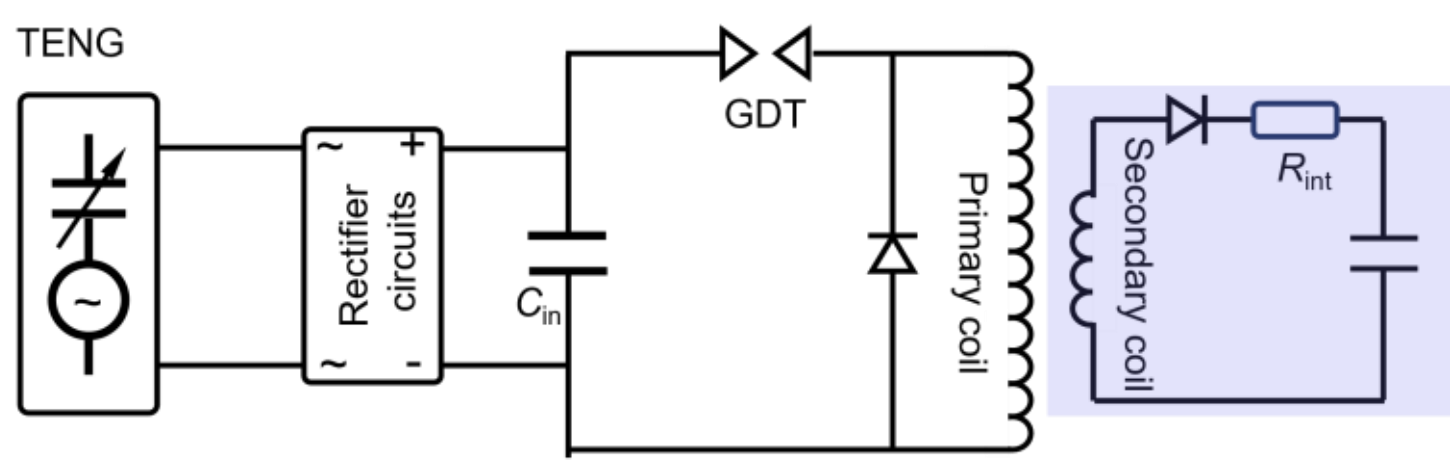

Figure R2 (Revised Figure S18). Equivalent circuit diagrams of transformer coupled circuits. The shaded area represents the secondary coil circuit, whose structure is similar to the second stage in the LC circuit (**Figure S10B**). Consequently, it is also subject to energy loss due to diode voltage drop and circuit internal resistance.

3. **Commercial Energy Harvesting Chips (e.g., LTC3588)**

   While such chips exhibit high internal conversion efficiency (>90%) within their specified input range, their relatively low input voltage requirement is severely mismatched with the high-voltage (often hundreds of volts) and high-impedance characteristics typical of TENGs. Direct connection results in most of the energy failing to be effectively accepted by the chip input, leading to poor output performance.

Then, we conducted a performance comparison among the LC circuit, transformer-based circuit, commercial chips, and the multi-stage buck circuit proposed in this work.

Since the theoretical model of FSCC is well-established and sufficiently demonstrates its inherent issue of efficiency decline under low voltages, while its mechanical switches have limited applicability and are unsuitable for the device presented in this work—coupled with the high complexity (e.g., a 7-order FSCC requires 128 capacitors and 381 diodes)—it was not included in the experimental comparison.

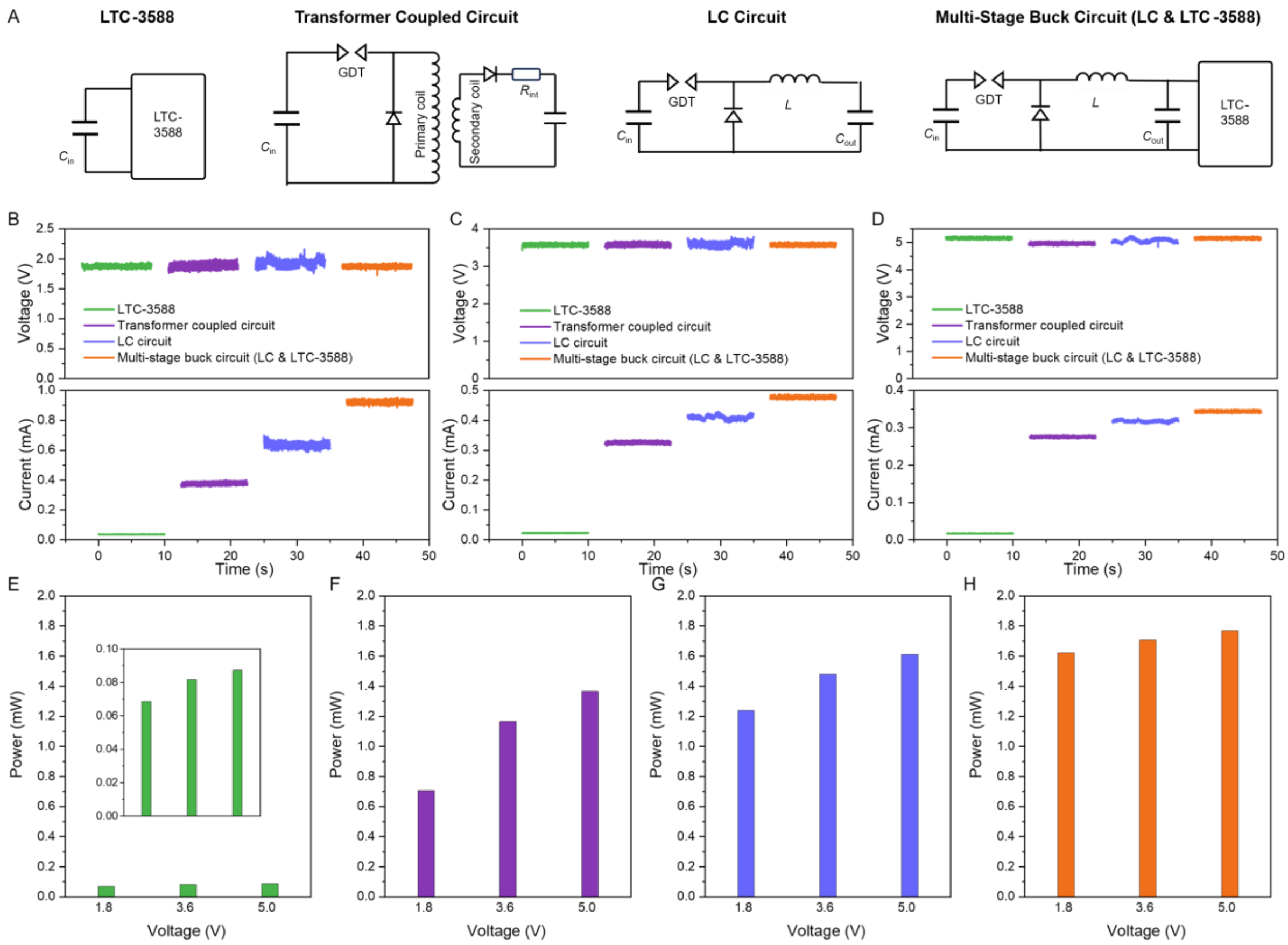

Figure R3 (**Added Figure S19**). **Performance comparison among the LTC3588, transformer coupled circuit, LC circuit, and multi-stage buck circuit.** (A) Equivalent circuit diagrams. (B)–(D) Output voltage and current. The multi-stage buck circuit delivers the highest output current at output voltages of approximately 1.8 V, 3.6 V, and 5.0 V, respectively. (E)–(H) Output power. The LTC3588 exhibits lower output power due to its mismatch with the TENG's output characteristics. Moreover, both the LC circuit and the transformer coupled circuit show a decline in output power at lower output voltages.

The experimental results are shown in the **Figure R3**. Under the same output voltage conditions (approximately

1.8 V, 3.6 V, and 5.0 V), the output current of the multi-stage buck circuit consistently exceeded that of the other three circuit (**Figure R3B-D**). Both the transformer and LC circuits exhibited a decrease in output power as the output voltage decreased (**Figure R3E-H**). This further confirms that the performance improvement reported in this work is not due to differences in testing conditions, but addresses the common challenge of sharply dropping efficiency in TENG power management under low-voltage applications.

More importantly, after replacing the primary circuit with a transformer coupled circuit and applying the multi-stage buck strategy, the output current at 1.8 V also increased significantly (**Figure R4**). This further demonstrates the broad applicability of the proposed strategy across different primary circuit topologies.

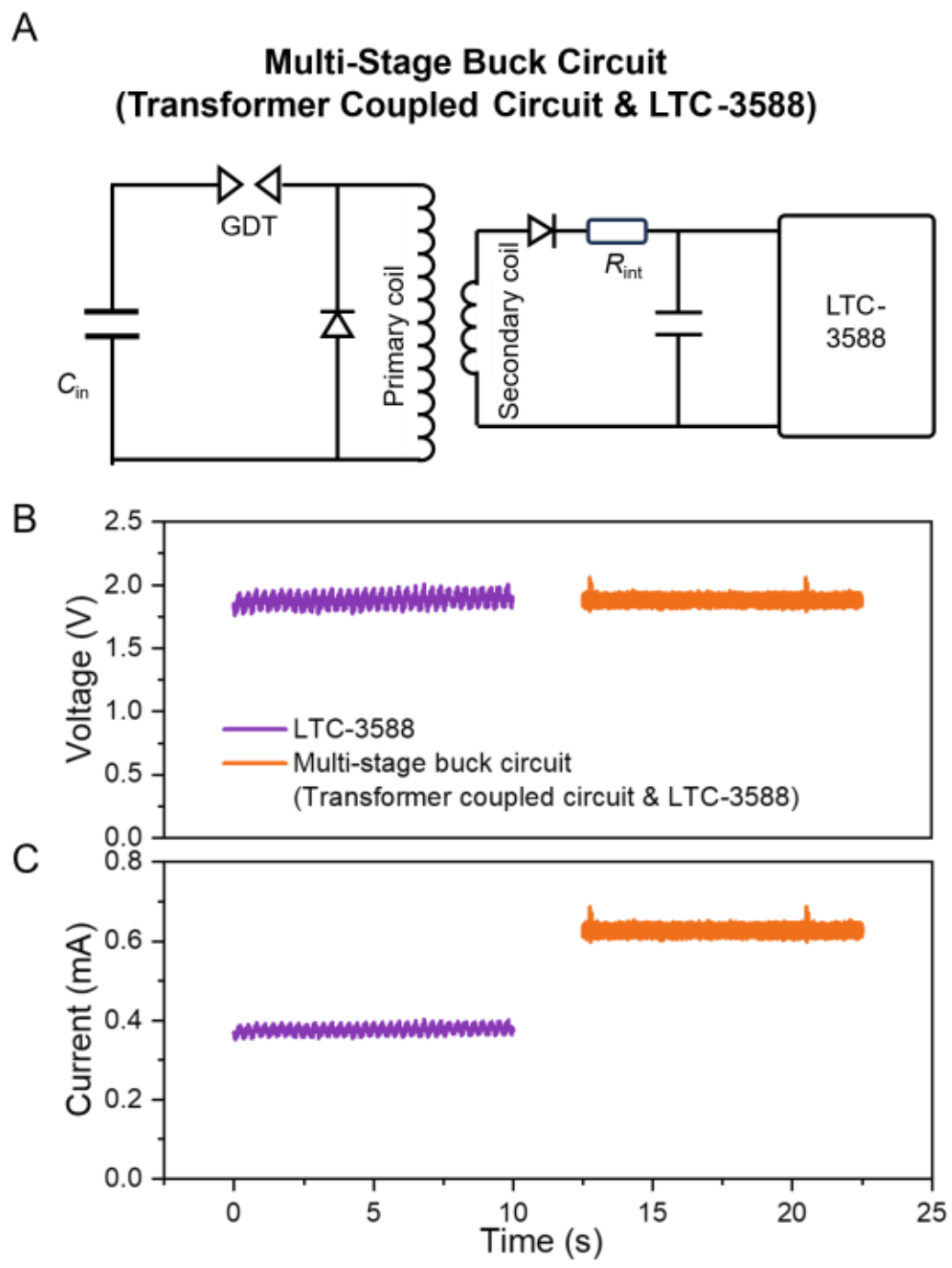

Figure R4 (Added Figure S20). Multi-stage buck circuit based on transformer coupled circuit. (A) Equivalent circuit diagram. (B) Output voltage. (C) Output current. At an output voltage of 1.8 V, the multi-stage buck circuit achieves an approximately 60% increase in output current compared to the transformer coupled circuit.

Revisions

The revised part in the present manuscript is as follows:

We have made a revision of "In addition, for SCC circuit and transformer coupled circuit, the energy loss caused by the diode and R_{int} is also the major reason for their low circuit efficiency at lower output voltages. This has been theoretically demonstrated and experimentally verified in **Note S14** and **Figure S17-19**." in the last paragraph "**The reasons for low efficiency in primary circuits**".

We have made a revision of "Comparison results robustly demonstrate that, compared to conventional approaches, our strategy can maintain high performance more effectively across a wide voltage range, especially

at low voltages, with a significantly smaller degree of performance decay. More importantly, after replacing the LC circuit with a transformer circuit and applying the multi-stage buck strategy, the output performance also increased significantly (**Note 14** and **Figure S20**). This further demonstrates the broad applicability of the proposed strategy across different primary circuit topologies." in the last paragraph "**The reasons for low efficiency in primary circuits**".

We have made a revision of "By restructuring the single-stage step-down path into a multi-stage path via the secondary buck circuit, efficiency improves significantly, reaching approximately 80% in low-voltage ranges. The strategy demonstrates broad applicability, having been successfully implemented in LC and transformer-coupled circuits (**Figure S20** and **Note S14**)." in the last paragraph "**Discussions**".

The revised part in the Supplementary Information is as follows:

We have added the above discussion into the "**Note S14 Efficiency limitations of existing power management circuits for TENGs**" in current Supplementary Information.

We have added the "**Table S1 Performance of different high-performance TENGs**" in current Supplementary Information.

We have revised the **"Figure S17. Fractal switched-capacitor converter."** and **"Figure S18. Equivalent circuit diagrams of transformer coupled circuits"** in current Supplementary Information.

We have added the "**Figure S19. Performance comparison among the LTC3588, transformer coupled circuit, LC circuit, and multi-stage buck circuit**" and "**Figure S20. Multi-stage buck circuit based on transformer coupled circuit"** in current Supplementary Information.

Comment 2

Second, the electrolysis experiments demonstrating hydrogen generation are promising but presently lack the quantitative rigor expected in an energy research article. The manuscript reports a hydrogen-production rate of 16.5 mL/(min·m^2), which is nearly an order of magnitude higher than previous reports, yet no details are provided on the Faradaic efficiency, gas composition, or stability of the process. Without these data, it is difficult to verify that the increased production rate originates from true electrochemical efficiency rather than differences in system configuration or measurement artifacts. The authors should conduct additional experiments to measure the H_2 : O_2 ratio, quantify Faradaic efficiency over several cycles, and evaluate performance stability over at least one hour of continuous operation.

Question: Could the authors provide Faradaic efficiency measurements, gas composition analysis (e.g., via GC or volumetric displacement), and long-term stability data to confirm that the enhanced hydrogen-production rate reflects genuine electrochemical efficiency?

Response: We sincerely thank the reviewer for the careful evaluation and constructive suggestions.

In this work, hydrogen production was measured using the volumetric displacement method. The anode is a ruthenium iridium titanium electrode, and the cathode is a graphite electrode. The concentration of the NaCl solution used is 1 mol/L. No catalysts were used in this work. The time was recorded at every 25 μL increment based on the graduated cylinder scale, with 100 μL constituting one measurement cycle. Five independent tests were performed. The results are summarized in the **Table R1** below.

Table R1 (Added Table S6) The production rate of hydrogen and faradaic efficiency

	The 1st cycle					The 2nd cycle					The 3rd cycle				
Time (s)	0	18	39	64	86	0	25	46	75	84	0	21	42	68	84
Yield (μL)	0	25	50	75	100	0	25	50	75	100	0	25	50	75	100
Production rate (μL/min)	69.77					71.4					71.4				
Faradaic efficiency	98.46%					96.27%					95.29%				

	The 4th cycle					The 5th cycle				
Time (s)	0	17	35	55	80	0	22	43	60	81
Yield (μL)	0	25	50	75	100	0	25	50	75	100
Production rate (μL/min)	75.00					74.07				
Faradaic efficiency	98.08%					97.63%				

The average hydrogen production rate was determined to be 72.33 μL min^{-1} (**SATP**, 2.92 **μmol/min**), which corresponds to 16.5 mL/(min·m^2) (calculated by the effective working area of the TENG). The average faraday efficiency measured is 97.15%. The corresponding current data are presented in the **Figure R5**. **These results sufficiently demonstrate that the significant performance improvement reported in this work is not attributable to differences in system configuration or measurement artifacts.**

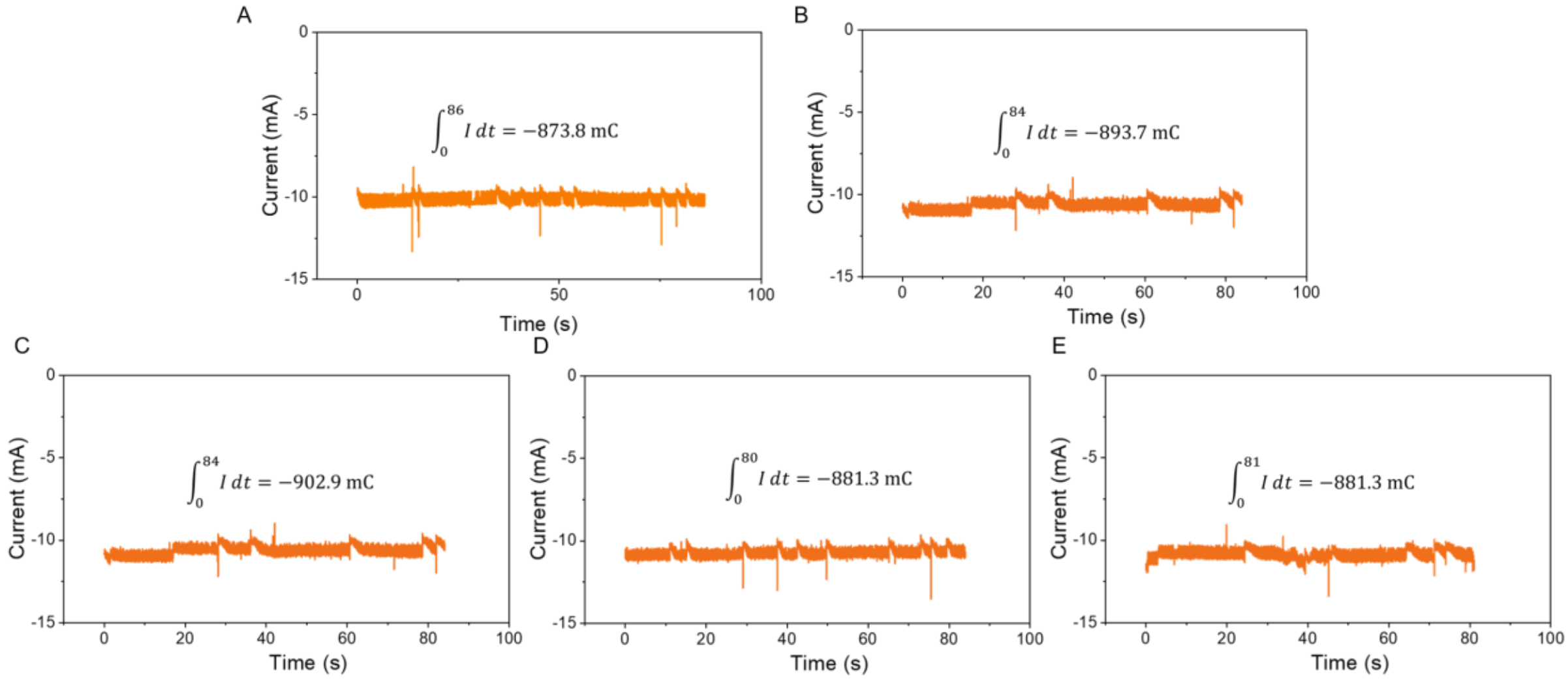

Figure R5. The operating current of the self-power hydrogen production system. (A)-(E) Test current from the first to the fifth time.

Furthermore, the TENG serves solely as a power source to supply voltage and current for the electrochemical reaction. Therefore, provided that both the TENG and the circuit operate stably, the final hydrogen output maintain stability. The stability of the TENG and the circuit is shown **Figure R6**. Additionally, stability tests were performed by measuring the time required to produce 250 μL of hydrogen, comparing the initial measurement with that taken after one hour (**Table R2**). The results confirm a stable production rate over time.

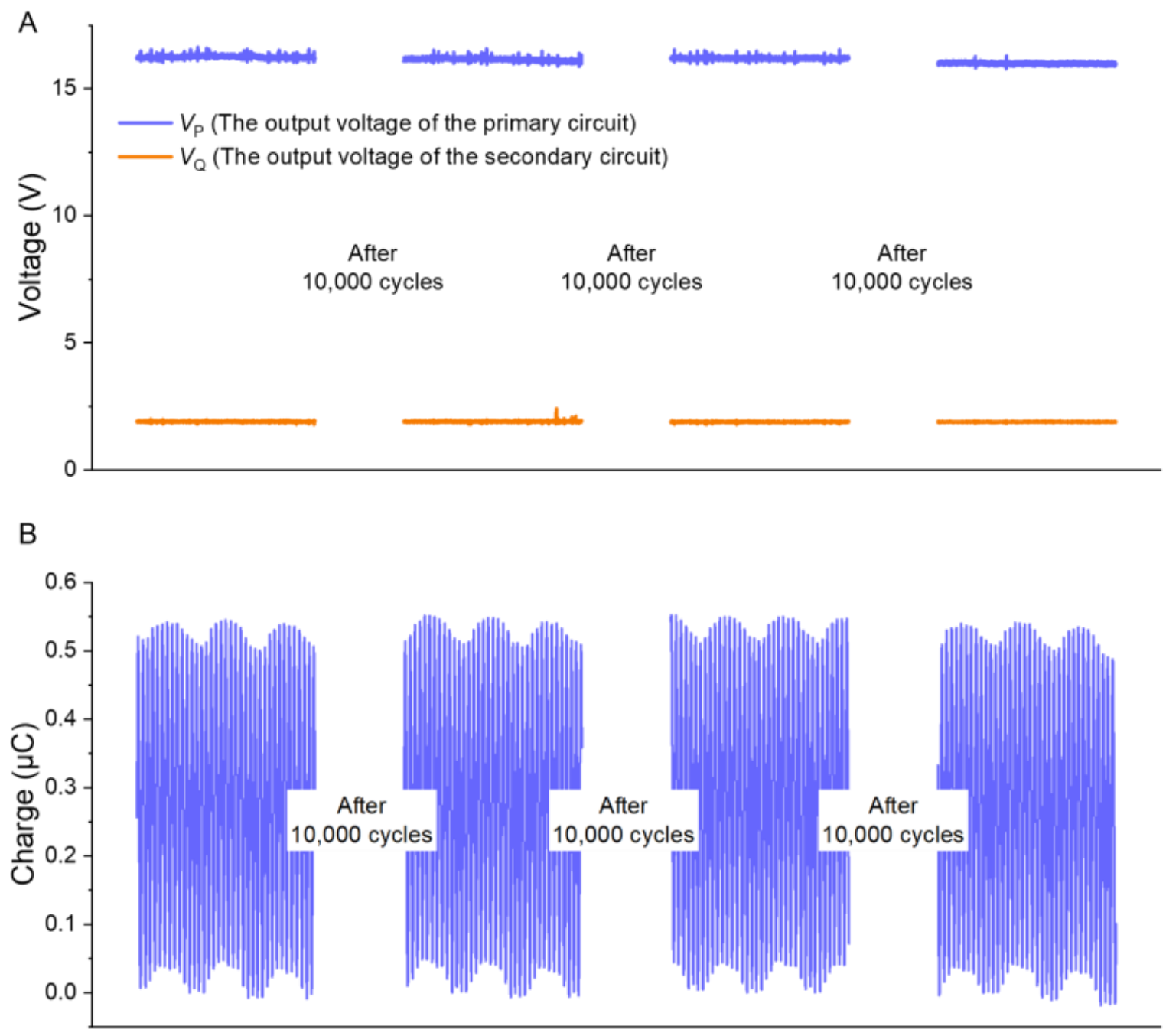

Figure R6. The circuit and TENG stability testing experiments. (A) The output voltage of primary circuit and secondary circuit after 30,000 cycles. (B) The output charge of TENG after 30,000 cycles.

Table R2 (Added Table S7) The stability tests of production rate

	Initial value	After 1 hour
Time (s)	211	205
Yield (μL)	250	250
Production rate (μL/min)	71.43	73.17

In the hydrogen production process studied here, the cathode reaction is:

$2H_2O + 2e^- \rightarrow H_2\uparrow + 2OH^-$

The anode may undergo two reactions:

1. $2Cl^- - 2e^- \rightarrow Cl_2$

   $Cl_2 + 2OH^- \rightarrow Cl^- + ClO^- + H_2O$

2. $2H_2O \rightarrow O_2 + 4H^+ + 4e^-$

To identify the dominant anode pathway, we quantified ClO^- production using ultraviolet-visible (UV-Vis) absorption spectroscopy. The principle is as follows: under acidic conditions, ClO^- oxidizes excess iodide (I^-) to iodine (I_2), which further combines with I^- to form triiodide (I_3^-). I_3^- exhibits a characteristic absorption peak in the UV region (288 nm, shown in **Figure R7A**). Its absorbance is proportional to the I_3^- concentration, allowing ClO^- concentration to be determined via a calibration curve (**Figure R7B**).

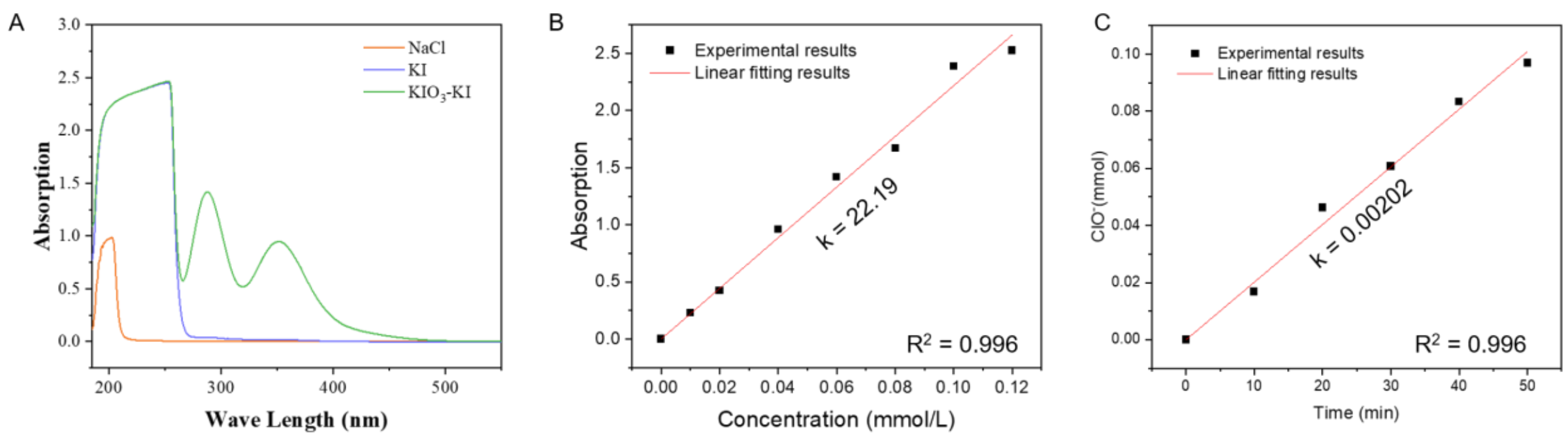

Figure R7 (Added Figure S37) UV-Vis spectra test. (A) UV-Vis spectra of NaCl, KI and KIO_3-KI solution and (B) the standard curve calibrating ClO^- concentration. (C) ClO^- production rate.

During the experiment, a 1 mL sample was taken from solution every 10 min and treated sequentially with:

- 0.1 mol/L KI (providing excess I^-)
- 1 mol/L H_2SO_4 and 0.5 mol/L KOH were used to adjusted PH to about 3.5.
- The mixture was diluted to 25 mL, reacted for 10 min, and then analyzed by UV-Vis.

The measured ClO^- production rate was 2.02 μmol/min (**Figure R7C**). Based on the stoichiometry of the reaction between anodically generated Cl_2 and OH^- ($Cl_2 + 2OH^- \rightarrow Cl^- + ClO^- + H_2O$), this rate is directly equivalent to the rate of Cl_2 generation that subsequently reacted to form ClO^-. This indicates that at least 69.2%

of the anode current was utilized for Cl_2 generation that contributed to the measured ClO^- (H_2 production rate is 2.92 μmol/min).

Table R3 (Added Table S8) The production rate of anode gas

	The 1st cycle	The 2nd cycle	The 3rd cycle	The 4th cycle	The 5th cycle
Yield (μL)	125	125	125	125	125
Time (s)	978	1052	947	1043	1035
Production rate (μL/min)	7.67	7.13	7.92	7.19	7.24

Gas collected from the anode via water displacement yielded a much lower rate of 7.43 μL/min (≈0.30 μmol/min at SATP) (**Table R3**). Given the high aqueous solubility of Cl_2, this collected gas is primarily composed of O_2. This gas collection data suggests that approximately **20.5% of the anode current** was directed toward the oxygen evolution reaction.

The apparent deficit between the total current and the sum of the currents accountable by ClO^- production (69.2%) and O_2 collection (20.5%) is approximately 10.3%. We attribute this primarily to the loss of Cl_2 via two pathways: (i) the reversibility of the reaction $Cl_2 + 2OH^- \rightleftharpoons Cl^- + ClO^- + H_2O$, and (ii) direct volatilization of Cl_2 gas into the atmosphere, which was corroborated by a distinct pungent odor detected during experiments. Therefore, the **total current fraction for Cl_2 generation** (including the portion that later hydrolyzed to ClO^- and the portion that was lost) was approximately 79.5% (69.2% + 10.3%).

Based on the above analysis, we assign **79.5% of the current to the Cl_2 generation pathway** (with a theoretical thermodynamic voltage of 1.36 V) and the remaining **20.5% to the O_2 generation pathway** (theoretical voltage of 1.23 V). With an actual applied cell voltage of 2.5 V and an overall measured Faradaic efficiency of 97.15%, the total energy efficiency of the system is calculated to be 51.3% using the ratio of the weighted average theoretical voltage to the input voltage.

Conclusion: Due to experimental limitations, a detailed quantitative analysis of the gas composition via gas chromatography (GC) was not performed. Therefore, reasonable assumptions were made regarding the gas collected from the anode. **Nevertheless, the repeated measurements and one-hour stability test for H_2 collection confirm that the performance enhancement is robust and results from the proposed strategy in this work, rather than systematic or measurement errors.** Although constrained by the lack of GC analysis for direct product quantification, the presented analysis based on ClO^- quantification and gas collection provides substantial supporting evidence for the enhanced hydrogen-production rate.

Revisions

The revised part in the present manuscript is as follows:

We have made a revision of "the hydrogen production rate of the self-power hydrogen generation system reaches an impressive 16.5 mL/(min·m^2)" in the last paragraph of "**Introduction**".

We have made a revision of "Specifically, the production rate has increased from 41.34 µL/min to 72.33 µL/min by introducing of the secondary circuit, representing a 75% improvement in overall rate. The detailed electrochemical performance, including the specific reaction voltage and current, is comprehensively presented and analyzed in **Figure S36-37**, **Table S6-8** and **Note S20**." in the last paragraph of "**The multi-stage buck strategy for self-power systems**".

We have made a revision of "Integrated self-powered hydrogen systems exhibit 16.5 mL/(min·m^2) production rate, also representing a nearly order of magnitude increase compared to prior best of 2.5 mL/(min·m^2)." in the first paragraph of "**Discussions**".

We have revised the **Figure 5G** as follow:

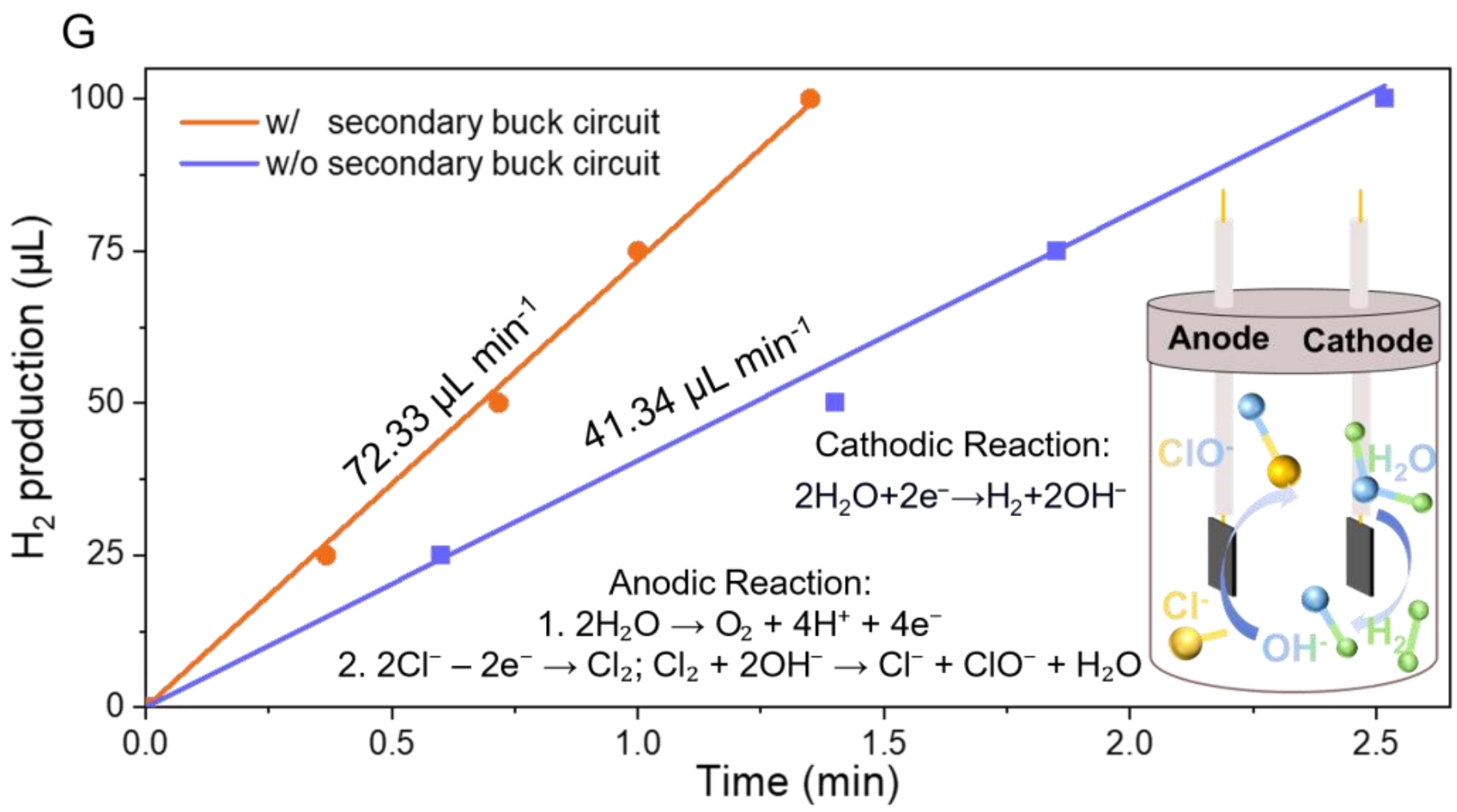

Revised Figure 5G The production rate of the self-powered hydrogen production system. The concentration of the NaCl solution used is 1 mol/L.

The revised part in the Supplementary Information is as follows:

We have revised the **Figure S36** as follow:

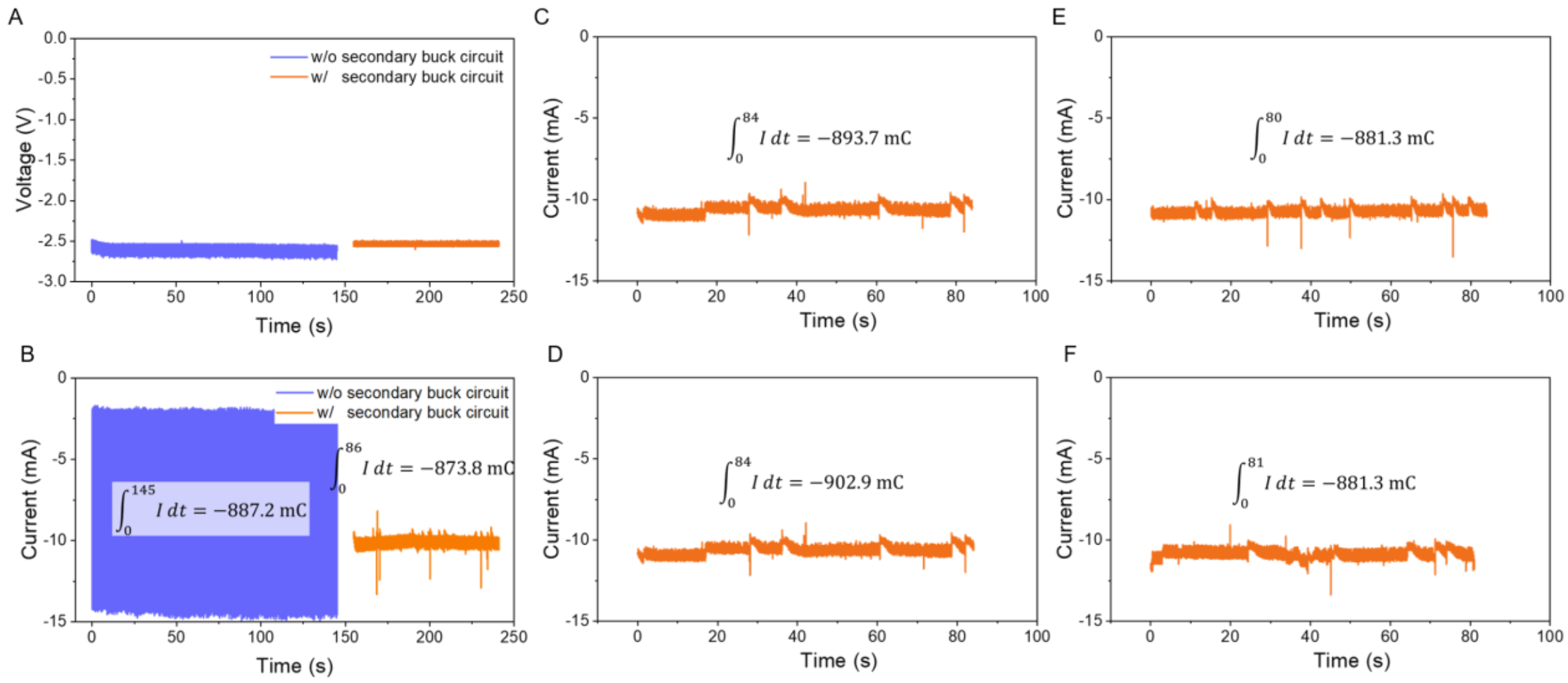

Figure S36. The operating voltage current of the self-power hydrogen production system. (A) Test voltage with/without secondary buck circuit; (B)-(E) Test current.

We have added the above discussion into the "**Note S20 TENG-Driven Electrochemical Hydrogen Production**" in current Supplementary Information.

We have added the "**Table S6 The production rate of hydrogen and faradaic efficiency**", "**Table S7 The stability tests of production rate**" and "**Table S8 The production rate of anode gas**" in current Supplementary Information.

We have added the "**Figure S37. UV-Vis spectra test.**" in current Supplementary Information.

Comment 3

Third, although the use of Q–V characteristics to guide circuit optimization is a conceptually elegant approach, the manuscript does not sufficiently delineate how this method differs from prior works that have employed Q–V-guided matching or multi-loop PMC strategies. Several studies from 2022–2024 have already explored dual-loop or charge-pump–buck combinations, though typically with efficiencies below 60% at 5 V. To strengthen the novelty claim, the authors should clarify whether their primary contribution lies in the system-level coordination of node voltages or in theoretical derivation of the switching threshold from Q–V analysis. A figure or table contrasting their architecture with representative prior approaches would be particularly useful.

Question: How does the proposed "multi-stage buck strategy" fundamentally differ from previously reported dual-loop or charge-pump–buck power-management circuits, and can the authors provide a comparative schematic or performance table to clarify this distinction?

Response: We sincerely thank the reviewer for the careful evaluation and constructive suggestions.

1. **Double Loops Power Management Circuit (DL-PMC)**

   The TENG used in DL-PMC work is Pulsed TENG, and the energy transfer path is: Pulsed TENG → Inductor (***L***) → Output capacitor (C_{out}) (**Figure R8**). For conventional PMC, when energy is transferred from the inductor to the C_{out}, it must pass through two diodes in the rectifier bridge, resulting in a significant voltage drop and increased energy loss[3]. To address this, DL-PMC strategy extra freewheeling diode in parallel between the inductor and C_{out}, allowing most of the energy to pass through only one diode, thereby reducing conduction energy loss.

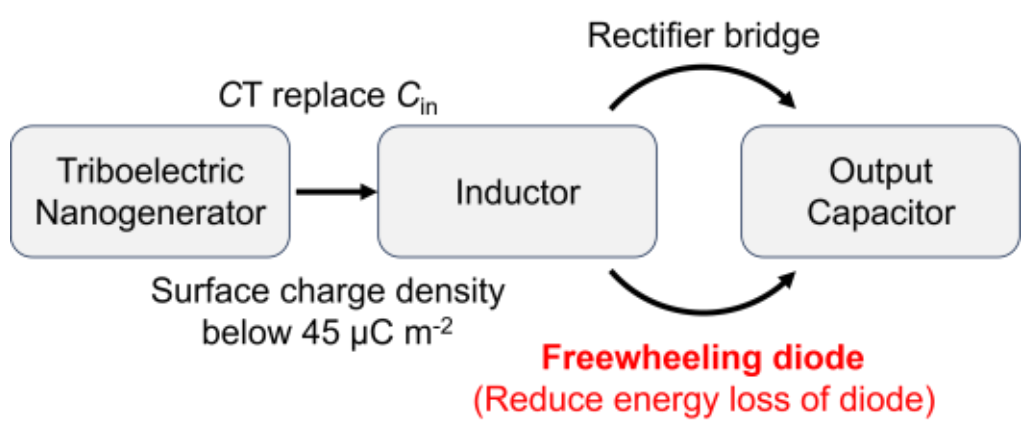

Figure R8. The energy transfer path of DL-PMC. Freewheeling diodes replace rectifier bridges to reduce energy loss.

   However, the Pulsed TENG essentially combines a freestanding TENG (FS-TENG) with a mechanical switch, where the TENG's inherent capacitance (C_T) replaces the input capacitor (C_{in}) of the PMC. This often leads to a relatively low surface charge density (estimated from the reported data as ~33 $\mu C \cdot m^{-2}$), which is only near the theoretical lower limit imposed by air breakdown under open-circuit conditions (~45 $\mu C \cdot m^{-2}$) and is far below the level of current high-performance TENGs. Therefore, such pulsed TENGs are not a suitable reference for power management of high-performance devices.

   Moreover, although that work recognizes the impact of diode voltage drops on efficiency, its DL-PMC still exhibits an efficiency below 60% at 5 V output, with even lower efficiency at reduced voltages (e.g., 1.8 V,

3.6 V). This indicates that the **fundamental issue of low efficiency at low voltages remains unresolved**.

2. **Charge-pump–buck circuit**

   The design of charge-pump－buck circuit generally consists of two stages: a front-end charge-pump circuit that aims to raise the TENG's output charge density toward the limit determined by the device structure and air breakdown, and a back-end power management circuit that follows one of two approaches: Charge-pump TENG → C_{in} → Inductor → C_{out}, or Charge-pump TENG → Inductor → C_{out} (**Figure R9**). Previous studies by Cao et al. have shown that for high-performance CS-TENGs with small amplitude and thin films, the second approach (without C_{in}) can harvest more energy[4], which is consistent with the conclusion of our previous work[5].

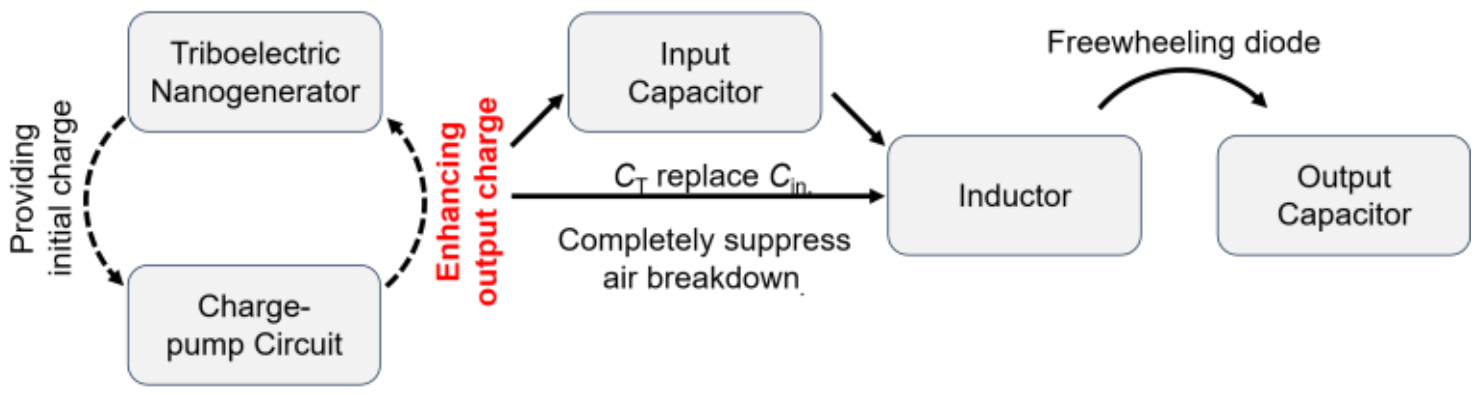

Figure R9. The energy transfer path of Charge-pump–buck circuit. Charge-pump circuit is used to enhance the output of TENG.

   Nevertheless, these optimizations still **fail to solve the core problem of low efficiency at low voltages**. More importantly, this strategy (C_T replaces C_{in}) above works requires that the surface charge density of the TENG does not decay as the voltage increases—meaning it is only applicable to devices whose surface charge density remains below the minimum air-breakdown limit or that can completely suppress air breakdown during voltage increase. As a result, **the strategy lacks universal applicability and cannot be broadly generalized to most high-performance TENGs**.

Our work in 2024 pointed out that most reported high-performance TENGs experience surface charge decay (**Note S2**) during power management due to effects such as electrode edge electrostatic breakdown and material current leakage[6]. This implies that **power management strategies based solely on the conventional CMEO theory** (cycle for maximum energy output, which was first proposed by Zi et al) **and guided only by the *V-Q* plot are not feasible**. The commonly observed significant performance degradation of high-performance TENGs when connected to existing PMCs further confirms the theory lack of a suitable design guideline for such devices (**Table S1**).

3. **Multi-Stage Buck Strategy**

   To address this, we proposed using Coulombic efficiency (the ratio of the actual output charge to the short-circuit charge) and the *Q-V* curve to quantify this collapse phenomenon. We found that maximum output energy is achieved when the Coulombic efficiency is around 50%. Accordingly, in this work, we introduce a *Q-V* curve guided PMC design strategy that sets the circuit operating voltage at the point corresponding to maximum energy extraction, thereby harvesting more energy from high-performance

TENGs. The experimental feasibility of this approach has been verified in the manuscript, and its theoretical basis is further explained in **Note S6**. **This corresponds to the first key node voltage regulated in our "multi-stage buck strategy," aiming to solve the problem of efficient matching between high-performance TENGs and PMCs.**

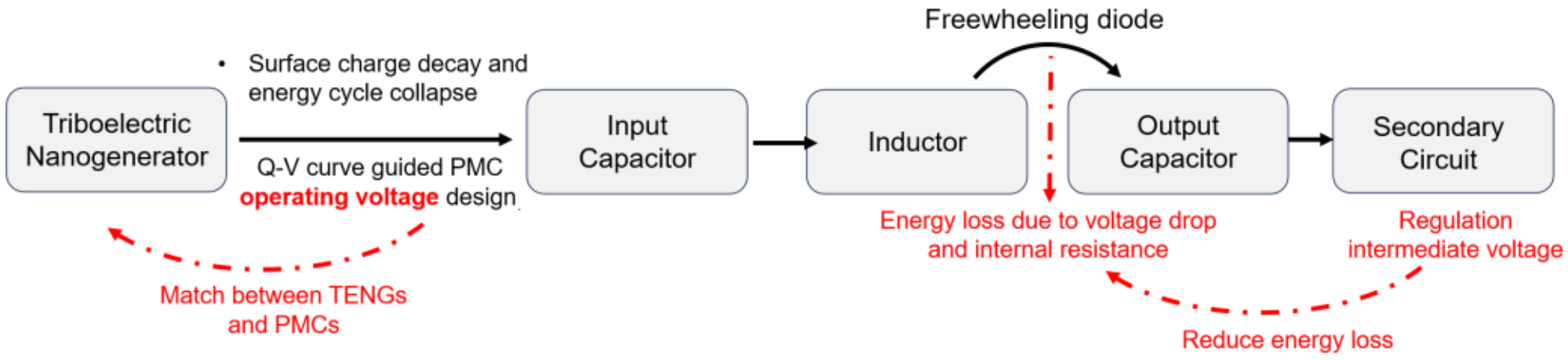

Figure R10. The energy transfer path of multi-stage buck circuit. *Q-V* curve guided PMC design to match between TENGs and PMCs. Regulation intermediate voltage by secondary circuit reduces the energy loss of primary.

The second key node voltage regulated in our strategy is the output voltage of the primary circuit (i.e., the input/intermediate voltage of the secondary circuit). Its purpose is to resolve the low-efficiency issue at low voltages. As discussed in **Comment 1** and the **Note S14**, this phenomenon is widespread in commonly used high-performance PMCs for TENGs (LC, FSCC, and transfer circuits). Raising the primary-circuit voltage can significantly improve its efficiency. Coupled with a high-performance commercial buck chip as the secondary stage, this enables efficient utilization of TENG energy at the target output voltage. The energy transfer path is shown in **Figure R10**.

4. **Summary**

Here, we conduct a comparison of above two works (Ref 23 and Ref 10) and other works cited in this manuscript from five aspects: the TENG-PMC Matching Mechanism, TENG-PMC Matching Method, Circuit Architectures, Optimization Strategies, as well as performance, as shown in the **Table R4**. Regarding the TENG-PMC matching mechanism, previous studies have primarily relied on the *V-Q* plot obtained from the CMEO theory, which assumes the surface charge density is stable as the voltage increase. Consequently, such methods are only suitable for devices with lower performance or those in which air breakdown can be completely suppresses by circuit design. Their applicability to high-performance devices is questionable, and employing these existing circuits often leads to significant performance degradation (**Table S1**). In contrast, our work is based on the energy cycle collapse theory and utilizes the *Q-V* curve to guide the design of the circuit's operating voltage. This approach is broadly applicable to most high-performance devices, thereby achieving substantially higher power density and current density. In terms of circuit optimization, prior works have focused more on component-level improvements while fundamentally neglecting the issue of efficiency degradation under low voltage. Our study not only identifies and explains this critical problem but also provides a feasible solution by regulating the intermediate voltage of the secondary circuit, ensuring high efficiency even under low-voltage conditions.

Table R4 (Added Table S10) Comparison of circuit design and optimization of different works

	Ref.12	Ref.19	Ref.24	Ref.11	Ref.17	Ref.23	Ref.13	Ref. 25	Ref. 10	This work
TENG-PMC Matching Mechanism	The *V*-*Q* plot based on ideal CMEO theory									The *Q*-*V* curve based on energy cycle collapse theory
TENG-PMC Matching Method	C_{in} extracts and stores energy			TENG's inherent capacitor directly serves as the storage unit.						Regulation the circuit operating voltage based on *Q*-*V* curve
Circuit Architectures	LC	FSCC	LC	LC	Transformer coupled circuit	LC	LC	LC & Commercial chips	Charge-pump circuit & LC	Multi-Stage Buck Strategy
Optimization Strategies	Utilizes SCR to achieve passive switching design	Fractional circuit to reduce voltage drop of diode	Optimized GDT	Optimized inductor	Optimized transformer	Double loops design	Optimized capacitor; Half-wave circuit completely suppresses air breakdown	Reduced charge and voltage losses; Achieved stable voltage output	Half-wave circuit completely suppresses air breakdown	Regulation the intermediate voltage of secondary circuit
Performance	1. Most optimal circuit efficiencies exceed 80%; however, efficiency significantly decreases below 5V.<br>2. The power density of the TENG used does not exceed 1 W/m^2. Whether it is suitable for high-performance TENG (power density exceeding 10W/m^2) is a question.									1. Efficiency remains around 80% even at low voltage.<br>2. Output power density exceeds 10 W/m^2.<br>3. Current density reaches up to 5.19 A m^{-2}

Revisions

The revised part in the present manuscript is as follows:

We have made a revision of "Comparison presented in **Table S10** and **Note S22** highlights that the energy cycle collapse theory adopted in this work differs fundamentally from the ideal CMEO theory commonly used in previous works, being particularly suitable for high-performance TENGs where air breakdown is difficult to avoid. The systematic nodal voltage regulation method within the proposed multi-stage buck strategy transcends conventional component-level circuit optimization, establishing a new paradigm for TENG power management design." in the first paragraph of "**Discussions**".

The revised part in the Supplementary Information is as follows:

We have added the above discussion into the **"Note S22 Comparison of circuit design and optimization of different works"** in current Supplementary Information.

We have added the **"Table S10 Comparison of circuit design and optimization of different works"** in current Supplementary Information.

Comment 4

Fourth, while the authors demonstrate the applicability of their approach using both rolling-mode and rotary-mode TENGs, further evidence of universality and scalability would strengthen the impact of the work. Since contact-separation and lateral-sliding TENGs dominate many practical implementations, validating the approach on one additional TENG configuration—or at least discussing its theoretical applicability—would support the claim that the method provides a generalized solution for TENG power management.

Question: Could the authors extend their study to another TENG architecture or provide simulations showing that the same *Q*–*V*-based optimization principle holds for contact-separation or lateral-sliding modes?

Response: We sincerely thank the reviewer for the careful evaluation and constructive suggestions.

The proposed strategy for voltage regulation based on the *Q* – *V* curve is primarily aimed to the "energy cycle collapse" issue observed in high-performance TENGs. In such devices, factors such as air breakdown near electrode edges or material leakage current cause the surface charge density to decrease as the output voltage rises, preventing the energy cycle from reaching an ideal state.

In contrast, if the surface charge density remains stable during voltage increase (named as energy cycle in stable state), prior studies have confirmed that the method—replacing the input capacitance (C_{in}) of the PMC with the TENG's inherent capacitance (C_T)—represents the optimal solution[4,5]. Such stable state typically occurs in devices with low surface charge density or where circuit regulation can fully suppress air breakdown at high voltages. CS-TENGs can employ this strategy for PMC design.

Therefore, the key criterion for determining the applicability of the *Q*–*V* method is not the motion type of the TENG (e.g., rolling, rotary, contact-separation, or sliding), but whether its energy cycle exhibits collapse characteristics—specifically, whether surface charge density declines as voltage increases. In our previous work[6], we have demonstrated that sliding-mode DC-TENGs, AC-TENGs, and their structural variants all display behavior consistent with the energy collapse theory, validating that our design principle also applies to these devices. Furthermore, the theory related to selecting the operating voltage of the PMC based on the *Q*–*V* curve has been verified in **Note S6**.

Here, we also have supplemented the experimental results applying the *Q*–*V* curve to sliding-mode devices, which further support the conclusions above. **Figure R11A** illustrates the schematic structure of the sliding-mode TENG. **Figures R11B** and **Figures R11C** present its output charge and output current curves, respectively. **Figure R11D** shows the corresponding *Q*–*V* characteristic curve. The test results indicate that the TENG achieves optimal output performance when the switching voltage of the primary circuit is set to 2500 V. **Figure R11E** compares the performance under the same load (0.1 MΩ) with switching voltages set to 1500 V, 2500 V, and 3500 V, demonstrating that the performance at 2500 V is the best, consistent with the *Q*–*V* curve results. This further confirms the rationale of using the *Q*–*V* curve to guide the design of the TENG PMC design.

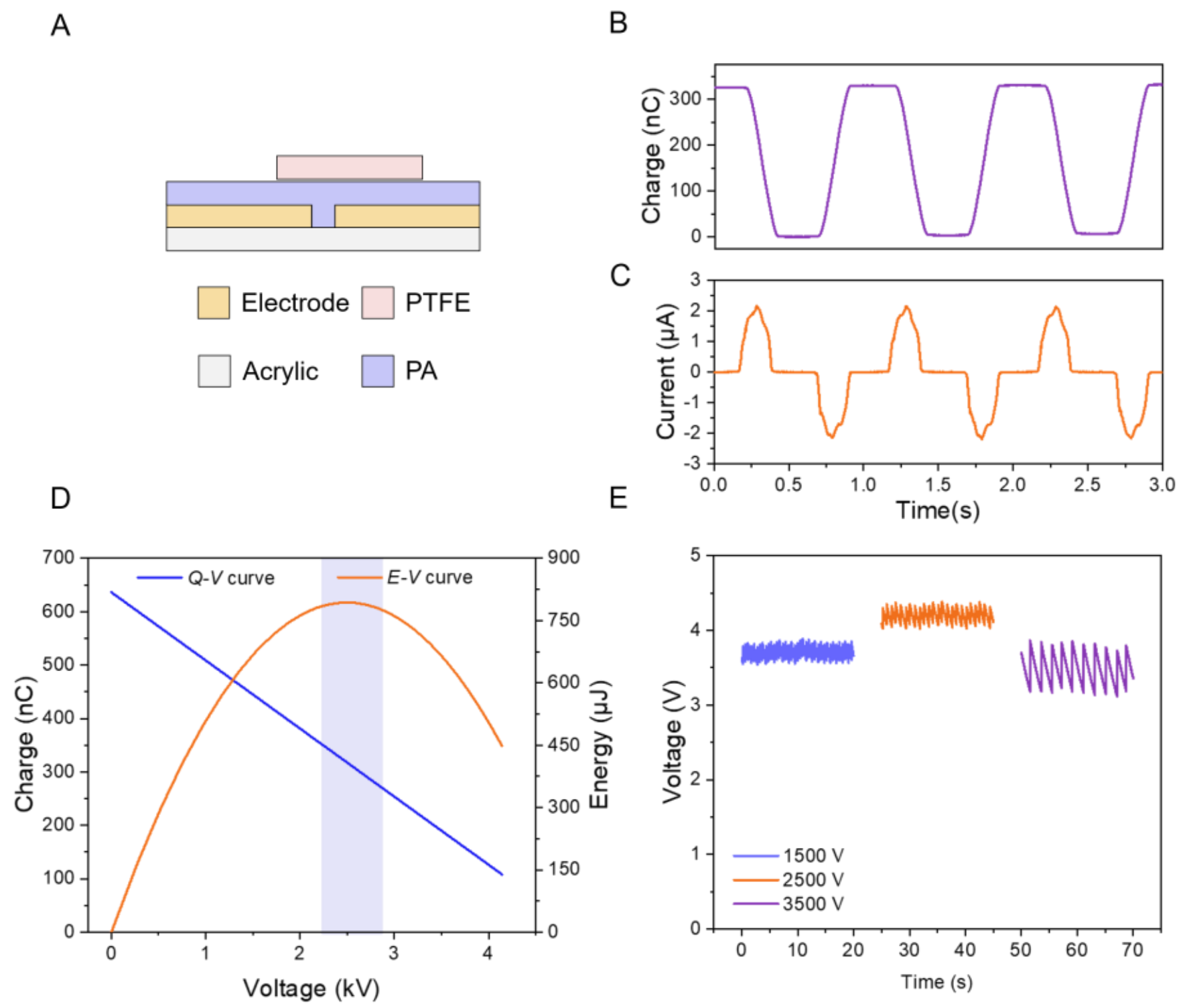

Figure R11 (Added Figure S38). The performance of sliding-mode TENG. (A) The schematic structure of TENG. The effective area of the device is 20 cm^2, and the motion frequency is 1Hz; (B) The output charge; (C) The output current; (D) The Q-V curve; (E) The output voltage of TENG with PMC switching voltages set to 1500 V, 2500 V, and 3500 V. The resisitance is 0.1 MΩ.

Revisions

The revised part in the present manuscript is as follows:

We have made a revision of "This strategy reveals the inherent relationship between TENG output characteristics and circuit design parameters through the *Q-V* characteristic curve, guiding circuit optimization to maximize output power. Validation has been achieved in experiments involving rolling-mode, rotary-mode, and sliding-mode TENGs (**Figure S38** and **Note S21**)." in the first paragraph of "**Discussions**"

The revised part in the Supplementary Information is as follows:

We have added the above discussion into the **"Note S21 *Q-V* curve guided voltage selection for PMCs: experimental validation in sliding-mode devices"** in current Supplementary Information.

We have added the **"Figure S38 The performance of sliding-mode TENG."** in current Supplementary Information.

Comment 5

Fifth, given that the primary buck stage operates at approximately 1.5 kV, details regarding insulation, circuit safety, and dielectric breakdown prevention are essential. The supplementary information briefly mentions polyimide insulation, but more quantitative data on leakage current, dielectric strength, and environmental stability (humidity and temperature dependence) should be presented to ensure reproducibility and safety of the high-voltage design.

Question: What specific insulation materials, spacing, and design standards were used to prevent dielectric breakdown in the 1.5 kV primary circuit, and how do these parameters affect long-term reliability?

Response: We sincerely thank the reviewer for the careful evaluation and constructive suggestions.

To ensure the insulation safety and long-term reliability of the primary circuit, some protection methods were implemented in this work, as detailed below with supporting data.

1. **Air Gap Insulation and Circuit Layout**

   Air breakdown is a critical consideration in high-voltage circuit design. We characterized the breakdown voltage for various air gaps. **Figure R12A** show that a 3 mm air gap can withstand up to 4400 V. In the circuit layout, air gap between high-voltage nodes were strictly controlled. As shown in **Figure R12B**, the high-voltage (red), output (purple), and ground (blue) nodes are clearly identified. **Figure R12C** shows the minimum distances between these critical nodes. This layout guarantees that internal air breakdown will not occur at the 1.5 kV operating voltage.

2. **Solid Insulation**

   To eliminate safety hazards and suppress corona discharge at pin tips of electronic components, all high-voltage pins were insulated with polyimide (PI). The employed PI material was characterized: at the 1500 V DC operating voltage, the leakage current is below 1 nA (**Figure R12D**); the dielectric strength of the 50 μm-thick film is approximately 340 V/μm, corresponding to a breakdown voltage of ~17 kV, which is an order of magnitude higher than the operating voltage (**Figure R12C**). Furthermore, an 8-hour continuous withstand voltage test at 1500 V showed stable leakage current, confirming the material's reliability (**Figure R12D**). When the temperature increased from 20°C to 60°C, the mobility of carriers within the dielectric material rose accordingly. Under a test voltage of 1500V, the leakage current increased to approximately 18nA (**Figure R12E**), while the insulation properties remained excellent.

3. **TENG Insulation Strategy**

   Rolling TENG: The air gap between electrodes is set to 5 mm, which is sufficient to prevent air breakdown at the 1.5 kV operating voltage.

   Rotary TENG: For higher integration density, the electrode gaps were reduced to 2 mm and 1.5 mm. Consequently, prior to triboelectric layer deposition, the electrodes were pre-wrapped with PI film. This

method effectively suppresses corona discharge and elevates the local breakdown threshold, thereby ensuring stable high-voltage output. Its efficacy has been demonstrated in our previous work[6].

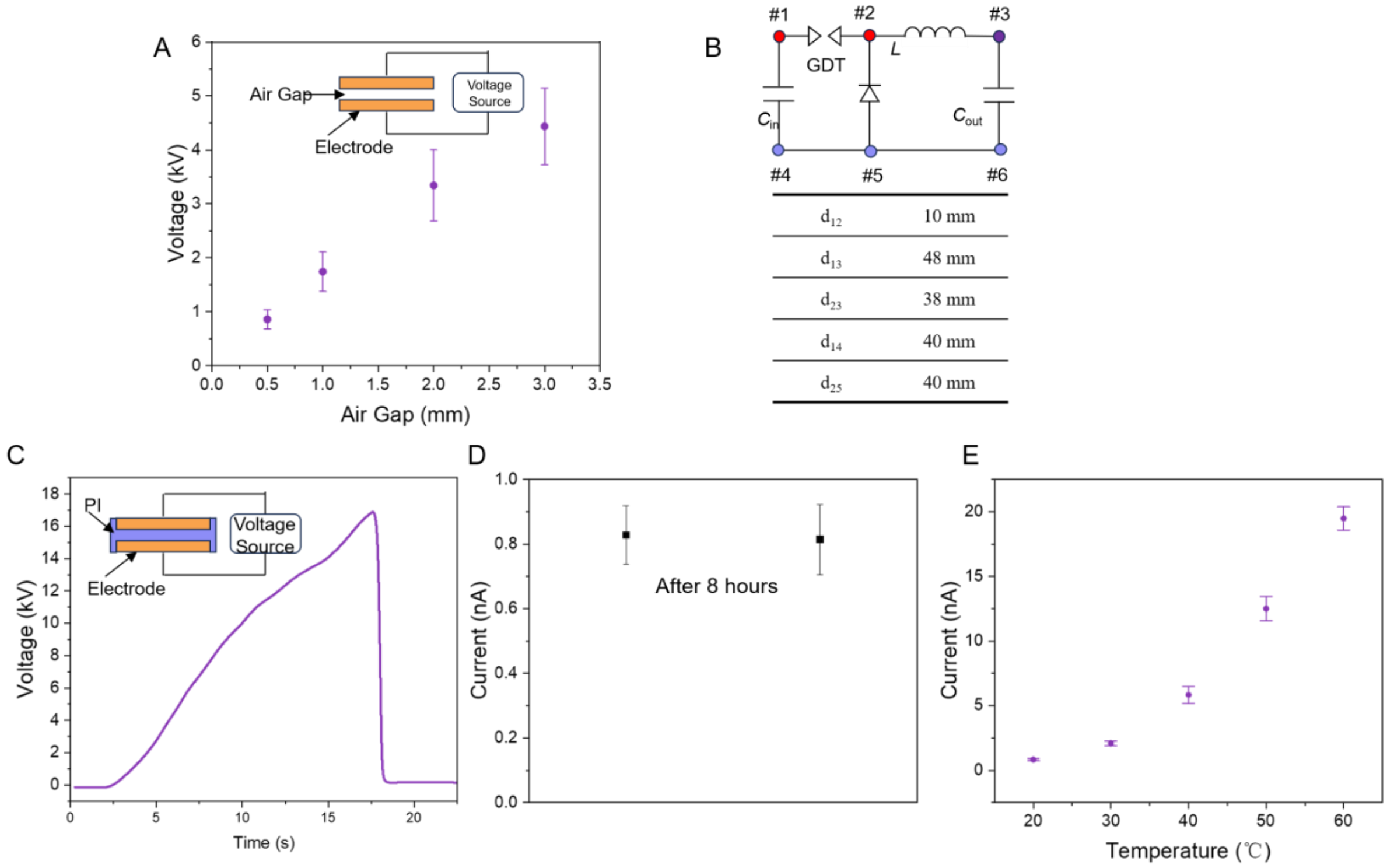

Figure R12 (Added Figure S9) The insulation performance of air and PI. (A) Breakdown voltage under different air gaps (the test circuit is shown in the illustration); (B) Minimum distance between primary circuit pins; (C) Dielectric strength test of PI (the test circuit is shown in the illustration); (D) Leakage current of PI at 1500V (temperature is 20 °C); (E) Leakage current test of PI under different temperatures (voltage is 1500 V).

Revisions

The revised part in the present manuscript is as follows:

We have made a revision of "The air gap between electrodes is set to 5 mm to avoid air breakdown inside the device. The electrodes, which are made of copper, are prepared using printed circuit board (PCB) technology on an epoxy resin substrate that is 1 mm thick." in the "**Method**"

We have made a revision of "A 50-μm-thick polyimide (PI) layer was then attached to the electrode surface to enhance the device's insulation performance. Subsequently, the PA/PTFE film was adhered above the PI." in the "**Method**"

The revised part in the Supplementary Information is as follows:

We have added the above discussion into the **"Note S9 Insulation design considerations for TENGs and PMCs"** in current Supplementary Information.

We have added the **"Figure S9. The insulation performance of air and PI."** in current Supplementary Information.

Comment 6

Finally, the data presentation can be improved for quantitative clarity. Efficiency values should include error bars or statistical variations from repeated measurements, as well as explicit uncertainty sources. The manuscript would also benefit from a concise summary table comparing the proposed strategy with existing PMCs (including LC, transformer, SCC, and commercial ICs) at standard output voltages. This would make the performance comparison transparent and accessible for readers.
Question: Can the authors include statistical analysis of efficiency data and a summary table comparing their method with state-of-the-art PMCs under standard output voltages (1.8 V, 3.3 V, and 5 V)?

Response: We sincerely thank the reviewer for the careful evaluation and constructive suggestions.

Comparative Summary Table: A summary table comparing the performance of our proposed strategy with conventional PMCs (including LC, transformer, FSCC) has been added (**Table R5**). We note that many existing works on do not explicitly report efficiency data with statistical details or standardized outputs in their works. Therefore, the data for these works presented in the table were extracted from the performance curves or values reported in the respective references.

Statistical Analysis: We have conducted five independent measurement sets for our circuit's output at various voltages (**Table R6**). The resulting statistical analysis (mean ± standard deviation) has been incorporated.

Table R5 (Added Table S3) The reported efficiency in different works*

REF	Circuit	η (1.8 V)	η (3.6 V)	η (5.0 V)	Optimal efficiency
11	LC	32.1%	45.0%	51.1%	81.6%
12	LC	51.1%	67.8%	73.6%	89.8%
17	Transformer	42.6%	55.8%	60.3%	78.5%
19	FSCC	35.2%	62.5%	84.7%	94.5%
23	LC	18.3%	37.8%	48.1%	90.6%
24	LC	36.3%	50.4%	55.2%	83.9%
This Work	Multi-stage buck circuit	75.5%	86.3%	85.1%	90.9%

*The data for these works presented in the table were extracted from the performance curves or values reported in the respective references.

Table R6 (Added Table S2) The results of five measurements of circuit efficiency

	1st	2nd	3rd	4th	5th	Mean	Standard Deviation
1.8V	75.0%	75.4%	75.6%	75.4%	76.3%	75.5%	0.47749%
3.6V	85.6%	86%	87.4%	86.8%	85.9%	86.3%	0.74027%
5.0V	84.6%	85%	85.3%	85.4%	85.3%	85.1%	0.30822%

Revisions

The revised part in the present manuscript is as follows:

We have made a revision of "Additionally, the overall efficiency of the circuit (including both the primary and secondary circuits), improves from 73.5%, 64.6% and 42.2% to 85.1%, 86.3% and 75.5% respectively, as verified through multiple tests (**Table S2**). Furthermore, as verified through circuit stability testing experiments (**Figure S24**), after 30,000 working cycles of TENG, the output voltages of both the primary and secondary circuits can still maintain a stable state." in the second paragraph of the "**Regulation of input voltage for secondary buck circuit**".

The revised part in the Supplementary Information is as follows:

We have added the above discussion into the **"Note S9 Insulation design considerations for TENGs and PMCs"** in current Supplementary Information.

We have added the **"Table S3 The reported efficiency in different works"** and **"Table S2 The results of five measurements of circuit efficiency"** in current Supplementary Information.

In conclusion, the manuscript presents a technically interesting approach that could contribute to the advancement of efficient power management for TENGs. However, the scientific foundation and comparative validation are not yet sufficient to justify publication in Nature Communications. The concept is incremental rather than revolutionary in circuit design, and the practical gains, while notable, need stronger experimental proof and clearer theoretical grounding. If the authors can deliver robust benchmarking, quantitative electrochemical data, and improved mechanistic analysis, the paper could become a valuable contribution to the field. At its current stage, however, it remains primarily an engineering optimization with insufficient depth of scientific validation for this journal.

Response: Based on the valuable comments, we have provided detailed responses and additional data, summarized as follows:

- **Originality of the Theoretical Foundation**: The *Q-V* curve-guided circuit design method proposed in this work originates from a profound understanding of the energy cycle collapse mechanism in TENGs. This represents a fundamental difference from the design strategies of conventional PMCs. Our approach to addressing circuit efficiency is not merely focused on parameter optimization of individual components. Instead, it systematically reveals that the key to high-efficiency output lies in optimizing the complete step-down path from the TENG to the application—a critical aspect that has been largely overlooked in previous studies. It is precisely this principled breakthrough and systematic solution to a key scientific challenge in TENG field that has enabled us to achieve remarkable improvements in current density, power density, and hydrogen production rate.
- **Generality Verification and Artifact Exclusion**: The guiding significance of *Q-V* curve has been verified successfully replicated with sliding-mode TENGs, further attesting to its generality. Direct comparison of different circuits under identical mechanical excitation confirms that the performance enhancement stems from the superiority of our core circuit design, not from test environment variations or accidental factor. Combined quantitative electrochemical analysis and product characterization consistently rule out potential measurement artifacts and demonstrate the long-term operational stability of the system.
- **Engineering Reliability and Optimized Data Presentation**: The supplemented analysis on high-voltage insulation and layout optimization ensures the practical feasibility of this strategy. We have optimized the data presentation to provide a more comprehensive and fair comparison between our circuit and previously reported high-performance circuits.

We hope that our responses and additional evidence have adequately addressed all your concerns. We sincerely appreciate your insightful comments, which have significantly strengthened the completeness and persuasiveness of this work.

References

1. Liu W, *et al.* Switched-capacitor-convertors based on fractal design for output power management of triboelectric nanogenerator. *Nature Communications* **11**, 1883 (2020).
2. Wang Z, *et al.* Ultrahigh Electricity Generation from Low-Frequency Mechanical Energy by Efficient Energy Management. *Joule* **5**, 441-455 (2021).
3. Zhang Z, *et al.* Double loops power management circuit of pulsed triboelectric nanogenerator with enhanced efficiency at low operating voltage and its application in self-powered flue gas monitoring system. *Nano Energy* **110**, 108360 (2023).
4. Cao Z, *et al.* Ultrahigh power generation from low-frequency and micro motion by suppressing air breakdown. *Energy & Environmental Science* **17**, 5002-5012 (2024).
5. Gao Y, *et al.* Achieving high-efficiency triboelectric nanogenerators by suppressing the electrostatic breakdown effect. *Energy & Environmental Science* **16**, 2304-2315 (2023).
6. Gao Y, *et al.* Spontaneously established reverse electric field to enhance the performance of triboelectric nanogenerators via improving Coulombic efficiency. *Nature Communications* **15**, 4167 (2024).

Response to Reviewer #2

The manuscript entailed, "Multi-Stage Buck Strategy for Power Management of Triboelectric Nanogenerators" presents multistage buck strategy for Triboelectric nanogenerators (TENGs) that synergistically combines dual-stage voltage conversion circuit with TENG's output characteristic by the *Q*-*V* curve, enabling precise voltage regulation at critical nodes for performance improvement. As per the demonstrated results, the proposed strategy achieves approximately 80% circuit efficiency at the low-voltage range while delivering 3.85 A/m^2 current density at 2.5 V. The work is worthy to publish in this journal.

Response: We highly appreciate the reviewer for carefully reviewing our work, and thank your positive comments on our research work, which has encouraged us greatly.

Response to Reviewer #3

The paper addresses a significant limitation in TENG power management and presents a technically robust solution. However, before publication, the authors should provide:

1. The claimed novelty over existing transformer-based and switched-capacitor TENG PMCs (Refs. 11–19) should be made more explicit. Please elaborate how the "multi-stage buck" differs in operational principle, not only in topology but also in efficiency optimization mechanism.

Response: We highly appreciate the reviewer for carefully reviewing our work and reasonable suggestion.

Compared to conventional single-stage buck circuits, such as transformer-based or switched-capacitor types, the multi-stage buck strategy proposed in this work is not merely an optimization of circuit topology. Altering the topology alone is insufficient to achieve the performance reported in our work (e.g., current density, power density, circuit efficiency, and hydrogen production rate). Instead, our proposed strategy aims to address the two core issues below through precise regulation of key voltage nodes, thereby enabling the aforementioned high performance.

First, the issue of low efficiency under low voltage. Although many studies report optimal conversion efficiencies of PMCs as high as over 90%, when actually applied in electronic devices operating below 5V, the practical circuit efficiency is often significantly lower than the reported values as shown in **Figure R13.** What are the main reasons behind this efficiency gap, and what are the potential approaches to address this issue?

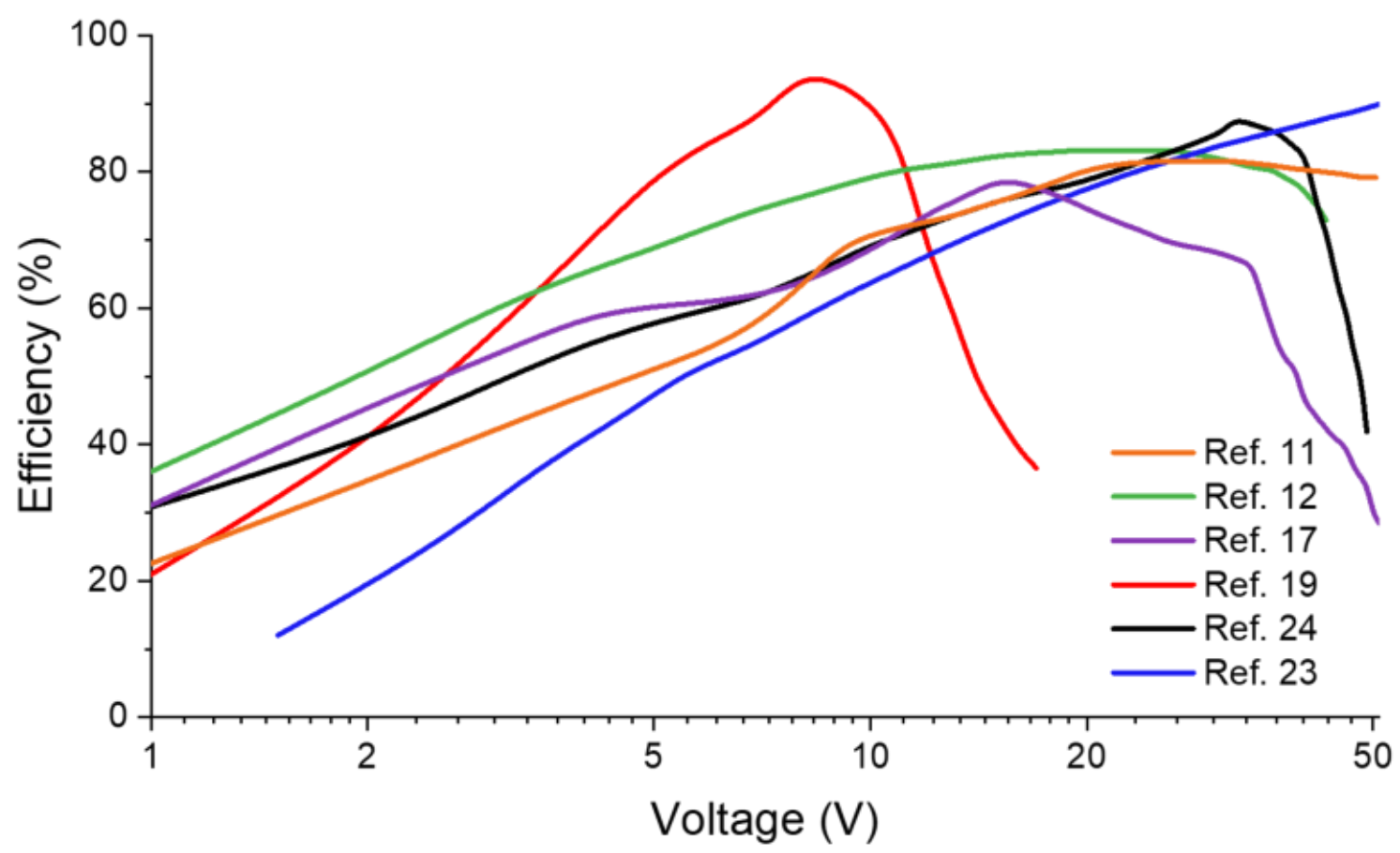

Figure R13 (**Revised Figure S1**) Efficiency of different circuits in different works.

The second issue is the significant drop in output power and current density exhibited by high-performance TENGs when operating under low-voltage after the integration of a PMCs. As shown in **Table R7**, for the high performance TENGs from recent years (the power density over 10 W/m^2), their actual output power with PMC is often less than one-tenth of the reported optimal performance (**Table R7**). Beyond the reason of reduced circuit efficiency at low voltage, what are the deeper underlying constraints causing this problem?

Table R7 (Revised Table S1) Performance of different high-performance TENGs with/without PMCs*.

Ref.	26	27	28	29	30	31	32
Power density without PMCs/Input power density (W/m^2)	7.62	21.6	20.7	12.3	87.26	32.12	23.2
Current density without PMCs (A/m^2)	0.0084	0.0121	0.0067	0.0129	0.032	0.012	0.0061
Power density with PMCs/Output power density (W/m^2)	0.17	0.68	0.33	0.34	0.38	1.41	0.23
Current density with PMCs (A/m^2)	0.12	0.44	0.22	0.24	0.26	0.71	0.11
The ratio of power density with PMCs to power density without PMCs	2.2%	3.1%	1.6%	2.8%	0.4%	4.4%	1.0%
Frequency (Hz)	1	1	1	2	1	2	0.375
The type and operating voltage (kV) of the switch	SCR < 0.8	SCR < 0.8	SCR < 0.8	SCR < 0.8	SCR < 0.8	SCR < 0.8	GDT 0.8
Circuit architectures	LC	LC	LC	LC	LC	LC	LC

*Some works did not report the relevant current density and power density after PMCs; therefore, comparisons were made based on the average charging rate and average power when the capacitor was charged to approximately 3V.

Regarding the first issue, our analysis identifies that the primary reason for efficiency drop in low output voltage is the excessive energy loss caused by diodes and internal resistance. As shown in **Figure R14**, the energy loss increases as output voltage decreases. In addition to LC circuits, we have extended the discussion in the **Note S6** to include FSCC and transformer -coupled circuits, and experimentally demonstrated that this issue is common across all reported circuits to date. Therefore, we designed a multi-stage buck circuit with an intermediate voltage of 16V, which significantly improves the efficiency of the primary circuit. Coupled with a high-efficiency LTC-3588 integrated circuit as the secondary circuit, this design effectively overcomes the low-efficiency limitation at low output voltage of conventional single-stage circuits. More importantly, our experimental comparisons of two step-down paths (1500V→4V→1.8V and 1500V→16V→1.8V) confirm that the multi-stage circuit with an appropriate intermediate voltage delivers higher overall efficiency (**Figure R15**). The strategy demonstrates broad applicability, having also been successfully implemented in transformer-coupled circuits (**Note S14**).

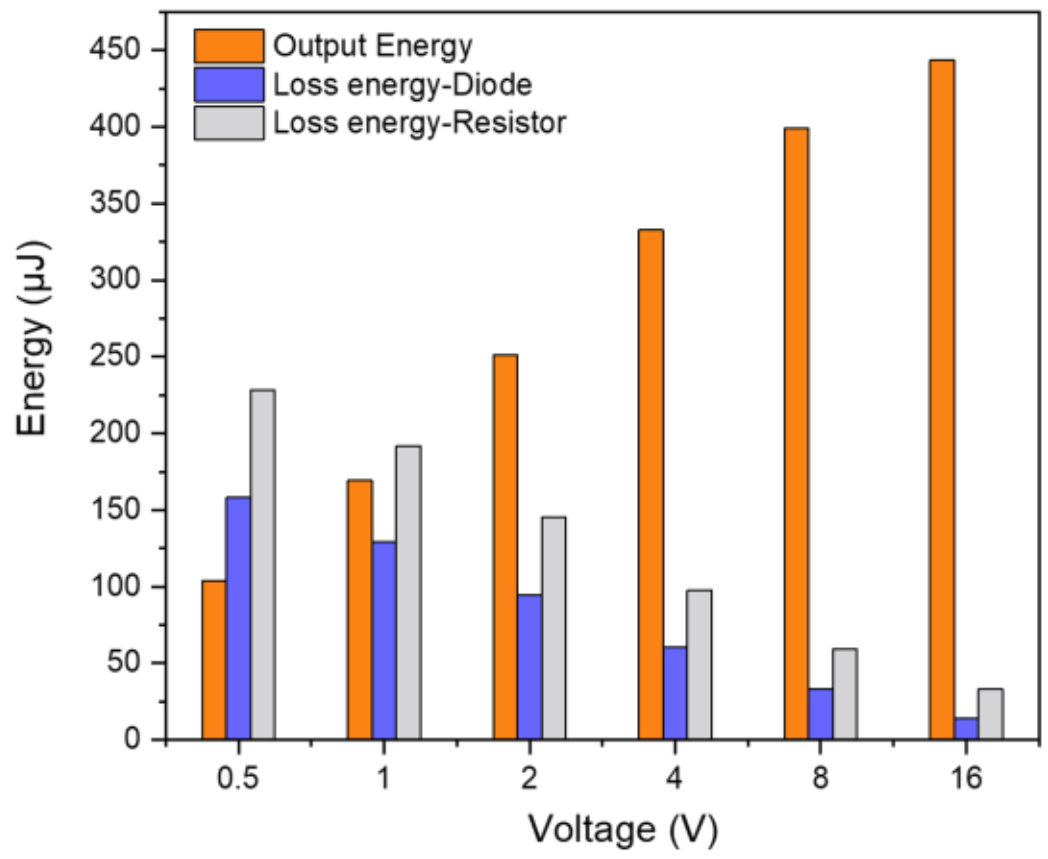

Figure R14 (**Revised Figure 3D**). The comparison of output energy and energy consumed by internal impedance as the output voltage gradually increases from 0.5 V to 5 V.

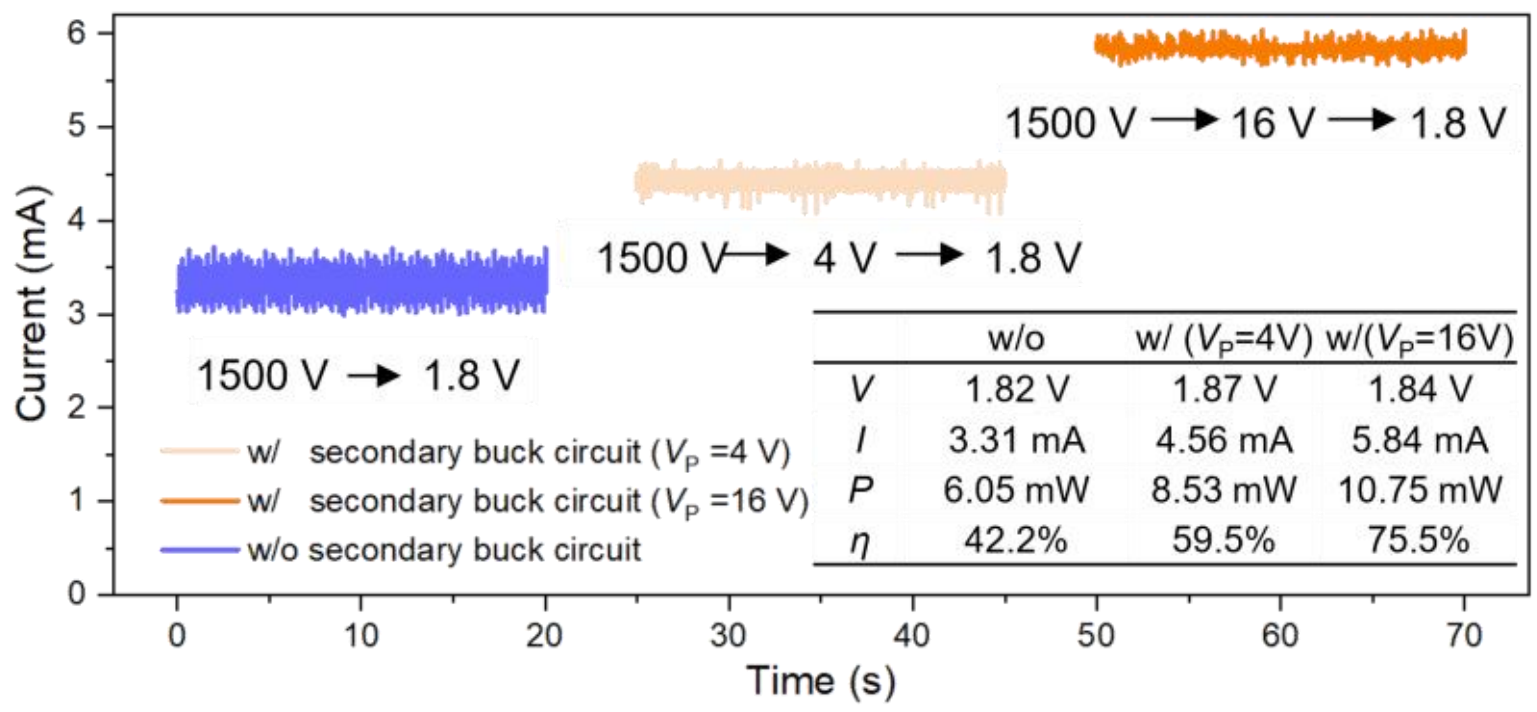

Figure R15. The comparison of output current with different step-down paths.

The key to addressing the second issue lies in adjusting the input voltage of the primary circuit to achieve voltage matching between the TENG and PMC. In this work, we employ the *Q-V* characteristic curve reported in our prior research (***Nat. Commun.*** **15**, 4167 (2024).). This curve reveals the TENG's output characteristics, which can used to guide circuit design to maximize the actual output power, a premise validated by the experimental results shown in **Figure R16 (**the *Q-V* curve was also discussed in **Comment 3** and **Note S6)**. An analysis of recently reported high-performance TENGs reveals that the working voltages of their employed PMCs do not exceed 800V (**Table R7**). However, the optimal operating voltages of the TENGs themselves in these studies are often in the range of several kilovolts. Consequently, a significant mismatch exists between the 800V switch rating and the TENGs' optimal high operating voltages. In summary, the efficiency drop of single-stage circuits at low voltages coupled with the circuit design's failure to match the high-performance of TENGs jointly leads to the observed low current density and low power density in these works.

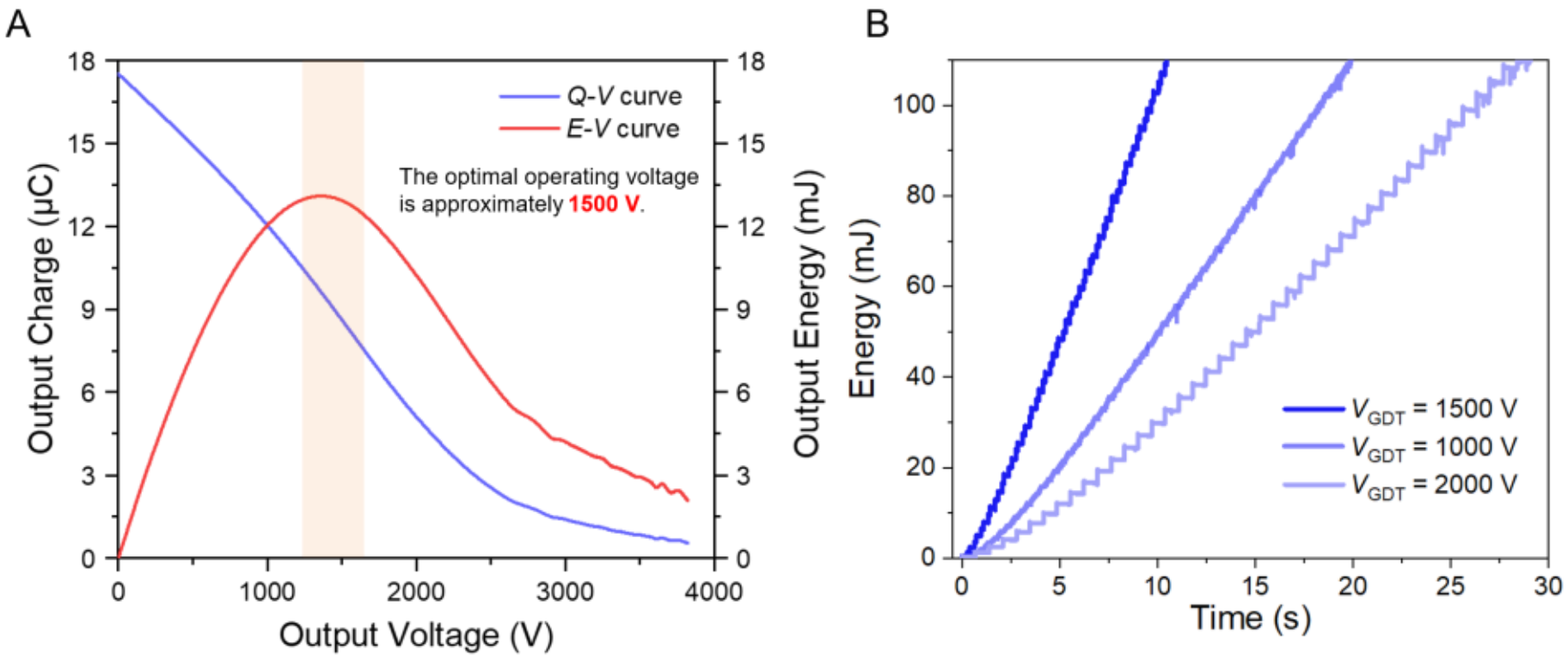

Figure R16. (A) The *Q-V* and *E-V* curves of the TENG. (B) Output energy of the TENG with the primary circuit at different gas discharge tube (GDT) switching voltages. Based on the curves in (A), the optimal GDT switching voltage is determined to be approximately 1500 V. This conclusion is corroborated by the experimental results in (B), which show that the output energy reaches its maximum when the GDT voltage is set to 1500 V.

In summary, this work establishes the design concept of a multi-stage circuit and clarifies the importance of regulating key voltage nodes for improving circuit efficiency and achieving efficient matching with high-performance TENGs. The reported performance and the production rate of electrochemical application both represent breakthrough progress in the field. This work can be regarded as a significant advancement in TENG field, whether viewed from the perspective of conceptual innovation, methodological design, or output performance.

To further contextualize these contributions, we compare the present works across five key dimensions: TENG-PMC Matching Mechanism, TENG-PMC Matching Method, Circuit Architectures, Optimization Strategies, and overall Performance (**Table R8**). Regarding the TENG-PMC matching mechanism, previous studies have primarily relied on the *V-Q* plot obtained from the CMEO theory, which assumes the surface charge density is stable as the voltage increase. Consequently, such methods are only suitable for devices with lower performance or those in which air breakdown can be completely suppresses by circuit design. Their applicability to high-performance devices is questionable, and employing these existing circuits often leads to significant performance degradation (**Table R7**). In contrast, our work is based on the energy cycle collapse theory and utilizes the *Q-V* curve to guide the design of the circuit's operating voltage. This approach is broadly applicable to most high-performance devices, thereby achieving substantially higher power density and current density. In terms of circuit optimization, prior works have focused more on component-level improvements while fundamentally neglecting the issue of efficiency degradation under low voltage. Our study not only identifies and explains this critical problem but also provides a feasible solution by regulating the intermediate voltage of the secondary circuit, ensuring high efficiency even under low-voltage conditions.

Table R8 (Added Table S10) Comparison of circuit design and optimization of different works

	Ref.12	Ref.19	Ref.24	Ref.11	Ref.17	Ref.23	Ref.13	Ref. 25	Ref. 10	This work
TENG-PMC Matching Mechanism	The *V*-*Q* plot based on ideal CMEO theory									The *Q*-*V* curve based on energy cycle collapse theory
TENG-PMC Matching Method	C_{in} extracts and stores energy			TENG's inherent capacitor directly serves as the storage unit.						Regulation the circuit operating voltage based on *Q*-*V* curve
Circuit Architectures	LC	FSCC	LC	LC	Transformer coupled circuit	LC	LC	LC & Commercial chips	Charge-pump circuit & LC	Multi-Stage Buck Strategy
Optimization Strategies	Utilizes SCR to achieve passive switching design	Fractional circuit to reduce voltage drop of diode	Optimized GDT	Optimized inductor	Optimized transformer	Double loops design	Optimized capacitor; Half-wave circuit completely suppresses air breakdown	Reduced charge and voltage losses; Achieved stable voltage output	Half-wave circuit completely suppresses air breakdown	Regulation the intermediate voltage of secondary circuit
Performance	1. Most optimal circuit efficiencies exceed 80%; however, efficiency significantly decreases below 5V.<br>2. The power density of the TENG used does not exceed 1 W/m^2. Whether it is suitable for high-performance TENG (power density exceeding 10W/m^2) is a question.									1. Efficiency remains around 80% even at low voltage.<br>2. Output power density exceeds 10 W/m^2.<br>3. Current density reaches up to 5.19 A m^{-2}

Revisions

The revised part in the present manuscript is as follows:

We have made a revision of "These strategies involve increasing the energy storage density of inductors, suppressing leakage currents in capacitors, and decreasing energy losses of switching elements. However, these efforts remain confined to confined to component-level optimization and have yet to recognize, let alone resolve, two systemic challenges inherent to the TENG-PMC interface (**Figure S1**, **Table S1** and **Note S1**)." in the second paragraph of the "**Introduction**".

We have made a revision of "Therefore, accurately understanding these issues and developing a power management paradigm that is compatible with the output characteristics of TENGs and maintains high efficiency in the low-voltage range has become an urgent problem to be solved." in the second paragraph of the "**Introduction**".

We have made a revision of "Comparison presented in **Table S10** and **Note S22** highlights that the energy cycle collapse theory adopted in this work differs fundamentally from the ideal CMEO theory commonly used in previous works, being particularly suitable for high-performance TENGs where air breakdown is difficult to avoid. The systematic nodal voltage regulation method within the proposed multi-stage buck strategy transcends conventional component-level circuit optimization, establishing a new paradigm for TENG power management design." in the first paragraph of "**Discussions**".

The revised part in the Supplementary Information is as follows:

We have revised the above discussion into the "**Note S1 Two Core Issues of TENG's PMC**", "**Note S2 The Energy Cycle Collapse and *Q–V* Characteristic Curve**", "**Note S3 Technical Advance of This Work**" and "**Note S22 Comparison of circuit design and optimization of different works**" in current Supplementary Information.

We have revised the "**Table S1 Performance of different high-performance TENGs**" and **"Table S10 Comparison of circuit design and optimization of different works"** in current Supplementary Information.

We have revised the "**Figure S1 Efficiency of different circuits in different works**" in current Supplementary Information.

2. The manuscript frequently cites “one order of magnitude improvement” but lacks a consistent baseline definition. Clarify which benchmark (voltage, power density, or current density) is used for this claim.

Response: We appreciate your professional review and reasonable suggestion. We have clarified and unified the claim of “one order of magnitude improvement” by specifying the following three performance benchmarks:

Current Density: The current densities achieved in this work are 5.17 A/m^2 at 1.8 V. This represents an improvement of nearly one order of magnitude compared to the best previously reported value of approximately 0.71 A/m^2.

Power Density: The power densities achieved in this work are 9.71 W/m^2 at 1.8 V. This represents an improvement of nearly one order of magnitude compared to the best previously reported value of approximately 1.41 W/m^2.

Hydrogen Production Rate: The TENG-driven electrochemical system exhibits a hydrogen production rate of 16.5 $mL/(min \cdot m^2)$, which is also nearly an order of magnitude higher than the best previously reported rate of 2.5 $mL/(min \cdot m^2)$.

Accordingly, we have revised the manuscript to explicitly include these baseline definitions, ensuring consistency and clarity throughout.

Revisions

The revised part in the present manuscript is as follows:

We have made a revision of “The strategy achieves approximately 80% circuit efficiency at the low-voltage range (90.9% in the primary circuit and 93% in the secondary circuit). Concurrently, the current and power density reach 5.19 A/m^2 and 9.71 W/m^2 at 1.8 V, which is nearly an order of magnitude improvement over best prior work.” in the “**Abstract**”

We have made a revision of “In the application of rotary mode TENG, we have achieved outstanding current density of 5.19 A/m^2 and power density of 9.71 W/m^2 at 1.8 V, which represents nearly an order of magnitude improvement compared to the best previously reported value of 0.71 A/m^2 and 1.41 W/m^2.” in the last paragraph of “**Introduction**”

We have made a revision of “In particular, the production rate of the self-power hydrogen generation system reaches an impressive 16.5 $mL/(min \cdot m^2)$, also marking a nearly order of magnitude higher than the prior best of 2.5 $mL/(min \cdot m^2)$” in the last paragraph of “**Introduction**”

We have made a revision of “When applied to the rotary-mode TENG, the system achieves a current density of 5.19 A/m^2 and power density of 9.77 W/m^2 at 1.8 V, which represents a nearly order of magnitude improvement over previously reported values of 0.71 $A/m^2$and 1.41 W/m^2. Moreover, both the energy system and the

electrochemical system constructed based on multi-stage buck strategy shown substantially enhanced efficiency. Integrated self-powered hydrogen systems exhibit 16.5 mL/(min·m^2) production rate, also representing a nearly order of magnitude increase compared to prior best of 2.5 mL/(min·m^2).” in the first paragraph of “**Discussions**”.

3. The use of the *Q*–*V* curve to determine optimal GDT threshold voltage (around 1.5 kV) is conceptually strong, but should include mathematical reasoning or modeling showing how energy extraction efficiency varies with switch threshold.

Response: We sincerely thank the reviewer for the professional evaluation and valuable suggestions.

Regarding the theoretical basis for determining the optimal GDT threshold voltage using the *Q*–*V* curve, we have conducted an analysis based on the "energy cycle collapse" theory, as detailed below.

1. Theoretical Background: Energy Cycle Collapse

In high-performance TENGs, as the output voltage increases, the strong electric field at the electrode edges can induce air breakdown, leading to decline of the surface charge density (σ) on the triboelectric layer. This phenomenon causes the energy cycle of the TENG to "collapse", preventing it from reaching the ideal curve. The *Q*–*V* curve is a key tool for describing this practical output characteristic.

On the contrary, if the surface charge density remains stable while the output voltage rises, the energy cycle can achieve the theoretically ideal state. We name this phenomenon is energy cycle stable state.

2. *Q*–*V* Characteristic Curve and the Optimal Output Energy

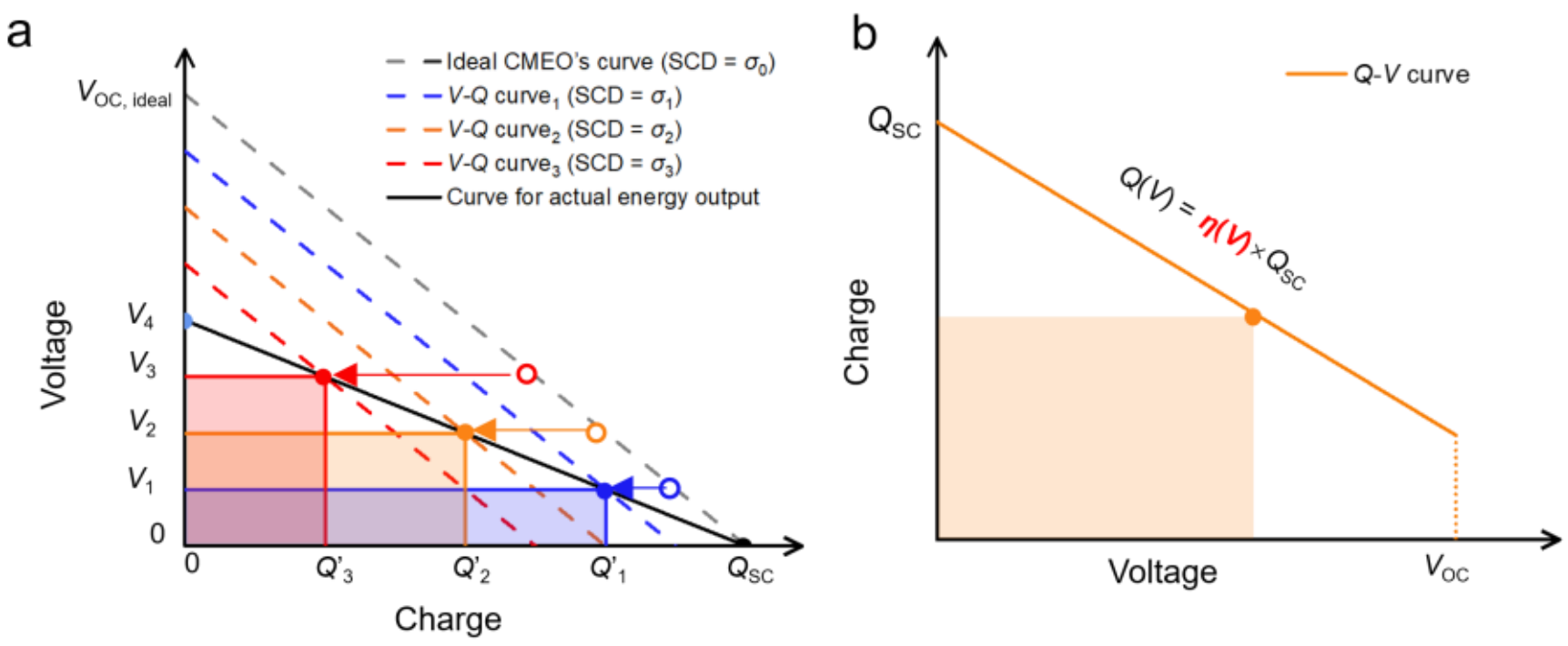

Figure R17 (Revised Figure S2). The collapsed state of the energy cycle and the *Q-V* output characteristics curve of TENG. (a) The collapsed state of the energy cycle. The $V_{oc,\ ideal}$ is the ideal open-circuit voltage. It can be calculated by the short-circuit output charge (Q_{sc}) divided by inherent capacitor of TENG. As the output voltage gradually increases from 0 to V_3, the surface charge density gradually decreases from σ_0 to σ_3. The energy cycle gradually collapses, and the slope of the curve (dashed line) remains unchanged. The corresponding output charge and output energy decrease. (b) The *Q-V* output characteristics curve of TENG. Coulombic efficiency ($\eta(V)$) defined as the ratio of the output charge to the short-circuit charge at a given output voltage. V_{oc} is the actual open-circuit voltage. When the output voltage of the TENG reaches V_{oc}, the voltage stops rising due to breakdown occurs inside the device or the output charge drops to zero.

Under ideal conditions (without air breakdown), the TENG output is determined by a fixed charge density σ_0 and the inherent capacitance (C_T). This stable energy cycle state (surface charge density of TENG remains stable) indicates that in power management design, using the TENG's own C_T in place of the PMC's input capacitance

(C_{in}) can achieve optimal energy transfer. This point has been well-established in previous works[1,2].

However, air breakdown causes σ to decrease dynamically with increasing voltage, producing a series of actual *V*–*Q* curves that vary with the output voltage (**Figure R17**). Connecting the actual operating points at different voltages yields the "actual energy-output curve". Swapping the horizontal and vertical coordinates of this curve gives the *Q*–*V* output characteristic curve, which directly reflects the amount of charge that the TENG can actually deliver at a given output voltage.

In our previous work, we defined the Coulombic efficiency as the ratio of the actual output charge to the short-circuit charge[3]. Theoretical analysis shows that the energy output of the TENG reaches maximum when the Coulombic efficiency is 50%.

Therefore, in this work we propose that the operating voltage corresponding to this efficiency on the *Q*–*V* curve represents the theoretical optimal voltage for achieving the best energy match between the TENG and PMC.

3. Verification

To verify the above conclusion, we start from the energy cycle of the TENG and derive the matching process with a capacitive load (the PMC input capacitance C_{in}).

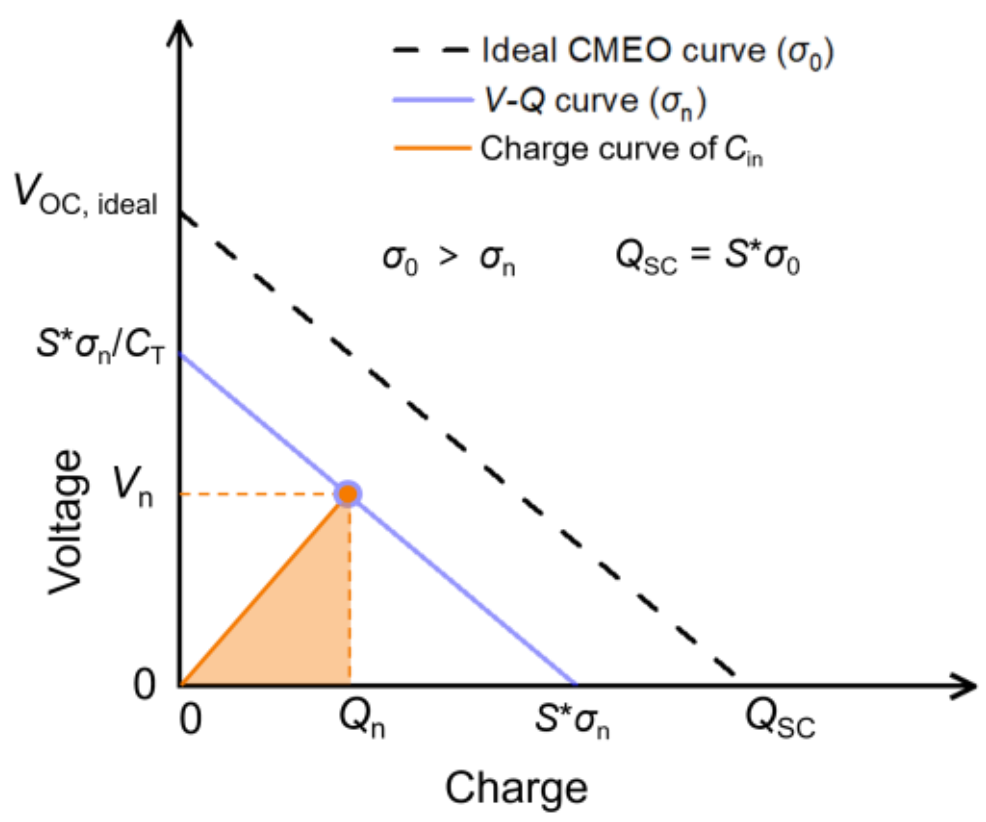

Figure R18 (Added Figure S6). Energy storage process of the capacitor during TENG energy cycle collapse. Assuming the capacitor is ultimately charged to a voltage V_n, its charging curve will intersect the actual energy cycle curve of the TENG corresponding to that voltage V_n. At this point, due to air breakdown, the TENG's surface charge density has decayed to σ_n. The orange shaded area in the figure represents the energy stored in the capacitor. This area is maximized when the TENG's actual output charge Q_n is half of its short-circuit charge Q_{SC}.

When the C_{in} is charged to a voltage V_n, the surface charge density of the TENG decays to σ_n. The energy (E) stored in the capacitor is then:

$$E = \frac{1}{2} C_{in} V_n^2$$

Intersection point of two curves in **Figure R18** simultaneously satisfies the intrinsic *V*–*Q* equation of the TENG

and the capacitor characteristic:

$$V_n = \frac{\sigma_n S}{C_T} - \frac{Q_n}{C_T}$$

$$V_n = \frac{Q_n}{C_{\text{in}}}$$

Where S is the equivalent area of TENG, Q_n is the output charge of TENG.

Combining above three equations yields:

$$E = \frac{1}{2}(\sigma_n S - C_T V_n)V_n$$

The key point is that the surface charge density σ_n is a function of the voltage V_n. Our earlier work[3] has demonstrated that, within a certain voltage range, the two follow a linear decay relationship:

$$\sigma_n = \sigma_0 - kV_n \quad (k \text{ is a constant})$$

Substituting this relation into the energy expression and maximizing it, we obtain the optimal operating condition:

$$Q_n = \frac{\sigma_0 S}{2}$$

At this point, the actual output charge Q_n is exactly half of the ideal short-circuit charge Q_{sc}, and the Coulombic efficiency is 50%. This validates the correctness of the conclusions we have drawn above.

It is worth noting that the operating voltage selected in this work corresponds to a Coulombic efficiency on the $Q-V$ curve of approximately 50%, which aligns well with the results discussed above.

Revisions

The revised part in the present manuscript is as follows:

We have made a revision of "Herein, we utilize the Q-V characteristic curve of the TENG to assist in regulating the nodal voltage V_N, so as to ensure that the primary buck circuit achieves optimal matching with the TENG[41]. The rationale of this method was substantiated by a derivation based on the energy cycle collapse process of the TENG (**Note S6** and **Figure S6**). Furthermore, the validity of this method has been confirmed by experimental results presented in subsequent experiments." in the first paragraph of "**Regulation of input voltage for primary buck circuit**"

The revised part in the present Supplementary Information is as follows:

We have revised the above discussion into the "**Note S2 The Energy Cycle Collapse and *Q*–*V* Characteristic Curve**" and "**Note S6 Theoretical verification of *Q*-*V* curve guidance for optimal operating voltage of PMC**"

in current Supplementary Information.

We have revised the "**Figure S2. The collapsed state of the energy cycle and the *Q-V* output characteristics curve of TENG.**" and "**Figure S6. Energy storage process of the capacitor during TENG energy cycle collapse.**" in current Supplementary Information.

4. Equations (1)–(10) should be supported with circuit simulation validation (LT-SPICE waveforms, efficiency plots). Some terms such as $Imax$, Pin, and $Rint$ are not clearly defined with units and measured values.

Response: Thank you very much for your careful review and suggestion.

The equivalent circuit for the LC circuit is shown in **Figure R19A**, and the simulation schematic is shown in **Figure R19B**. In the simulation, a voltage-controlled switch is used to replace the GDT for circuit control. The parameters are as follows: input capacitance (C_{in}) 440 pF, output capacitance (C_{out}) 10 mF, inductance (L) 1 mH, switch threshold voltage 1500 V. The circuit internal resistance (R_{int}, mainly from the inductor) is 1.82 Ω, and the freewheeling diode forward voltage drop (V_{diode}) is 0.83 V (increasing the number of diodes to improve voltage drop). Although a precise GDT switch model was not established and the circuit is idealized, this model still effectively analyzes the impact of V_{diode} and R_{int} on energy loss. **Figures R19C-G** present key waveforms for one operating cycle. Specifically, they show the circuit current, the voltage across the internal resistance, the diode voltage, the input voltage, and the output voltage.

Equation (1) calculates the maximum current (I_{max}) in the circuit:

$$I_{max} = V_{in}\sqrt{\frac{C_{out}C_{in}}{L(C_{in}+C_{out})}}$$

By substituting the circuit parameters into equation (1), we obtained/caculated an I_{max} of 0.995A, which is consistent with the simulation results (**Figure R19C**).

Equation (2) indicates that both the diode conduction current (I_{diode}) and the inductor saturation current (I_{sat}) should be no less than the I_{max}. This equation guides practical component selection and does not require simulation validation.

Equations (3) and (4) are used to calculate the actual input power and the primary circuit efficiency, respectively. They are also do not rely on simulation verification.

Equations (5) and (6) describe the input energy (E_{in}) distribution of C_{in}. The input energy is mainly dissipated in three parts: diode loss (E_{diode}), internal resistance loss (E_R), and energy stored in the output capacitor (E_{out}):

$$E_{in}=E_{diode}+E_R+E_{out}$$

$$P_{in}=P_{diode}+P_R+P_{out}=I(t)*(V_{diode}+V_R+V_{out})$$

Calculations show that the input energy is approximately 495 μJ. In the simulation, the sum of the three parts is about 490 μJ (**Figure R19D**), verifying the correctness of Equations (5) and (6).

Equation (7) describes the current waveform during the second stage of the LC circuit:

$$I(t) = \left(I_{max} + \frac{V_{diode}+V_{out}}{R_{int}}\right) e^{-\frac{R_{int}}{L}t} - \frac{V_{diode}+V_{out}}{R_{int}}$$

This equation is primarily used to derive Equation (8), the expression for current duration:

$$T=-\frac{L}{R_{int}}\ln\left(\frac{V_{out}+V_{diode}}{R_{int}I_{max}+V_{out}+V_{diode}}\right)$$

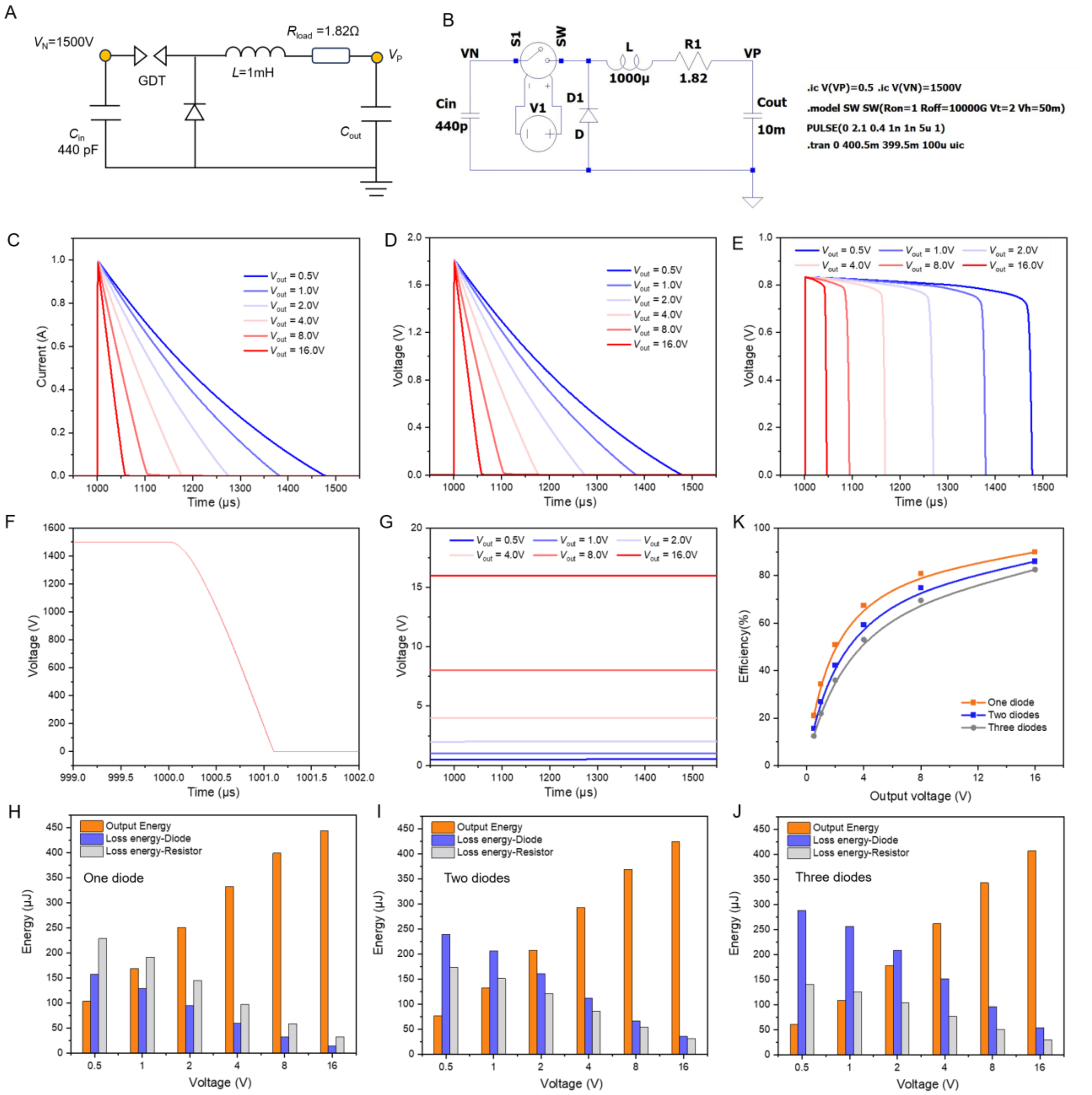

Figure R19 (Added Figure S16). The circuit simulation results. (A) The equivalent circuit of LC circuit; (B) The circuit simulation parameters; (C) The waveform of current; (D) The waveform of voltage across the internal resistance; (E) The waveform of diode voltage; (F) The voltage of input capacitor; (G) The voltage of output capacitor; (H) The loss energy of resistor and one diode and the output energy. V_{diode} is about 0.83 V. (D) The loss energy of resistor and two diodes and the output energy. V_{diode} is about 1.66 V. (E) The loss energy of resistor and three diodes and the output energy. V_{diode} is about 2.49 V. The input energy is 495 μJ.

Equation (7) and (8) shows that as the output voltage increases, the current duration gradually decreases, while the current peak remains essentially constant. Substituting circuit parameters into equation (8), as the output

voltage increases from 0.5 V to 16 V, the calculated current durations are 473 μs, 379 μs, 272 μs, 175 μs, 103 μs, and 56 μs, respectively, with the maximum current remaining nearly unchanged. These results match the simulation (**Figure R19E**).

Equations (9) and (10) describe the energy dissipation on the internal resistance and the diode, both related to the current duration. Since the current waveform shape and peak remain largely unchanged, an increase in output voltage only shortens the duration (**Figure R19D and Figure R19E**), thereby affecting these losses.

Revisions

The revised part in the present manuscript is as follows:

We have revised the **Figure 3B-D**.

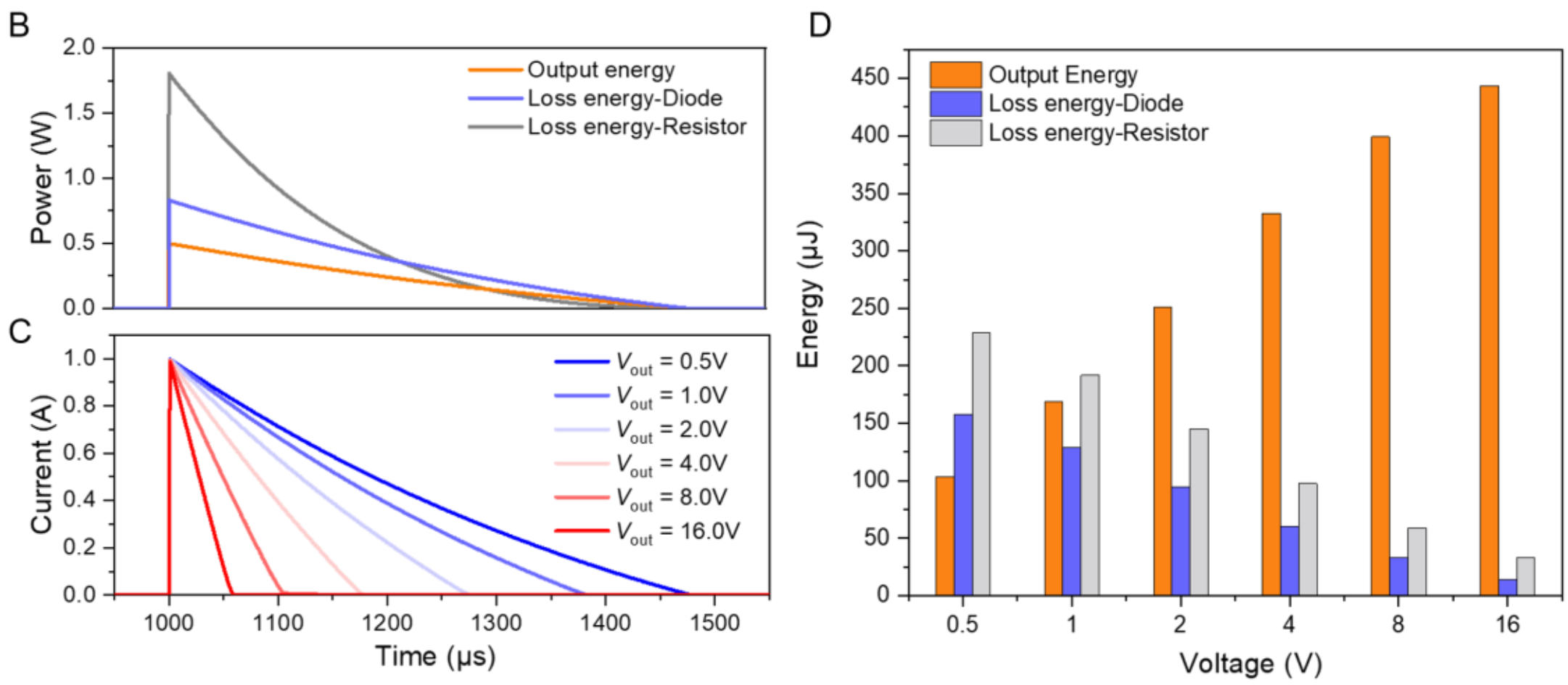

Figure 3. (B) The output energy and energy consumed by internal impedance when the output voltage is 0.5 V. (C) The current of the primary buck circuit as the output voltage gradually increases from 0.5 V to 16 V. (D) The comparison of output energy and energy consumed by internal impedance as the output voltage gradually increases from 0.5 V to 16 V.

We have revised the "At this moment, the freewheeling diode conducts to provide a path for the inductor's current to continue flowing (**Figure 3A <ii>**, and the detailed analysis is shown in **Note S10**.). Our simulation analysis revealed that the primary cause of the LC circuit's efficiency degradation is the significant increase in energy loss during this process at low voltages (**Figure S16** and **Note S13**)." in the first paragraph of the part of "**The reasons for low efficiency in primary circuits**"

We have revised the "the circuit efficiency of the LC circuit also gradually improves accordingly (**Figure S16K**). This trend is consistent with the experimental results shown in **Figure 2M**." in the penultimate paragraph of the part of "**The reasons for low efficiency in primary circuits**"

The revised part in the present Supplementary Information is as follows:

We have revised and merged the original **Figure S13, Figure S15** and **Figure S15** into **Figure S16.**

We have consolidated the previous "**Note 8 The specific working process of LC circuit**" and "**Note 12 The calculation process for current of LC circuit**" into a single "**Note 10 The calculation process for LC circuit**".

We have consolidated the previous "**Note S11 The influence of the number of freewheeling diodes on the multi-stage buck circuit.**" and "**Note S13 The impact of increasing output voltage on energy loss**" into a single "**Note S13 The simulation for LC circuit**" in the present supplementary information, and the above discussion has been incorporated into **Note S13**.

Response: We have revised all unclear definitions of abbreviations in the manuscript for clarity. The calculation of P_{in} is illustrated in **Figure S14**. Once the capacitance, inductor and input voltage of the LC circuit are determined, I_{max} remains essentially constant. R_{in} is primarily determined by the internal resistance of the inductor, which is 1.82 Ω as provided in the response to **Comment 11**.

Revisions

We have revised the "Furthermore, the results of simulation calculations and experiments reveal that increasing the operating voltage of the secondary buck circuit can subsequently reduce the energy wastage in the form of joule heating caused by V_{diode} (voltage drop of diode) and R_{int} (internal resistance in the circuit)," in the last paragraph of "**Introduction**"

We have revised the "Secondly, the conduction current of the freewheeling diode must be greater than the maximum current (I_{max}) during the LC circuit's working process." in the second paragraph of "**Regulation of input voltage for primary buck circuit**"

We have revised the "Where P_{in} and P_{out} represent the input and output power of LC circuit, P_R and P_{diode} represent the power loss on internal resistance and diode of LC circuit," in the first paragraph of "**The reasons for low efficiency in primary circuits**"

5. The reported 80–90% circuit efficiency is remarkably high for TENG-driven systems. Please provide calibration details, measurement uncertainties, and comparison under identical load conditions with other PMCs.

Response: We appreciate the reviewer's professional assessment and valuable suggestions.

Efficiency Analysis: As discussed in response to your **Comment #1**, the high efficiency is achieved by precisely regulating the key node voltage (the intermediate voltage) within the multi-stage circuit. Our experimental results show that when maintaining a final output voltage of 1.8V, the overall circuit efficiency drops to approximately 42.2% and 59.5%, respectively, under two suboptimal conditions: 1) not using secondary circuit (single LC) design, and 2) employing a multi-stage design but with the intermediate voltage set to 4V (**Figure R20**). These drops are primarily due to the low output voltage of the primary (LC) circuit, which increases energy losses. In contrast, regulating the intermediate voltage to 16V significantly improves the primary circuit's efficiency, raising the overall system efficiency to 75.5%. This strongly validates the critical importance of intermediate voltage optimization.

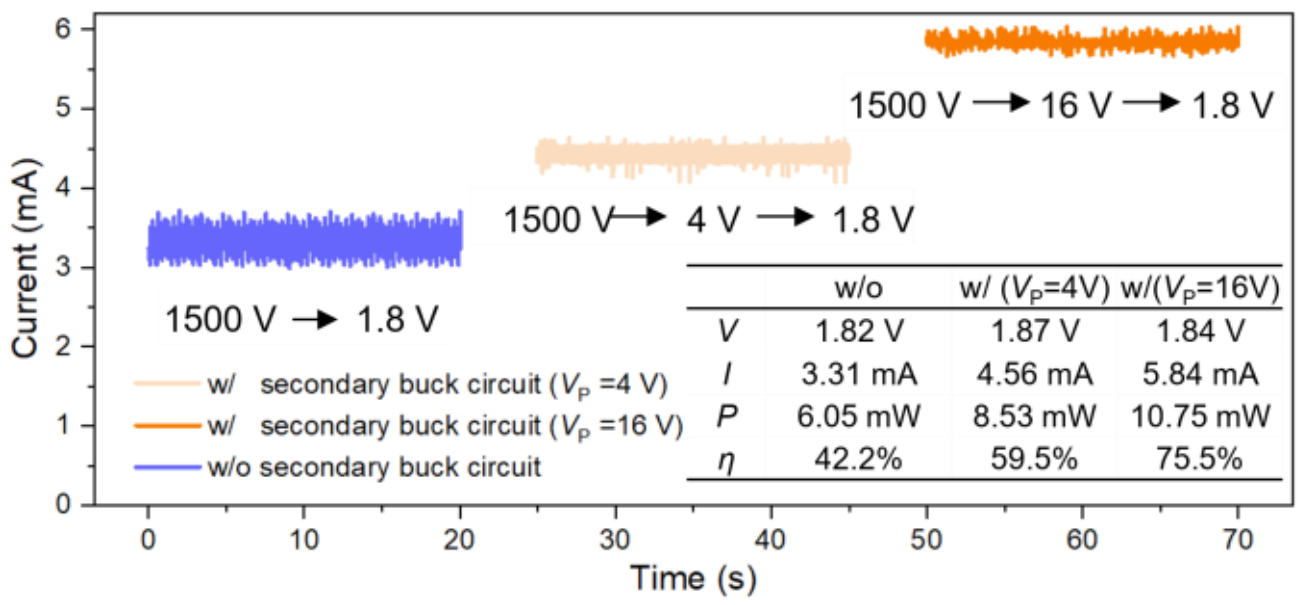

Figure R20. The comparison of output current with different step-down paths.

Regulation Method: The intermediate voltage is regulated by switching the secondary circuit chips, which have different preset input voltages. In this work, we used the LTC-3588-1 (preset 4V) and LTC-3588-2 (preset 16V) to construct the secondary circuit.

Measurement Method: The output current and voltage of both the LC circuit and the multi-stage circuit were measured by varying the resistance at the output, and the output power was calculated by current, voltage and resistance. For the multi-stage circuit tests, the load resistance was carefully adjusted to ensure stable input and output voltages for the secondary circuit (**Figure R21**), guaranteeing the accuracy of the measurements.

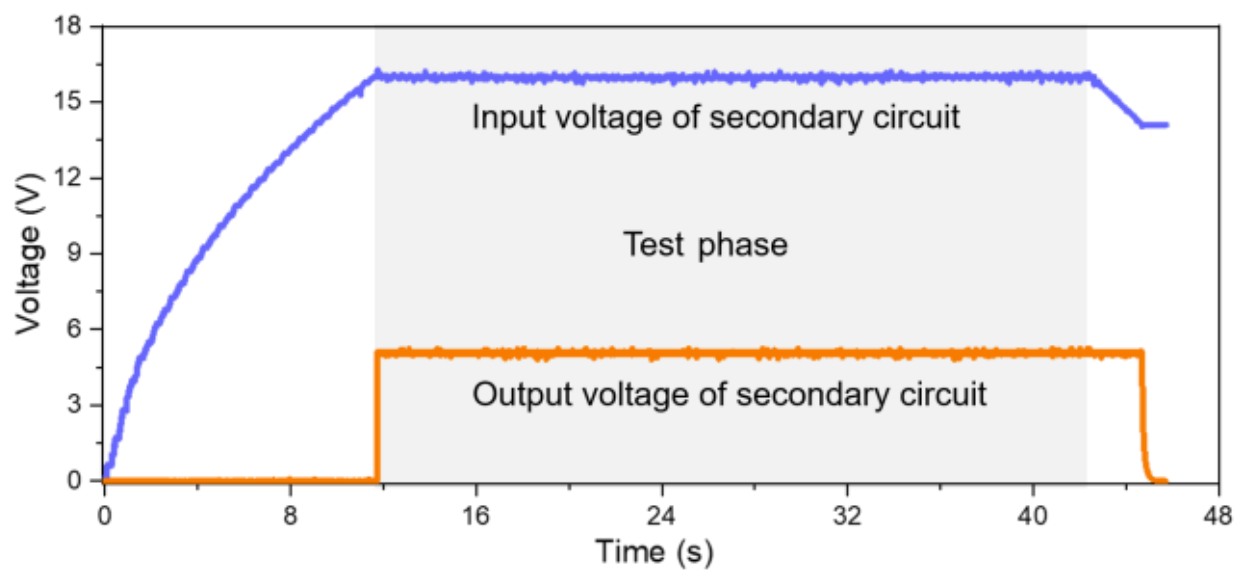

Figure R21. The input voltage and output voltage of the secondary circuit.

Comparative Summary: A new **Table R9** has been added to the manuscript, comparing the performance of our strategy at output voltages of 1.8V, 3.6V, and 5.0V against conventional PMCs (including LC circuits, transformer-based solutions, and FSCC). It should be noted that many existing studies do not explicitly report efficiency with statistical details under these voltages. Therefore, the data for these works presented in the table were extracted from the performance curves or values reported in the respective works.

Table R9 (Revised Table S3) The reported efficiency in different works*

REF	Circuit	η (1.8 V)	η (3.6 V)	η (5.0 V)	Optimal efficiency
11	LC	32.1%	45.0%	51.1%	81.6%
12	LC	51.1%	67.8%	73.6%	89.8%
17	Transformer	42.6%	55.8%	60.3%	78.5%
19	FSCC	35.2%	62.5%	84.7%	94.5%
23	LC	18.3%	37.8%	48.1%	90.6%
24	LC	36.3%	50.4%	55.2%	83.9%
This Work	Multi-stage buck circuit	75.5%	86.3%	85.1%	90.9%

*The data for these works presented in the table were extracted from the performance curves or values reported in the respective references.

Revisions

The revised part in the present manuscript is as follows:

We have added the "The output current and charge of TENG were measured using an electrostatic electrometer (Keithley 6514). The output current and voltage of both the LC circuit and the multi-stage circuit were measured by varying the resistance at the output, and the output power was calculated by current, voltage and resistance. For the multi-stage circuit tests, the load resistance was carefully adjusted to ensure stable input and output voltages for the secondary circuit (**Figure 3F and Figure S22**), guaranteeing the accuracy of the measurements." in the part of "**Method**"

The revised part in the present supplementary information is as follows:

We have revised the **Table S3** in the present supplementary information.

6. The demonstration powering 16 sensors and a mobile phone is compelling, but quantitative stability (voltage ripple, current stability) and endurance under continuous operation should be discussed.

Response: We appreciate your professional and valuable comments.

A low output voltage ripple is crucial for the stable operation of electronic devices. This is well achieved in our design. From an energy perspective, when the TENG delivers an average input power P_{in} at a switching frequency f, the energy transferred per cycle of circuit (E) that is available at the output satisfies:

$$E = \eta \cdot \frac{P_{\text{in}}}{f} = \frac{1}{2} C_{\text{out}} \left(V_{\text{max}}^2 - V_{\text{min}}^2 \right)$$

Where C_{out} is the output capacitance, and V_{max} and V_{min} are the peak and valley voltages, respectively. This relationship shows that for a constant input power, increasing the operating frequency f is an effective way to reduce the voltage ripple (V_{max} - V_{min}). Here, the secondary circuit (LTC-3588) operates at a high switching frequency, which ensures that the final output voltage ripple is suppressed to merely tens of millivolts (**Figure R22A and Figure R22B**).

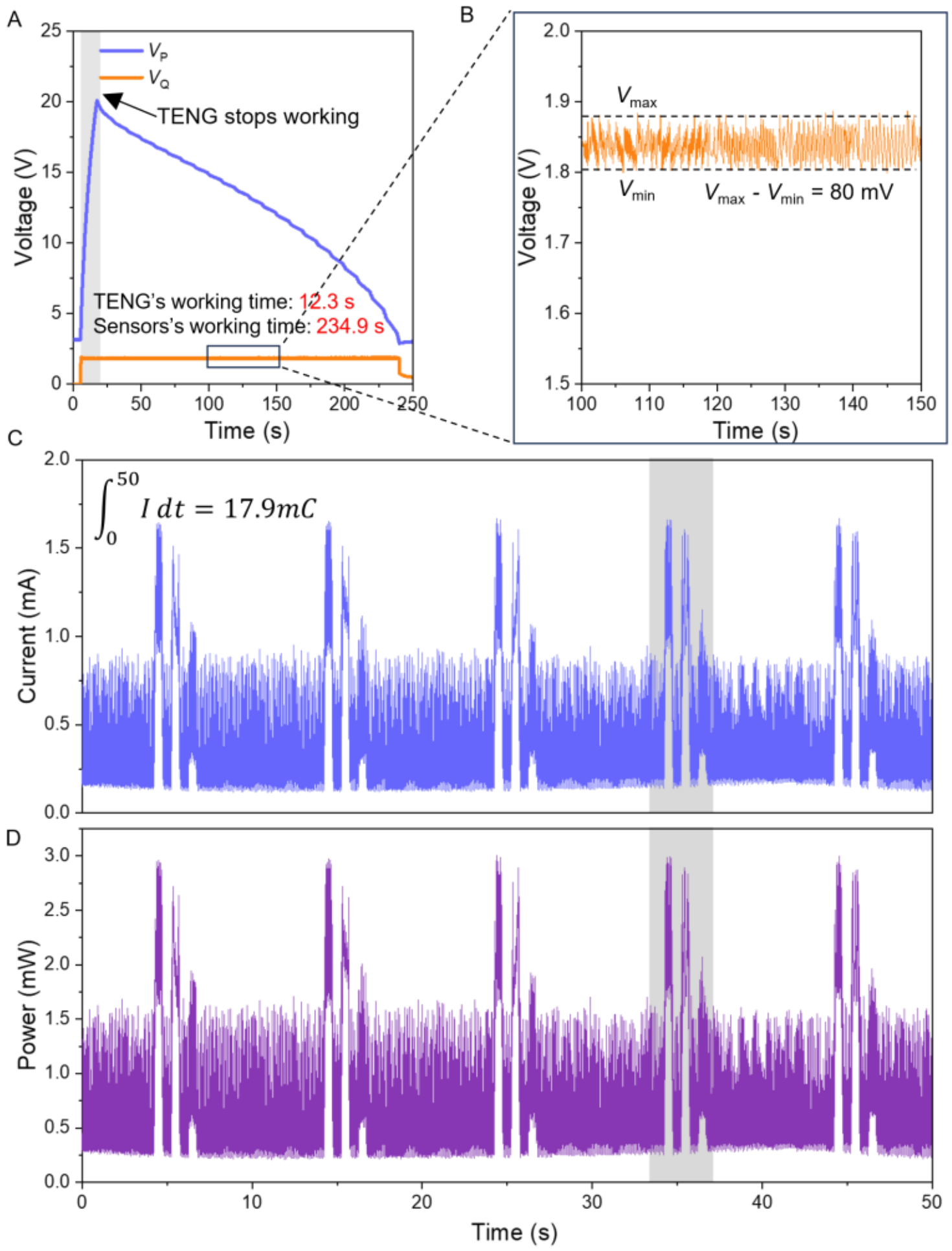

Figure R22 (revised Figure S32). The analysis of voltage ripple for 16 temperature and humidity sensors. (A) Primary and secondary circuit output voltage, (B) secondary circuit output voltage ripple (80 mV), (C) operating current, and (d) operating power of sensors.

Additionally, in a conventional single-stage circuit, intermittent high-current and high-power events (such as the periodic data acquisition and transmission by a hygrothermograph, **Figure R22C and Figure R22D**) can cause significant voltage fluctuations at the output. In our design, the first circuit (LC circuit) with its large storage capacitor (1 mF) acts as a stable energy buffer, and the secondary circuit (LTC-3588) provides high-bandwidth voltage regulation. These ensuring that the final output voltage remains stable and unaffected by backend power surges.

Furthermore, the long-term stability of the system under continuous operation is shown in **Figure R23.** Both the intermediate voltage (the output voltage of primary circuit) and the final output voltage/current of secondary circuit remain stable over 30,000 operating cycles, confirming the robustness and endurance of our design.

In summary, the combination of a high-frequency circuit and a multi-stage design with sufficient energy buffering effectively minimizes output voltage ripple and ensures stable power delivery under dynamic loads.

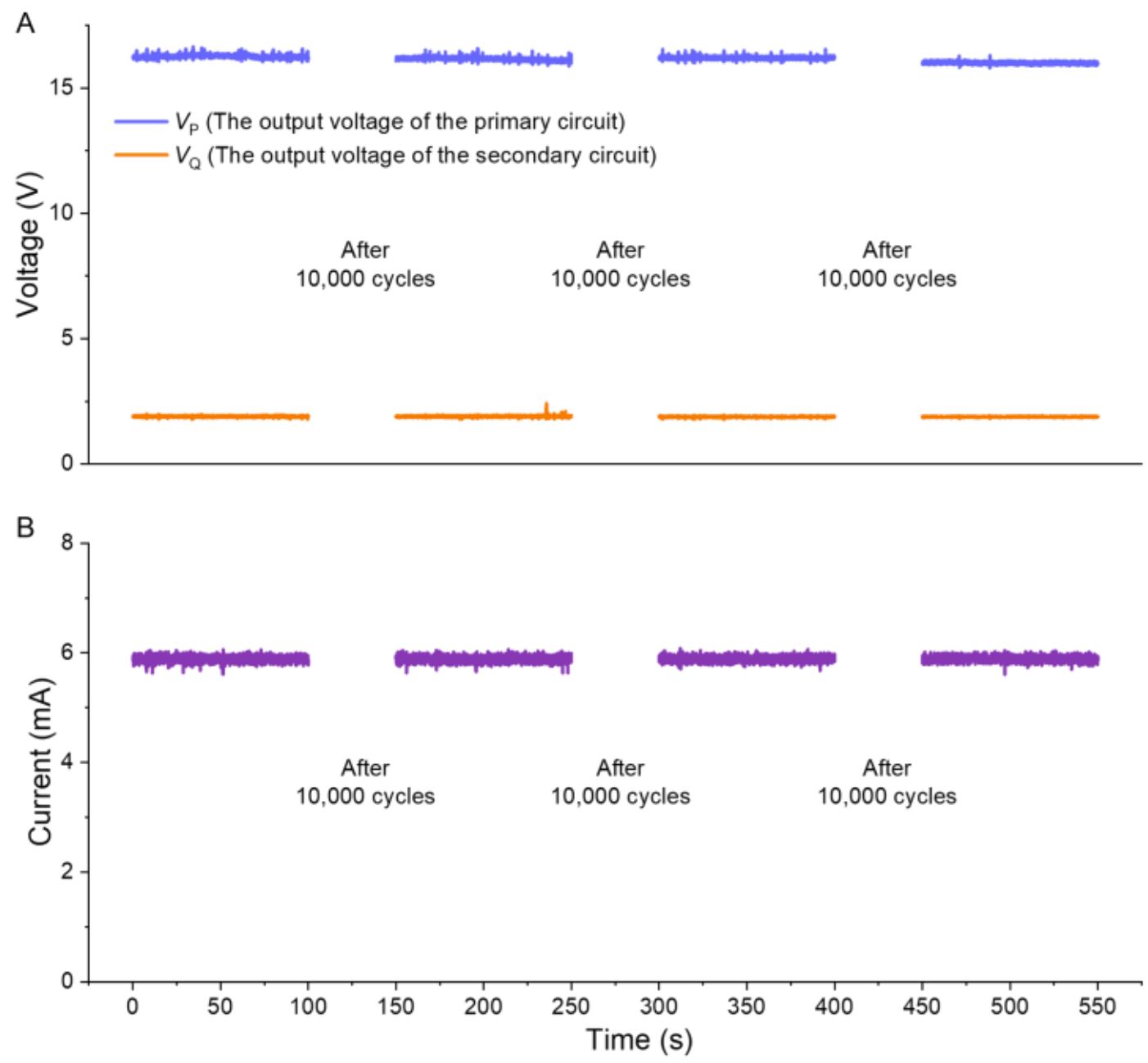

Figure R23 (Revised Figure S24). The circuit stability testing experiments. (A) The output voltage of primary circuit and secondary circuit after 30,000 cycles. (B) The output current after 30,000 cycles.

Revisions

The revised part in the present manuscript is as follows:

We have made a revision of the "This demonstrates that the secondary circuit provides energy storage and regulation functions, specifically utilizing high-frequency switching to achieve a low voltage ripple (**Figure S32** and **Note S18**), thereby enabling TENGs to deliver a stable and continuous power supply to electronic devices despite fluctuating energy input[32]." in the second paragraph of "**The multi-stage buck strategy for self-power systems**"

The revised part in the present supplementary information is as follows:

We have revised the **Figure S24** and **Figure S32** in the present supplementary information.

We have revised the above discussion into the "**Note S18 Analysis of output voltage ripple and stability in multi-stage buck circuit**" in current Supplementary Information.

7. The hydrogen production rate of 16.5 mL/(min·m²) should be compared against theoretical Faradaic limits to assess electrochemical efficiency. Include more context on catalyst material, electrode configuration, and electrolyte concentration. Also, it would strengthen the study to present the energy conversion efficiency of the electrolysis system.

Response: We sincerely thank the reviewer for the careful evaluation and constructive suggestions.

In this work, hydrogen production was measured using the volumetric displacement method. The anode is a ruthenium iridium titanium electrode, and the cathode is a graphite electrode. The concentration of the NaCl solution used is 1 mol/L. No catalysts were used in this work. At the request of **Reviewer 1**, the following five repeated tests were performed to ensure the reliability of the experimental results. The time was recorded at every 25 μL increment based on the graduated cylinder scale, with 100 μL constituting one measurement cycle. Five independent tests were performed. The results are summarized in the **Table R10** below.

Table R10 (Added Table S6) The production rate of hydrogen and faradaic efficiency

	The 1st cycle					The 2nd cycle					The 3rd cycle				
Time (s)	0	18	39	64	86	0	25	46	75	84	0	21	42	68	84
Yield (μL)	0	25	50	75	100	0	25	50	75	100	0	25	50	75	100
Production rate (μL/min)	69.77					71.4					71.4				
Faradaic efficiency	98.46%					96.27%					95.29%				
	The 4th cycle					The 5th cycle									
Time (s)	0	17	35	55	80	0	22	43	60	81					
Yield (μL)	0	25	50	75	100	0	25	50	75	100					
Production rate (μL/min)	75.00					74.07									
Faradaic efficiency	98.08%					97.63%									

The average hydrogen production rate was determined to be 72.33 μL min^{-1} (**SATP**, 2.92**μmol/min**), which corresponds to 16.5 mL/(min·m^2) (calculated by the effective working area of the TENG). The average faraday efficiency measured is 97.15%. The corresponding current data are presented in the **Figure R24**.

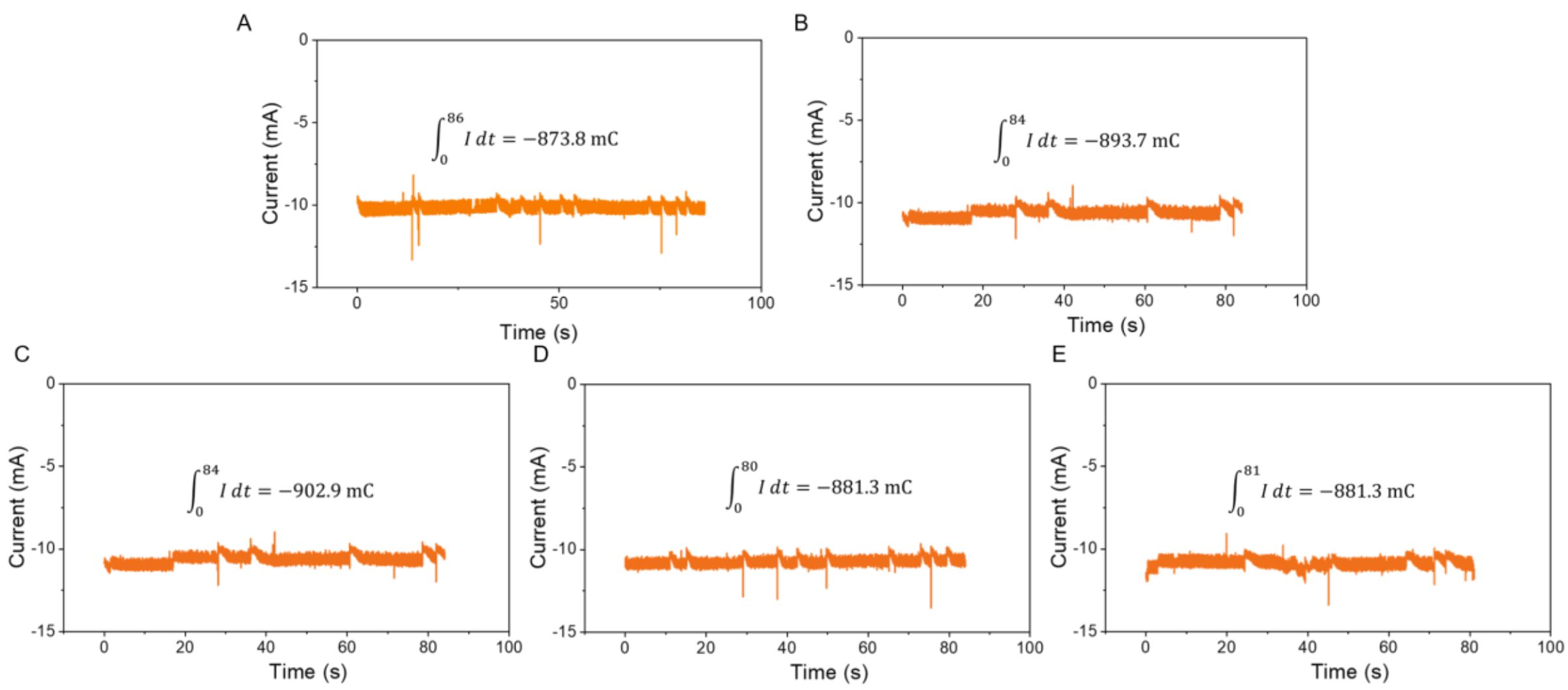

Figure R24. The operating current of the self-power hydrogen production system. (A)-(E) Test current from the first to the fifth time.

In the hydrogen production process studied here, the cathode reaction is:

$2H_2O + 2e^- \rightarrow H_2\uparrow + 2OH^-$

The anode may undergo two reactions:

1. $2Cl^- - 2e^- \rightarrow Cl_2$

   $Cl_2 + 2OH^- \rightarrow Cl^- + ClO^- + H_2O$
2. $2H_2O \rightarrow O_2 + 4H^+ + 4e^-$

To identify the dominant anode pathway, we quantified ClO^- production using ultraviolet-visible (UV-Vis) absorption spectroscopy. The principle is as follows: under acidic conditions, ClO^- oxidizes excess iodide (I^-) to iodine (I_2), which further combines with I^- to form triiodide (I_3^-). I_3^- exhibits a characteristic absorption peak in the UV region (288 nm, shown in **Figure R25A)**. Its absorbance is proportional to the I_3^- concentration, allowing ClO^- concentration to be determined via a calibration curve (**Figure R25B**).

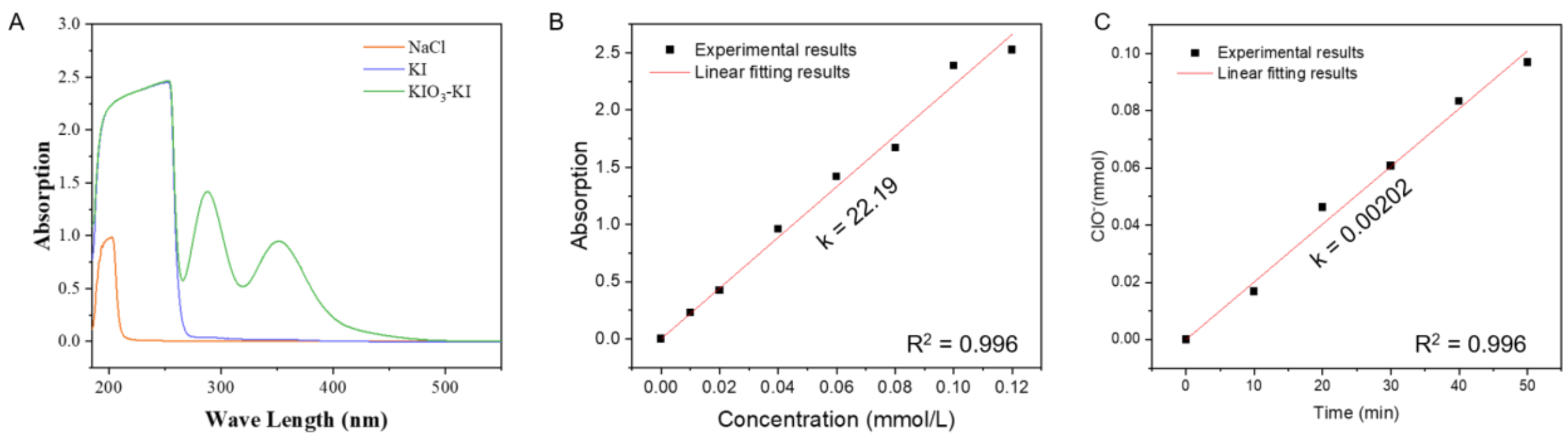

Figure R25 (Added Figure S37) UV-Vis spectra test. (A) UV-Vis spectra of NaCl, KI and KIO_3-KI solution and (B) the standard curve of KI solution for calibrating ClO^- concentration. (C) ClO^- production rate.

During the experiment, a 1 mL sample was taken from solution every 10 min and treated sequentially with:

- 0.1 mol/L KI (providing excess I^-)
- 1 mol/L H_2SO_4 and 0.5 mol/L KOH were used to adjusted PH to about 3.5.
- The mixture was diluted to 25 mL, reacted for 10 min, and then analyzed by UV-Vis.

The measured ClO^- production rate was 2.02 μmol/min (**Figure R25C**). Based on the stoichiometry of the reaction between anodically generated Cl_2 and OH^- ($Cl_2 + 2OH^- \rightarrow Cl^- + ClO^- + H_2O$), this rate is directly equivalent to the rate of Cl_2 generation that subsequently reacted to form ClO^-. This indicates that at least 69.2% of the anode current was utilized for Cl_2 generation that contributed to the measured ClO^- (H_2 production rate is 2.92 μmol/min).

Table R11 (Added Table S8) The production rate of anode gas

	The 1st cycle	The 2nd cycle	The 3rd cycle	The 4th cycle	The 5th cycle
Yield (μL)	125	125	125	125	125
Time (s)	978	1052	947	1043	1035
Production rate (μL/min)	7.67	7.13	7.92	7.19	7.24

Gas collected from the anode via water displacement yielded a much lower rate of 7.43 μL/min (≈0.30 μmol/min at **SATP**) (**Table R11**). Given the high aqueous solubility of Cl_2, this collected gas is primarily composed of O_2. This gas collection data suggests that approximately **20.5% of the anode current** was directed toward the oxygen evolution reaction.

The apparent deficit between the total current and the sum of the currents accountable by ClO^- production (69.2%) and O_2 collection (20.5%) is approximately 10.3%. We attribute this primarily to the loss of Cl_2 via two pathways: (i) the reversibility of the reaction $Cl_2 + 2OH^- \rightleftharpoons Cl^- + ClO^- + H_2O$, and (ii) direct volatilization of Cl_2 gas into the atmosphere, which was corroborated by a distinct pungent odor detected during experiments. Therefore, the **total current fraction for Cl_2 generation** (including the portion that later hydrolyzed to ClO^- and the portion that was lost) was approximately 79.5% (69.2% + 10.3%).

Based on the above analysis, we assign **79.5% of the current to the Cl_2 generation pathway** (with a theoretical thermodynamic voltage of 1.36 V) and the remaining **20.5% to the O_2 generation pathway** (theoretical voltage of 1.23 V). With an actual applied cell voltage of 2.5 V and an overall measured Faradaic efficiency of 97.15%, the total energy efficiency of the system is calculated to be 51.3% using the ratio of the weighted average theoretical voltage to the input voltage.

Due to experimental limitations, a detailed quantitative analysis of the gas composition via gas chromatography (GC) was not performed. Therefore, reasonable assumptions were made regarding the gas collected from the anode. **Nevertheless, the repeated measurements for H_2 collection confirm that the performance enhancement is robust and results from the proposed strategy in this work, rather than systematic or measurement errors.** Although constrained by the lack of GC analysis for direct product quantification, the presented analysis based on ClO^- quantification and gas collection provides substantial supporting evidence for the enhanced hydrogen-production rate.

The hydrogen production demonstration in this work primarily aims to validate the high performance of the proposed multi-stage buck strategy, and thus no further optimization of the electrochemical reaction itself was performed. The achieved hydrogen production rate of 16.5 mL/(min·m^2) further underscores the critical importance of efficient circuit management in TENG-based self-powered electrochemical systems. We believe that future research could combine the circuit strategy proposed here with systematic reaction optimizations to

further enhance hydrogen production performance. For instance, developing efficient catalysts and designing structured electrodes could increase active sites and thereby reduce the reaction overpotential. Alternatively, screening suitable electrolyte systems and optimizing their concentration ranges could lower the ohmic loss of the system. These measures are expected to decrease the output voltage and improve the output current of the TENG and the overall energy conversion efficiency of the system while maintaining a high Faradaic efficiency.

Revisions

The revised part in the present manuscript is as follows:

We have made a revision of "the hydrogen production rate of the self-power hydrogen generation system reaches an impressive 16.5 mL/(min·m^2)" in the last paragraph of "**Introduction**".

We have made a revision of "Specifically, the production rate has increased from 41.34 μL/min to 72.33 μL/min by introducing of the secondary circuit, representing a 75% improvement in overall rate. The detailed electrochemical performance, including the specific reaction voltage and current, is comprehensively presented and analyzed in **Figure S36-37**, **Table S6-8** and **Note S20**." in the last paragraph of "**The multi-stage buck strategy for self-power systems**".

We have made a revision of "Integrated self-powered hydrogen systems exhibit 16.5 mL/(min·m^2) production rate, also representing a nearly order of magnitude increase compared to prior best of 2.5 mL/(min·m^2)." in the first paragraph of "**Discussions**".

We have revised the **Figure 5G** as follow:

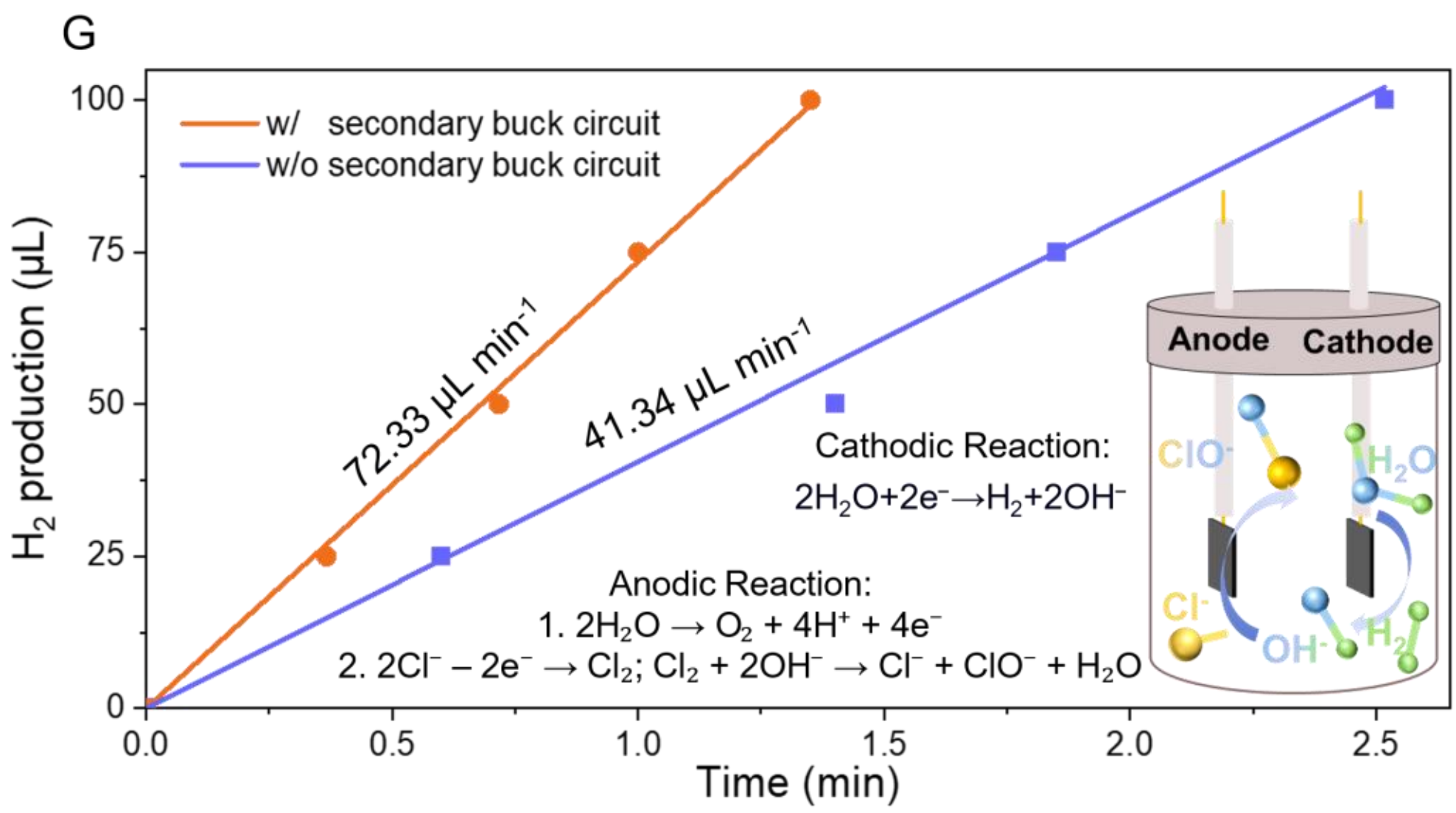

Revised Figure 5G The production rate of the self-powered hydrogen production system. The concentration of the NaCl solution used is 1 mol/L.

The revised part in the Supplementary Information is as follows:

We have revised the **Figure S36** as follow:

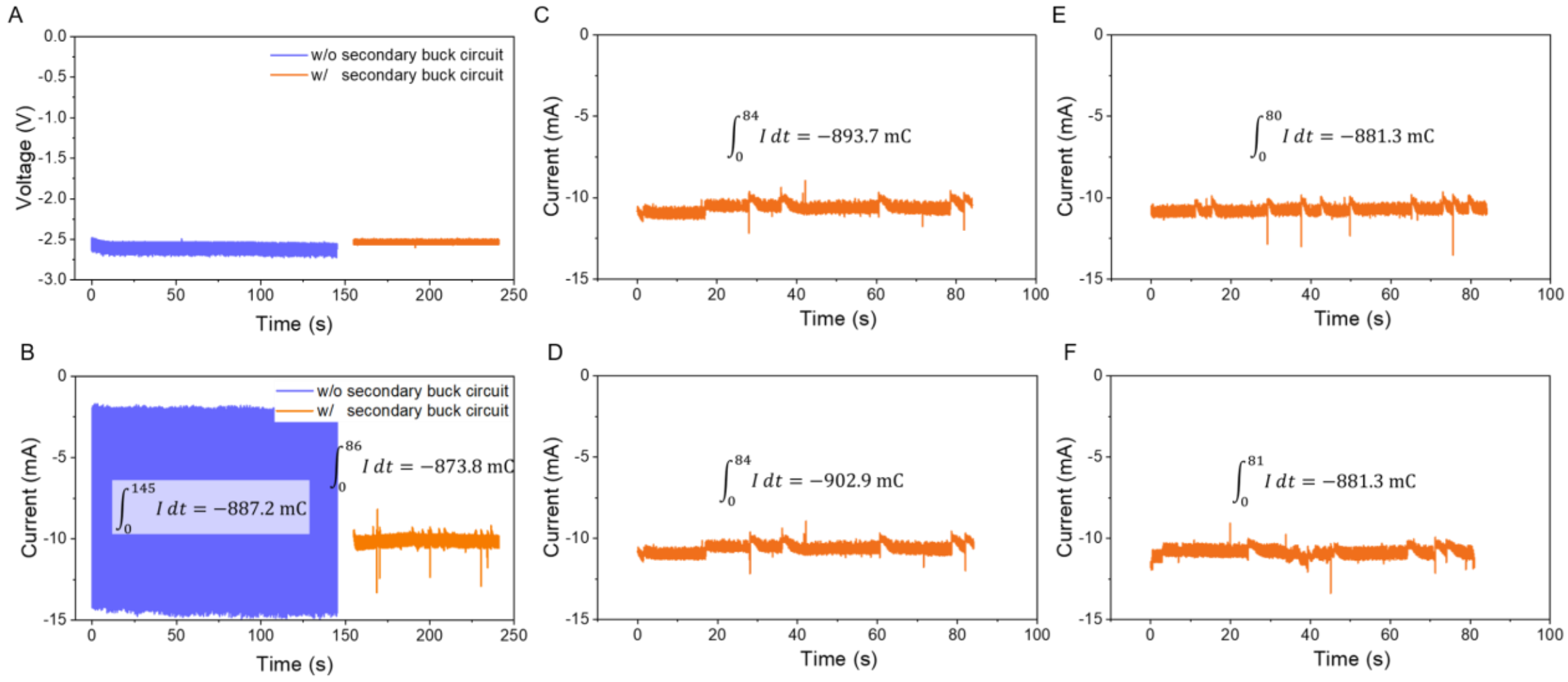

Figure S36. The operating voltage current of the self-power hydrogen production system. (A) Test voltage; (B)-(E) Test current.

We have added the above discussion into the "**Note S20 TENG-Driven Electrochemical Hydrogen Production**" in current Supplementary Information.

We have added the "**Table S6 The production rate of hydrogen and faradaic efficiency**", "**Table S7 The stability tests of production rate**" and "**Table S8 The production rate of anode gas**" in current Supplementary Information.

We have added the "**Figure S37. UV-Vis spectra test.**" in current Supplementary Information.

8. Figures 1–5 contain excessive sub-panels and small text, making them difficult to interpret. Simplify or enlarge critical plots, especially efficiency vs. voltage comparisons.

Response: We appreciate your professional suggestions. We have made the following modifications to Figure 1-5.

Revisions

The revised part in the present manuscript is as follows:

We have revised the **Figure 3** as follows:

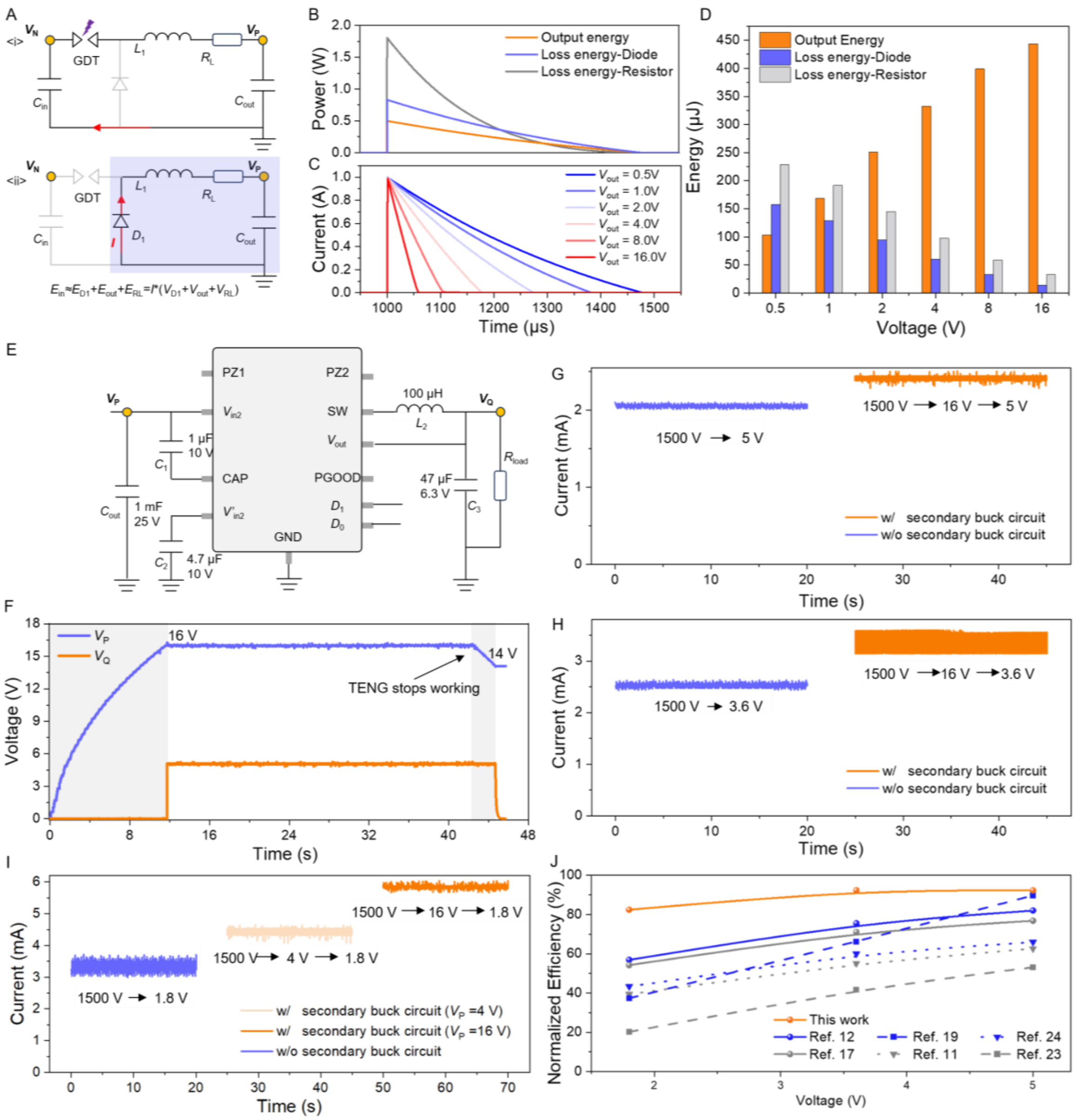

Figure 3. The secondary buck circuit. (A) The schematic diagrams of the primary buck circuit at different stages. Energy loss primarily occurs in the second stage (**Figure 3A< ii >**). (B) The output energy and energy consumed by internal impedance when the output voltage is 0.5 V. (C) The current of the primary buck circuit

as the output voltage gradually increases from 0.5 V to 16 V. (D) The comparison of output energy and energy consumed by internal impedance as the output voltage gradually increases from 0.5 V to 6 V. (E) The schematic diagram of the secondary buck circuit. (F) The input voltage V_P and output voltage V_Q of the secondary buck circuit. Set V_Q to 5 V. (G)-(I) The comparison of output currents when the output voltage is 5 V, 3.6 V and 1.8 V. (J) The normalization efficiency of different works under low-voltage range.

We have revised the **Figure 4** as follows:

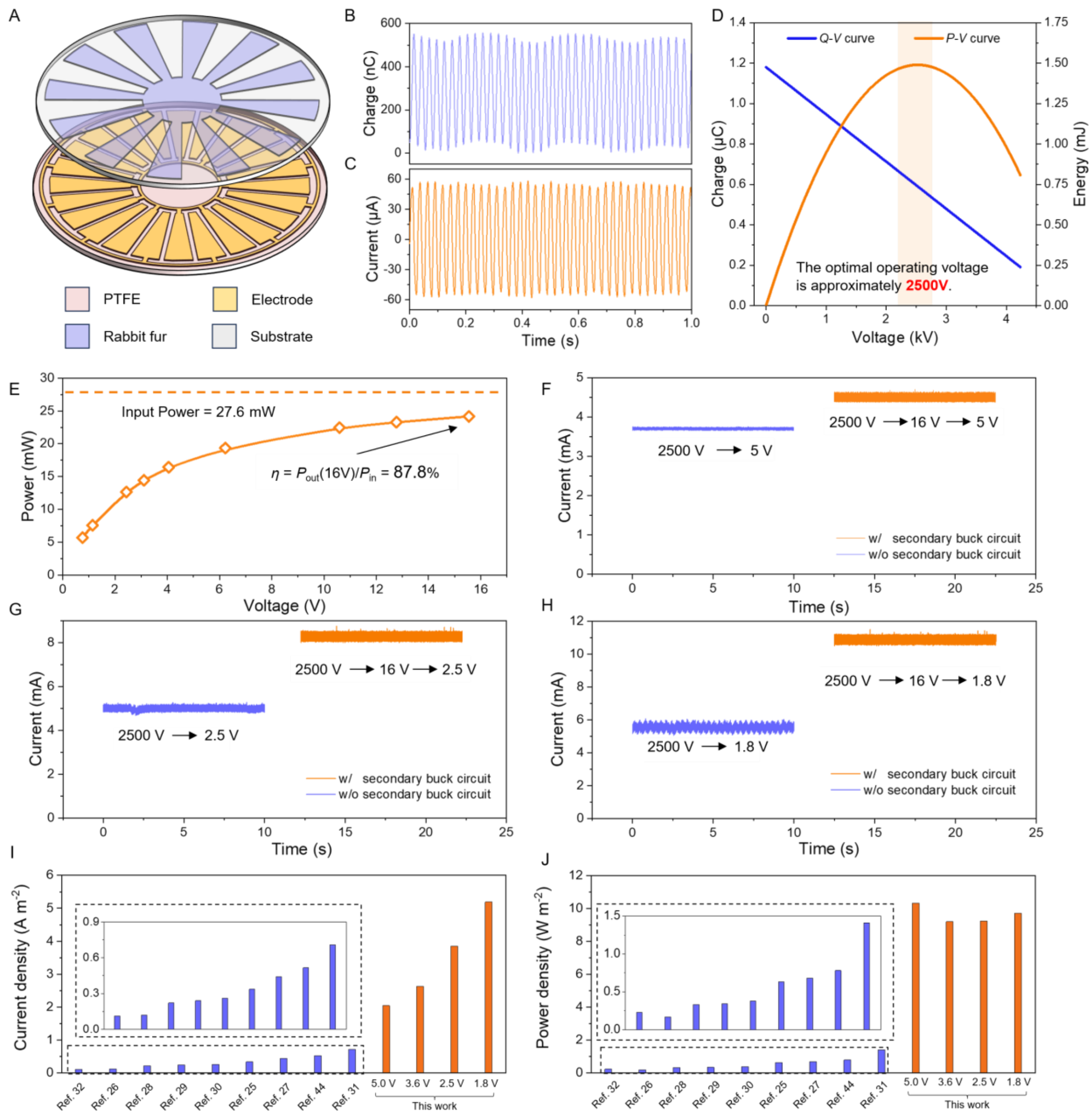

Figure 4. The multi-stage buck strategy for rotary mode TENG. (A) The schematic diagram of the structure for the rotary mode TENG. (B) The output charge and (C) the output current of the rotary mode TENG. The output charge density is about 116 μC m^{-2}. (D) The *Q-V* curve and *E-V* curve of the rotary mode TENG. The optimal operating voltage of the primary circuit is approximately 2500 V. (E) The output power of TENG with the primary circuit. When the output voltage is 16V, the circuit efficiency is 87.8%. (F)-(H) The comparison of output currents when the output voltage is 5 V, 2.5 V and 1.8 V. (I) The comparison of current density in different

works, (J) The comparison of power density in different works. The detail parameters are shown in **Table S5**.

We have made a revision of "At the V_Q of 1.8V (**Figure 3I** and **Figure S22B**), these figures rise to 177.7% (3.31 mA to 5.84 mA) and 176.4% (6.05 mW to 10.75 mW) of their original values (detailed data comparison is shown in the **Figure S23**)." in the second paragraph of the "**Regulation of input voltage for secondary buck circuit**".

We have made a revision of "Consequently, the overall circuit efficiencies improved from 68.0%, 53.3%, and 38.5% to 82.1%, 83.0%, and 73.0%, respectively (detailed data comparison is shown in the **Figure S28**)." in the "**The multi-stage buck strategy for rotary mode TENGs**

The revised part in the present supplementary information is as follows:

We have added the **Figure S23** in the present supplementary information.

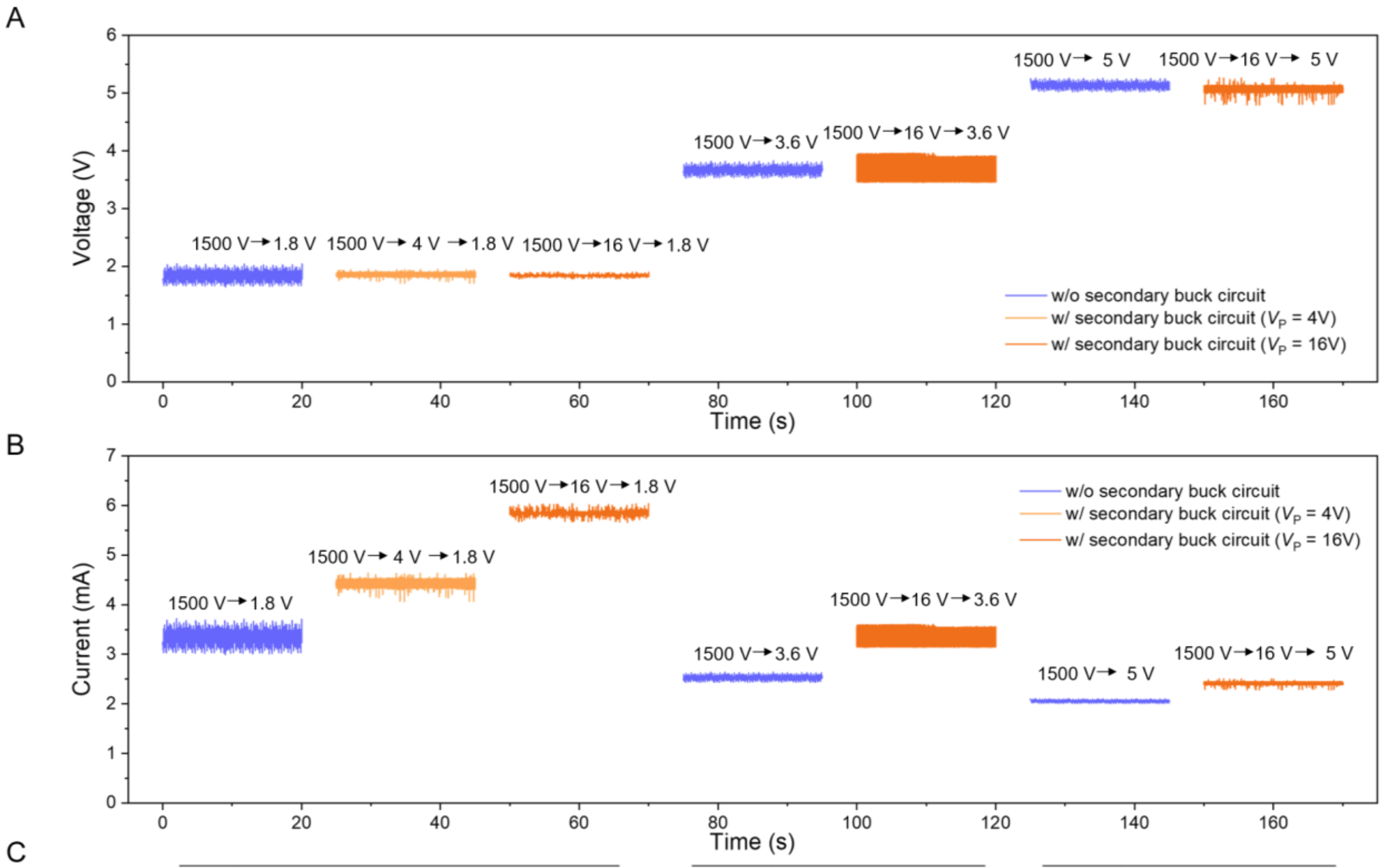

C

	w/o	w/ (V_P=4V)	w/(V_P=16V)
V	1.82 V	1.87 V	1.84 V
I	3.31 mA	4.56 mA	5.84 mA
R_{test}	0.55 kΩ	0.42 kΩ	0.315 kΩ
P	6.05 mW	8.53 mW	10.75 mW
η	42.2%	59.5%	75.0%

	w/o	w/
V	3.66 V	3.67 V
I	2.52 mA	3.34 mA
R_{test}	1.45 kΩ	1.1 kΩ
P	9.25 mW	12.27 mW
η	64.6%	85.6%

	w/o	w/
V	5.13 V	5.05 V
I	2.05 mA	2.41 mA
R_{test}	2.5 kΩ	2.1 kΩ
P	10.53 mW	12.12 mW
η	73.5%	84.6%

Figure S23 Performance comparison of rolling-mode TENG under different step-down paths. (A) The output voltage; (B) The output current; (C) The equivalent voltage (V), equivalent current (I), output power (P), test resistance (R_{test}) and efficiency (η)

We have added the **Figure S23** in the present supplementary information.

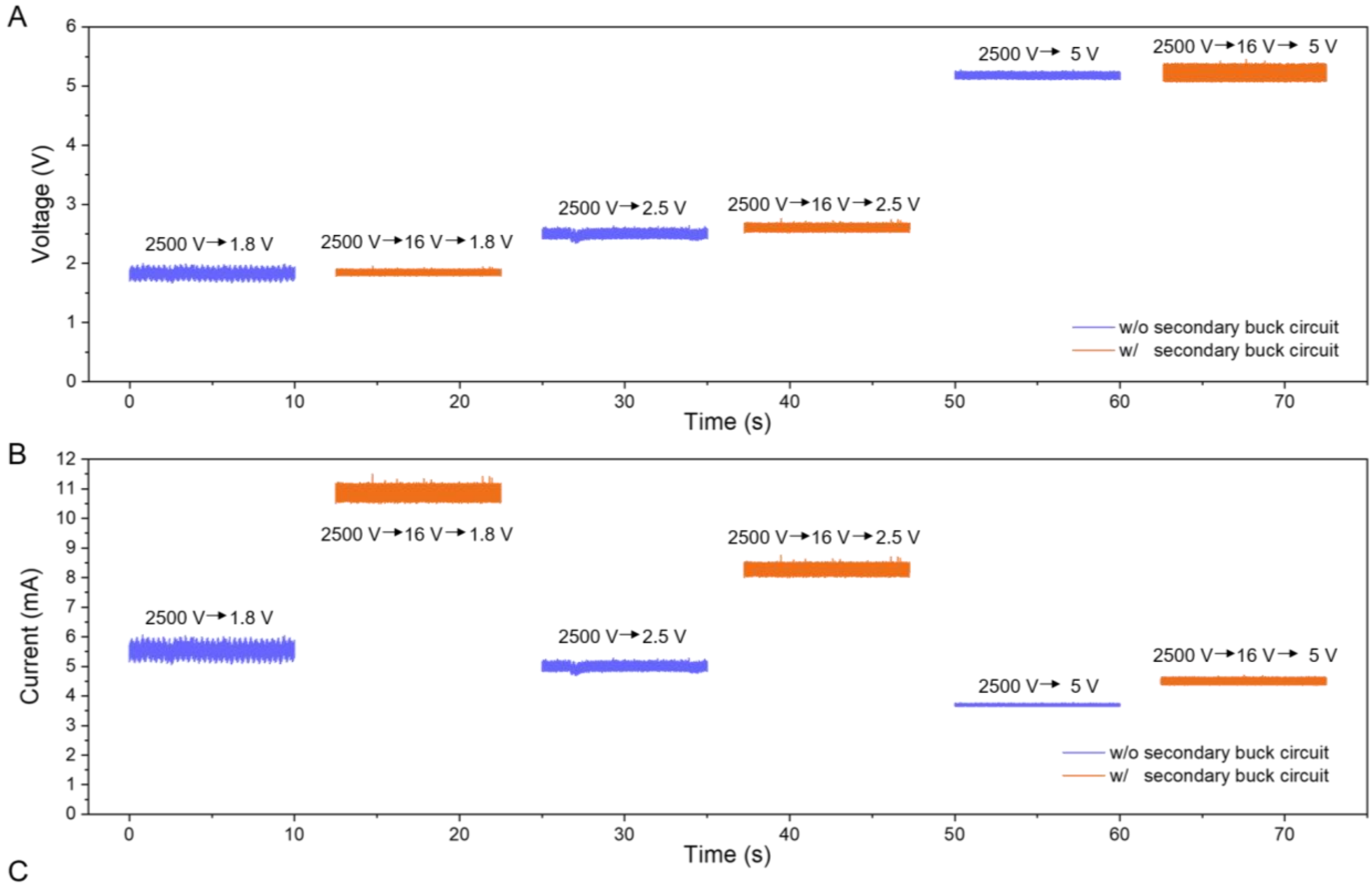

C

	w/o	w/
V	5.07 V	5.06 V
I	3.70 mA	4.48 mA
R_{test}	1.16 kΩ	1.4 kΩ
P	18.76 mW	22.67 mW
η	68.0%	82.1%

	w/o	w/
V	2.49 V	2.57 V
I	4.98 mA	8.23 mA
R_{test}	0.5 kΩ	0.315 kΩ
P	12.40 mW	21.3 mW
η	44.9%	77.2%

	w/o	w/
V	1.82 V	1.85 V
I	5.52 mA	10.89 mA
R_{test}	0.33 kΩ	0.17 kΩ
P	10.05 mW	20.15 mW
η	36.4%	73.0%

Figure S28 Performance comparison of rotary-mode TENG with 12 pairs of electrodes under different step-down paths. (A) The output voltage; (B) The output current; (C) The equivalent voltage (V), equivalent current (I), output power (P), test resistance (R_{test}) and efficiency (η).

9. Figure 3J ("Normalized efficiency of different works") requires a clearer explanation of the normalization criteria and error margins.

Response: We appreciate your professional review and reasonable suggestion.

1. **The difference between η_{PMC} between η_{total}**

   The calculation of "efficiency" varies fundamentally in different works. Some works use the maximum output power of the TENG under a resistive load as the denominator, and the "efficiency" calculated seems to reflect the TENG's total energy utilization rate (e.g., refs. 11, 17, 19 in **Figure 3J**). For instance, in ref. 17, the maximum output power is 1.09 mW at 2 GΩ, and the maximum output with PMC is 0.89 mW, yielding an efficiency of 81.6 %. Other works adopt the actual input energy to the PMC as the denominator, representing the actual conversion efficiency of the circuit (e.g., refs. 12, 23, 24 in **Figure 3J** and this work). For example, in ref. 12, with an input of 280.5μW and an output of 251.9 μW, the efficiency is 89.8%.

   The efficiencies reported in this work belong to the actual conversion efficiency of the PMC. To enable a fair and unified comparison across different works, and particularly to highlight the severe efficiency drop of conventional circuits at low voltages, all data were normalized.

2. **Normalization Method**

   For each referenced work, we divided all efficiency data across various voltages by the highest efficiency value reported in respective work. Thus, the ordinate "Normalized efficiency" represents the efficiency of a circuit at a given voltage as a percentage of its own best performance. This approach effectively eliminates systematic deviations arising from differences in "efficiency" definitions, allowing all data to be compared on a common baseline, which is the degree of performance drop relative to its own optimum.

   Taking ref. 19 in **Figure 3J** as an example, the circuit reported in that work exhibits a peak efficiency of 94.5% at 7.7 V. When the voltage drops to 1.8 V, its efficiency decreases to 35.2%. After normalization: 35.2% / 94.5% ≈ 37.3%. This indicates that at 1.8 V, the performance of this circuit is only 37.3% of its optimal value.

3. **Data Extraction and Comparison**

   Following the normalization method described above, we extracted the original data points from the relevant figures in all cited references and calculated the corresponding "normalized efficiency" using the highest efficiency value reported in each work. For a clearer and more direct comparison, the circuit efficiencies at 1.8 V, 3.6 V, and 5.0 V are summarized in the **Table R12** and **Table R13**. With the secondary circuit integrated, the efficiency of our design at 1.8 V remains as high as 83.0% of its optimum value, demonstrating that our strategy can sustain circuit performance much closer to the peak level, especially under low-voltage conditions.

   It should be noted that none of the cited references included error bars or corresponding raw datasets in their figures, therefore a quantitative comparison of uncertainties was not feasible in this analysis.

Table R12 (Revised Table S3) The reported efficiency in different works*

REF	Circuit	η (1.8 V)	η (3.6 V)	η (5.0 V)	Optimal efficiency
11	LC	32.1%	45.0%	51.1%	81.6%
12	LC	51.1%	67.8%	73.6%	89.8%
17	Transformer	42.6%	55.8%	60.3%	78.5%
19	FSCC	35.2%	62.5%	84.7%	94.5%
23	LC	18.3%	37.8%	48.1%	90.6%
24	LC	36.3%	50.4%	55.2%	83.9%
This Work	Multi-stage buck circuit	75.5%	86.3%	85.1%	90.9%

*The data for these works presented in the table were extracted from the performance curves or values reported in the respective references.

Table R13 (Added Table S4) The normalized efficiency in different works*

REF	Circuit	η (1.8 V)	η (3.6 V)	η (5.0 V)	Optimal efficiency
11	LC	39.4%	55.1%	62.6%	100%
12	LC	56.9%	75.5%	82.0%	100%
17	Transformer	54.2%	71.1%	76.8%	100%
19	FSCC	37.3%	66.1%	89.7%	100%
23	LC	20.2%	41.7%	53.1%	100%
24	LC	43.3%	60.0%	65.9%	100%
This Work	Multi-stage buck circuit	83.0%	94.9%	93.6%	100%

*The data for these works presented in the table were extracted from the performance curves or values reported in the respective references.

Summary

The core objective of this normalization method is to focus on the performance characteristic of "circuit efficiency drop with decreasing voltage." This effectively avoids the inherent differences in absolute efficiency values. It robustly demonstrates that, compared to conventional approaches, our strategy can maintain high performance more effectively across a wide voltage range, especially at low voltages, with a significantly smaller degree of performance decay.

Revisions

The revised part in the present manuscript is as follows:

We have revised the "Here, we normalized the data against using the best efficiency reported in each cited work (the discussions on different calculation methods are shown in the Note and Table S3-4). The core objective of

this normalization method is to focus on the one of core issues in circuits of "circuit efficiency drop with decreasing voltage". This effectively avoids the inherent differences in absolute efficiency values. Comparison results robustly demonstrate that, compared to conventional approaches, our strategy can maintain high performance more effectively across a wide voltage range, especially at low voltages, with a significantly smaller degree of performance decay." in the last paragraph of the part of "**Regulation of input voltage for secondary buck circuit**"

The revised part in the present supplementary information is as follows:

We have revised the **Table S3 The reported efficiency in different works** and **Table S4 The normalized efficiency in different works** in the present supplementary information.

We have merged the original **Note S17** and **Note S18** into **Note16 Efficiency comparison of different works**, and have made revisions according to above discussion.

10. Clarify whether "multi-stage buck" can be extended to >2 stages and discuss potential trade-offs (complexity vs. gain).

Response: We appreciate your professional review.

The proposed two-stage design can indeed be extended to three or more stages. This becomes particularly necessary for applications requiring an extreme voltage conversion ratio (e.g., from tens of kilovolts down to a few volts), because an input voltage at the ten-kilovolt level imposes extremely high rated-voltage requirements on the components in the primary circuit. Taking an LC circuit as an example, even though the voltage rating demand can be reduced by using series-connected freewheeling diodes, the substantially larger step-down ratio and the energy loss due to increased internal resistance would lead to a more pronounced circuit efficiency drop.

Therefore, for such ultra-high input voltages, a practical multi-stage approach could be:

First Stage: The FSCC could efficiently reduce the voltage from the ten-kilovolt level to the kilovolt level. This stage offers two key advantages: it relaxes the voltage rating requirements for subsequent components, and, since its output voltage is far greater than the forward voltage drops of its diodes, it can achieve a conversion efficiency exceeding 99%.

Second Stage: A robust LC resonant or transformer-coupled circuit could then step down the kilovolt-level voltage to an intermediate range (e.g., tens of volts).

Third Stage: Finally, a high-efficiency integrated buck converter (like the LTC3588) would provide precise, stable output at the target low voltage (e.g., 1.8V, 3.6V).

Beyond question, the adding stages is increased system complexity, encompassing control coordination and potential stability considerations. Therefore, while technically feasible, the decision to employ more than two stages should be driven by the specific voltage conversion ratio and power requirements.

Ultimately, our perspective is that optimizing TENG power management extends beyond circuit design alone; it critically depends on matching the output characteristics of the TENG with the input requirements of the PMC. A just pursuit of extremely high TENG output voltage can create significant challenges for circuit design, while a just pursuit of highly efficient circuit may not be compatible with existing high-performance TENGs.

Revisions

The revised part in the present manuscript is as follows:

We have added the above discussion into the "**Note S20 TENG-Driven Electrochemical Hydrogen Production**" in current Supplementary Information.

We have made the revision of the "Second, need for a three-stage buck circuit. For high-performance TENGs with output voltages reaching tens of thousands of volts, an additional pre-regulation circuit is necessary before

the proposed multi-stage buck circuit. This pre-regulation circuit is crucial to adjust the ultra-high voltage to levels that conventional electronic components can handle (**Note S23**)." in the last paragraph of the "**Discussion**".

The revised part in the present supplementary information is as follows:

We have added the above discussion into the "**Note S23 The three-stage buck circuit**" in current Supplementary Information.

11. The Methods section should specify model numbers and tolerances of GDTs, diodes, and inductors used.

Response: We appreciate your professional review and reasonable suggestion. We have added the specific model numbers of the relevant electronic components in the Methods section, with their parameters summarized in **Figure S12** and **Figure S21**.

Revisions

The revised part in the present manuscript is as follows:

We have made a revision of "

The electric component model numbers

The key components used in the primary circuit include the input capacitor [CBB81-2000V-221J], the GDTs [SXH81-152X], the inductor [SMDRI127-102MT], the freewheeling diode [FR1006GP-TP], and the output capacitor [UHE1E102MHD6TO]. The details parameters are shown in **Figure S12**. Adjusting threshold voltage and capacitance through series or parallel connection of electronic components.

The main components employed in the secondary circuit are capacitor C1 [GRM319R71A105KA01D], capacitor C2 [GRM31CR71A475KA01K], capacitor C3 [GRM31CC80J476KE18K], inductor L [SLF7045T-101MR50-PF], and the integrated circuit [LTC3588EMSE-2/LTC3588EMSE-1]. The details parameters are shown in **Figure S21**.

" in the "**Methods**".

The revised part in the present supplementary information is as follows:

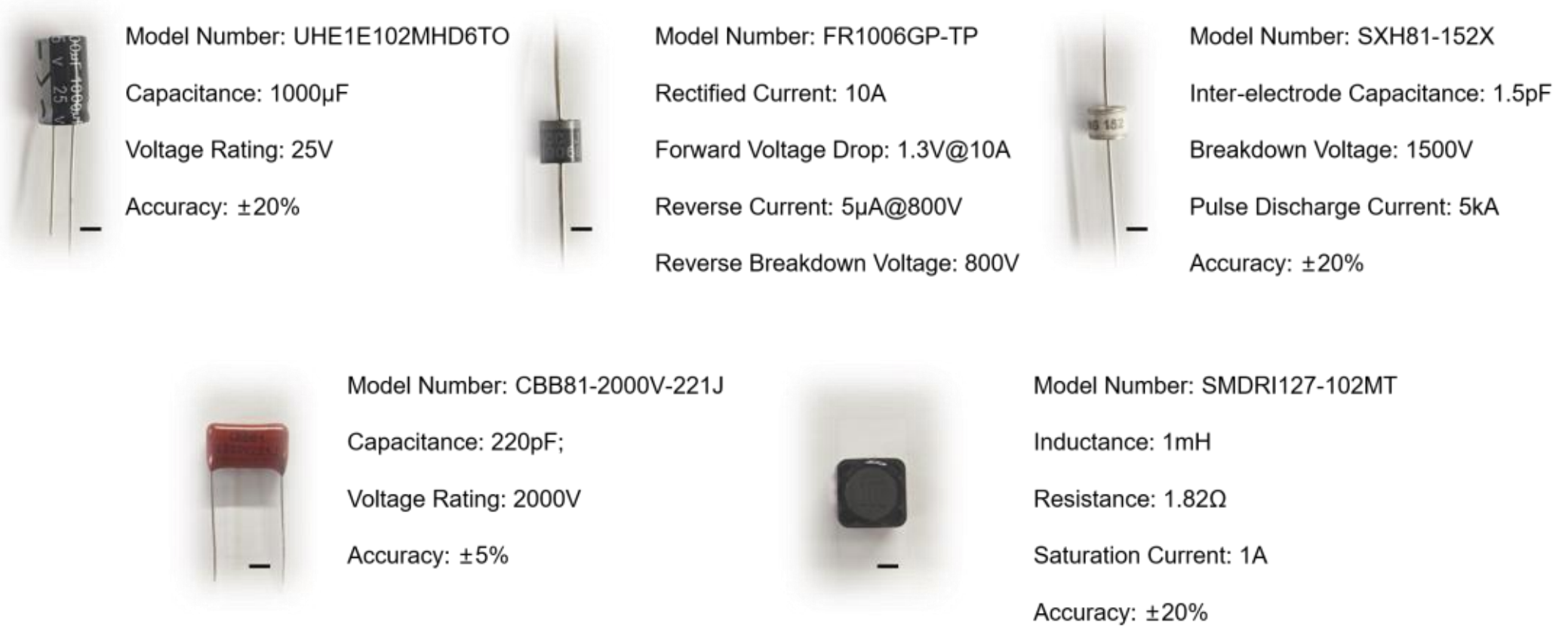

Figure S12. The photos and parameters of electronic components used in the primary circuit. Scale bar: 5 mm

LTC3588-1

LTC3588-2

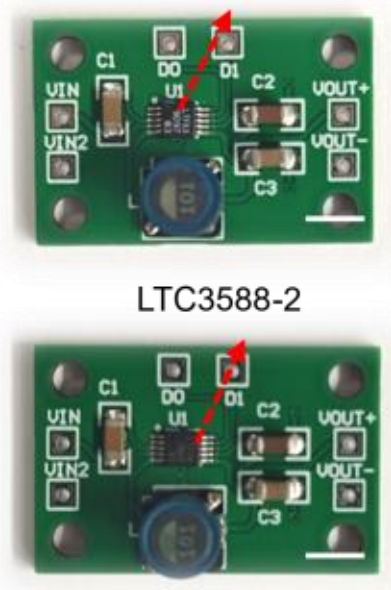

D0	D1	V_{out}(LTC3588-1)	V_{out}(LTC3588-2)
GND	GND	1.8V	3.45V
GND	Vin2	2.5V	4.1V
Vin2	GND	3.3V	4.5V
Vin2	Vin2	3.6V	5.0V

Designator	Description	Model	Parameters
C1	Chip Capacitor	GRM319R71A105KA01D	1uF/10V
C2	Chip Capacitor	GRM31CR71A475KA01K	4.7uF/10V
C3	Chip Capacitor	GRM31CC80J476KE18K	47uF/6.3V
L	Chip Shielded Power Inductor	SLF7045T-101MR50-PF	100uH
U1	Integrated Circuit (IC)	LTC3588EMSE-2/ LTC3588EMSE-1	/

Figure S21. The photos and parameters of electronic components used in the secondary circuit. Scale bar: 5 mm**.**

12. Add a brief limitations statement in the Discussion to indicate scalability and reliability challenges for industrial adaptation.

Response: We appreciate your professional review and reasonable suggestion. We have included a discussion of the scalability and reliability challenges for industrial adaptation.

Revisions

The revised part in the present manuscript is as follows:

We have made the revision of the "Despite the significant progress achieved with the proposed multi-stage buck strategy in this work, TENG power management will likely continue to face several key challenges in future practical applications. …… Finally, challenges for industrial transformation. For successful industrial adoption, the scalability of the circuit fabrication process and the long-term reliability of the integrated system under different environmental conditions are critical challenges that future work must address. In summary, this novel paradigm shift of TENG power management proposed in this work not only overcomes fundamental two challenges but also enables the practical implementation of high-performance self-charging power systems and self-powered electrochemical systems. This progress accelerates the transition of TENG technology from laboratory prototypes toward industrial-scale applications." in the last paragraph of the "**Discussion**".

References

1. Cao Z, *et al.* Ultrahigh power generation from low-frequency and micro motion by suppressing air breakdown. *Energy & Environmental Science* **17**, 5002-5012 (2024).
2. Gao Y, *et al.* Achieving high-efficiency triboelectric nanogenerators by suppressing the electrostatic breakdown effect. *Energy & Environmental Science* **16**, 2304-2315 (2023).
3. Gao Y, *et al.* Spontaneously established reverse electric field to enhance the performance of triboelectric nanogenerators via improving Coulombic efficiency. *Nature Communications* **15**, 4167 (2024).

Response to Reviewer #4

In the reviewed manuscript, the authors have introduced an innovative method for power control in Triboelectric Nanogenerators, termed the multi-stage buck strategy. The effectiveness of the proposed method has been successfully tested and validated on various TENGs.

The experiments, the obtained results, and the presentation are highly successful. This work makes a significant and original contribution to the literature.

I congratulate the authors for their valuable work. I recommend that the article be accepted for publication.

Response: We highly appreciate the reviewer for carefully reviewing our work, and thank your positive comments on our research work, which has encouraged us greatly.

Reviewer #5 (Remarks to the Author):

This study proposes a multi-stage buck strategy that synergistically combines a dual-stage voltage conversion circuit with the TENG's output characteristics based on the Q–V curve, enabling precise voltage regulation at critical nodes. I recommend major revision.

1. The authors should provide a more detailed discussion of the state-of-the-art to better support the significance of this work in introduction. To improve the efficiency of PMC for TENGs, what kinds of technologies have been utilized previously? Please include representative studies, their main contributions, and their limitations. Additionally, clarify whether similar work has been reported by other research groups, and clearly state how this work is unique.

Response: We have revised the Introduction section to include a more detailed discussion of relevant works. A comparative analysis of circuits with architectures similar to ours has been added in **Note S3**. While some reported circuit architectures share similarities with ours, the essence of our proposed multi-stage buck design lies not merely in its topology, but in the precise setting and dynamic regulation of key nodal voltages, which is precisely what addresses the two core issues of TENGs' PMC and represents a critical breakthrough not achieved in other works. Such similarity only serves to further underscore the deeper insight our study offers regarding TENG energy cycling and circuit design compared to these prior efforts.

Revisions

The revised part in the present manuscript is as follows:

We have made a revision of "The PMCs for TENGs have primarily utilized classic and efficient circuit architectures, such as inductor-capacitor (LC) circuits[10-16], transformer-coupled circuits[17,18], and switched-capacitor convertor (SCC)[19,20]. The optimization strategies for these circuits have consistently adhered to a core principle that achieves comprehensive efficiency improvement by minimizing energy loss within the PMC's electronic components[12,17,21-23]. For instance, Wang et al. optimized the design of transformers and inductors to provide guidance for LC and inductor-coupled circuits; Liu et al. developed a fractional-order SCC to mitigated voltage drop of conventional SCC; Wu et al. reduced charge and voltage losses at switch while achieving a stable voltage output by commercial chips; He et al. proposed a dual-loop circuit to minimizing rectifier bridge energy loss; and our prior work focused on reducing capacitor leakage current to increase efficiency. Through the optimization of electronic components, reported circuit efficiencies have reached as high as 90%, significantly enhancing the competitiveness of TENG-based systems. However, these efforts remain confined to component-level optimization and have yet to recognize, let alone resolve, two systemic challenges inherent to the TENG-PMC interface (**Note S1**)." in the second paragraph of "**Introduction**".

We have made a revision of "Its crucial mission is to further regulates the V_P to the target output voltage V_Q (the output voltage of the secondary circuit). As shown in **Figure 1D**, the multi-stage buck circuit designed in this work exhibits an efficiency of over 75% in the low-voltage range, outperforming circuits designed in other works. Although similar circuit architectures have been reported in previous works[32], the core issues in the TENG field have not been clearly understood and resolved (**Note S2-3**). This high efficiency of multi-stage buck circuit is largely attributed to the crucial regulation of V_N and V_P instead of circuit architecture. Specifically, V_N plays a vital role in determining the energy extraction efficiency from the TENG to C_{in}. Meanwhile, V_P ensures optimal circuit efficiency for the primary circuit. Detailed discussions on these aspects are presented in the following parts." in the "**Multi-stage buck strategy**".

The revised part in the present supplementary information is as follows:

Note S3 Technical Advance of This Work

Although the circuit architecture of some works is similar to that proposed in this work[6], the power management strategy in this work is fundamentally based on the TENG energy cycle collapse theory. It aims to address the two core issues mentioned above through precise regulation of key voltage nodes, representing a fundamental difference in design logic from these works. This distinction is clearly demonstrated in the following aspects.

One of our technical advances is accurately understanding the root cause of the efficiency drop in conventional circuits under low output voltage and proposing a practical and referable solution. Our analysis reveals that the primary reason for efficiency drop under low output voltage is the energy loss caused by diodes and internal resistance. As shown in **Figure 3D**, this energy loss increases significantly as the output voltage decreases. To address this, we designed a multi-stage buck circuit with an intermediate voltage of 16 V. This intermediate voltage was chosen based on a clear efficiency advantage: the primary circuit achieves an efficiency of 90.9 % at 16 V, thereby substantially reducing energy loss in primary circuit. More importantly, experimental comparison of two step-down paths (1500 V → 4 V → 1.8 V and 1500 V → 16 V → 1.8 V) shows that when the intermediate voltage is set to 4 V, the overall system efficiency is much lower than with a 16 V intermediate voltage, due to the considerably higher energy loss in the primary circuit (**Figure 3I**). This clearly confirms the importance of regulating the intermediate voltage node. Through this design, we effectively improve the overall system efficiency from the source. In contrast, other works neither recognized the fundamental issue described above nor provided a targeted solution.

Another key technical advance lies in the application of our proposed *Q-V* output characteristic curve based on Coulombic efficiency, which provides a direct and practical method for the optimal match between TENG and PMCs. This curve visually represents the output performance of a TENG across different output voltages, enabling accurate determination of the circuit's optimal operating voltage to maximize power delivery. The theoretical analysis on guiding PMC design using the *Q-V* curve demonstrate the feasibility and rationality of this strategy (**Note S6**). As a result, this work achieves a current density of 5.19 A/m² and a power

density of 9.71 W/m^2 at 1.8 V, which is markedly higher than the 0.34 A/m^2 and 0.61 W/m^2 reported in Reference [32]. The significant improvement is attributed **not only to resolving the low-efficiency issue at low output voltage, but also to synergy mechanism between the output characteristics of TENGs and the PMCs.** Furthermore, the enhanced performance is convincingly demonstrated in electrochemical applications, where an impressively high hydrogen production rate of 16.5 mL/(min·m^2) was achieved. This outcome also confirms that the efficiency breakthrough in TENG-powered electrochemical systems not only need application-specific optimizations, but also need to match the TENG's electrical output effectively with the subsequent electrochemical application. In contrast, most works lack such an output-oriented guidance method. The inherent issue of energy cycle collapse in the TENG used by their study has been clarified in our prior work. The phenomenon of energy cycle collapse significantly undermines the validity of their optimization mechanism, which relies solely on the ideal *V-Q* plot.

Thus, the similarity in circuit architecture further underscores the deeper understanding embodied in our work regarding TENG performance enhancement and circuit optimization. The essence of our multi-stage buck design lies not merely in its topological configuration, but in the precise setting and dynamic regulation of the key nodal voltages—which is precisely the key to solving the aforementioned two core issues and represents the critical advancement that other works failed to achieve.

2. Please include more detailed information in Table S2 such as input and output power, operating frequency of the TENG, and the circuit topology used.

Response: Thank you very much for your careful review and suggestion. We have added the information about frequency of the TENG, and the circuit architectures used in **Table S2**. Additionally, the input power and output power correspond to the power density without PMCs and power density with PMCs in Table 2, respectively, which have been adjusted accordingly in **Table S2**.

Revisions

The revised part in the present supplementary information is as follows:

Table S2. Performance of different high-performance TENGs with/without PMCs*.

Ref.	26	27	28	29	30	31	32
Power density without PMCs/Input power density (W/m^2)	7.62	21.6	20.7	12.3	87.26	32.12	23.2
Current density without PMCs (A/m^2)	0.0084	0.0121	0.0067	0.0129	0.032	0.012	0.0061
Power density with PMCs/Output power density (W/m^2)	0.17	0.68	0.33	0.34	0.38	1.41	0.23
Current density with PMCs (A/m^2)	0.12	0.44	0.22	0.24	0.26	0.71	0.11
The ratio of power density with PMCs to power density without PMCs	2.2%	3.1%	1.6%	2.8%	0.4%	4.4%	1.0%
The type and operating voltage (kV) of the switch	SCR < 0.8	SCR < 0.8	SCR < 0.8	SCR < 0.8	SCR < 0.8	SCR < 0.8	GDT 0.8
Frequency (Hz)	1	1	1	2	1	2	0.375
Circuit architectures	LC	LC	LC	LC	LC	LC	LC

*Some works did not report the relevant current density and power density after PMCs; therefore, comparisons were made based on the average charging rate and average power when the capacitor was charged to approximately 3V.

3. Provide a complete list of major components, including the diodes, inductors (for both the first and second stages of converter), and MOSFETs

Response: We appreciate your professional review and reasonable suggestion. We have added the specific model numbers of the relevant electronic components in the Methods section, with their parameters summarized in **Figure S12** and **Figure S21**.

Revisions

The revised part in the present manuscript is as follows:

We have made a revision of "

The electric component model numbers

The key components used in the primary circuit include the input capacitor [CBB81-2000V-221J], the GDTs [SXH81-152X], the inductor [SMDRI127-102MT], the freewheeling diode [FR1006GP-TP], and the output capacitor [UHE1E102MHD6TO]. The details parameters are shown in **Figure S12**. Adjusting threshold voltage and capacitance through series or parallel connection of electronic components.

The main components employed in the secondary circuit are capacitor C1 [GRM319R71A105KA01D], capacitor C2 [GRM31CR71A475KA01K], capacitor C3 [GRM31CC80J476KE18K], inductor L [SLF7045T-101MR50-PF], and the integrated circuit [LTC3588EMSE-2/LTC3588EMSE-1]. The details parameters are shown in **Figure S21.**

" in the "**Methods**".

The revised part in the present supplementary information is as follows:

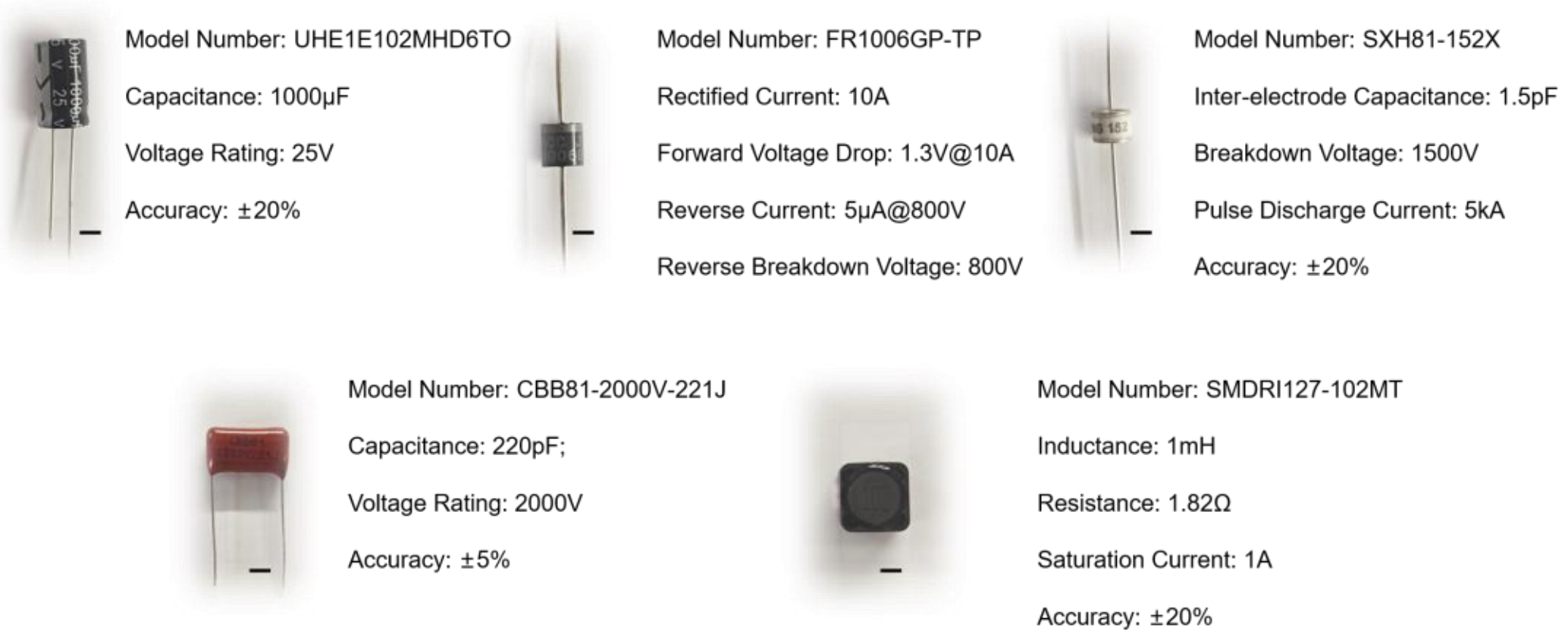

Figure S12. The photos and parameters of electronic components used in the primary circuit. Scale bar: 5 mm

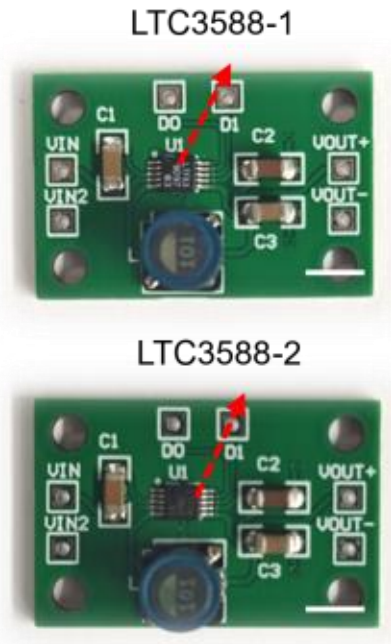

D0	D1	V_{out}(LTC3588-1)	V_{out}(LTC3588-2)
GND	GND	1.8V	3.45V
GND	Vin2	2.5V	4.1V
Vin2	GND	3.3V	4.5V
Vin2	Vin2	3.6V	5.0V

Designator	Description	Model	Parameters
C1	Chip Capacitor	GRM319R71A105KA01D	1uF/10V
C2	Chip Capacitor	GRM31CR71A475KA01K	4.7uF/10V
C3	Chip Capacitor	GRM31CC80J476KE18K	47uF/6.3V
L	Chip Shielded Power Inductor	SLF7045T-101MR50-PF	100uH
U1	Integrated Circuit (IC)	LTC3588EMSE-2/ LTC3588EMSE-1	/

Figure S21. The photos and parameters of electronic components used in the secondary circuit. Scale bar: 5 mm

4. Include the power losses of major components (diodes, inductors in the first and second stages, and MOSFETs) to support the claimed efficiency.

Response: Thank you very much for your careful review and suggestion.

A primary objective of the multi-stage circuit design in this work is to enhance the efficiency of the primary (LC) circuit by regulating the intermediate voltage, which also is the output voltage of primary circuit and the input voltage of secondary circuit, thereby improving the system's overall performance.

Our analysis confirms that using a single-stage LC circuit alone leads to significantly increased energy loss at low output voltages. The fixed losses from the inductor's internal resistance and the diode forward voltage drop dominate the energy transfer process at low output voltages, resulting in drastically reduced efficiency. This negative effect becomes even more pronounced when the diode's forward voltage drop is higher.

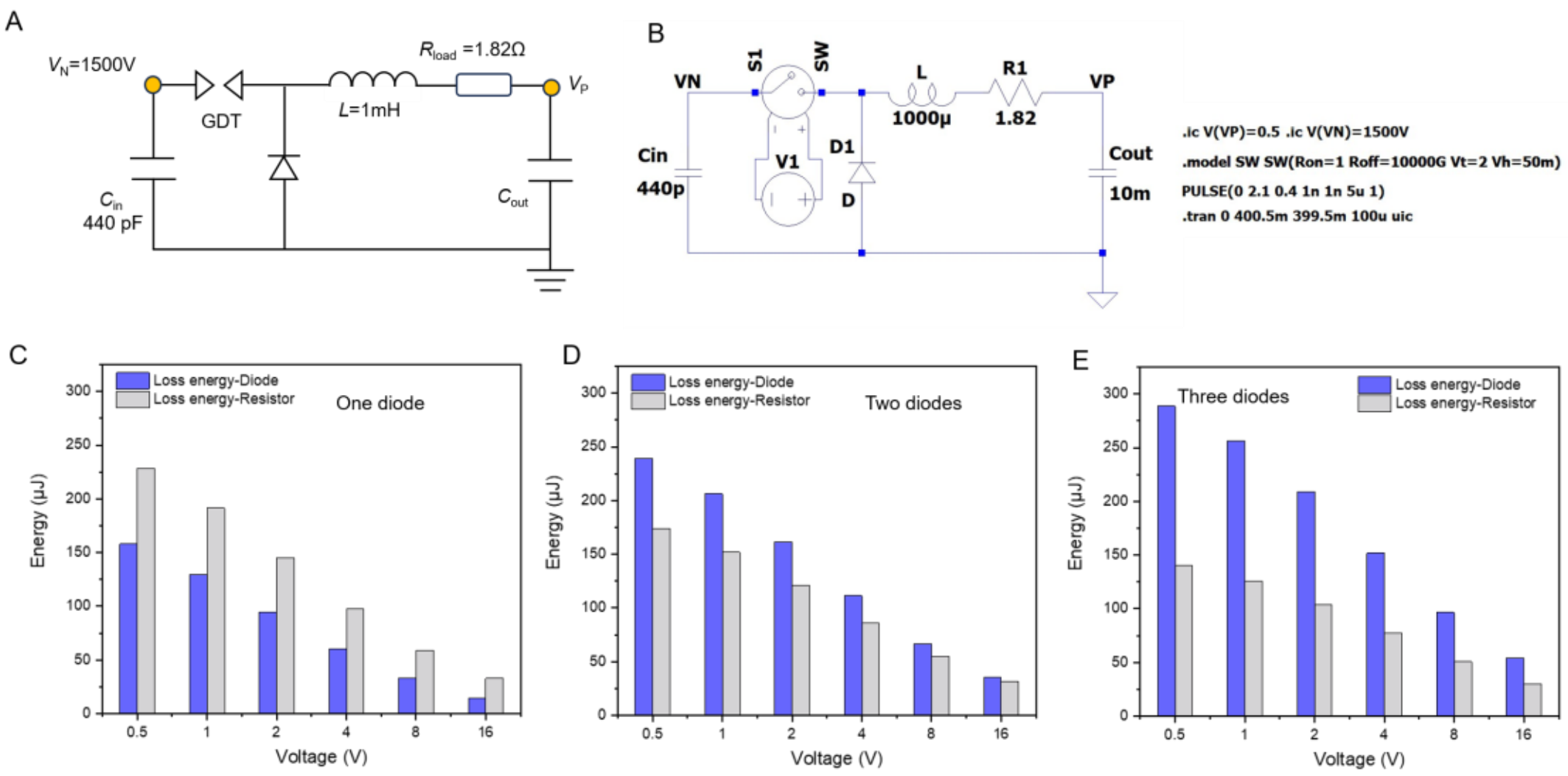

Figure R26. The circuit simulation results. (A) The equivalent circuit of LC circuit; (B) The circuit simulation parameters; (C) The loss energy of resistor and one diode. V_{diode} is about 0.83 V. (D) The loss energy of resistor and two diodes. V_{diode} is about 1.66 V. (E) The loss energy of resistor and three diodes. V_{diode} is about 2.49 V. The input energy is 495 μJ.

To estimate these losses, we performed circuit simulations. The equivalent circuit for the LC circuit is shown in **Figure R26A**, and the simulation schematic is shown in **Figure R26B**. In the simulation, a voltage-controlled switch is used to replace the GDT for circuit control. The parameters are as follows: input capacitance (C_{in}) 440 pF, output capacitance (C_{out}) 10 mF, inductance (L) 1 mH, switch threshold voltage 1500 V. The circuit internal resistance (R_{int}, mainly from the inductor) is 1.82 Ω, and the freewheeling diode forward voltage drop (V_{diode}) is 0.83 V (increasing the number of diodes to improve voltage drop). Although a precise GDT switch model was not established and the circuit is idealized, this model still effectively analyzes the impact of V_{diode} and R_{int} on energy loss.

The results clearly demonstrate that energy dissipation across the diode and the internal resistance decreases substantially as the output voltage increases (**Figure R26C-E**). This trend directly supports our design rationale

and the reported high efficiency. Therefore, in the multi-stage circuit design, an intermediate voltage of 16 V was selected to significantly enhance the efficiency of the primary circuit.

Regarding the secondary circuit, it is implemented using a commercial integrated circuit (IC). It internally employs synchronous rectification, where integrated MOSFETs replace Schottky diodes. The on-resistance of these MOSFETs is extremely low (typically tens of milliohms), resulting in minimal voltage drop and lower energy loss during the freewheeling period. Furthermore, the energy losses associated with its internal components are well-characterized in the manufacturer's datasheet. Under the optimized operating conditions achieved by our primary circuit design, the secondary circuit operates with high efficiency (~93%). Therefore, additional loss simulations for the secondary circuit were not conducted in here.

Revisions

The revised part in the present manuscript is as follows:

We have revised the **Figure S3B-D**.

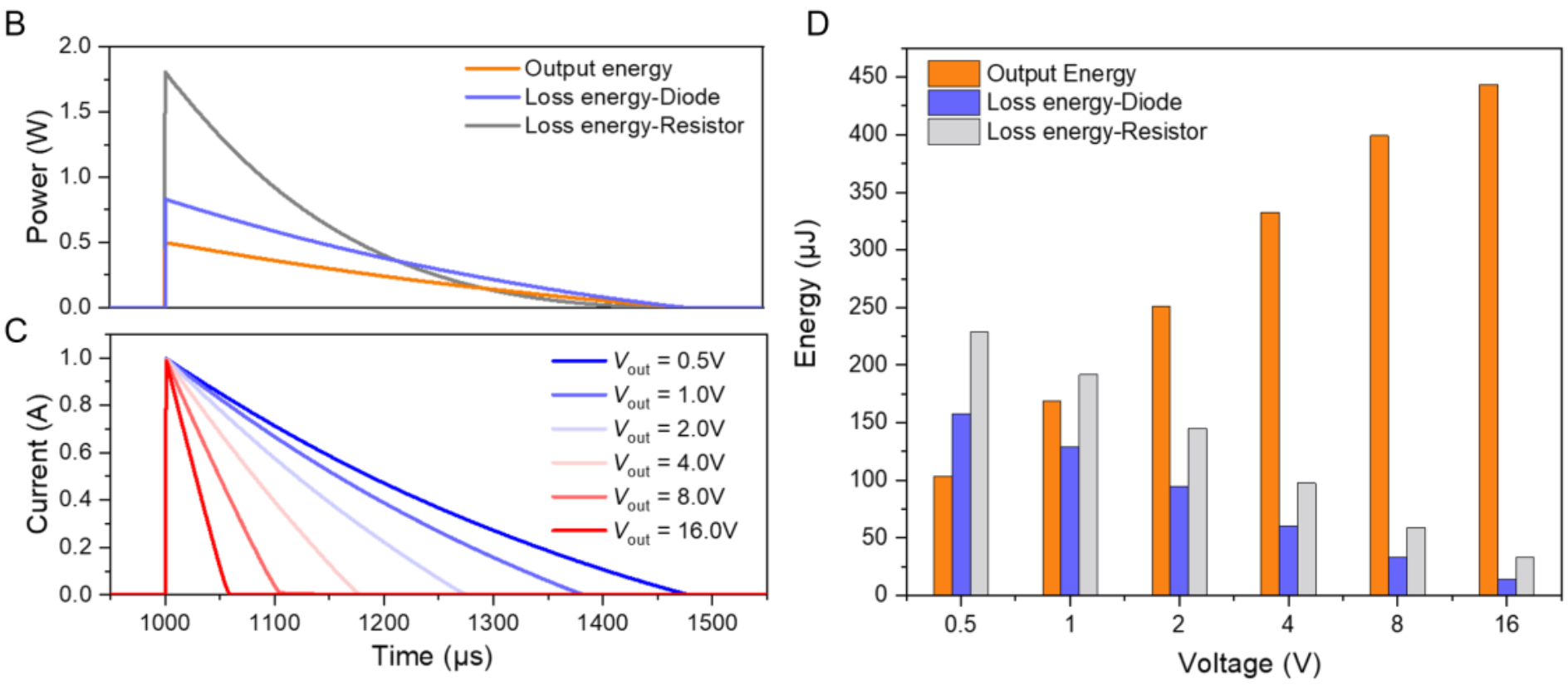

Figure 3. (B) The output energy and energy consumed by internal impedance when the output voltage is 0.5 V. (C) The current of the primary buck circuit as the output voltage gradually increases from 0.5 V to 16 V. (D) The comparison of output energy and energy consumed by internal impedance as the output voltage gradually increases from 0.5 V to 16 V.

We have revised the "At this moment, the freewheeling diode conducts to provide a path for the inductor's current to continue flowing (**Figure 3A <ii>**, and the detailed analysis is shown in **Note S10**.). Our simulation analysis revealed that the primary cause of the LC circuit's efficiency degradation is the significant increase in energy loss during this process at low voltages (**Figure S16** and **Note S13**)." in the first paragraph of the part of "**The reasons for low efficiency in primary circuits**"

We have revised the "

the circuit efficiency of the LC circuit also gradually improves accordingly (**Figure S16K**). This trend is consistent with the experimental results shown in **Figure 2M**.

” in the penultimate paragraph of the part of “**The reasons for low efficiency in primary circuits**”

The revised art in the present Supplementary Information is as follows:

We have revised and merged the original **Figure S13, Figure S15** and **Figure S15** into **Figure S16.**

We have consolidated the previous “**Note S11 The influence of the number of freewheeling diodes on the multi-stage buck circuit.**” and “**Note S13 The impact of increasing output voltage on energy loss**” into a single “**Note S13 The simulation for LC circuit**” in the present supplementary information as follow:

Note S13 The simulation for LC circuit

The equivalent circuit for the LC circuit is shown in **Figure S16A**, and the simulation schematic is shown in **Figure S16B**. In the simulation, a voltage-controlled switch is used to replace the GDT for circuit control. The parameters are as follows: input capacitance (C_{in}) 440 pF, output capacitance (C_{out}) 10 mF, inductance (L) 1 mH, switch threshold voltage 1500 V. The circuit internal resistance (R_{int}, mainly from the inductor) is 1.82 Ω, and the freewheeling diode forward voltage drop (V_{diode}) is 0.83 V (increasing the number of diodes to improve voltage drop). Although a precise GDT switch model was not established and the circuit is idealized, this model still effectively analyzes the impact of V_{diode} and R_{int} on energy loss.

Figures S16C-G present key waveforms for one operating cycle. Specifically, they show the circuit current, the voltage across the internal resistance, the diode voltage, the input voltage, and the output voltage. Substituting the circuit parameters into **Equations (27) and (32)** allows calculation of the current duration and the max current. As the voltage increases from 0.5 V to 16 V, the duration decreases sequentially: 473 μs, 379 μs, 272 μs, 175 μs, 103 μs, and 56 μs. Throughout this range, the maximum current is essentially unchanged at 0.995 A. These calculated values validate the simulation results presented in **Figure S16E**.

Figures S16H-J show the energy distribution across the diode, internal resistance, and output capacitor. With a total input energy of 495 μJ, most of the energy is distributed among these three components. As the output voltage increases, the energy dissipated by the diode and internal resistance decreases, leading to a higher useful output energy. In addition, increasing the number of diodes raises the diode energy loss at low output voltages, thereby reducing the available output energy. The simulated circuit efficiency in **Figure S16K** shows a trend consistent with the experimental results (**Figure 2M**). This trend directly supports our design rationale and the reported high efficiency. Therefore, in the multi-stage circuit design, an intermediate voltage of 16 V was selected to significantly enhance the efficiency of the primary circuit.

Regarding the secondary circuit, it is implemented using a commercial integrated circuit (IC). It internally employs synchronous rectification, where integrated MOSFETs replace Schottky diodes. The on-resistance of these MOSFETs is extremely low (typically tens of milliohms), resulting in minimal voltage drop and lower energy loss during the freewheeling period. Furthermore, the energy losses associated with its internal

components are well-characterized in the manufacturer's datasheet. Under the optimized operating conditions achieved by our primary circuit design, the secondary circuit operates with high efficiency (~93%). Therefore, additional loss simulations for the secondary circuit were not conducted in here.

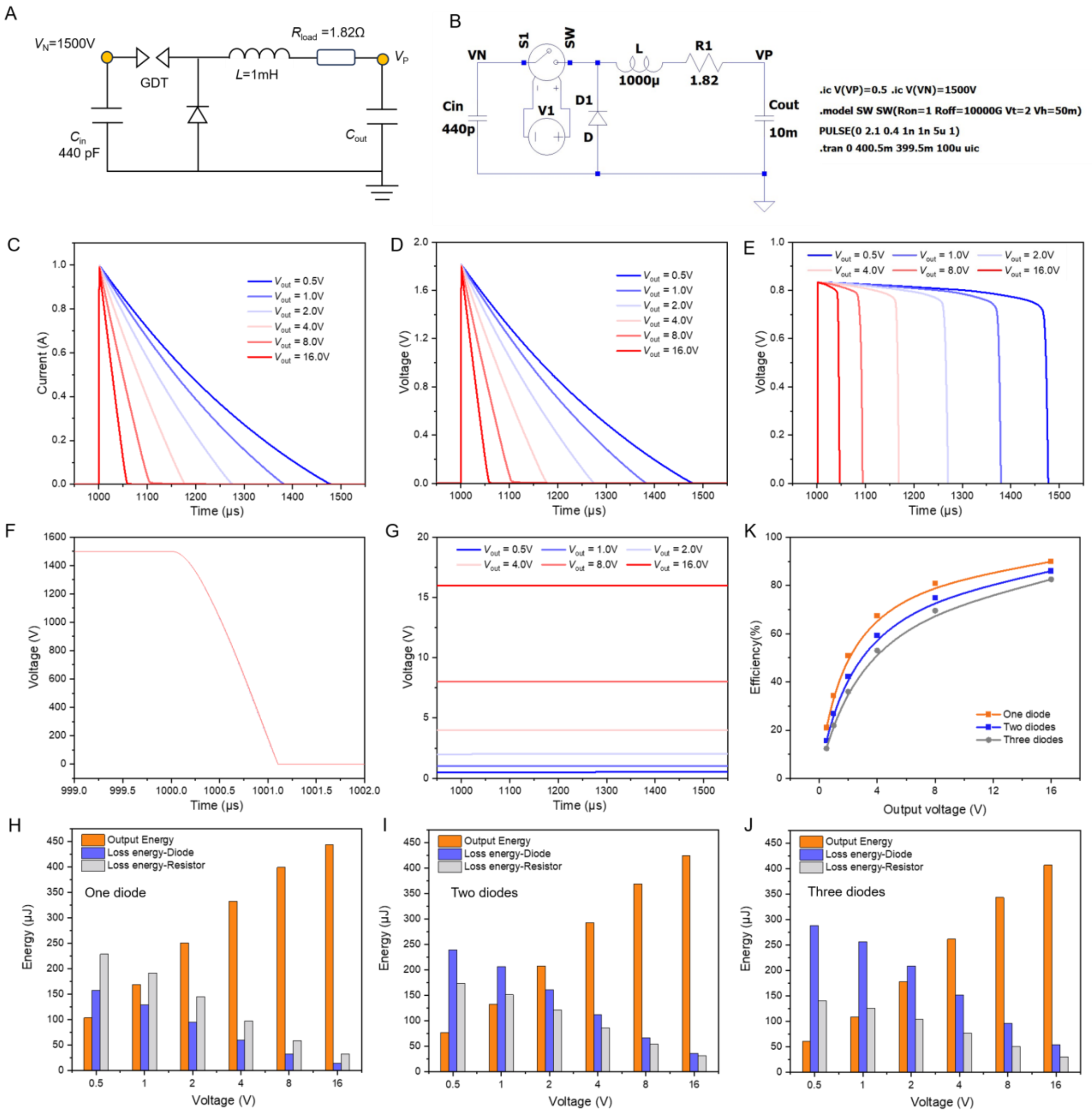

Figure S16. The circuit simulation results. (A) The equivalent circuit of LC circuit; (B) The circuit simulation parameters; (C) The waveform of current; (D) The waveform of voltage across the internal resistance; (E) The waveform of diode voltage; (F) The voltage of input capacitor; (G) The voltage of output capacitor; (H) The loss energy of resistor and one diode and the output energy. V_{diode} is about 0.83 V. (D) The loss energy of resistor and two diodes and the output energy. V_{diode} is about 1.66 V. (E) The loss energy of resistor and three diodes and the output energy. V_{diode} is about 2.49 V. The input energy is 495 µJ.

5. The description of circuit efficiency is unclear in abstract. Please provide more specific details (e.g., the efficiencies of the first and second buck converter stages).

Response: We appreciate your professional review and reasonable suggestion.

The intermediate voltage of the multi-stage buck circuit is 16 V. When the output voltage of the primary circuit is 16 V, the output power is 13.0 mW with an efficiency of 90.9% (the input power is 14.3 mW) (**Figure R27A**). The input voltage of the secondary circuit is 16 V, the output voltage is 5.05 V, and the output power is 12.12 mW with an efficiency of 93.2% (the input power is 13.0 mW) (**Figure R27B**).

We have revised the abstract to include details about the efficiency of the primary and secondary circuits.

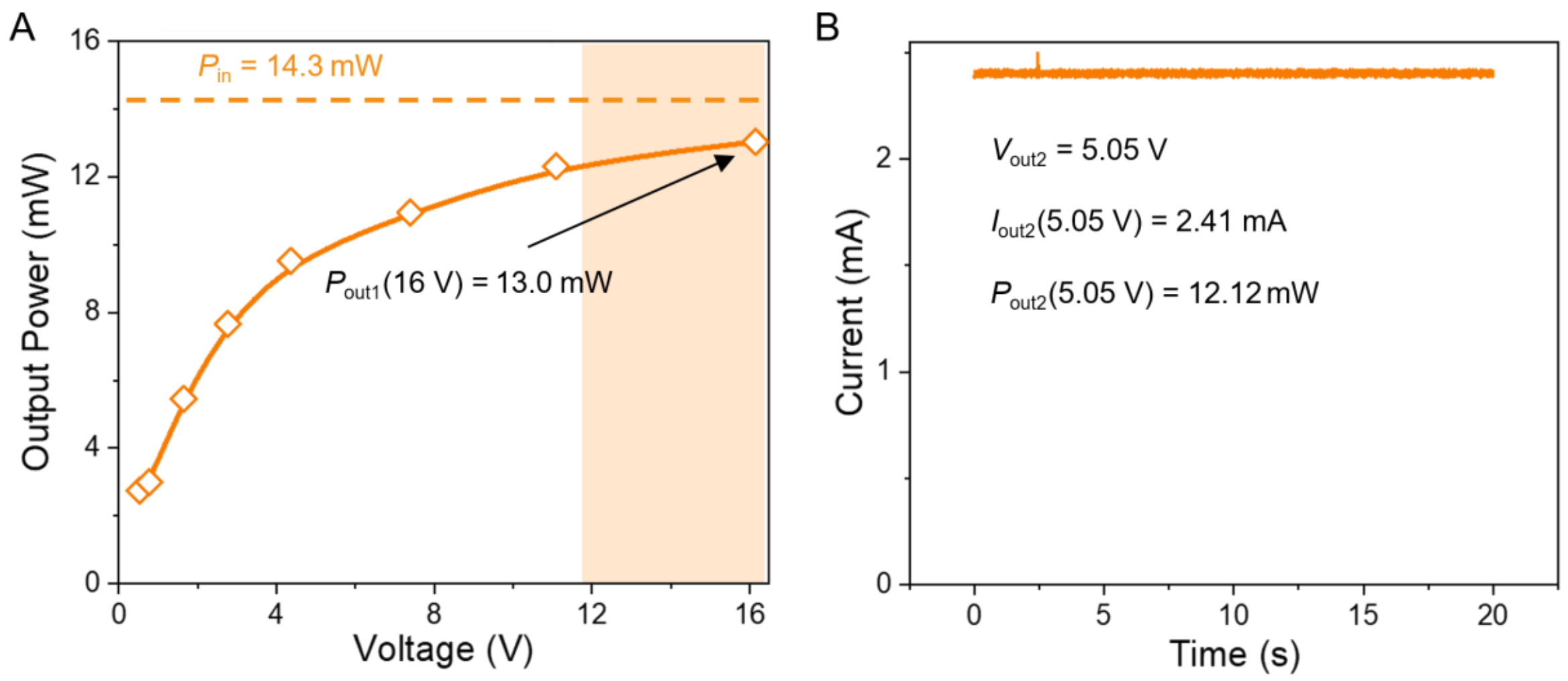

Figure R27. (A) Output power of the primary circuit; (B) Output power of the secondary circuit at the output voltage of 5 V.

Revisions

The revised part in the present manuscript is as follows:

We have made a revision of "The system attains an overall circuit efficiency of approximately 80% in the low-voltage range (90.9% in the primary circuit and 93.2% in the secondary circuit)." in the "**Abstract**"

Point-by-point responses to the reviewers' comments

We (the authors) would like to thank the editor and reviewers for dedicating their valuable time and expertise to assess the manuscript. We appreciate the reviewers' insightful comments, which contributed to enhancing the quality of the present work. In the following we elucidate our response to each remark, along with the corresponding revisions made in the manuscript. (**Comments in Black, responses in Blue, revisions in Red**)

Reviewer #3 (Remarks to the Author):

The authors are required to address the following critical points for further consideration:

1.The efficiency claims (80–86% at low voltage) require clearer definition of measurement boundaries, calibration method, and uncertainty analysis; without this, the reported improvement cannot be independently validated.

Response: Thank you for your professional comments.

Measurement boundaries: Figure R1 shows a simplified schematic of our measurement circuit. For efficiency calculation, the input power is the power at node N, and the output power is the power at node Q. The reported efficiency of this work is only the efficiency of the overall multi-stage circuit.

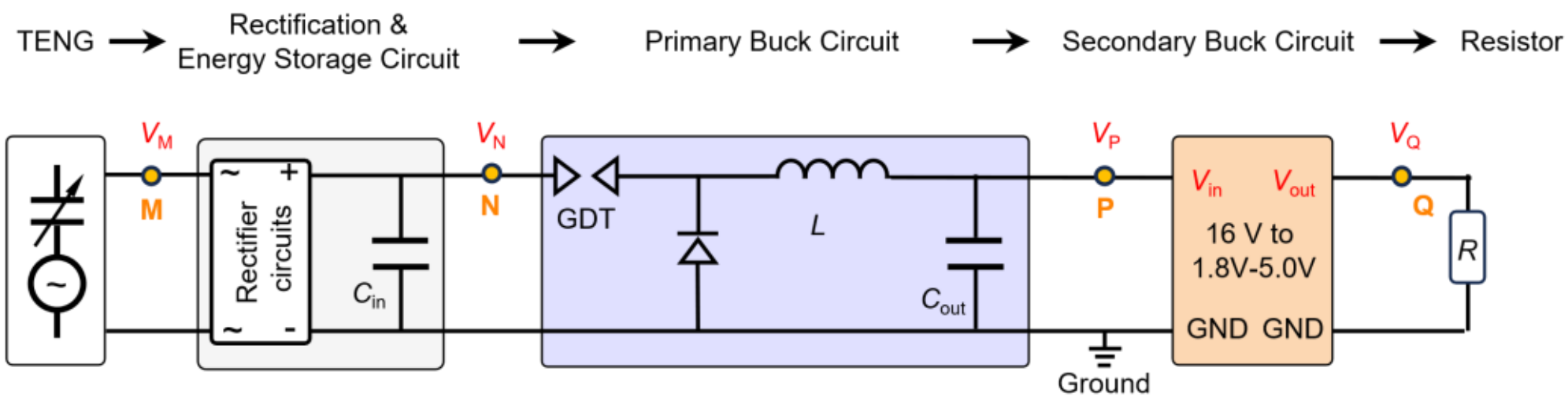

Figure R1. Simplified schematic diagram of the multi-stage circuit.

Calibration method: Prior to each measurement, the electrometer was powered on and allowed to warm up for at least 30 minutes. Then, the built-in zero check and auto-calibration routine was executed to null the offset voltage and bias current. This procedure was repeated before each individual measurement to eliminate drift.

Uncertainty analysis: Five independent repeated measurements of efficiency were performed under identical operating conditions including the same excitation, load, and environmental conditions. The results are shown in **Table R1** below. These results demonstrate good repeatability and stability of the measurement method, with

the systematic error being well controlled.

Table R1 (Table S2) The results of five measurements of circuit efficiency

	1st	2nd	3rd	4th	5th	Mean	Standard Deviation
1.8V	75.0%	75.4%	75.6%	75.4%	76.3%	75.5%	0.47749%
3.6V	85.6%	86%	87.4%	86.8%	85.9%	86.3%	0.74027%
5.0V	84.6%	85%	85.3%	85.4%	85.3%	85.1%	0.30822%

Furthermore, according to the Model 6514 electrometer user manual, the accuracy of the 20 V voltage range is ±0.025%, and the accuracy of the 2 mA and 20 mA current ranges is ±0.1%. The test resistor used has an accuracy of ±0.1%.

This work has comprehensively demonstrated that a multi-stage circuit can address the low-efficiency issue at low voltages commonly observed in single-stage circuits in current TENG research. We agree with the reviewer that a more rigorous uncertainty analysis, including instrument accuracy, connection losses, environmental fluctuations, and other factors, is important for the future engineering applications of TENGs. At present, however, the field still lacks unified measurement standards for such analyses. Given that the primary objective of this work is to validate a proof-of-concept efficiency improvement, we performed five independent repeated measurements to ensure the reliability of the main conclusions.

2.The central theoretical premise—the 50% Coulombic efficiency condition derived from a linear σ–V decay model—needs stronger experimental verification across the full operating range to confirm general validity.

Response: Thank you for your professional comments.

In high-performance TENGs, particularly those operating in sliding mode, the linear decay of surface charge density with increasing output voltage is a common phenomenon. This is mainly attributed to the fact that discharge preferentially occurs at the electrode edges, as demonstrated in our previous work (*Nat. Commun.* **15**, 4167 (2024)).

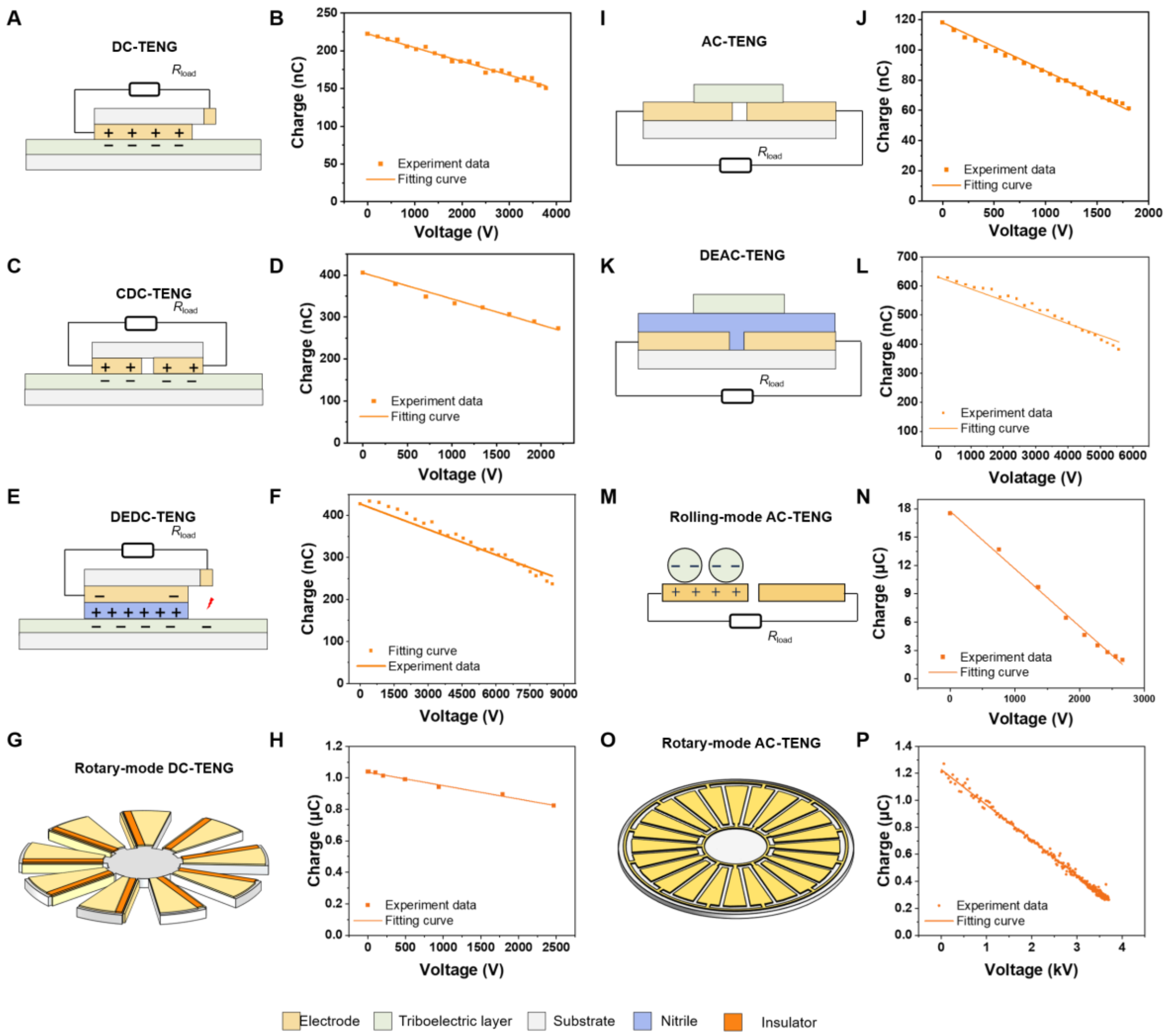

Figure R2. The *Q-V* curves of TENGs with different structures. (A) The schematic diagram of DC-TENG and (B) its *Q-V* curve; (C) The schematic diagram of coplanar-electrode DC-TENG (CDC-TENG) and (D) its *Q-V* curve; (E) The schematic diagram of double dielectric layer DC-TENG (DEDC-TENG) and (F) its *Q-V* curve; (G) The schematic diagram of rotary-mode DC-TENG and (H) its *Q-V* curve; (I) The schematic diagram of AC-TENG and (J) its *Q-V* curve; (K) The schematic diagram of double dielectric layer AC-TENG (DEAC-TENG) and (L) its *Q-V* curve; (M) The schematic diagram of rolling-mode AC-TENG and (N) its *Q-V* curve; (O) The schematic diagram of rotary-mode AC-TENG and (P) its *Q-V* curve;

Briefly, the electric field strength is typically highest at the electrode edges. The magnitude of the edge electric field is proportional to the surface charge density and the operating voltage of the device. Under fixed structural and environmental parameters, the breakdown threshold remains essentially constant. Consequently, as the operating voltage increases, the edge electric field intensifies, triggering air breakdown, which leads to a decrease in surface charge density until the edge electric field drops below the breakdown threshold.

This linear relationship has been validated across various TENG configurations in our previous work (*Nat. Commun.* **15**, 4167 (2024)) and this work, including direct-current TENG (DC-TENG) and its variants, as well as alternating-current TENG (AC-TENG) and its variants (**Figure R2**). Furthermore, this work also demonstrates that the optimal operating voltage remains unchanged (**Figure R3**) even when the operating frequency is varied. Together, these results demonstrate the general validity of the linear σ–V decay model.

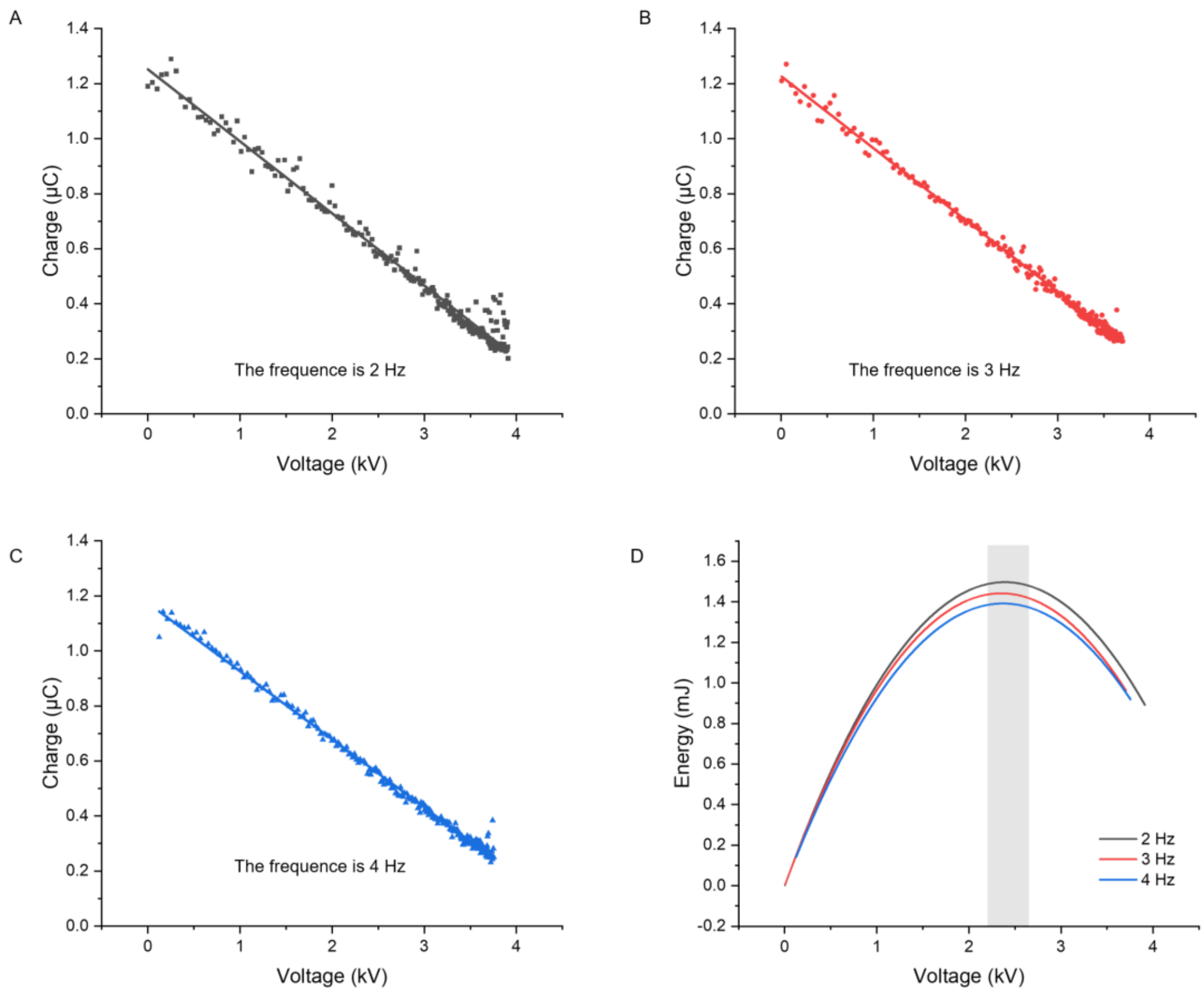

Figure R3. The *Q*-*V* curves of TENG at different frequencies. (A) The *Q*-*V* curve of TENG at 2 Hz. (B) The *Q*-*V* curve of TENG at 3 Hz. (C) The *Q*-*V* curve of TENG at 4 Hz. (D) The corresponding *E*-*V* curves of TENG at different frequencies.

3.The novelty relative to recent dual-loop and charge-pump–buck PMC strategies is not sufficiently differentiated; a concise comparative schematic and performance table against 2022–2024 literature is necessary.

Response: Thank you for your professional comments.

The essence of the dual-loop strategy is to add an additional freewheeling branch into the LC circuit to reduce the energy loss across the freewheeling diode. However, the reported experimental results indicate that this approach offers only limited improvement in efficiency. The optimal efficiency reported in the literature is 88.7% at an output voltage of 50 V; however, when the output voltage drops to 5 V, the efficiency decreases to only 43.1%, which is less than half of the peak value. This demonstrates that the issue of efficiency degradation at low voltages, as discussed in that work, has not been effectively resolved.

Meanwhile, the charge-pump–buck PMC strategy is more suitable for improving TENG performance using a charge pump strategy, and it does not provide any specific optimization to address the efficiency loss of the buck circuit. Therefore, these two works are still essentially single-stage buck strategies.

The multi-stage buck strategy proposed in this work regulates two key node voltages. It successfully addresses both the matching problem between high performance TENGs and the primary circuit and the large efficiency drop of conventional single-stage circuits at output voltages below 5 V. This represents an innovative contribution. Moreover, the current density, power density, and hydrogen production rate achieved at low voltage in this work all reach the highest levels reported in this field.

Revisions

We have supplemented **Table S10** with additional data on power density and circuit efficiency under low-voltage conditions to facilitate comparison for readers.

Table S10 Comparison of circuit design and optimization of different works

	Ref.12	Ref.19	Ref.24	Ref.11	Ref.17	Ref.23	Ref.13	Ref. 25	Ref. 10	This work
TENG-PMC Matching Mechanism	The *V*-*Q* plot based on ideal CMEO theory									The *Q*-*V* curve based on energy cycle collapse theory
TENG-PMC Matching Method	C_{in} extracts and stores energy			TENG's inherent capacitor directly serves as the storage unit.						Regulation the circuit operating voltage based on *Q*-*V* curve
Circuit Architectures	LC	FSCC	LC	LC	Transformer coupled circuit	LC	LC	LC & Commercial chips	Charge-pump circuit & LC	Multi-Stage Buck Strategy
Optimization Strategies	Utilizes SCR to achieve passive switching design	Fractional circuit to reduce voltage drop of diode	Optimized GDT	Optimized inductor	Optimized transformer	Double loops design	Optimized capacitor; Half-wave circuit completely suppresses air breakdown	Reduced charge and voltage losses at switch; Achieved stable voltage output	Half-wave circuit completely suppresses air breakdown	Regulation the intermediate voltage of secondary circuit
Circuit efficiency	67.8% (3.6V) 89.8% (Max Eff.)	62.5% (3.6V) 94.5% (Max Eff.)	50.4% (3.6V) 83.9% (Max Eff.)	45.0% (3.6V) 81.6% (Max Eff.)	55.8% (3.6V) 78.5% (Max Eff.)	37.8% (3.6V) 90.6% (Max Eff.)	46.5% (3.6V) 76.9% (Max Eff.)	N/A	40.5% (3.6V) 52.9% (Max Eff.)	86.3% (3.6 V) 90.9% (Max Eff.)
Power density	0.10 W/m^2	0.04 W/m^2	0.08 W/m^2	0.09 W/m^2	0.29 W/m^2	0.07 W/m^2	0.28 W/m^2	0.63 W/m^2	0.15 W/m^2	10.33 W/m^2
Performance	1. Most optimal circuit efficiencies exceed 80%; however, efficiency significantly decreases below 5V.<br>2. The power density of the TENG used does not exceed 1 W/m^2. Whether it is suitable for high-performance TENG (power density exceeding 10W/m^2) is a question.									1.Efficiency remains around 80% even at low voltage.<br>2.Power density exceeds 10 W/m^2.<br>3.Current density reaches up to 5.19 A/m^2

4.The hydrogen-production validation lacks independent gas-composition confirmation (e.g., GC analysis), which weakens the strength of the electrochemical performance claims.

Response: Thank you for your professional comments.

We performed gas-composition confirmation of the hydrogen generated at the cathode using gas chromatograph with argon as the carrier gas, as shown in **Figure R4**. This result validates the accuracy of the reported hydrogen yield.

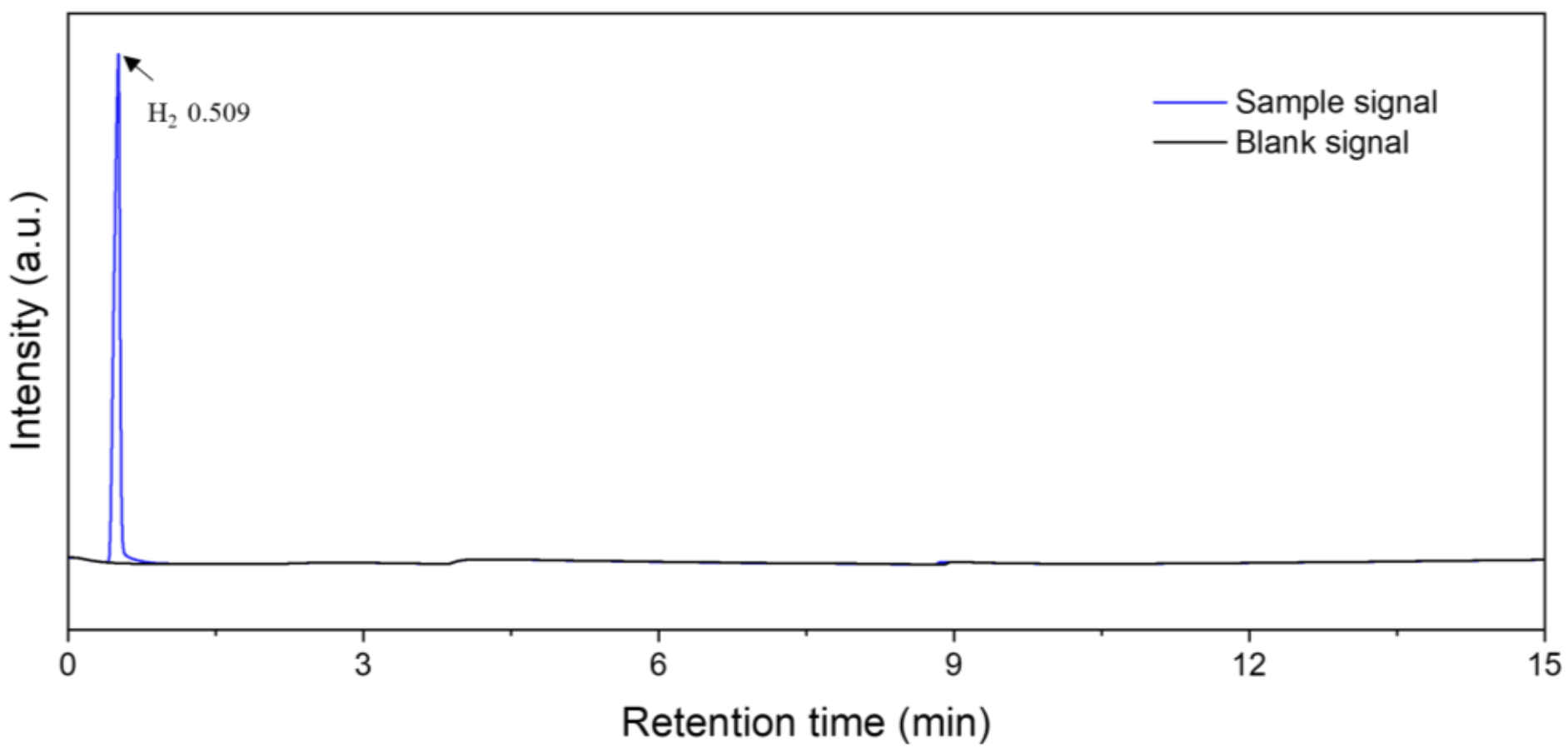

Figure R4 (Figure S39). GC spectrum of H_2.

Revisions

We have added the "Hydrogen gas (10 mL) was collected and analyzed by GC-FID (Agilent 7890B-5977A) using argon (99.999%) as the carrier gas." in the last paragraph of "**Methods**"

We have added **Figure S39** in the Supporting Information.

We have revised "Due to experimental limitations, a detailed quantitative analysis of the gas collected from the anode via gas chromatography (GC) was not performed (chlorine is a corrosive gas that can damage the instrument). In contrast, we performed gas-composition confirmation of the H_2 generated at the cathode with argon as the carrier gas, as shown in **Figure S39**. The repeated measurements for H_2 collection and the GC results confirm that the performance enhancement is robust and results from the proposed strategy in this work, rather than systematic or measurement errors." in the second to last paragraph of **Note S20**.

1. Long-term operational stability and scalability toward sustained higher power density operation are insufficiently demonstrated for practical deployment relevance.

Response: Thank you for your professional comments.

Circuit stability is crucial for the practical application of TENGs. Therefore, all electronic components used in our circuit are rated to meet the required voltage, current, and power conditions to ensure sustained operation. In addition, we extended the stability test to 24 hours of continuous operation, during which the circuit operated reliably without observable performance degradation (**Figure R5**).

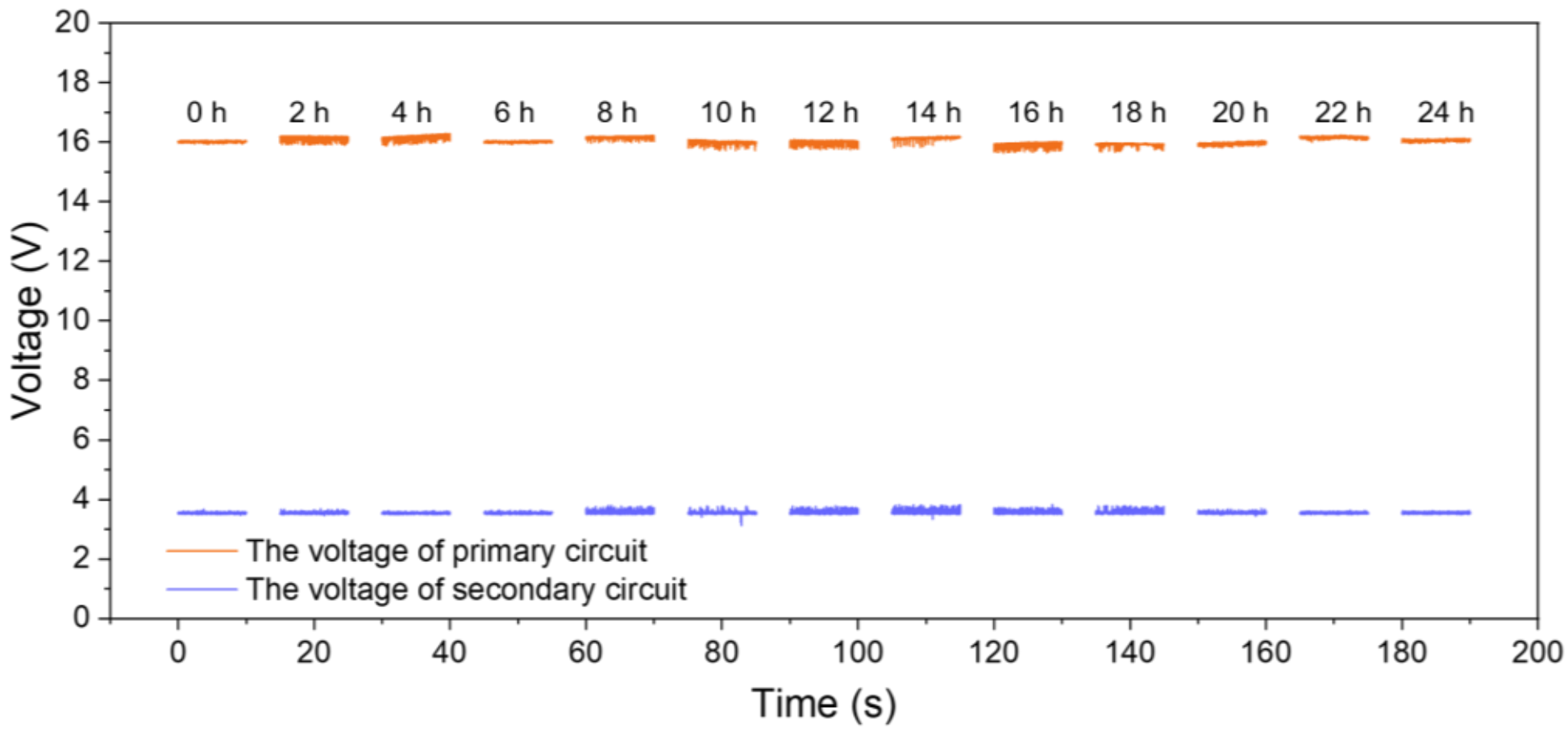

Figure R5 (Figure S25A). The output voltage of the circuit after 24 hours of continuous operation

The strategy reported in this work has been successfully validated in three different device configurations: rolling-mode TENG, a rabbit fur/PTFE-based rotary-mode TENG, and a PA/PTFE-based rotary-mode TENG. In all three cases, the power density was progressively improved through iterative design improvements, with the highest power density exceeds 10 W m^{-2}. Moreover, most of the existing devices with higher power densities are variants of the sliding-mode AC-TENG, which inherently suffer from air breakdown at the electrode edges (as we discussed in our response to **Comment #2**). Our theoretical model directly addresses this phenomenon and is applicable to such device types.

Revisions

We have revised **Figure S24** in the Supporting Information

6.The impact of parasitic elements, switching losses, and thermal behavior in the high-voltage primary stage is not quantitatively analyzed, which may affect real-world efficiency.

Response: Thank you for your professional comments.

1. Parasitic resistance:

In our circuit simulation, a parasitic resistance of 1.82 Ω was explicitly included to account for ohmic losses. As the output voltage increased from 0.5 V to 16 V, the energy dissipated in this parasitic resistor decreased from 228 μJ to 32 μJ (**Figure R6**). This trend confirms that parasitic resistance loss is more significant at low output voltages. Therefore, the combination of circuit parasitic resistance and the voltage drop across the diode together causes the efficiency reduction at low voltages in current single-stage circuits. As shown in the **Figure R6**, the relevant content has already been presented in **Figure S16**.

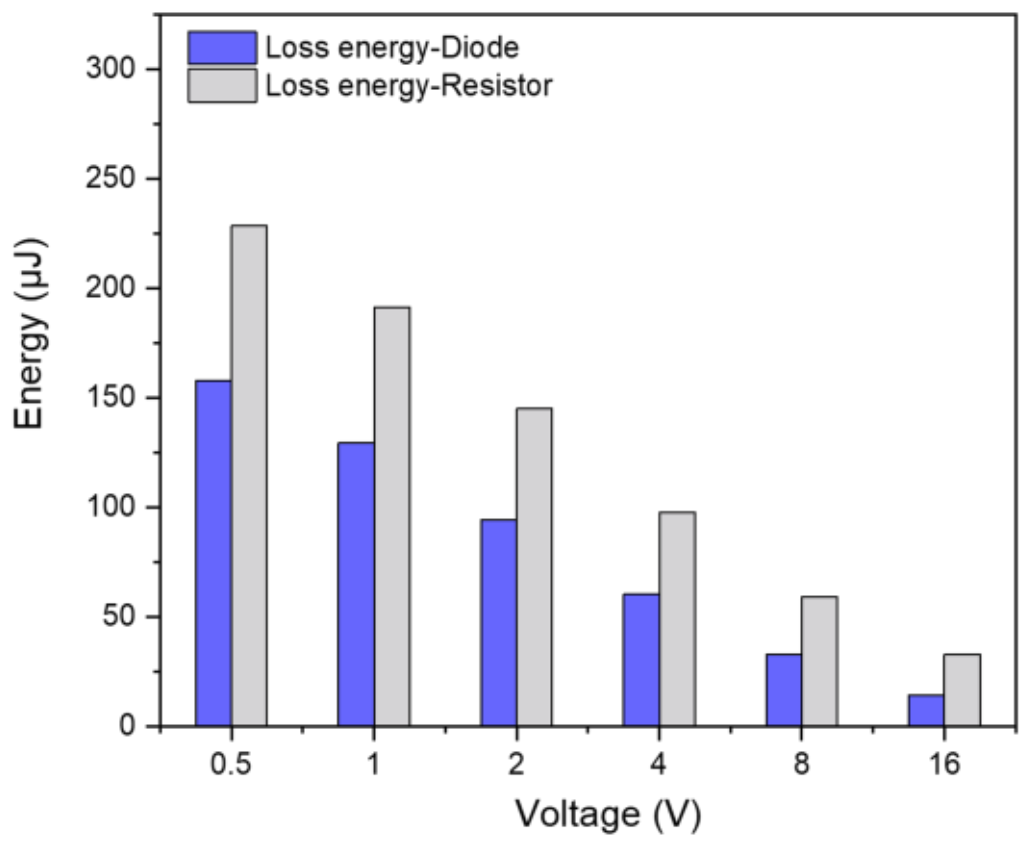

Figure R6. The circuit simulation results for loss energy of resistor and one diode.

2. Switching device (GDT) parasitic parameters:

We acknowledge that the gas discharge mechanism in a GDT is complex, making it challenging to construct a highly accurate physical model. Nevertheless, based on the manufacturer's datasheet, we adopted a simplified model: a parasitic capacitance of 1.5 pF and an on-state resistance of 10 Ω (**Figure R7A**). The simulation shows that the energy loss of GDT associated with these parasitic parameters is only 8.2 μJ (**Figure R7B**). Compared with the diode voltage drop and the circuit parasitic resistance, the loss at the switch is not the dominant cause of the efficiency reduction at low voltages. However, further optimization of the switch to reduce this loss could further improve the efficiency in practical applications.

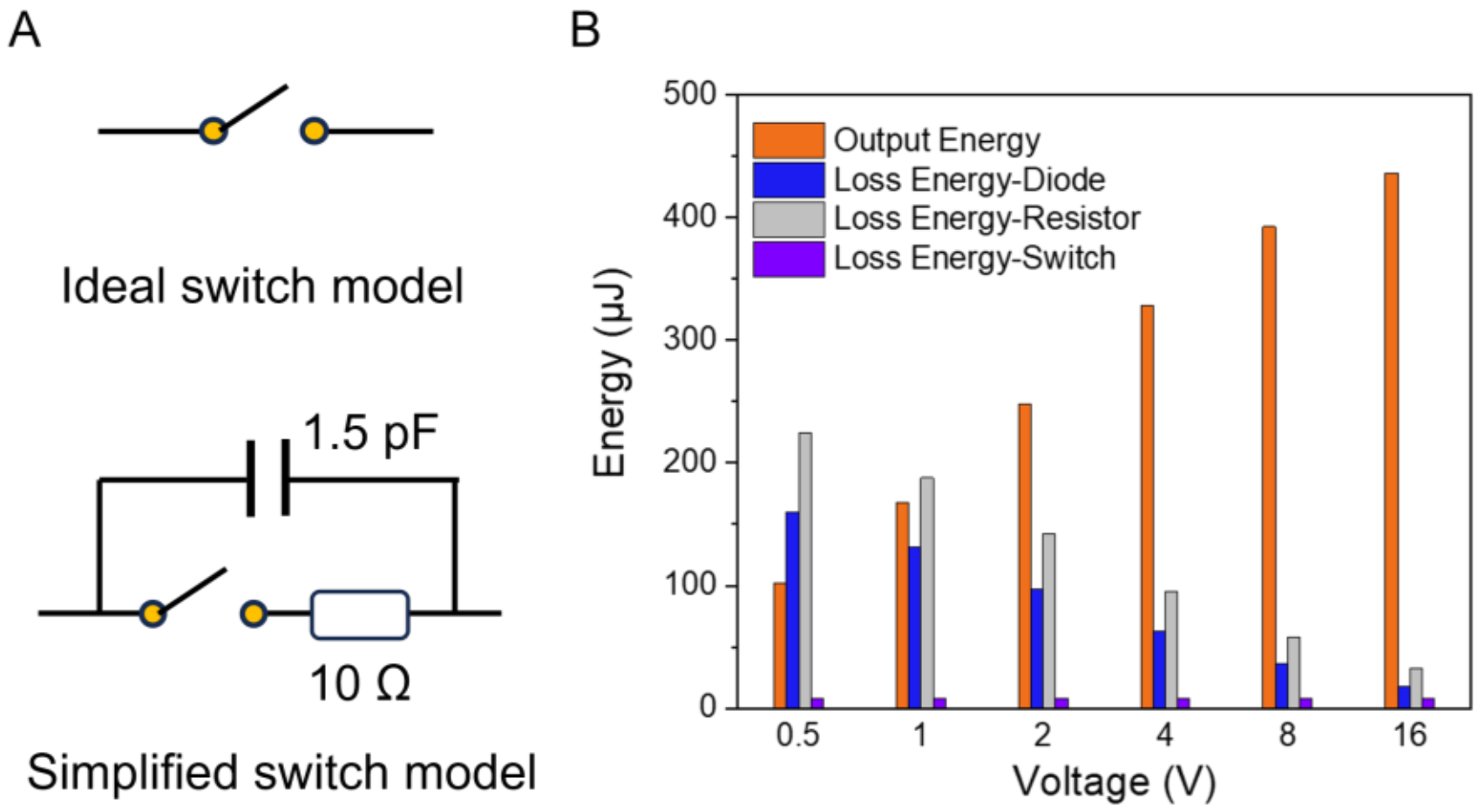

Figure R7 (Figure S17). Schematic diagram of a simple circuit model of a gas discharge tube (GDT) switch and its energy loss simulation. (A) Schematic diagram of a simple circuit model of GDT; (B) The results of energy loss simulation.

3. Thermal behavior:

We operated the circuit continuously for four hours under ambient conditions (30 ℃, 40% RH) and recorded infrared thermographs (**Figure R8**). The temperature rises on the inductor, capacitor, and GDT were all below 1 ℃, confirming negligible self-heating during this period.

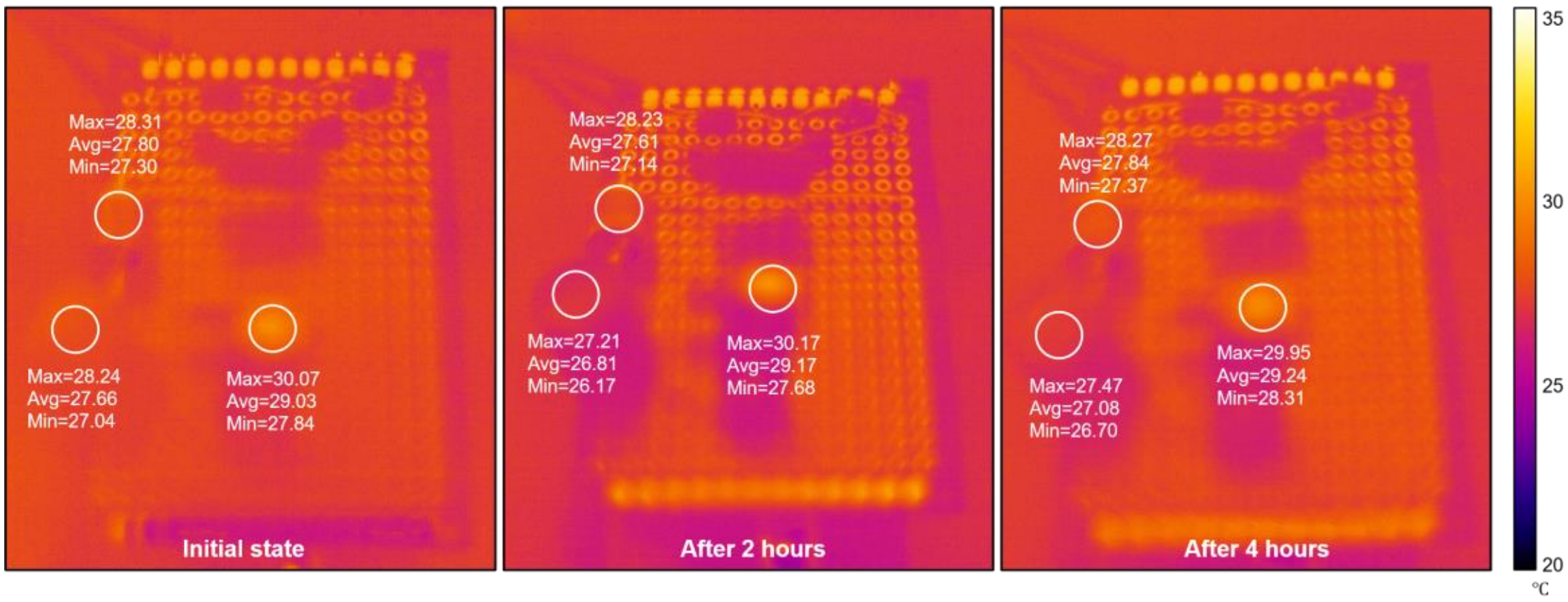

Figure R8 (Figure S25B). Thermal image of the primary circuit after four hours of continuous operation.

In addition, we varied the operating temperature from 30 ℃ up to 70 ℃ and then back to 30 ℃ while monitoring the output voltage under a fixed load. The output voltage changed from 4.60 V to 4.51 V (a decrease of ~2.0%) at 70 ℃ and returned to 4.60 V after cooling (**Figure R9**). This small and reversible change indicates that the circuit efficiency is only weakly affected by temperature variations within this range.

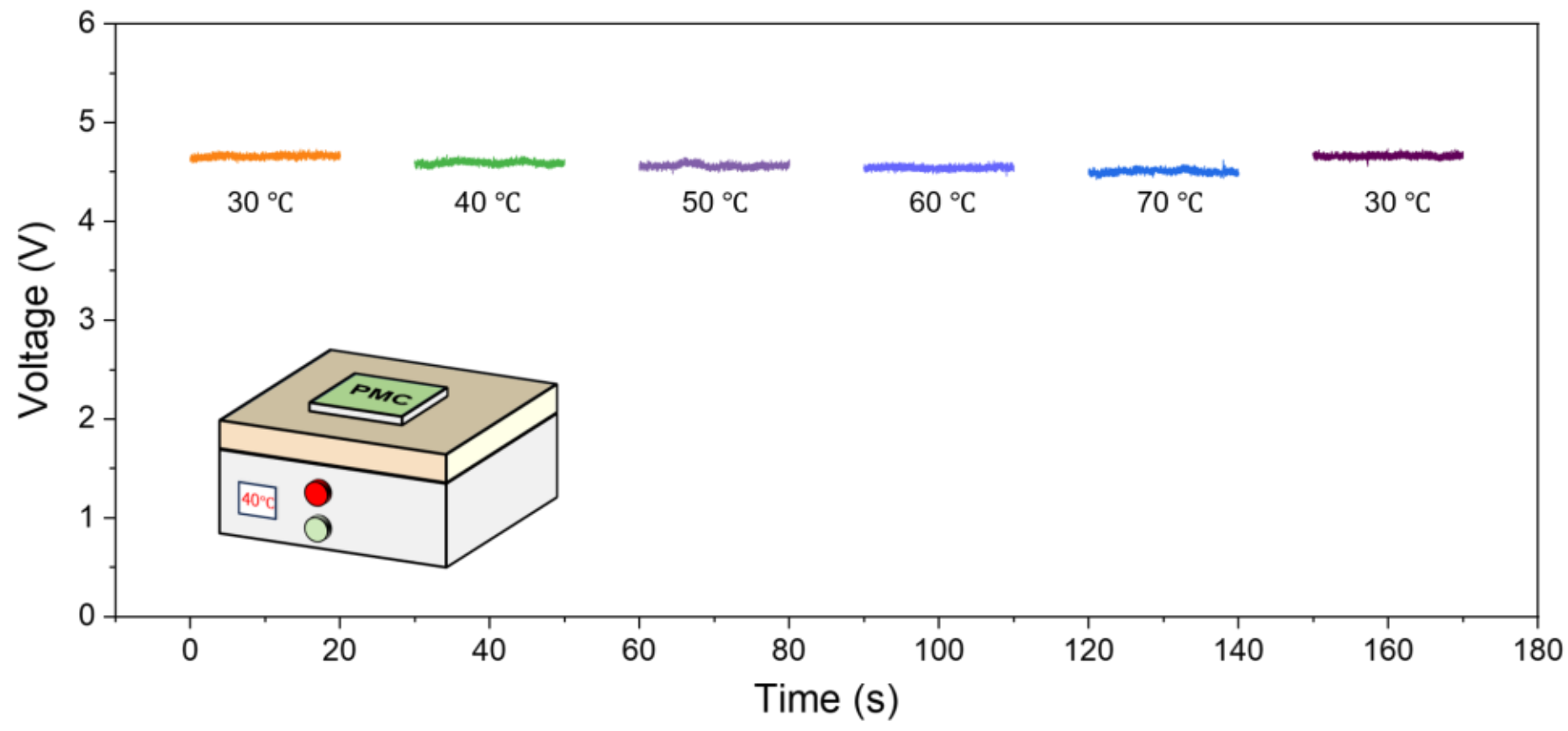

Figure R9 (Figure S25C). Output voltage of primary circuit variation with temperature.

Revisions

We have revised "The parameters are as follows: input capacitance (C_{in}) 440 pF, output capacitance (C_{out}) 10 mF, inductance (L) 1 mH, switch threshold voltage 1500 V. The circuit internal resistance (R_{int}, mainly from the inductor) is 1.82 Ω, and the freewheeling diode forward voltage drop (V_{diode}) is 0.83 V (increasing the number of diodes to improve voltage drop). Although a precise GDT switch model was not established, the simulation results of the simplified switch model built based on the parasitic parameters of the GDT indicate that the energy loss caused by the switch is much smaller than that caused by the resistor and the diode (**Figure S17**). Therefore, it does not affect our analysis, and this model still effectively analyzes the impact of V_{diode} and R_{int} on energy loss" in the **Note S13**.

We have added the thermal analysis of the primary circuit to the Supporting Information as **Figure S25**.

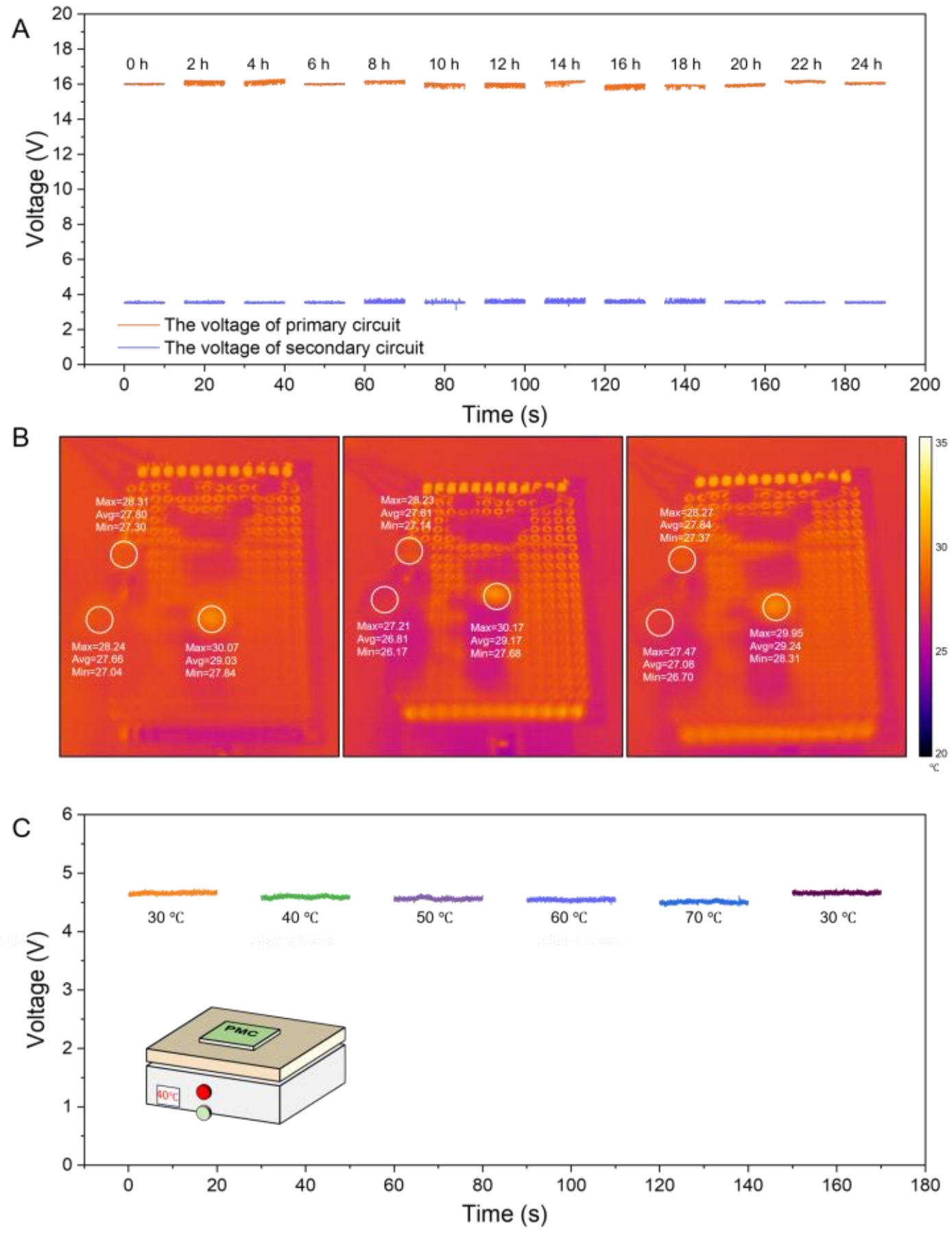

Figure S25. The long-term stability and thermal behavior of the circuit. (A) The output voltage of the circuit after 24 hours of continuous operation. (B) Thermal image of the primary circuit after four hours of continuous operation. The temperature rises on the inductor, capacitor, and GDT were all below 1 °C, confirming negligible self-heating during this period (30 °C, 40% RH). (C) Output voltage of primary circuit variation with temperature. The output voltage changed from 4.60 V to 4.51 V (a decrease of ~2.0%) at 70 °C and returned to 4.60 V after cooling.

Reviewer #6 (Remarks to the Author):

The authors have made substantive revision to the manuscript and addressed the major concerns raised by the reviewers. In particular, the authors now include a detailed benchmarking against other power-management configurations, which substantiates their claim that the proposed multi-stage buck strategy offers a low-voltage performance advantage over these other configurations. Further, the inclusion of repeated efficiency measurements with statistical variation, additional validation on a sliding-mode TENG, and expanded analysis of energy extraction efficiency has improved the rigor, scope, and reproducibility of the work. Overall, I consider the present version suitable for publication.

Response: Thank you very much for your positive and encouraging comments on our revised manuscript. We greatly appreciate your recognition that the present version is suitable for publication.
